# From Acyclic Intramolecular-[4 + 2]- to Transannular Bis-[4 + 2]-Cycloaddition of the Macrodiolide for the Stereoselective Synthesis of the Octahydronaphthalene Core of Polyenic Macrolactam Sagamilactam

**DOI:** 10.1021/acs.orglett.4c02239

**Published:** 2024-07-30

**Authors:** Oscar Iglesias-Menduiña, Diego Novegil, Claudio Martínez, Rosana Alvarez, Angel R. de Lera

**Affiliations:** CINBIO, Departamento de Química Orgánica, Universidade de Vigo, 36310 Vigo, Spain

## Abstract

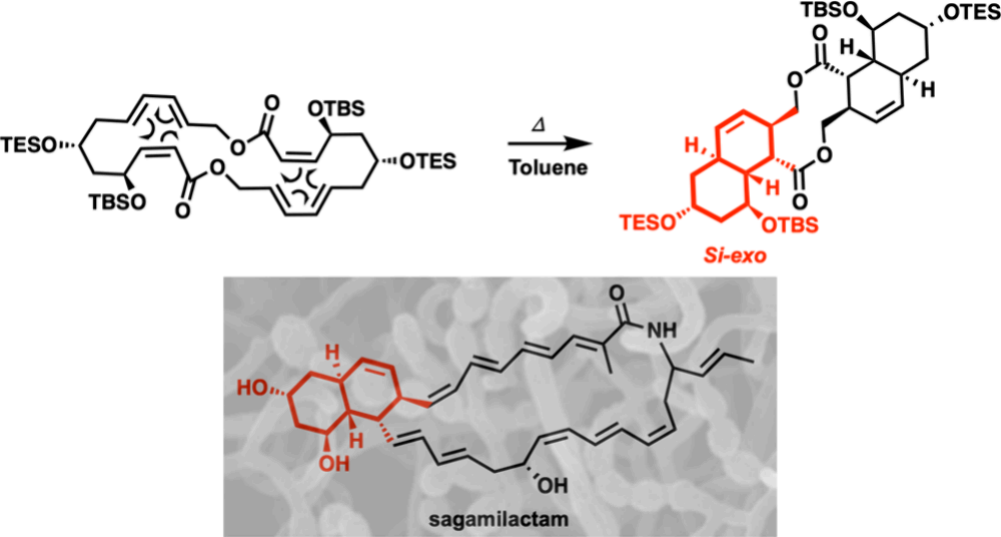

The strategy for the synthesis of the octahydronaphthalene core of natural macrolide sagamilactam has unintentionally evolved from the acyclic intramolecular (IMDA) to the transannular (TADA) Diels–Alder reaction. Lewis acid-promoted IMDA of a protected 2*Z*,8*E*,10*E*-4,6,12-trihydroxy-2,8,10-decatrienal model with a diol of 4,6-*anti* relative configuration, as proposed by DP4+-based computational studies, afforded the *cis*-octahydronaphthalene diastereomer through the *Re-endo* approach. The 26-membered macrodiolide generated, under thermal reaction conditions, the *trans*-octahydronaphthalene by a double TADA reaction along the desired *Si-exo* orientation.

Polyenic macrolactam sagamilactam (**1**) was isolated in 2016 after bioassay-guided fractionation of the culture broth of *Actinomadura* sp. K13–0306 bacteria present in a soil sample collected in Kanagawa Prefecture, Japan, and exhibited potent activity (IC_50_ = 0.14 ± 0.06 μg/mL or 0.25 ± 0.11 μM) against the parasitic protozoan *Trypanosoma brucei* GUTat 3.1 strain.^1^

The structural characterization of tricyclic sagamilactam (**1**) relied on the interpretation of NMR data, which revealed a main *trans*-octahydronaphthalene core^2^ with four additional stereocenters, fused to a 26-membered macrolactam. The latter contains two arms with polyunsaturated substructures, namely a conjugated 2*E*,4*E*,6*E*,8*Z*-tetraenoate and a nonconjugated 20*E*,22*E*,26*Z*,28*E*,30*Z*-pentaene, as well as an (*E*)-1-propenyl substituent at the secondary amine carbon, a methyl group vicinal to the unsaturated amide carbonyl, and a hydroxyl group at the allylic C25-position. However, the relative configurations of the secondary alcohol at C15 and the amine-substituted carbon C33, and therefore the absolute configuration of sagamilactam (**1**), could not be determined.^1^

The biogenesis of the octahydronaphthalene moiety^2,3^ of sagamilactam (**1**) was suggested^1,4^ to involve an intramolecular Diels–Alder reaction (IMDA)^5^ of a 34-membered macrolactam precursor (**2**), namely a transannular Diels–Alder (TADA) reaction.^6^ According to the biogenetic proposal,^1^ the relative configuration of the chiral centers at the octahydronaphthalene core of sagamilactam generated by the diastereoselective TADA reaction could be promoted by a Diels–Alderase enzyme, and the outcome would require a *Si*-*exo* relative orientation of the reacting components (see descriptors *Re/Si* for heterotopic faces^7^ in S.I.) with formal 10*E*,12*E*,18*Z* geometries as part of the 34-membered macrolactam (**2**).^3^ Diels–Alderases from several natural sources have been structurally and functionally characterized,^8^ and grouped under the general family term “biosynthetic pericyclases”.^9^

In this context, our prior approach to the dihydroxyoctahydronaphthalene core of nahouic acid A based on the IMDA of the corresponding acyclic precursors, and the total synthesis of the natural product,^10^ inspired us to address as synthetic target the more challenging octahydronaphthalene skeleton of sagamilactam (**1**).

## Results and Discussion

**Figure 1:**
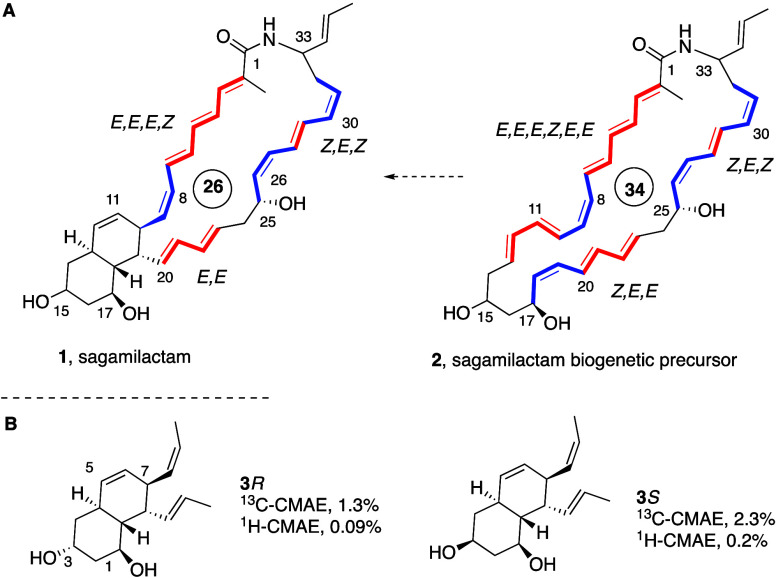
**A**. Proposed structure of sagamilactam (**1**) and the putative biogenetic precursor (**2**).^1^**B**. ^1^H-CMAE and ^13^C-CMAE values for the NMR simulations [B3LYP/6-31+G**-PCM(MeOH)] of **3**.

Given the uncertainty on the relative configuration at C15 of sagamilactam (**1**), we first decided to predict the corresponding NMR chemical shift values through computations on model systems **3***R* and **3***S* (Figure 1B) with a customizable DP4+ methodology developed by Sarotti and co-workers^11^ and compare them with the reported NMR data.^1^ DFT-based structural optimization using the Gaussian 16 suite of programs^12^ at the B3LYP/6-31+G**-PCM(MeOH) level was followed by GIAO (Gauge-Including Atomic Orbitals) NMR calculation of isotropic shielding values and translation of these values to scaled chemical shifts. The corrected mean absolute error (CMAE) computed for proton and carbon are shown in Figure 1B. The smaller CMAE for the **3***R* diastereomer suggested the relative configuration at C3 for the natural product model system. Lastly, the NMR simulations were incorporated in the DP4+ probability tool with the probability distribution terms,^11^ which confirmed the results and supported the *R* relative configuration at C3 (99.97% for ^1^H NMR and 99.98% for ^13^C NMR) of model system **3** and, by extension, the *R* configuration at C15 for the sagamilactam octahydronaphthalene structure.

**Scheme 1:**
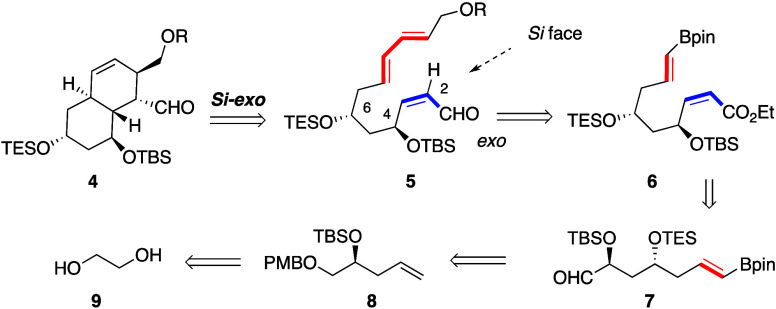
Retrosynthetic analysis of the (Protected) Dihydroxyoctahydronaphthalene Model System **4**

Adhering to the IMDA biogenetic proposal, and in order to understand the structural factors likely responsible for the diastereoselection on the proposed biogenesis of sagamilactam (**1**), we focused on model system **5** containing a protected nonconjugated 2*Z*,8*E*,10*E*-4,6,12-trihydroxydodecatrienal skeleton, with the expectation that it would generate the octahydronaphthalene core of **4** by a stereoselective cycloaddition following the *Si*-*exo* approach (Scheme 1, see S.I.) of the reacting components through chair like TSs.^5^ As a simple target-oriented retrosynthetic analysis (Scheme 1), the IMDA precursor dodecatrienal **5** could be generated by Suzuki–Miyaura cross-coupling reaction of **6** following stereoselective olefination from aldehyde **7**, which was proposed to be generated from ethylene glycol **9** by a sequence of Krische’s allylation reactions promoted by enantiopure biphosphine ligands through homoallylic alcohol **8** followed by oxidative cleavage.^13^

**Scheme 2:**
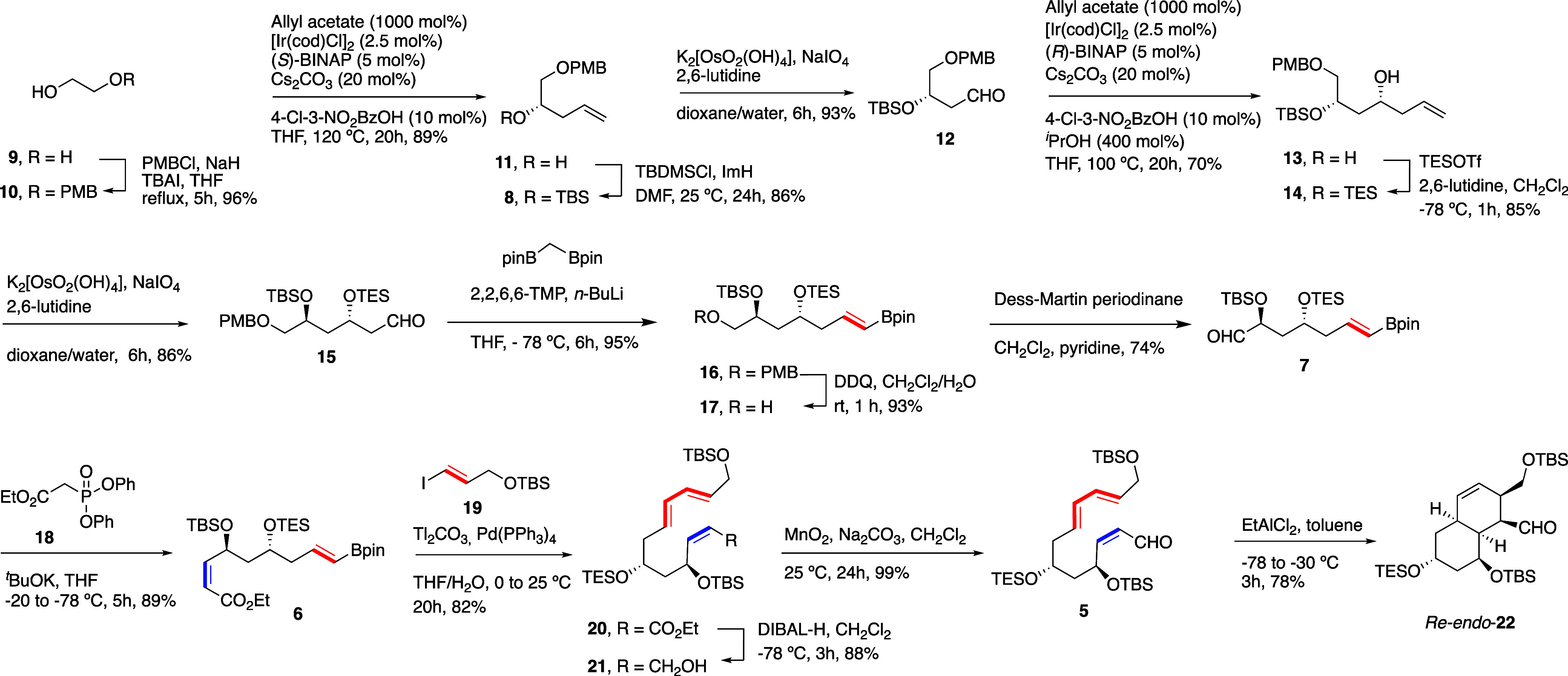
Synthesis of (2*Z*,4*S*,6*R*,8*E*,10*E*)-4,6,12-Trihydroxydodeca-2,8,10-trienal **5** and IMDA

The *S* enantiomer (corresponding to C17 of natural sagamilactam **1**, Figure 1A) of the homoallylic alcohol **11**(14) was prepared in high yield by consecutive oxidation of monoprotected ethylene glycol **10** and transfer of an allyl group^15^ from allyl acetate promoted by [Ir(cod)Cl]_2_ and (*S*)-BINAP^13^ (Scheme 2) (for less-efficient alternative methods, see S.I.). Enantioselectivity (*ee* = 87%) was determined by chiral HPLC and confirmed (see S.I.) by formation of the diastereomeric esters with (*R*)- and (*S*)-MTPA in the presence of DCC and DMAP.^16^

Aldehyde **12**(17) was efficiently obtained by protection of homoallylic alcohol **11** and Lemieux–Johnson oxidative cleavage of **8**.^18^ The synthesis of the protected *anti*-1,3-diol used a diastereoselective Krische’s allylation reaction^13^ of **12** but with (*R*)-BINAP and at lower temperature (100 °C), which provided homoallylic alcohol **13** in 70 yield. Diastereoselectivity was determined by ^1^H NMR analysis of the cyclic acetals generated after deprotection of **13** (see S.I.). For the protection of the alcohol, we opted for the more labile triethylsilyl ether, which eventually could allow to selectively deprotect that functionality and invert the configuration if required. Oxidative cleavage of **14** as described above and treatment of the resulting aldehyde **15** with bis[(pinacolato)boryl]methane, 2,2,6,6-tetramethylpiperidine (2,2,6,6-TMP) and *n*-BuLi according to the protocol of Morken,^19^ generated in 95% yield the alkenylboronate **16**. Deprotection of the primary alcohol of **16** under oxidative conditions, followed by oxidation of **17** with Dess–Martin periodinane afforded aldehyde **7** (Scheme 2). Stereoselective HWE condensation reaction of aldehyde **7** with the anion of Ando’s ethyl diphenylphosphonoacetate **18**(20) afforded **6** diastereoselectively in high yield (89%). The Suzuki–Miyaura cross-coupling reaction of alkenylboronate **6** with protected 3-iodoprop-2-en-1-ol **19**(21) provided nonconjugated ethyl trienoate **20** in 82% yield. Uneventful DIBAL-H reduction and oxidation of the allylic alcohol **21** with MnO_2_/Na_2_CO_3_ led to the desired trienal **5** in high yields.

Acyclic model system **5** generated an octahydronaphthalene derivative as major product (78% yield) only under EtAlCl_2_-promoted IMDA reaction conditions, which was characterized as the *cis* diastereomer *Re-endo*-**22** (see S.I.). A complex mixture of products in low yields was detected by ^1^H NMR analysis of the reaction mixture upon thermal treatment of **5**.^22^

Given the failure to selectively obtain the targeted *Si-exo* diastereomers by the IMDA^5^ of trienal **5**, a TADA reaction^5,6^ was considered as alternative approach starting from the corresponding macrocyclic compound. However, precedents in the literature indicate that the thermal IMDA of nonconjugated 2*Z*-trienals or methyl 2*Z*-trienoates (with different substituents) and TADA reactions of their macrolides proceeded with similar stereochemical outcomes.^23^ Mixtures of diastereomers in contrasting *exo/endo* diastereomeric ratios were obtained as a function of the substitution pattern.^23^ Even subtle structural differences, in particular the presence of a methyl substituent at the internal position of the diene, were found to play a major role on the stereoselectivity of the IMDA and TADA cycloadditions, with preferential formation of the *Si-endo*(23a) or *Si-exo* diastereomers,^23b^ for the 2*Z*-unsaturated systems on each of these structurally similar variants.

Being also aware of the structural differences between the reacting triene units of our system and the reported TADA reactions that afforded the *Si-exo* diastereomers,^23b^ we nevertheless decided to study the cycloaddition reactions of the corresponding macrolactone. To ensure the formation of the *Z*-unsaturated macrolide, recourse was also made to the intramolecular Ando’s variant of the modified HWE condensation^20b^ and therefore to previously prepared alkenyl pinacolboronate **17** and *E*-iodoallylphosphorylethanoate **28**.

**Scheme 3:**
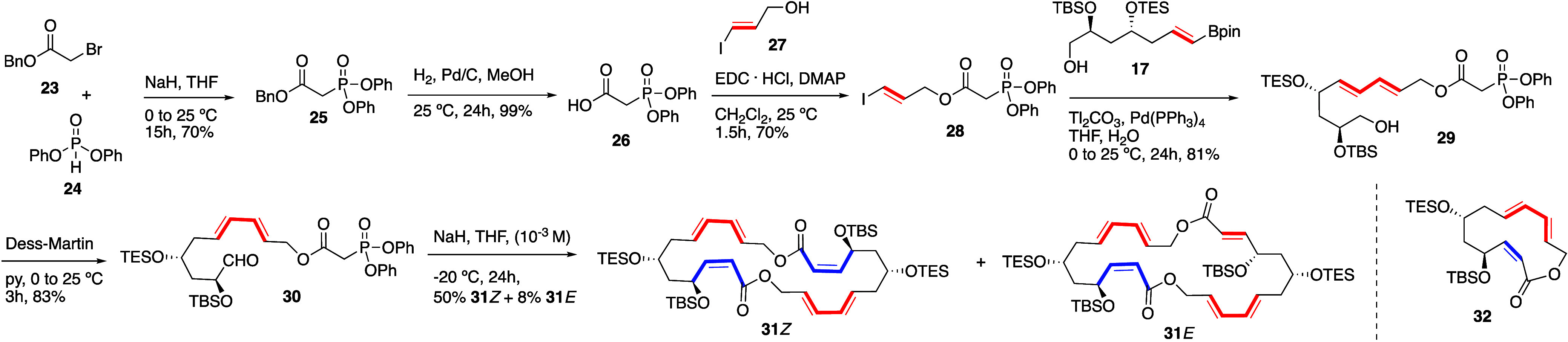
Synthesis of Macrodiolide **31**

The synthesis of **28** (Scheme 3) started with the quantitative deprotection of the benzyl ester of **25**, itself obtained by reaction of the bromomethyl benzyl ester **23** with the deprotonated H-phosphonate **24**, and condensation of the so-generated carboxylic acid **26** with (*E*)-3-iodopropenol **27**(21) promoted by EDC·HCl and DMAP. Suzuki–Miyaura cross-coupling of alkenylpinacol boronate **17** and alkenyl iodide **28** provided hydroxyester **29** (81% yield), and oxidation of the primary alcohol with Dess–Martin periodinane afforded functionalized trihydroxyundecadienal **30** (83% yield).

Surprisingly, the intramolecular HWE reaction using Ando’s variant (NaH, THF, −20 °C)^20^ under diluted conditions (10^–3^ M) provided the 26-membered macrodiolide **31***Z* in 50% yield, together with the *Z*,*E* isomer at the enoate bond **31***E* (8% yield), instead of the expected 13-membered macrolactone **32** (Scheme 3). The dimeric structure of **31***Z* and **31***E* was inferred from analysis of the MS data (see S.I.).

**Scheme 4:**
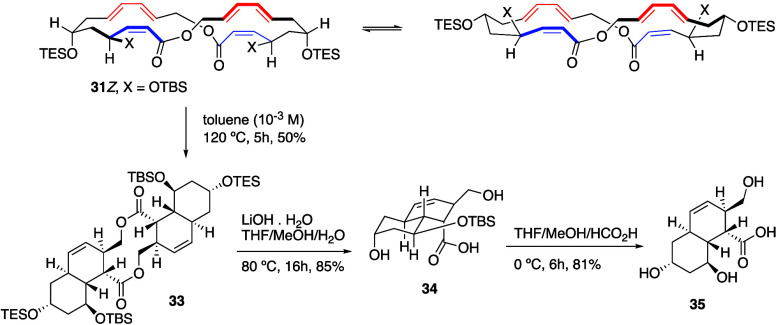
Double TADA Cycloaddition of 26-Membered Macrodiolide **31***Z* to Octahydronaphthalene-Fused 10-Membered Macrodiolide **33** and Synthesis of Octahydronaphthalene **35**

The formation of macrodiolides akin to **31** but with *E*,*E*,*E* geometries has previously been reported by Nicolaou et al.^24^ upon attempted macrolactone formation. A double transannular IMDA reaction,^6^ resulting from the *Re-exo* cycloaddition was obtained under thermal reaction conditions. Based on these precedents, heating toluene solutions of 2*Z*-macrodiolide **31***Z* with catalytic amounts of BHT at 120 °C for 5 h generated a major product (**33**) in 58% yield. A partial octahydronaphthalene structure could be determined for **33** upon interpretation of the NMR spectroscopic data (Scheme 4). Analysis of NOE data based on the relative and absolute configurations of the diol fragment suggested the formation of the *Si*-*exo* diastereomer of the decalin cores within the pentacyclic skeleton of **33**.

Confirmation of the relative and absolute configurations of the newly generated stereocenters of macrodiolide **33** rested on X-ray diffraction analysis (see S.I.), which supported the presence of two *trans*-octahydronaphthalenes fused to the 10-membered macrodiolide as the structure resulting from a double intramolecular/transannular Diels–Alder reaction (TADA)^6^ along the desired *Si-exo* orientation of the reacting components.

The contrasting results experimentally obtained on the IMDA^5^ and TADA reactions^6^ of the formal *anti*-4,6-diol diastereomers (Schemes 2 and 4) confirmed the assumption that the diastereoselectivity of the cycloaddition reaction could derive from a combination of transannular steric repulsion and electronic interactions on a transition state in which the conformation of the macrocycle is governed by the preferred location of the oxygen substituents at the allylic positions.^6^

The octahydronaphthalene core of sagamilactam (**1**) was obtained from macrodiolide **33** after saponification with LiOH, which was accompanied by deprotection of the labile triethylsilyl ether to dihydroxy acid **34** (85% yield), and further removal of the *tert*-butyldimethylsilyl protecting group to trihydroxy acid **35** (Scheme 4).

Comparison of the computed NMR spectroscopic data with those of the fully deprotected triol **35** and structurally related analogues confirmed the prediction on the relative configuration at C15 of sagamilactam already described (see S.I.).

To summarize, a synthetic approach to the octahydronaphthalene core of sagamilactam has been developed based on a macrolactonization/dimerization followed by a stereoselective intramolecular bis-[4π + 2π]-cycloaddition of a 26-membered macrodiolide with an *anti*-4,6-diol relative configuration. Whereas the intramolecular cycloaddition of the acyclic counterpart afforded the octahydronaphthalene resulting from the *Re*-*endo* relative orientation of the reacting components, the double transannular Diels–Alder reaction diverted the cycloaddition diastereocontrol along the desired *Si-exo* manifold and forged the highly substituted bis-*trans*-octahydronaphthalene fused to the 10-membered ring macrodiolide with the proper relative and absolute configuration of sagamilactam, as previously predicted by computations. The results pave the way to construct the natural product and the proposed 34-membered macrolactam precursor. Achieving this challenge would allow to enquire about the putative biogenesis of sagamilactam using Diels–Alderase enzymes.

## Data Availability

The data underlying this study are available in the published article and its Supporting Information.

## Supporting Information Available

Experimental procedures, computational data, summary of X-ray crystallography reports, and NMR spectra (PDF)
