# Correction to “From Acyclic Intramolecular-[4 + 2]- to Transannular Bis-[4 + 2]-Cycloaddition of the Macrodiolide for the Stereoselective Synthesis of the Octahydronaphthalene Core of Polyenic Macrolactam Sagamilactam”

**DOI:** 10.1021/acs.orglett.4c03915

**Published:** 2024-10-28

**Authors:** Oscar Iglesias-Menduiña, Diego Novegil, Claudio Martínez, Rosana Alvarez, Angel R. de Lera

**Affiliations:** CINBIO, Departamento de Química Orgánica, Universidade de Vigo, 36310 Vigo, Spain

A funding statement needs to be added.

