# Supplementary material for: From Acyclic Intramolecular-[4 + 2]- to Transannular Bis-[4 + 2]-Cycloaddition of the Macrodiolide for the Stereoselective Synthesis of the Octahydronaphthalene Core of Polyenic Macrolactam Sagamilactam
Source: Org Lett. 2024 Jul 30;26(31):6614–8. doi: 10.1021/acs.orglett.4c02239 (PMC11472481; doi:10.1021/acs.orglett.4c02239)

Supporting Information (S.I.)

FROM ACYCLIC INTRAMOLECULAR-[4+2]- TO TRANSANNULAR BIS-[4+2]-CYCLOADDITION OF THE MACRODIOLIDE FOR THE STEREOSELECTIVE SYNTHESIS OF THE OCTAHYDRONAPHTHALENE CORE OF POLYENIC MACROLACTAM SAGAMILACTAM

Oscar Iglesias-Menduiña,[1] Diego Novegil,[1] Claudio Martínez,[1] Rosana Alvarez,[1]* and Angel R. de Lera[1,]*

[1]CINBIO, Departamento de Química Orgánica, Universidade de Vigo. 36310 Vigo, SPAIN

Email: qolera@uvigo.es, rar@uvigo.es

Table of contents

1. General remarks 3

1. IMDA. Prochirality and descriptors for prostereoisomerism-prochirality 5

2. TADA reactions 7

3. Less-efficient alternative methods to obtain (S)-2-(tert-butyldimethylsilyloxy)-1-(4-methoxybenzyloxy)pent-4-ene 8 9

4. Synthesis of Mosher's esters (R)-S10 and (S)-S10 10

5. Synthesis of acetonide S11 11

6. Synthesis of alkenyliodide 19 12

7. Experimental procedures 13

8. X-Ray structure of compound 33 41

9. DFT computational methods and computations on the octahydronaphthalene diastereomers 43

10. References 57

11. Copies of NMR spectra 59

1. General remarks

Solvents were dried using a Puresolv™ solvent purification system. All other reagents were commercial compounds of the highest purity available. If not specified, all reactions were carried out under an argon atmosphere. Those not involving aqueous reagents were carried out in oven-dried glassware. For reactions that require heating, a metallic heating block was used and the indicated is the external temperature. All solvents and anhydrous solutions were transferred through syringes and cannulae previously dried in the oven for at least 12h and kept in a dessicator. Analytical TLC was performed on aluminium plates with Merck Kieselgel $60F_{254}$ and visualized by UV irradiation (254 nm) or by staining with a solution of phosphomolybdic acid in ethanol. Flash column chromatography was carried out using Merck Kieselgel 60 (230–400 mesh) with a CombiFlash® Rf Teledyne Isco. The enantiomeric excess was determined by chiral-HPLC using a Waters system comprised of a 515 Waters pump, a PDA detector (Waters 996) and a 717 Plus Autosampler Waters injector; the column was a Chiralcel OD-H (150 x 4.6 mm, 5 μm) and the mobile phase was an isocratic elution at 0.7 mL/min with a mixture of eluents A:B:C = 95.5:4:0.5 (v/v), being eluent A, *n*-hexane; eluent B, isopropanol and eluent C, CH_2Cl_2.

HRMS (ESI$^+$) were measured with an FT-ICR-MS Solarix 7T mass spectrometer (Bruker Daltonics). ^{1}H-NMR spectra were recorded in $CDCl_3$, C_6D_6, $(CD_3)_2CO$ and CD_3OD at 293 K or 323 K with a Bruker AMX-400 spectrometer operating at 400.16 MHz with residual protic solvent as the internal reference [$CDCl_3$, δ = 7.26 ppm; C_6D_6, δ = 7.16 ppm; CD_2Cl_2, δ = 5.32 ppm; $(CD_3)_2CO$, δ = 2.05 ppm, CD_3OD = 3.31 ppm]; chemical shifts (δ) are given in parts per million (ppm) and coupling constants (*J*) are given in Hertz (Hz). The proton spectra are reported as follows: δ (multiplicity, coupling constant *J*, number of protons). ^{13}C-NMR spectra were recorded in $CDCl_3$, C_6D_6, $(CD_3)_2CO$ and CD_3OD at 293 K or 323 K and with the same spectrometer operating at 100.63 MHz with the central peak of $CDCl_3$ (δ = 77.26 ppm), C_6D_6 (δ = 128.06 ppm), $(CD_3)_2CO$ (δ = 206.26 ppm) and CD_3OD (δ = 49.00 ppm) as the internal reference. DEPT-135 pulse sequences and HSQC bidimensional NMR spectra were used to aid in the assignment of signals in the ^{13}C- and ^{1}H-NMR spectra. ^{31}P-NMR spectra were recorded in $CDCl_3$ and C_6D_6 at 298 K and with the same spectrometer operating at 162 MHz with the peak of 85% H_3PO_4 in H_2O (δ = 0.0 ppm) as the reference. Multiplicity and ^{13}C-^{31}P coupling constants are indicated in parenthesis after the ^{13}C-^{1}H multiplicity. Different NOE-1D experiments were also performed in selected cases. Infrared spectra (IR) were obtained on a JASCO FT/IR-4200 infrared spectrometer, from a thin film

deposited onto a NaCl glass. IR data include only characteristic absorptions. Peaks are quoted in wave numbers (cm^{-1}), and their relative intensities are reported as follows: s = strong, m = medium, w = weak. Specific optical rotations were measured on a JASCO P-1020 polarimeter with a Na lamp (glass cell, 3.5 x 100 mm). The crystallographic data were collected at 100 K using a Bruker D8 Venture diffractometer with a Photon 100 CMOS detector and Mo-Kα radiation (λ = 0.71073 Å) generated by an Incoatec high brilliance microfocus source equipped with Incoatec Helios multilayer optics.

1. IMDA. Prochirality and descriptors for prostereoisomerism-prochirality

Stereochemical descriptors for prostereoisomerism/prochirality. Homotopic and Diastereotopic ligands and faces.[1]

Topicity: Describes the different properties of constitutionally equivalent groups.

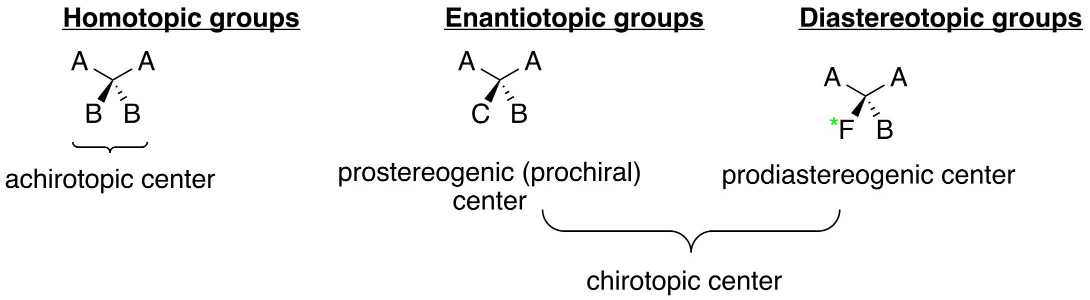

Topicity Descriptors: *Re*-*Si* are the descriptors for heterotopic faces as in abX=Y, indicating their two-dimensional chirality.
Enantiotopic groups: *Re*-*Si* are assigned by the chirotopic semispace defined by the remaining groups according to the local chirality. One of the constitutionally equivalent substituents is taken out of the plane, and the tripod is followed according to the sense of priority of the CIP rules.
Diastereotopic groups: The descriptor of the closest stereogenic center is added to the first Re/Si topicity descriptor.

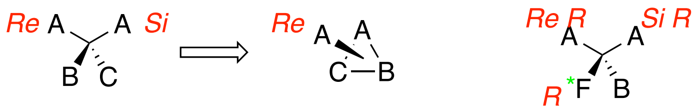

Stereochemical descriptors for the IMDA reaction: *Re/Si* are used to describe the relative topicity of the dienophile.

For the IMDA reaction[2–5] of the the *Z*- and *E*-geometric isomers of the *syn* and *anti* diastereoisomers, the sixteen possible bicyclic octahydronaphthalene diastereomers should have the depicted relative and absolute configurations of Scheme S1.

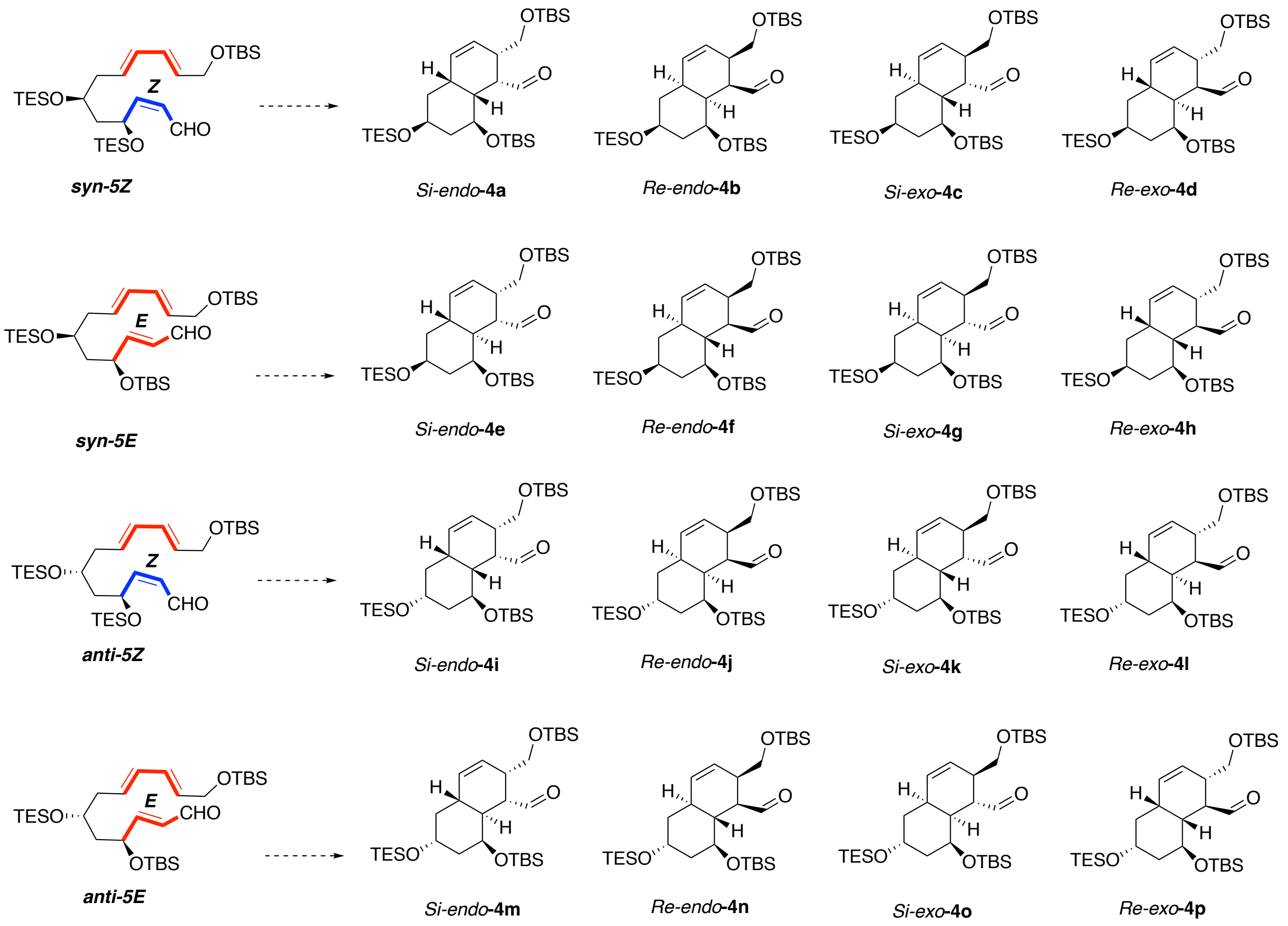

Scheme S1. Analysis of the stereochemical outcome on the IMDA reaction of the (2*Z*,8*E*,10*E*)- and (2*E*,8*E*,10*E*)-4,6,12-trihydroxy-2,8,10-decatrienal and relative and absolute configurations of the protected dihydroxyoctahydronaphthalenes.

2. TADA reactions

Precedents for the formation of macrodiolides following a similar reactivity pattern were reported by Nicolaou *et al*. on their synthesis of the antibiotic CJ-16,264.[6,7] The formation of 13-membered lactones from trienoic acids **S1*E*** and **S1*Z*** using various conditions (Scheme S2), including Yamaguchi,[1] and Keck[8] protocols, could not be achieved. This led to the alternative generation of 26-membered macrolides of *E* geometries upon treatment of the trienoic acids **S1*E*** under Shiina's conditions (MNBA, Et_3N, cat. DMAP),[9] and regardless of the geometry of the unsaturated carboxylic acid, since **S1*Z*** underwent a rapid isomerization perhaps resulting from Michael addition/bond rotation/elimination of DMAP[10] or Et_3N[11]. Interestingly, although the acyclic hydroxy-trienoic acids also failed to undergo the IMDA reaction, treatment of the *E*,*E*,*E*-macrodiolides under thermal reaction conditions (*m*-xylene, 220 °C, 12h) generated the product of the double transannular IMDA reaction,[12] namely the 10-membered ring macrodiolide **S4** resulting from the *Re*-*exo* cycloaddition, as demonstrated by X-Ray diffraction analysis, which also confirmed the relative configuration of the generated octahydronaphthalenes (Scheme S2).

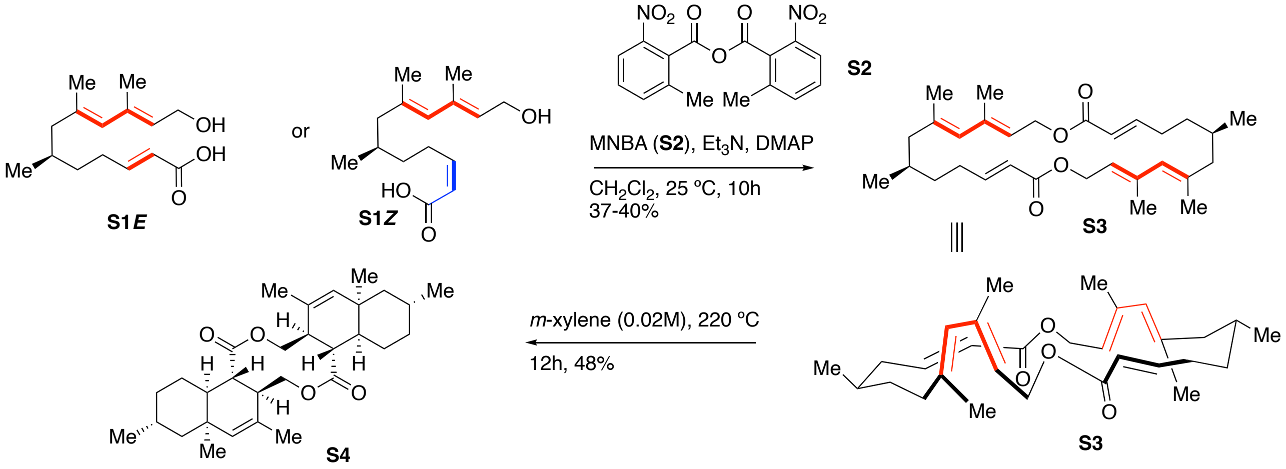

Scheme S2. Steps based on the TADA reaction for the synthesis of the antibiotic CJ-16,264.[6,7]

Further treatment of diolide **S4** with NaOMe in THF:wet MeOH at 65 °C led to γ-lactone epimerized at C1 **S6** as the major product, and hydroxy acid **S5** as minor product (3:2 ratio). Alternatively, the desired hydroxy acid was obtained as major product (2:1 ratio) upon treatment of diolide **S4** with LiOH in a solution of THF:MeOH:H_2O. Conformational analysis of the *cis*-octahydronaphthalene suggested the conformational preference that avoids two *syn*-pentane interaction of the carboxylic acid and the 1,3-diaxial interactions of the methyl substituents.[6,7]

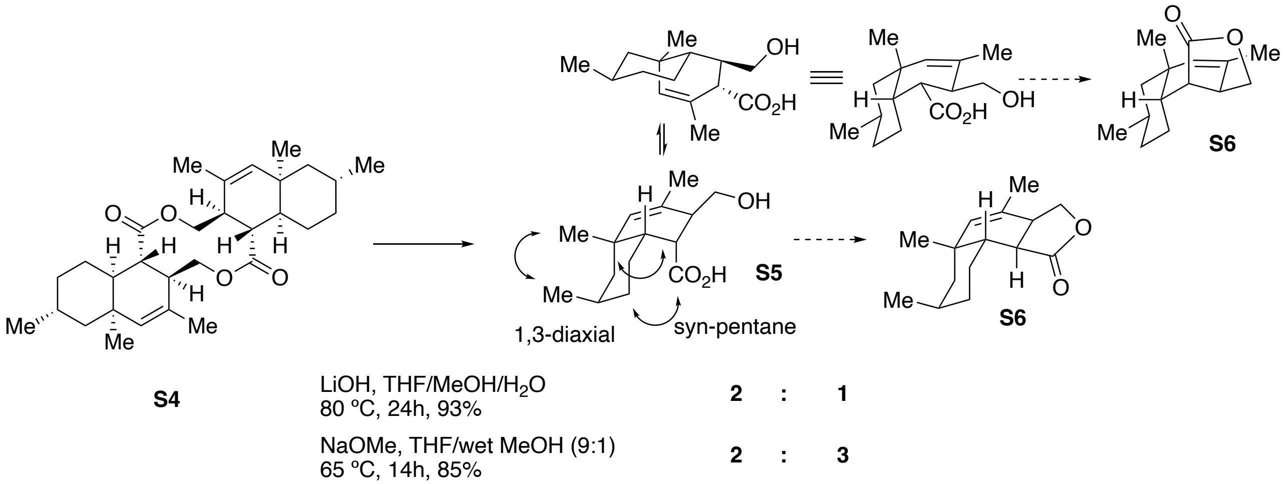

Scheme S3. Transformation of the TADA reaction product.[6,7]

3. Less-efficient alternative methods to obtain (*S*)-2-(*tert*-butyldimethylsilyloxy)-1-(4-methoxybenzyloxy)pent-4-ene 8

The protected homoallylic alcohol **8** was first prepared by protection of (Z)-but-2-ene-1,4-diol **S7** (Scheme S4), ozonolysis combined with PPh_3 to obtain the aldehyde **S9**. The Brown[13] allylation and the consecutive protection of the homoallylic alcohol generated **8** in 22% yield.

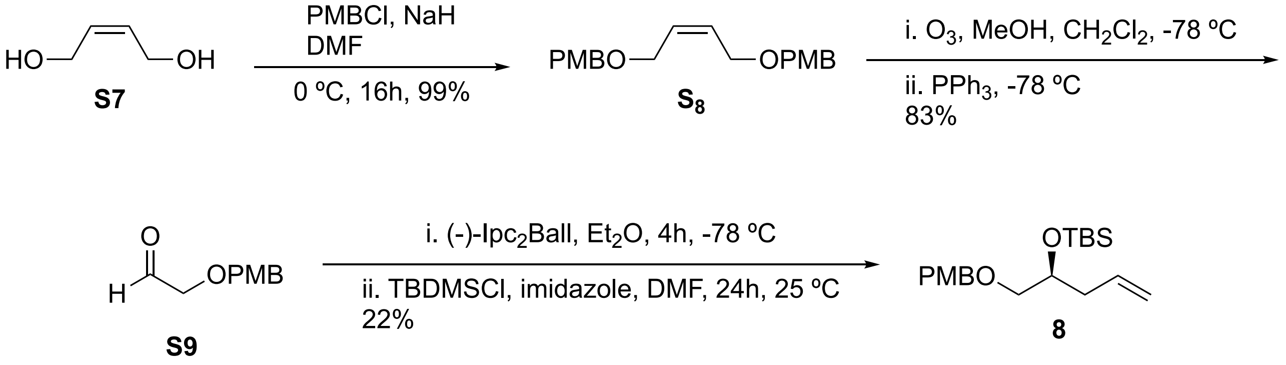

Scheme S4. Less-efficient synthesis of **8** using Brown[13] allylation.

The krische[14] allylation was first set up using aldehyde **S9** (41%, scheme S5). The combination of (*S*)-Cl,MeO-BIPHEP and *m*-nitrobenzoic acid using the protected ethylene glycol **10** generated homoallylic alcohol **11** in 71% yield. When (*S*)-BINAP was used instead of (*S*)-Cl,MeO-BIPHEP afforded the expected product in 55% yield. The best combination of (*S*)-BINAP and 4-Cl-3NO_2BzOH afforded the homoallylic alcohol **11** in 89% yield.

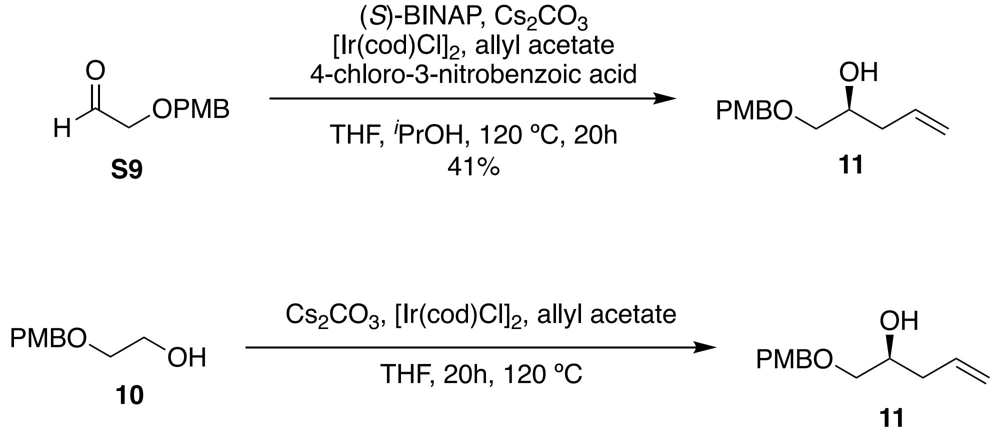

Ligand	Acid	Yield
(*S*)-BINAP	4-Cl-3-NO_2BzOH	89%
(*S*)-BINAP	*m*-nitrobenzoic acid	55%
(*S*)-Cl,MeO-BIPHEP	*m*-nitrobenzoic acid	71%

Scheme S5. Krische[14] allylation with aldehyde **S9** and protected ethylene glycol **10**.

4. Synthesis of Mosher's esters (*R*)-S10 and (*S*)-S10

The absolute configuration of the homoallylic alcohol 11 was confirmed by the formation of the diastereomeric esters with (*R*)- and (*S*)-MTPA in the presence of DCC and DMAP. Analysis of the differences in the chemical shifts of the PMB and olefin signals (due to the effect of the phenyl ring) allowed us to confirm the absolute configuration of homoallyl alcohol **11**.

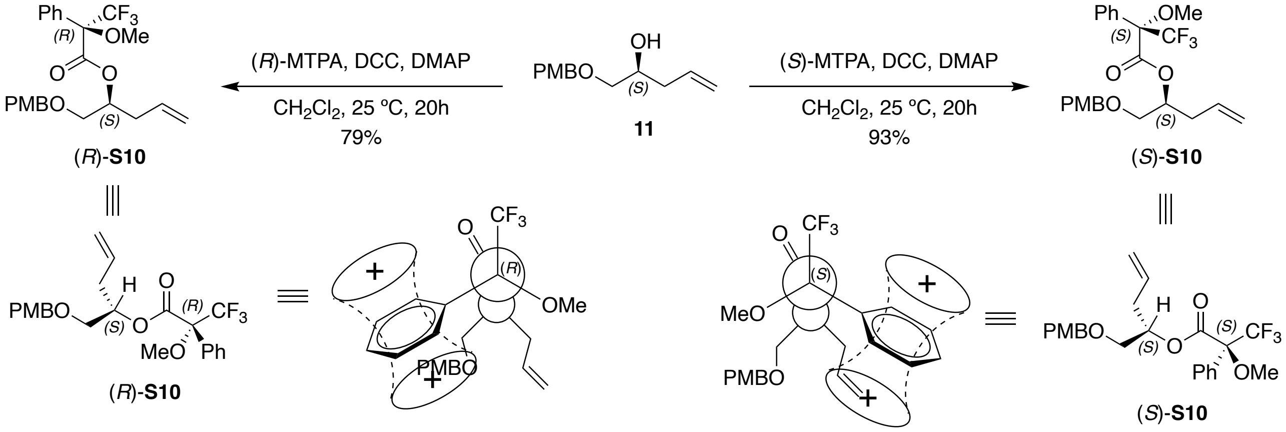

Scheme S6. Synthesis of Mosher's esters. (*R*)-**S10** and (*S*)-**S10**.

5. **Synthesis of acetonide S11**

The relative configuration of the second homoallylic alcohol generated was confirmed by formation of the acetonide **S11** after deprotection of the silyl ether **13** (Scheme S7).

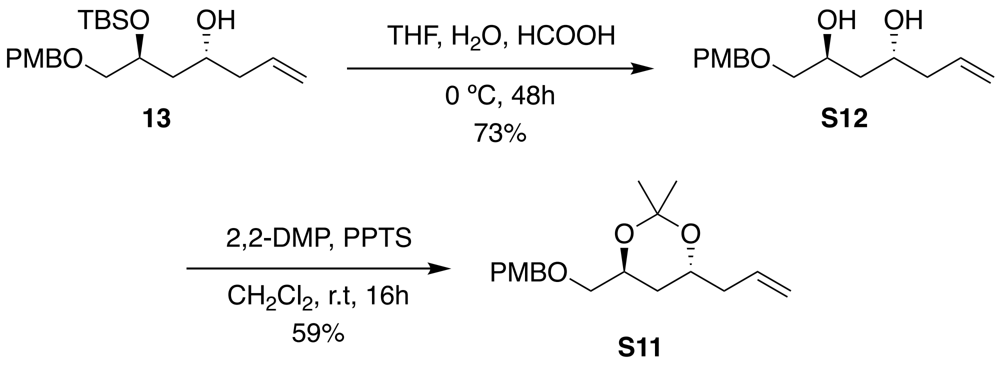

Scheme S7. Synthesis of acetonide **S11**.

When R_1 and R_2 are very bulky the most stable conformation is a twisted boat where the methyl groups occupy equivalent positions. [15]

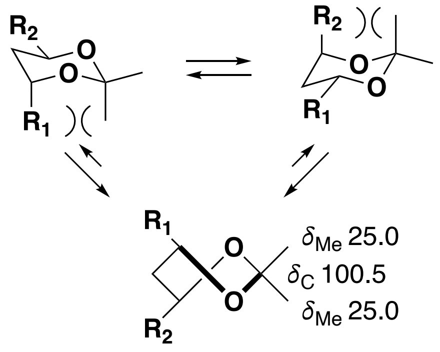

Figure S1. Analysis of the different conformations of the acetonide with *anti-* relative configuration.

6. Synthesis of alkenyliodide 19

This compound was synthesized in very high yield (84% combined yield) following the reported procedure (Scheme S8).[16]

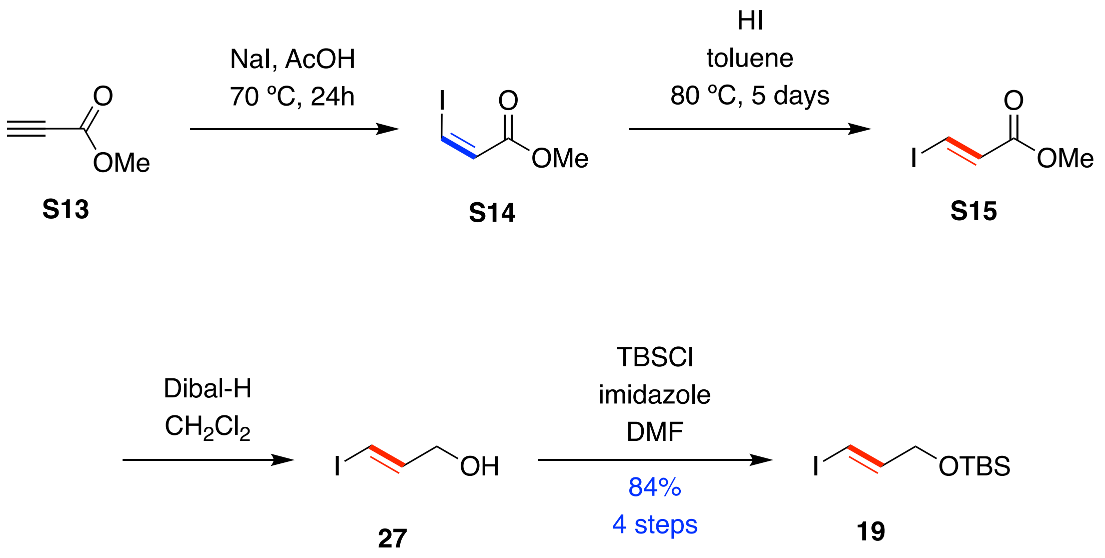

Scheme S8. Synthesis of iodide **19**.

7. Experimental procedures

(*Z*)-1,4-bis(4-Methoxybenzyloxy)but-2-ene S8

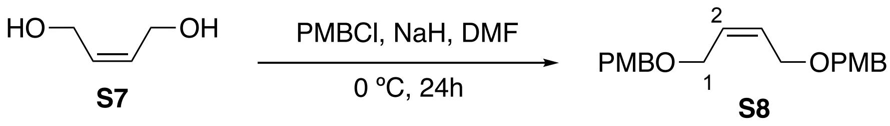

p-Methoxybenzyl chloride (8.8 mL, 62.43 mmol) was added to a cooled (0 °C) suspension of NaH (2.50 g, 60% in mineral oil, 62.43 mmol) in DMF (37 mL). After stirring for 30 min, (*Z*)-but-2-ene-1,4-diol **S7** (2.5 g, 28.38 mmol) was added, and the reaction mixture was stirred at room temperature for 16h. After cooling down to 0 °C, a saturated aqueous solution of NH_4Cl was added and the mixture was extracted with ethyl acetate (3x). The combined organic layers were washed with a saturated aqueous solution of $NaHCO_3$ (3x), brine (3x), dried over anhydrous Na_2SO_4, filtered and the solvent was evaporated. The residue was purified by flash column chromatography (silica gel, from 95:5 to 80:20 (v/v) *n*-hexane/EtOAc) to afford 9.31 g (99% yield) of a yellow oil, which was identified as (*Z*)-1,4-bis(4-methoxybenzyloxy)but-2-ene **S8**. The spectroscopic data matched those for the same product previously reported in the literature.[17]

^{1}H-NMR (400.16 MHz, $CDCl_3$): δ 7.28 – 7.26 (m, 4H, 4xArH), 6.91 – 6.88 (m, 4H, 4xArH), 5.82 – 5.77 (m, 2H, H_2 + H_3), 4.44 (s, 4H, 2xOCH_2Ar), 4.05 (d, *J* = 4.8 Hz, 4H, 2H_1 + 2H_4), 3.83 (s, 6H, 2xOCH_3) ppm.

^{13}C-NMR (100.63 MHz, $CDCl_3$): δ 159.3 (s, 2x), 130.3 (s, 2x), 129.6 (d, 4x), 129.4 (d, 4x), 113.8 (d, 2x), 71.9 (t, 2x), 65.5 (t, 2x), 55.3 (q, 2x) ppm.

IR (NaCl): υ 3001 (w, C-H), 2836 (w, C-H), 1612 (m, C=C), 1513 (s, C=C), 1248 (s, C-O) cm^{-1}.

2-(4-Methoxybenzyloxy)acetaldehyde S9

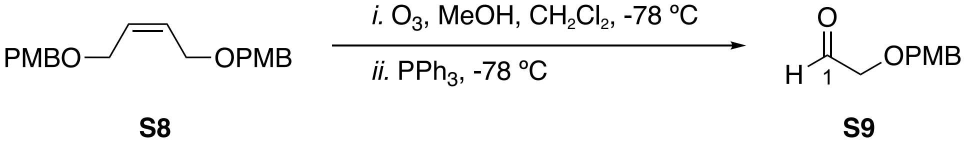

A cooled (-78 °C) solution of (*Z*)-1,4-bis(4-methoxybenzyloxy)but-2-ene **S8** (9.33 g, 28.41 mmol) in CH_2Cl_2 (137 mL) and MeOH (137 mL) was treated with O_3 for 30 min. When the solution turned blue, PPh_3 (1.18 g, 42.62 mmol) was added at the same temperature. The mixture was allowed to reach room temperature and the solvent was evaporated. The residue was purified by flash column chromatography (silica gel, from 80:20 to 60:40 (v/v) *n*-hexane/EtOAc) to afford 8.48 g (83% yield) of a yellow oil, which was identified as 2-(4-methoxybenzyloxy)acetaldehyde **S9**. The spectroscopic data matched those for the same product previously reported in the literature.[17]

^{1}H-NMR (400.16 MHz, $CDCl_3$): δ 9.73 (t, *J* = 0.9 Hz, 1H, H_1), 7.35 – 7.28 (m, 2H, 2xArH), 6.94 – 6.91 (m, 2H, 2xArH), 4.59 (s, 2H, OCH_2Ar), 4.09 (d, *J* = 1.0 Hz, 2H, $2H_2$), 3.84 (s, 3H, OCH_3) ppm.

^{13}C-NMR (100.63 MHz, $CDCl_3$): δ 200.7 (d), 159.7 (s), 129.8 (d, 2x), 128.9 (s), 114.0 (d, 2x), 75.0 (t), 73.3 (t), 55.3 (q) ppm.

IR (NaCl): υ 2866 (w, C-H), 2837 (w, C-H), 1736 (w, C=O), 1612 (m, C=C), 1514 (s, C=C), 1249 (s, C-O) cm^{-1}.

(*S*)-2-(*tert*-Butyldimethylsilyloxy)-1-(4-methoxybenzyloxy)pent-4-ene 8

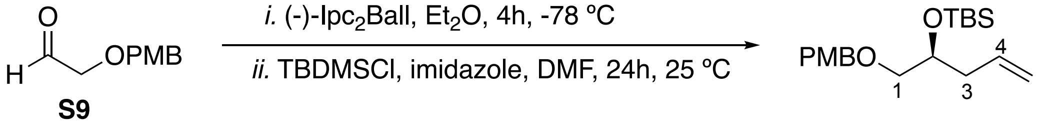

S9 **8**

To a cooled (-78 °C) solution of 2-(4-methoxybenzyloxy)acetaldehyde **S9** (0.50 g, 2.78 mmol) in Et_2O (6 mL) was added dropwise a solution of (-)-Ipc_2Ball (1.81 g, 5.56 mmol) in Et_2O (6 mL). The reaction mixture was stirred at this temperature for 3h and then diluted with H_2O_2 (7 mL, 30% w/v) and with a 3M aqueous solution of NaOH (14 mL). The mixture was stirred at room temperature for 2h and then extracted with ethyl acetate (3x). The combined organic layers were washed with a saturated aqueous solution of $NaHCO_3$ (3x), brine (3x), dried over anhydrous Na_2SO_4 and the solvent was evaporated.

Imidazole (0.05 g, 0.80 mmol) and TBSCl (0.12 g, 0.80 mmol) were added to a solution of the crude obtained above in DMF (4.5 mL). The reaction was stirred at room temperature for 24h. Water was added, and the mixture was extracted with Et_2O (3x). The combined organic layers were washed with water (3x), dried over anhydrous Na_2SO_4, filtered and the solvent was evaporated. The residue was purified by flash column chromatography (silica gel, from 95:5 to 80:20 (v/v) *n*-hexane/EtOAc) to afford 109 mg (22% yield) of a colourless oil, which was identified as (*S*)-2-(*tert*-butyldimethylsilyloxy)-1-(4-methoxybenzyloxy)pent-4-ene **8**. The spectroscopic data matched those for the same product previously reported in the literature.[18]

$[\alpha]_D$^{27} = +1.6 (*c* 0.49, $CHCl_3$).

^{1}H-NMR (400.16 MHz, $CDCl_3$): δ 7.28 – 7.23 (m, 2H, 2xArH), 6.90 – 6.84 (m, 2H, 2xArH), 5.82 (ddt, *J* = 17.2, 10.2, 7.2 Hz, 1H, H_4), 5.09 – 4.98 (m, 2H, $2H_5$), 4.45 (s, 2H, OCH_2Ar), 3.85 (app q, *J* = 6.3 Hz, H_2), 3.81 (s, 3H, OCH_3), 3.41 – 3.31 (m, 2H, H_{1A} + H_{1B}), 2.38 – 2.29 (m, 1H, H_{3A}), 2.27 – 2.15 (m, 1H, H_{3B}), 0.88 (s, 9H, $SiC(CH_3)_3$), 0.05 (s, 3H, Si-CH_3), 0.04 (s, 3H, Si-CH_3) ppm.

^{13}C-NMR (100.63 MHz, $CDCl_3$): δ 159.1 (s), 135.0 (d), 130.6 (s), 129.2 (d, 2x), 117.0 (t), 113.7 (d, 2x), 74.0 (t), 73.0 (t), 71.2 (d), 55.3 (q), 39.4 (t), 25.9 (q, 3x), 18.2 (s), -4.5 (q), -4.7 (q) ppm.

IR (NaCl): υ 2929 (w, C-H), 2856 (w, C-H), 1612 (w, C=C), 1514 (m, C=C), 1249 (s, C-O), 1106 (s, Si-O-C) cm^{-1}.

2-(4-Methoxybenzyloxy)ethan-1-ol 10

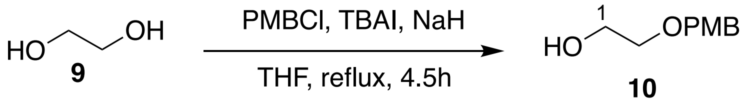

Ethylene glycol **9** (4.7 mL, 83.28 mmol) was added to a stirred suspension of NaH (1.11 g, 27.76 mmol) in THF (50 mL) and the mixture was stirred at room temperature for 30 min. Then, PMBCl (4 mL, 27.76 mmol) and TBAI (1.03 g, 2.77 mmol) were added, and the mixture was stirred under reflux for 4.5h. A saturated aqueous solution of NH_4Cl was added and the mixture was extracted with Et_2O (3x). The combined organic layers were washed with brine, dried over anhydrous Na_2SO_4, filtered and the solvent was evaporated. The residue was purified by flash column chromatography (silica gel, from 90:10 to 45:55 (v/v) *n*-hexane/EtOAc) to afford 4.84 g (96% yield) of a yellow oil, which was identified as 2-(4-methoxybenzyloxy)ethan-1-ol **10**.

(*S*)-1-(4-Methoxybenzyloxy)pent-4-en-2-ol 11

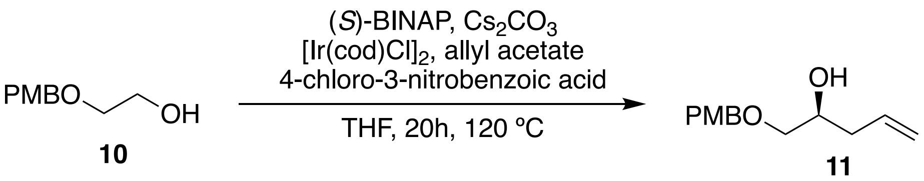

A sealing tube was charged with (*S*)-BINAP (25 mg, 0.04 mmol), $[Ir(cod)Cl]_2$ (13 mg, 0.02 mmol), 4-chloro-3-nitrobenzoic acid (16 mg, 0.08 mmol), Cs_2CO_3 (49 mg, 0.15 mmol) and a solution of 2-(4-methoxybenzyloxy)ethan-1-ol **10** (140 mg, 0.77 mmol) in THF (3.8 mL). Then, allyl acetate (0.8 mL, 7.7 mmol) was added, and the mixture was stirred at 120 °C for 20h. The solvent was evaporated and the residue was purified by flash column chromatography (silica gel, from 95:5 to 65:35 (v/v) *n*-hexane/EtOAc) to afford 152.2 mg (89% yield) of a yellow oil, which was identified as (*S*)-1-(4-methoxybenzyloxy)pent-4-en-2-ol **11**. The enantiomeric excess (*e.e.* 87%) was determined by chiral-HPLC (Chiralcel OD-H, *n*-hexane/^{i}PrOH/CH_2Cl_2 95.5:4:0.5 v/v, 0.7 mL/min, 275 nm), t_R = 10.7 min for the *S*-enantiomer and t_R = 11.9 min for the *R*-enantiomer; *e.e.* = 87%). The spectroscopic data matched those for the same product previously reported in the literature.[19]

$[\alpha]_D$^{27} = -2.0 (*c* 1.30, $CHCl_3$).

^{1}H-NMR (400.16 MHz, $CDCl_3$): δ 7.30 – 7.22 (m, 2H, 2xArH), 6.91 – 6.86 (m, 2H, 2xArH), 5.82 (ddt, *J* = 17.2, 10.2, 7.1 Hz, 1H, H_4), 5.15 – 5.06 (m, 2H, 2H_5), 4.48 (s, 2H, OCH_2Ar), 3.90 – 3.83 (m, 1H, H_2),

3.81 (s, 3H, OCH_3), 3.49 (dd, J = 9.5, 3.4 Hz, 1H, H_{1A}), 3.34 (dd, J = 9.5, 7.4 Hz, 1H, H_{1B}), 2.28 – 2.23 (m, 2H, $2H_3$) ppm.

^{13}C-NMR (100.63 MHz, $CDCl_3$): δ 159.1 (s), 134.1 (d), 129.9 (s), 129.2 (d, 2x), 117.4 (t), 113.7 (d, 2x), 73.4 (t), 72.8 (t), 69.5 (d), 55.1 (q), 37.7 (t) ppm.

IR (NaCl): υ 3500 - 3200 (br, O-H), 2931 (m, C-H), 2837 (m C-H), 1612 (s, C=C), 1514 (s, C=C), 1249 (s, C-O) cm^{-1}.

(*S*)-1-(4-Methoxybenzyloxy)pent-4-en-2-ol 11

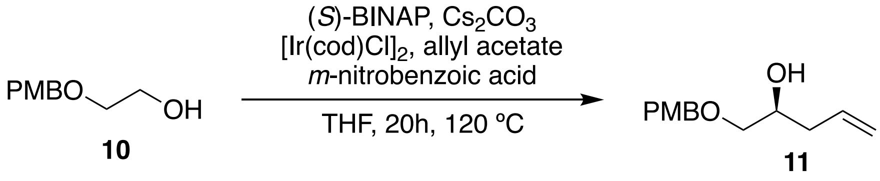

A sealing tube was charged with (*S*)-BINAP (11 mg, 0.02 mmol), $[Ir(cod)Cl]_2$ (6 mg, 0.01 mmol), *m*-nitrobenzoic acid (6 mg, 0.04 mmol), Cs_2CO_3 (23 mg, 0.07 mmol) and a solution of 2-(4-methoxybenzyloxy)ethan-1-ol **10** (64 mg, 0.35 mmol) in THF (1.8 mL). Then, allyl acetate (0.4 mL, 3.5 mmol) was added, and the mixture was stirred at 120 °C for 20h. The solvent was evaporated and the residue was purified by flash column chromatography (silica gel, from 95:5 to 60:40 (v/v) *n*-hexane/EtOAc) to afford 43 mg (55% yield) of a yellow oil, which was identified as (*S*)-1-(4-methoxybenzyloxy)pent-4-en-2-ol **11**.

(*S*)-1-(4-Methoxybenzyloxy)pent-4-en-2-ol 11

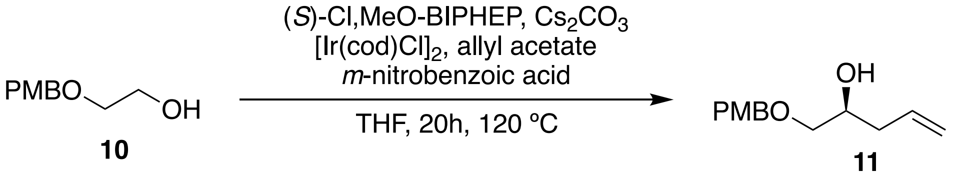

A sealing tube was charged with (*S*)-Cl,MeO-BIPHEP (14 mg, 0.02 mmol), $[Ir(cod)Cl]_2$ (7 mg, 0.01 mmol), *m*-nitrobenzoic acid (7 mg, 0.04 mmol), Cs_2CO_3 (28 mg, 0.09 mmol) and a solution of 2-(4-methoxybenzyloxy)ethan-1-ol **10** (79 mg, 0.4 mmol) in THF (2.2 mL). Then, allyl acetate (0.5 mL, 4.3 mmol) was added, and the mixture was stirred at 120 °C for 20h. The solvent was evaporated and the residue was purified by flash column chromatography (silica gel, from 95:5 to 60:40 (v/v) *n*-hexane/EtOAc) to afford 69 mg (71% yield) of a yellow oil, which was identified as (*S*)-1-(4-methoxybenzyloxy)pent-4-en-2-ol **11**.

rac-1-(4-Methoxybenzyloxy)pent-4-en-2-ol rac-11

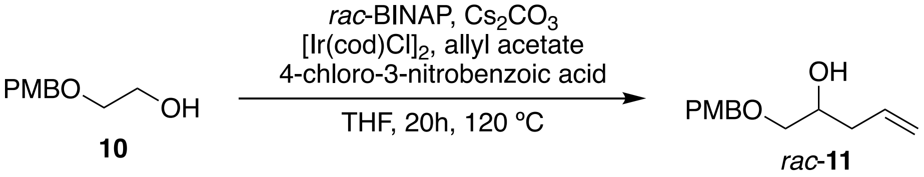

A sealing tube was charged with (*rac*)-BINAP (26 mg, 0.04 mmol), $[Ir(cod)Cl]_2$ (14 mg, 0.02 mmol), 4-chloro-3-nitrobenzoic acid (17 mg, 0.08 mmol), Cs_2CO_3 (54 mg, 0.17 mmol) and a solution of 2-(4-methoxybenzyloxy)ethan-1-ol **10** (150 mg, 0.82 mmol) in THF (4.1 mL). Then, allyl acetate (0.9 mL, 8.2 mmol) was added, and the mixture was stirred at 120 °C for 20h. The solvent was evaporated and the residue was purified by flash column chromatography (silica gel, from 95:5 to 60:40 (v/v) *n*-hexane/EtOAc) to afford 83 mg (45% yield) of a yellow oil, which was identified as *rac*-1-(4-methoxybenzyloxy)pent-4-en-2-ol *rac*-**11**.

(*S*)-1-(4-Methoxybenzyloxy)pent-4-en-2-ol 11

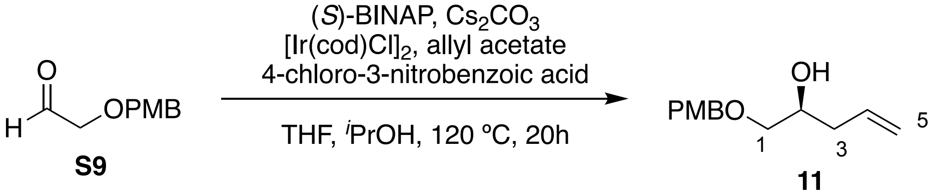

A sealing tube was charged with (*S*)-BINAP (17 mg, 0.03 mmol), $[Ir(cod)Cl]_2$ (9 mg, 0.01 mmol), 4-chloro-3-nitrobenzoic acid (11 mg, 0.06 mmol), Cs_2CO_3 (36 mg, 0.11 mmol) and a solution of 2-(4-methoxybenzyloxy)acetaldehyde **S9** (100 mg, 0.56 mmol) in THF (2.8 mL). Then, allyl acetate (0.6 mL, 5.55 mmol) and ^{i}PrOH (83 μL, 1.12 mmol) were added and the reaction mixture was stirred at 120 °C for 20h. The solvent was evaporated and the residue was purified by flash column chromatography (silica gel, from 95:5 to 80:20 (v/v) *n*-hexane/EtOAc) to afford 50 mg (41% yield) of a yellow oil, which was identified as (*S*)-1-(4-methoxybenzyloxy)pent-4-en-2-ol **11**.

(2*R*,2'*S*)-1-(4-Methoxybenzyloxy)pent-4-en-2-yl 3,3,3-Trifluoro-2-methoxy-2-phenylpropanoate (*R*)-S10

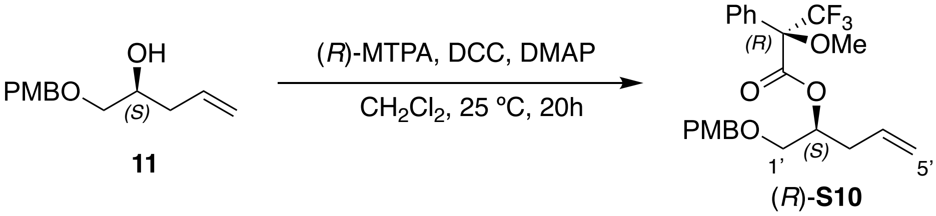

To a stirred solution of (*S*)-1-(4-methoxybenzyloxy)pent-4-en-2-ol **11** (32 mg, 0.14 mmol) in CH_2Cl_2 (2.8 mL), (*R*)-MTPA (40 mg, 0.17 mmol), DMAP (1 mg, 0.01 mmol) and DCC (41 mg, 0.20 mmol)

were added and the mixture was stirred overnight at room temperature. Then, the reaction mixture was filtered off and the solvent was evaporated. The residue was purified by flash column chromatography (silica gel, from 100:0 to 80:20 (v/v) *n*-hexane/EtOAc) to afford 49 mg (79% yield) of a colorless oil, which was identified as (2*R*,2'*S*)-1-(4-methoxybenzyloxy)pent-4-en-2-yl 3,3,3-trifluoro-2-methoxy-2-phenylpropanoate (*R*)-**S10**.

[α]$_D$^{22} = +31.7 (*c* 0.28, $CHCl_3$).

^{1}H-NMR (400.16 MHz, $CDCl_3$): δ 7.55 (d, *J* = 7.6 Hz, 2H, 2xArH), 7.40 – 7.31 (m, 3H, 3xArH), 7.15 (d, *J* = 8.7 Hz, 2H, 2xArH), 6.84 (d, *J* = 8.7 Hz, 2H, 2xArH), 5.76 (ddt, *J* = 17.1, 10.2, 7.0 Hz, 1H, $H_{4'}$), 5.39 (app. dq, *J* = 7.3, 5.4 Hz, 1H, $H_{2'}$), 5.16 – 5.09 (m, 2H, $H_{5'A}$ + $H_{5'B}$), 4.39 (d, *J* = 11.5 Hz, 1H, OCH_2Ar), 4.34 (d, *J* = 11.5 Hz, 1H, OCH_2Ar), 3.80 (s, 3H, OCH_3), 3.60 – 3.50 (m, 3H, OCH_3), 3.50 (d, *J* = 5.5 Hz, 2H, 2$H_{1'}$), 2.47 (dt, *J* = 5.4, 1.3 Hz, 1H, $H_{3'A}$), 2.44 (dt, *J* = 7.4, 1.1 Hz, 1H, $H_{3'B}$) ppm.

^{13}C-NMR (100.63 MHz, $CDCl_3$): δ 166.3 (s), 159.3 (s), 132.8 (d), 132.4 (s), 129.9 (s), 129.6 (d, 2x), 129.3 (d, 2x), 128.4 (d, 2x), 127.7 (d), 123.5 (s, $^1J_{C-F}$ = 289 Hz), 118.9 (t), 113.8 (d, 2x), 84.8 (s, $^2J_{C-F}$ = 27.5 Hz), 74.7 (d), 72.9 (t), 70.3 (t), 55.7 (q, $^4J_{C-F}$ = 1.6 Hz), 55.4 (q), 35.4 (t) ppm.

IR (NaCl): υ 2933 (w, C-H), 2853 (w, C-H), 1747 (s, C=O), 1613 (w, C=C), 1514 (m, C=C), 1250 (s, C-O), 1171 (s, C-F) cm^{-1}.

HRMS (ESI$^+$): calcd. for $C_{23}H_{25}F_3O_5Na$ ($[M+Na]^+$) 461.1546; found, 461.1561.

(2*S*,2'*S*)-1-(4-Methoxybenzyloxy)pent-4-en-2-yl 3,3,3-Trifluoro-2-methoxy-2-phenylpropanoate (*S*)-S10

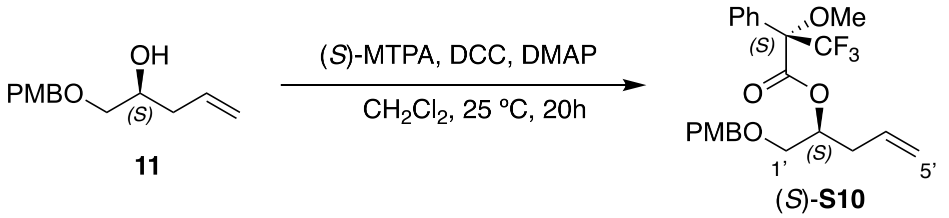

Following the procedure described above, the reaction of (*S*)-MTPA (38 mg, 0.17 mmol), DMAP (1 mg, 0.01 mmol), DCC (39 mg, 0.19 mmol) and (*S*)-1-(4-methoxybenzyloxy)pent-4-en-2-ol **11** (30 mg, 0.13 mmol) in CH_2Cl_2 (2.7 mL) afforded, after purification by flash column chromatography (silica gel, from 100:0 to 80:20 (v/v) *n*-hexane/EtOAc), 55 mg (93% yield) of a colorless oil, which was identified as (2*S*,2'*S*)-1-(4-methoxybenzyloxy)pent-4-en-2-yl 3,3,3-trifluoro-2-methoxy-2-phenylpropanoate (*S*)-**S10**.

[α]$_D$^{23} = -16.6 (*c* 0.34, $CHCl_3$).

^{1}H-NMR (400.16 MHz, $CDCl_3$): δ 7.57 (d, *J* = 7.6 Hz, 2H, 2xArH), 7.39 – 7.29 (m, 3H, 3xArH), 7.22 (d, *J* = 8.8 Hz, 2H, 2xArH), 6.87 (d, *J* = 8.8 Hz, 2H, 2xArH), 5.65 (ddt, *J* = 18.4, 9.5, 7.1 Hz, 1H, H_4), 5.39 (dt, *J* = 11.6, 5.7 Hz, 1H, $H_{2'}$), 5.06 – 5.00 (m, 2H, $H_{5'A}$ + $H_{5'B}$), 4.50 (d, *J* = 11.4 Hz, 1H, OCH_2Ar), 4.44 (d, *J* = 11.5 Hz, 1H, OCH_2Ar), 3.81 (s, 3H, OCH_3), 3.60 – 3.50 (m, 2H, $2H_{1'}$), 3.60 – 3.50 (m, 3H, OCH_3), 2.40 – 2.30 (m, 2H, $2H_{3'}$) ppm.

^{13}C-NMR (100.63 MHz, $CDCl_3$): δ 166.3 (s), 159.4 (s), 132.6 (s), 132.4 (d), 129.9 (s), 129.6 (d, 2x), 129.4 (d, 2x), 128.4 (d, 2x), 127.6 (d), 123.5 (s, $^{1}J_{C-F}$ = 289 Hz), 118.9 (t), 113.9 (d, 2x), 84.7 (s, $^{2}J_{C-F}$ = 27 Hz), 74.5 (d), 72.9 (t), 70.5 (t), 55.6 (q, $^{4}J_{C-F}$ = 1.6 Hz), 55.4 (q), 35.3 (t) ppm.

IR (NaCl): υ 2952 (w, C-H), 2853 (w, C-H), 1748 (s, C=O), 1612 (w, C=C), 1514 (m, C=C), 1249 (s, C-O), 1171 (s, C-F) cm^{-1}.

HRMS (ESI^+): calcd. for $C_{23}H_{25}F_3O_5Na$ ($[M+Na]^+$) 461.1546; found, 461.1548.

(*S*)-2-(*tert*-Butyldimethylsilyloxy)-1-(4-methoxybenzyloxy)pent-4-ene 8

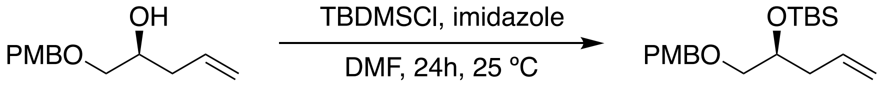

11 **8**

Imidazole (0.25 g, 3.65 mmol) and TBSCl (0.55 g, 3.65 mmol) were added to a solution of (*S*)-1-(4-methoxybenzyloxy)pent-4-en-2-ol **11** (0.68 g, 3.04 mmol) in DMF (21 mL) and the reaction mixture was stirred at room temperature for 24h. Water was added, and the mixture was extracted with Et_2O (3x). The combined organic layers were washed with water (3x), dried over anhydrous Na_2SO_4, filtered and the solvent was evaporated. The residue was purified by flash column chromatography (silica gel, from 100:0 to 90:10 (v/v) *n*-hexane/EtOAc) to afford 0.88 g (86% yield) of a yellow oil, which was identified as (*S*)-2-(*tert*-butyldimethylsilyloxy)-1-(4-methoxybenzyloxy)pent-4-ene **8**.

(*S*)-3-(*tert*-Butyldimethylsilyloxy)-4-(4-methoxybenzyloxy)butanal 12

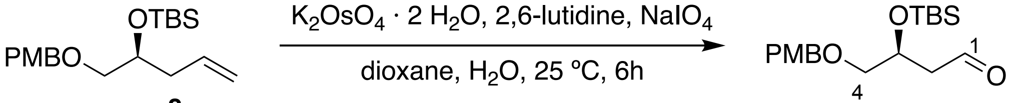

8 **12**

To a stirred solution of (*S*)-2-(*tert*-butyldimethylsilyloxy)-1-(4-methoxybenzyloxy)pent-4-ene **8** (0.37 g, 1.11 mmol) in dioxane-water (10 mL, 3:1 v/v), at 25 °C, were sequentially added 2,6-lutidine (195 μL, 1.67 mmol), $K_2OsO_4 \cdot 2H_2O$ (10 mg, 0.03 mmol) and $NaIO_4$ (0.71 g, 3.34 mmol). After stirring for 6h, water was added, and the mixture was extracted with CH_2Cl_2 (3x). The combined organic layers were washed with brine (3x), dried over anhydrous Na_2SO_4, filtered and

the solvent was evaporated. The residue was purified by flash column chromatography (silica gel, from 95:5 to 80:20 (v/v) *n*-hexane/EtOAc) to afford 0.35 g (93% yield) of a brown oil, which was identified as (*S*)-3-(*tert*-butyldimethylsilyloxy)-4-(4-methoxybenzyloxy)butanal **12**. The spectroscopic data matched those for the same product previously reported in the literature.[20]

[α]D^{26} = -13.8 (*c* 0.72, $CHCl_3$).

^{1}H-NMR (400.16 MHz, $CDCl_3$): δ 9.79 (dd, *J* = 2.7, 2.1 Hz, 1H, H_1), 7.25 - 7.21 (m, 2H, 2xArH), 6.90 - 6.85 (m, 2H, 2xArH), 4.45 (s, 2H, OCH_2Ar), 4.34 (app. tt, *J* = 6.5, 5.1 Hz, 1H, H_3), 3.81 (s, 3H, OCH_3), 3.48 (dd, *J* = 9.5, 5.1 Hz, 1H, H_{4A}), 3.36 (dd, *J* = 9.6, 6.3 Hz, 1H, H_{4B}), 2.64 (ddd, *J* = 15.9, 5.1, 2.1 Hz, 1H, H_{2A}), 2.56 (ddd, *J* = 15.9, 6.6, 2.7 Hz, 1H, H_{2B}), 0.86 (s, 9H, $SiC(CH_3)_3$), 0.05 (s, 3H, Si-CH_3), 0.06 (s, 3H, Si-CH_3) ppm.

^{13}C-NMR (100.63 MHz, $CDCl_3$): δ 201.5 (d), 159.3 (s), 130.0 (s), 129.3 (d, 2x), 113.8 (d, 2x), 73.7 (t), 73.1 (t), 67.4 (d), 55.3 (q), 49.0 (t), 25.7 (q, 3x), 18.0 (s), -4.5 (q), -5.0 (q) ppm.

IR (NaCl): υ 2954 (m, C-H), 2930 (m, C-H), 2857 (m, C-H), 1727 (s, C=O), 1613 (w, C=C), 1514 (s, C=C), 1250 (s, C-O), 1101 (s, Si-O-C) cm^{-1}.

(2*S*,4*R*)-2-(*tert*-Butyldimethylsilyloxy)-1-(4-methoxybenzyloxy)hept-6-en-4-ol 13

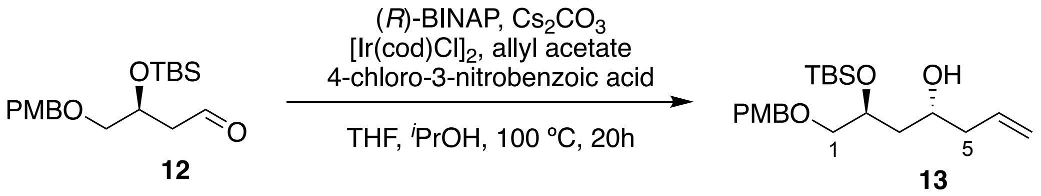

A sealing tube was charged with (*R*)-BINAP (30 mg, 0.05 mmol), $[Ir(cod)Cl]_2$ (16 mg, 0.02 mmol), 4-chloro-3-nitrobenzoic acid (20 mg, 0.10 mmol), Cs_2CO_3 (64 mg, 0.20 mmol) and a solution of (*S*)-3-(*tert*-butyldimethylsilyloxy)-4-(4-methoxybenzyloxy)butanal **12** (0.33 g, 0.98 mmol) in THF (4.9 mL). Then, allyl acetate (1.1 mL, 9.75 mmol) and ^{i}PrOH (300 μL, 3.90 mmol) were added and the reaction mixture was stirred at 100 °C for 20h. The solvent was evaporated and the residue was purified by flash column chromatography (silica gel, from 95:5 to 70:30 (v/v) *n*-hexane/EtOAc) to afford 0.26 g (70% yield) of a yellow oil, which was identified as (2*S*,4*R*)-2-(*tert*-butyldimethylsilyloxy)-1-(4-methoxybenzyloxy)hept-6-en-4-ol **13**.

[α]D^{26} = -11.9 (*c* 1.12, $CHCl_3$).

^{1}H-NMR (400.16 MHz, $CDCl_3$): δ 7.25 – 7.21 (m, 2H, 2xArH), 6.90 – 6.85 (m, 2H, 2xArH), 5.82 (ddt, *J* = 17.3, 10.3, 7.1 Hz, 1H, H_6), 5.13 – 5.06 (m, 2H, 2H_7), 4.57 – 4.40 (m, 2H, OCH_2Ar), 4.15 – 4.04 (m, 1H, H_4), 3.94 – 3.85 (m, 1H, H_2), 3.81 (s, 3H, O-CH_3), 3.49 – 3.40 (m, 2H, 2H_1), 3.20 (d, *J* = 2.3 Hz, 1H,

O-H), 2.28 – 2.14 (m, 2H, $2H_5$), 1.73 (ddd, J = 14.4, 5.2, 2.2 Hz, 1H, H_{3A}), 1.63 (ddd, J = 14.4, 10.0, 4.3 Hz, 1H, H_{3B}), 0.88 (s, 9H, $SiC(CH_3)_3$), 0.08 (s, 3H, $Si\text{-}CH_3$), 0.06 (s, 3H, $Si\text{-}CH_3$) ppm.

^{13}C-NMR (100.63 MHz, $CDCl_3$): δ 159.3 (s), 135.0 (d), 130.1 (s), 129.3 (d, 2x), 117.4 (t), 113.8 (d, 2x), 73.3 (t), 73.0 (t), 70.3 (d), 67.8 (d), 55.3 (q), 42.4 (t), 40.2 (t), 25.8 (q, 3x), 18.0 (s), -4.6 (q), -5.1 (q) ppm.

IR (NaCl): υ 3600 – 3300 (br, O-H), 2953 (m, C-H), 2930 (m, C-H), 2856 (m, C-H), 1613 (m, C=C), 1514 (s, C=C), 1249 (s, C-O), 1083 (s, Si-O-C) cm^{-1}.

HRMS (ESI^+): calcd. for $C_{21}H_{37}O_4Si$ ($[M+H]^+$), 381.2456; found, 381.2452.

(2*S*,4*R*)-1-(4-Methoxybenzyloxy)hept-6-en-2,4-diol S12

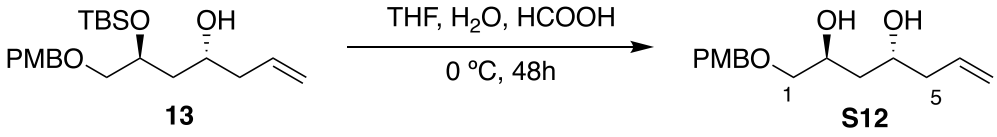

A solution of (2*S*,4*R*)-2-(*tert*-butyldimethylsilyloxy)-1-(4-methoxybenzyloxy)hept-6-en-4-ol 1**3** (100 mg, 0.26 mmol) in THF/H_2O/HCOOH (19 mL, 6:1.5:1 v/v) was stirred at 0 °C for 48h. A saturated aqueous solution of $NaHCO_3$ was then added and the mixture was extracted with EtOAc (3x). The combined organic layers were dried over anhydrous Na_2SO_4, filtered and the solvent was evaporated. The residue was purified by flash column chromatography (silica gel, from 90:10 to 20:70 (v/v) *n*-hexane/EtOAc) to afford 51 mg (73% yield) of a yellow oil, which was identified as (2*S*,4*R*)-1-(4-methoxybenzyloxy)hept-6-en-2,4-diol **S12**.

$[\alpha]_D^{23}$ = +0.7 (*c* 0.60, $CHCl_3$).

^{1}H-NMR (400.16 MHz, $CDCl_3$): δ 7.24 (d, J = 8.7 Hz, 2H, 2xArH), 6.87 (d, J = 8.7 Hz, 2H, 2xArH), 5.85 – 5.75 (m, 1H, H_6), 5.13 – 5.07 (m, 2H, $2H_7$), 4.47 (s, 2H, OCH_2Ar), 4.11 (ddt, J = 8.5, 7.3, 3.6 Hz, 1H, H_2), 3.97 – 3.90 (m, 1H, H_4), 3.79 (s, 3H, OCH_3), 3.46 (dd, J = 9.5, 3.7 Hz, 1H, H_{1A}), 3.36 (dd, J = 9.5, 7.7 Hz, 1H, H_{1B}), 2.59 (br s, 2H, 2xO-H), 2.26 – 2.21 (m, 2H, $2H_5$), 1.63 (ddd, J = 14.4, 8.5, 2.9 Hz, 1H, H_{3A}), 1.53 (ddd, J = 14.4, 8.8, 3.4 Hz, 1H, H_{3B}) ppm.

^{13}C-NMR (100.63 MHz, $CDCl_3$): δ 159.4 (s), 134.8 (d), 130.0 (s), 129.6 (d, 2x), 118.2 (t), 114.0 (d, 2x), 74.2 (t), 73.1 (t), 68.0 (d), 67.9 (d), 55.4 (q), 42.2 (t), 38.7 (t) ppm.

IR (NaCl): υ 3500 - 3200 (br, O-H), 2935 (m, C-H), 2910 (m, C-H), 2859 (m, C-H), 1612 (m, C=C), 1513 (s, C=C), 1249 (s, C-O) cm^{-1}.

HRMS (ESI^+): calcd. For $C_{15}H_{23}O_4$ ($[M+H]^+$) 267.1591; found, 267.1587.

(2*S*,4*R*)-Dioxolane S11

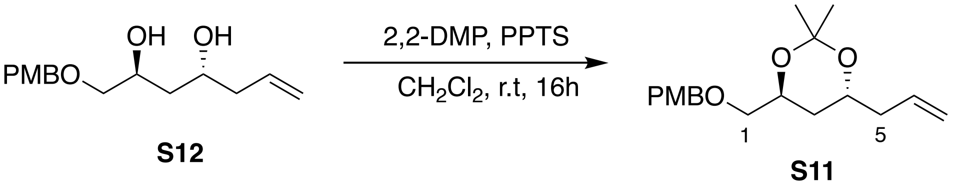

2,2-Dimethoxypropane (15 μL, 0.12 mmol) and pyridinium *p*-toluenesulfonate (1.4 mg, 0.006 mmol) were added to a solution of (2*S*,4*R*)-1-(4-methoxybenzyloxyhept-6-en-2,4-diol **S12** (12 mg, 0.05 mmol) in CH_2Cl_2 (0.5 mL) and the reaction mixture was stirred at room temperature overnight. A saturated aqueous solution of $NaHCO_3$ was added and the mixture was extracted with EtOAc (3x). The combined organic layers were washed with brine, dried over anhydrous Na_2SO_4, filtered and the solvent was evaporated. The residue was purified by flash column chromatography (silica gel, from 100:0 to 80:20 (v/v) *n*-hexane/EtOAc) to afford 8 mg (59% yield) of a yellow oil, which was identified as (2*S*,4*R*)-dioxolane **S11**.

$[\alpha]_D$^{24} = -6.9 (*c* 0.92, $CHCl_3$).

^{1}H-NMR (400.16 MHz, $CDCl_3$): δ 7.26 (d, *J* = 8.7 Hz, 2H, 2xArH), 6.87 (d, *J* = 8.7 Hz, 2H, 2xArH), 5.84 – 5.73 (m, 1H, H_6), 5.11 – 5.02 (m, 2H, $2H_7$), 4.55 (d, *J* = 11.9 Hz, 1H, OCH_2Ar), 4.47 (d, *J* = 11.9 Hz, 1H, OCH_2Ar), 4.06 – 3.99 (m, 1H, H_2), 3.89 – 3.82 (m, 1H, H_4), 3.80 (s, 3H, OCH_3), 3.46 (dd, *J* = 10.4, 6.5 Hz, 1H, H_{1A}), 3.39 (dd, *J* = 10.4, 4.1 Hz, 1H, H_{1B}), 2.34 – 2.26 (m, 1H, H_{5A}), 2.22 – 2.14 (m, 1H, H_{5B}), 1.67 – 1.61 (m, 1H, H_{3A}), 1.57 – 1.51 (m, 1H, H_{3B}), 1.39 (s, 3H, CH_3), 1.37 (s, 3H, CH_3) ppm.

^{13}C-NMR (100.63 MHz, $CDCl_3$): δ 159.3 (s), 134.5 (d), 129.6 (s), 129.5 (d, 2x), 117.1 (t), 113.9 (d, 2x), 100.5 (s), 73.1 (t), 72.4 (t), 66.4 (d), 66.3 (d), 55.4 (q), 40.2 (t), 34.3 (t), 25.0 (q, 2x) ppm.

IR (NaCl): υ 2924 (s, C-H), 2854 (s, C-H), 1614 (w, C=C), 1513 (w, C=C), 1248 (m, C-O) cm^{-1}.

HRMS (ESI^+): calcd. for $C_{18}H_{27}O_4$ ($[M+H]^+$) 307.1904; found, 307.1889.

(2*S*,4*R*)-2-(*tert*-Butyldimethylsilyloxy)-1-(4-methoxybenzyloxy)-4-(triethylsilyloxy)hept-6-ene 14

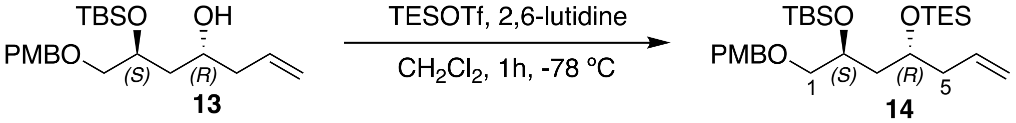

2,6-lutidine (0.15 mL, 1.37 mmol) and TESOTf (0.18 mL, 0.82 mmol) were added to a cooled (-78 °C) stirred solution of (4*R*,6*S*)-6-(*tert*-butyldimethylsilyloxy)-7-(4-methoxybenzyloxy)hept-1-en-4-ol **13** (0.21 g, 0.55 mmol) in CH_2Cl_2 (5.5 mL) and the resulting mixture was stirred at the same temperature for 1h. Then, water was added, and the mixture was extracted with CH_2Cl_2 (3x). The combined organic layers were dried over anhydrous Na_2SO_4, and the solvent was evaporated. The

residue was purified by flash column chromatography (silica gel, from 100:0 to 90:10 (v/v) *n*-hexane/EtOAc) to afford 0.23 g (85% yield) of a yellow oil, which was identified as (2*S*,4*R*)-2-(*tert*-butyldimethylsilyloxy)-1-(4-methoxybenzyloxy)-4-(triethylsilyloxy)hept-6-ene **14**.

[α]$_D$ ^{26} = -14.7 (*c* 1.18, $CHCl_3$).

^{1}H-NMR (400.16 MHz, $CDCl_3$, 323 K): δ 7.28 – 7.22 (m, 2H, 2xArH), 6.90 – 6.84 (m, 2H, 2xArH), 5.88 – 5.74 (m, 1H, H_6), 5.08 – 4.99 (m, 2H, $2H_7$), 4.44 (s, 2H, OCH_2Ar), 4.00 – 3.86 (m, 2H, H_2 + H_4), 3.81 (s, 3H, OCH_3), 3.38 – 3.35 (m, 2H, $2H_1$), 2.28 – 2.22 (m, 2H, H_{5A} + H_{5B}), 1.66 – 1.61 (m, 2H, H_{3A} + H_{3B}), 0.96 (t, *J* = 7.9 Hz, 9H, $3xSiCH_2C\underline{H_3}$), 0.89 (s, 9H, $SiC(CH_3)_3$), 0.60 (q, *J* = 7.9 Hz, 6H, 3xSi-$C\underline{H_2}CH_3$), 0.06 (s, 6H, 2xSi-CH_3) ppm.

^{13}C-NMR (100.63 MHz, $CDCl_3$, 323 K): δ 159.2 (s), 135.0 (d), 130.7 (s), 129.1 (d, 2x), 116.7 (t), 113.7 (d, 2x), 75.2 (t), 72.8 (t), 69.7 (d), 69.6 (d), 55.2 (q), 43.0 (t), 42.7 (t), 25.9 (q, 3x), 18.1 (s), 6.8 (q, 3x), 5.4 (t, 3x), -4.0 (q), -4.6 (q) ppm.

IR (NaCl): υ 2953 (s, C-H), 2934 (s, C-H), 2876 (s, C-H), 2856 (s, C-H), 1615 (w, C=C), 1514 (s, C=C), 1249 (s, C-O), 1092 (s, Si-O-C) cm^{-1}.

HRMS (ESI^+): calcd. for $C_{27}H_{51}O_4Si_2$ ($[M+H]^+$), 495.3320; found, 495.3302.

(3*S*,5*S*)-5-(*tert*-Butyldimethylsilyloxy)-6-(4-methoxybenzyloxy)-3-(triethylsilyloxy)hexanal 15

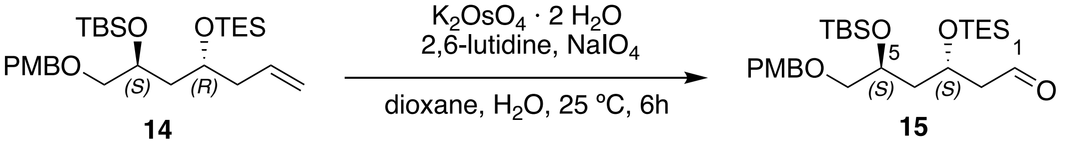

To a stirred solution of (2*S*,4*R*)-2-(*tert*-butyldimethylsilyloxy)-1-(4-methoxybenzyloxy)-4-(triethylsilyloxy)hept-6-ene **14** (6.60 g, 13.34 mmol) in dioxane-water (120 mL, 3:1 v/v) at 25 °C, 2,6-lutidine (2.3 mL, 20.01 mmol), $K_2OsO_4{\cdot}2H_2O$ (0.12 g, 0.32 mmol) and $NaIO_4$ (8.56 g, 40.01 mmol) were sequentially added. After 6h, water was added, and the mixture was extracted with CH_2Cl_2 (3x). The combined organic layers were washed with brine (3x), dried over anhydrous Na_2SO_4, filtered and the solvent was evaporated. The residue was purified by flash column chromatography (silica gel, from 95:5 to 85:15 (v/v) *n*-hexane/EtOAc) to afford 5.67 g (86% yield) of a dark oil, which was identified as (3*S*,5*S*)-5-(*tert*-butyldimethylsilyloxy)-6-(4-methoxybenzyloxy)-3-(triethylsilyloxy)hexanal **15**.

[α]$_D$^{26} = -7.0 (*c* 0.53, $CHCl_3$).

^{1}H-NMR (400.16 MHz, $CDCl_3$): δ 9.77 (dd, *J* = 2.9, 2.1 Hz, H_1), 7.24 (d, *J* = 8.6 Hz, 2H, 2xArH), 6.87 (d, *J* = 8.7 Hz, 2H, 2xArH), 4.43 (app. d, *J* = 2.9 Hz, 2H, OCH_2Ar), 4.37 – 4.31 (m, 1H, H_3), 3.93 -3.85 (m, 1H, H_5), 3.81 (s, 3H, OCH_3), 3.38 (dd, *J* = 9.7, 5.4 Hz, 1H, H_{6A}), 3.33 (dd, *J* = 9.7, 5.3 Hz, 1H, H_{6B}), 2.61

(ddd, J = 15.8, 5.0, 2.1 Hz, 1H, H_{2A}), 2.52 (ddd, J = 15.8, 6.4, 2.9 Hz, 1H, H_{2B}), 1.89 – 1.82 (m, 1H, H_{4A}), 1.71 – 1.64 (m, 1H, H_{4B}), 0.93 (t, J = 7.9 Hz, 9H, 3xSiCH$_2$C<u>H$_3$</u>), 0.87 (s, 9H, SiC(CH$_3$)$_3$), 0.59 (q, J = 8.2 Hz, 6H, 3xSi-C<u>H$_2$</u>CH$_3$), 0.06 (s, 3H, Si-CH$_3$), 0.05 (s, 3H, Si-CH$_3$) ppm.

^{13}C-NMR (100.63 MHz, $CDCl_3$): δ 202.3 (d), 159.3 (s), 130.4 (s), 129.4 (d, 2x), 113.8 (d, 2x), 74.8 (t), 73.1 (t), 69.4 (d), 66.1 (d), 55.4 (q), 51.9 (t), 43.9 (t), 26.0 (q, 3x), 18.3 (s), 7.0 (q, 3x), 5.2 (t, 3x), -4.0 (q), -4.5 (q) ppm.

IR (NaCl): υ 2954 (s, C-H), 2878 (m, C-H), 2857 (m, C-H), 1727 (m, C=O), 1613 (m, C=C), 1514 (m, C=C), 1249 (s, C-O), 1102 (s, Si-O-C) cm^{-1}.

HRMS (ESI$^+$): calcd. for $C_{26}H_{48}O_5Si_2Na$ ([M+Na]$^+$), 519.2932; found, 519.2926.

(2*S*,4*R*,6*E*)-2-(*tert*-Butyldimethylsilyloxy)-1-(4-methoxybenzyloxy)-7-(4,4,5,5-tetramethyl-1,3,2-dioxaborolan-2-yl)-4-triethylsilyloxyhept-6-ene 16.

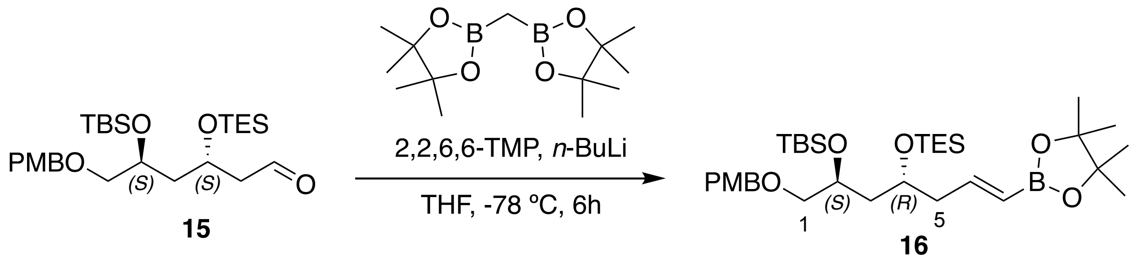

To a cooled (0 °C) solution of 2,2,6,6-TMP (1 mL, 5.88 mmol) in THF (2.1 mL), *n*-BuLi (2.70 mL, 2.20 M in hexanes, 5.88 mmol) was added and the mixture was stirred for 30 min at the same temperature. Then, a solution of bis(pinacolatoboryl)methane (1.57 g, 5.88 mmol) in THF (4.1 mL) was added and the reaction mixture was stirred for 10 min. The mixture was cooled down to -78 °C, a solution of (3*S*,5*S*)-5-(*tert*-butyldimethylsilyloxy)-6-(4-methoxybenzyloxy)-3-(triethylsilyloxy)hexanal **15** (0.73 g, 1.47 mmol) in THF (4.0 mL) was added dropwise, and the reaction mixture was stirred at -78 °C for 6h. A saturated aqueous solution of NH_4Cl was added and the mixture was extracted with Et_2O (3x). The combined organic layers were washed with brine, dried over anhydrous Na_2SO_4, filtered and the solvent was evaporated. The residue was purified by flash column chromatography (C18, CH_3CN) to afford 0.87 g (95% yield) of a yellow oil, which was identified as (2*S*,4*R*,6*E*)-2-(*tert*-butyldimethylsilyloxy)-1-(4-methoxybenzyloxy)-7-(4,4,5,5-tetramethyl-1,3,2-dioxaborolan-2-yl)-4-triethylsilyloxyhept-6-ene **16**.

[α]$_D$^{24} = -8.8 (*c* 1.04, CH_2Cl_2).

^{1}H-NMR (400.16 MHz, C_6D_6): δ 7.23 (d, J = 8.6 Hz, 2H, ArH), 7.08 (app. dt, J = 17.9, 6.9 Hz, 1H, H_6), 6.82 (d, J = 8.6 Hz, 2H, ArH), 5.88 (dt, J = 17.9, 1.4 Hz, 1H, H_7), 4.33 (d, J = 11.6 Hz, 1H, OCH$_2$Ar), 4.28 (d, J = 11.6 Hz, 1H, OCH$_2$Ar), 4.21 – 4.12 (m, 2H, H_2 + H_4), 3.32 (d, J = 5.3 Hz, 2H, 2H_1), 3.30 (s,

3H, OCH_3), 2.47 – 2.43 (m, 2H, $2H_5$), 1.80 (ddd, *J* = 6.8, 5.2, 1.6 Hz, 2H, $2H_3$), 1.09 (s, 12H, $4xCH_3$), 1.05 (s, 9H, $SiC(CH_3)_3$), 1.04 (t, *J* = 8.0 Hz, 9H, $3xSiCH_2C\underline{H_3}$), 0.66 (q, *J* = 8.0 Hz, 6H, 3xSi-$C\underline{H_2}CH_3$), 0.23 (s, 3H, Si-CH_3), 0.21 (s, 3H, Si-CH_3) ppm.

^{13}C-NMR (100.63 MHz, C_6D_6): δ 159.7 (s), 150.9 (d), 130.9 (s), 129.5 (d, 2x), 114.0 (d, 2x), 83.0 (s), 75.7 (t), 73.1 (t), 70.2 (d), 69.7 (d), 54.7 (q), 45.4 (t), 43.6 (t), 26.3 (q, 3x), 25.0 (q, 2x), 24.9 (q, 2x), 18.6 (s), 7.4 (q, 3x), 5.7 (t, 3x), -3.4 (q), -4.3 (q) ppm.

IR (NaCl): υ 2954 (s, C-H), 2934 (s, C-H), 2877 (m, C-H), 2856 (m, C-H), 1638 (m, C=C), 1613 (m, C=C), 1514 (m, C=C), 1249 (s, C-O), 1097 (s, Si-O-C) cm^{-1}.

HRMS (ESI^+): calcd. for $C_{33}H_{61}BO_6Si_2Na$ ($[M+Na]^+$), 643.3992; found, 643.3998.

(2*S*,4*R*,6*E*)-2-(*tert*-Butyldimethylsilyloxy)-7-(4,4,5,5-tetramethyl-1,3,2-dioxaborolan-2-yl)-4-triethylsilyloxyhept-6-en-1-ol 17

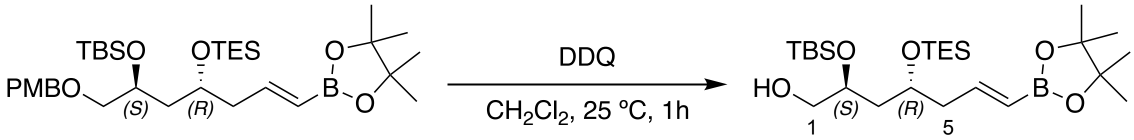

16 **17**

DDQ (1.27 g, 5.48 mmol) was added in small portions to a solution of (2*S*,4*R*,6*E*)-2-(*tert*-butyldimethylsilyloxy)-1-(4-methoxybenzyloxy)-7-(4,4,5,5-tetramethyl-1,3,2-dioxaborolan-2-yl)-4-triethylsilyloxyhept-6-ene **16** (2.43 g, 3.91 mmol) in CH_2Cl_2-water (118 mL, 10:1 v/v) and the reaction mixture was stirred at 25 °C for 1h. Then, a saturated aqueous solution of $NaHCO_3$ was added and the mixture was extracted with CH_2Cl_2 (3x). The combined organic layers were dried over anhydrous Na_2SO_4, filtered and the solvent was evaporated. The residue was purified by flash column chromatography (C18, from 50:50 to 100:0 (v/v) CH_3CN/H_2O) to afford 1.83 g (93% yield) of a yellow oil, which was identified as (2*S*,4*R*,6*E*)-2-(*tert*-butyldimethylsilyloxy)-7-(4,4,5,5-tetramethyl-1,3,2-dioxaborolan-2-yl)-4-triethylsilyloxyhept-6-en-1-ol **17**.

$[\alpha]_D$^{25} = -0.5 (*c* 0.46, CH_2Cl_2).

^{1}H-NMR (400.16 MHz, C_6D_6): δ 7.04 (app. dt, *J* = 17.9, 6.9 Hz, 1H, H_6), 5.85 (dt, *J* = 17.9, 1.4 Hz, 1H, H_7), 3.97 (p, *J* = 5.9 Hz, 1H, H_4), 3.91 – 3.84 (m, 1H, H_2), 3.53 – 3.46 (m, 1H, H_{1A}), 3.42 – 3.35 (m, 1H, H_{1B}), 2.44 – 2.32 (m, 2H, $2H_5$), 1.82 – 1.72 (m, 2H, $2H_3$), 1.54 (t, *J* = 6.2 Hz , 1H, OH), 1.10 (s, 12H, $4xCH_3$), 1.01 (t, *J* = 7.9 Hz, 9H, $3xSiCH_2C\underline{H_3}$), 0.96 (s, 9H, $SiC(CH_3)_3$), 0.63 (q, *J* = 7.9 Hz, 6H, 3xSi-$C\underline{H_2}CH_3$), 0.13 (s, 3H, Si-CH_3), 0.10 (s, 3H, Si-CH_3) ppm.

^{13}C-NMR (100.63 MHz, C_6D_6): δ 150.6 (d), 83.0 (s, 2x), 71.8 (d), 69.8 (d), 67.2 (t), 44.9 (t), 42.6 (t), 26.2 (q, 3x), 25.0 (q, 2x), 24.9 (q, 2x), 18.4 (s), 7.3 (q, 3x), 5.6 (t, 3x), -4.0 (q), -4.2 (q) ppm.

IR (NaCl): υ 3661-3242 (br, O-H), 2954 (s, C-H), 2878 (s, C-H), 1613 (s, C=C), 1361 (s, B-O), 1253 (s, C-O), 1108 (s, Si-O-C) cm^{-1}.

HRMS (ESI⁺): calcd. for $C_{25}H_{53}BO_5Si_2Na$ ($[M+Na]^+$), 523.3417; found, 523.3422.

(2*S*,4*R*,6*E*)-2-(*tert*-Butyldimethylsilyloxy)-7-(4,4,5,5-tetramethyl-1,3,2-dioxaborolan-2-yl)-4-triethylsilyloxyhept-6-enal 7

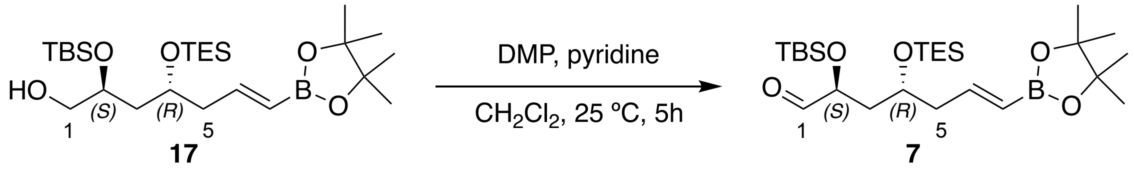

Pyridine (1.2 mL, 14.48 mmol) and DMP (2.46 g, 5.79 mmol) were added to a cooled (0 °C) stirred solution of (2*S*,4*R*,6*E*)-2-(*tert*-butyldimethylsilyloxy)-7-(4,4,5,5-tetramethyl-1,3,2-dioxaborolan-2-yl)-4-triethylsilyloxyhept-6-en-1-ol (*S*,*R*)-**17** (1.45 g, 2.90 mmol) in CH_2Cl_2 (32 mL) and the mixture was stirred at 25 °C for 5h. Then, a saturated aqueous solution of $NaHCO_3$ and $Na_2S_2O_3$ was added, and the mixture was extracted with Et_2O (3x). The combined organic layers were washed with brine, dried over anhydrous Na_2SO_4, filtered and the solvent was evaporated. The residue was purified by flash column chromatography (C18, from 70:30 to 100:0 (v/v) CH_3CN/H_2O) to afford 1.06 g (74% yield) of a yellow oil, which was identified as (2*S*,4*R*,6*E*)-2-(*tert*-butyldimethylsilyloxy)-7-(4,4,5,5-tetramethyl-1,3,2-dioxaborolan-2-yl)-4-triethylsilyloxyhept-6-enal **7**.

$[\alpha]_D^{25}$ = -10.2 (*c* 0.17, CH_2Cl_2).

^{1}H-NMR (400.16 MHz, C_6D_6): δ 9.37 (d, *J* = 1.5 Hz, 1H, H_1), 6.96 (app. dt, *J* = 17.9, 6.9 Hz, 1H, H_6), 5.83 (dt, *J* = 17.9, 1.4 Hz, 1H, H_7), 4.13 (ddd, *J* = 7.3, 4.9, 1.5 Hz, 1H, H_2), 4.05 (ddt, *J* = 6.4, 5.2, 3.2 Hz, 1H, H_4), 2.42 (app. dtd, *J* = 6.9, 5.5, 1.5 Hz, 2H, $2H_5$), 1.81 (ddd, *J* = 14.2, 6.8, 4.9 Hz, 1H, H_{3A}), 1.71 (ddd, *J* = 14.2, 7.3, 4.5 Hz, 1H, H_{3B}), 1.08 (s, 12H, $4xCH_3$), 0.99 (t, *J* = 7.8 Hz, 9H, $3xSiCH_2C\underline{H}_3$), 0.95 (s, 9H, $SiC(CH_3)_3$), 0.61 (q, *J* = 7.8 Hz, 6H, $3xSi\text{-}C\underline{H_2}CH_3$), 0.07 (s, 3H, $Si\text{-}CH_3$), 0.06 (s, 3H, $Si\text{-}CH_3$) ppm.

^{13}C-NMR (100.63 MHz, C_6D_6): δ 201.6 (d), 150.3 (d), 83.0 (s, 2x), 76.4 (d), 69.0 (d), 44.8 (t), 40.3 (t), 26.1 (q), 24.9 (q, 4x), 18.4 (s), 7.3 (q, 3x), 5.6 (t, 3x), -4.0 (q), -4.6 (q) ppm.

IR (NaCl): υ 2955 (s, C-H), 2933 (m, C-H), 2878 (m, C-H), 2859 (w, C-H), 1738 (s, C=O), 1639 (m, C=C), 1361 (s, C-O), 1106 (m, Si-O-C) cm^{-1}.

HRMS (ESI⁺): calcd. for $C_{25}H_{51}BO_5Si_2Na$ ($[M+Na]^+$), 521.3260; found, 521.3262.

(2*Z*,4*S*,6*R*,8*E*)-Ethyl 4-(*tert*-Butyldimethylsilyloxy)-9-(4,4,5,5-tetramethyl-1,3,2-dioxaborolan-2-yl)-6-triethylsilyloxyhepta-2,8-dienoate 6

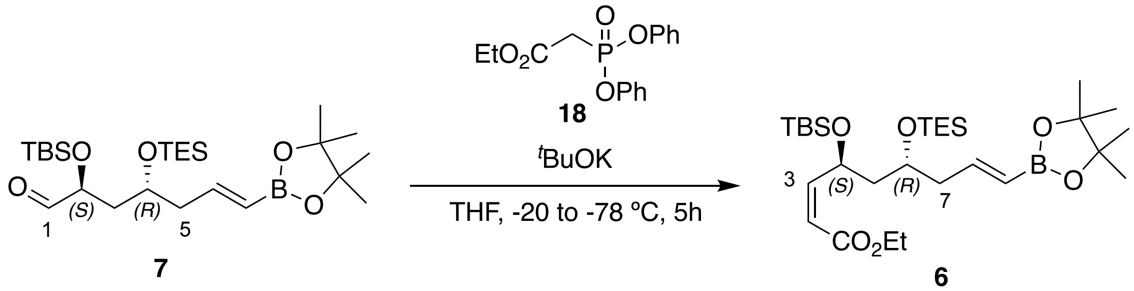

To a cooled (-20 °C) stirred solution of ethyl 2-(diphenoxyphosphoryl)acetate **18** (135 mg, 0.42 mmol) in THF (4 mL), ^{t}BuOK (0.26 mL, 1.65 M in THF, 0.42 mmol) was added and the reaction mixture was stirred for 30 min. Then, it was cooled down to -78 °C and a solution of (2*S*,4*R*,*E*)-2-(*tert*-butyldimethylsilyloxy)-7-(4,4,5,5-tetramethyl-1,3,2-dioxaborolan-2-yl)-4-triethylsilyloxyhept-6-enal **7** (0.20 g, 0.40 mmol) in THF (0.5 mL) was added and the reaction mixture was stirred at the same temperature for 5h. The reaction was quenched by addition of an aqueous saturated solution of NH_4Cl and the mixture was extracted with Et_2O (3x). The combined organic layers were washed with brine, dried over anhydrous Na_2SO_4, filtered and the solvent was evaporated. The residue was purified by flash column chromatography (C18, from 70:30 to 100:0 (v/v) CH_3CN/H2O) to afford 0.20 g (89% yield) of a yellow oil, which was identified as (2*Z*,4*S*,6*R*,8*E*)-ethyl 4-(*tert*-butyldimethylsilyloxy)-9-(4,4,5,5-tetramethyl-1,3,2-dioxaborolan-2-yl)-6-triethylsilyloxyhepta-2,8-dienoate **6**.

[α]$_D$^{25} = +0.6 (*c* 0.51, CH_2Cl_2).

^{1}H-NMR (400.16 MHz, $CDCl_3$): δ 6.62 (app. dt, *J* = 17.9, 7.2 Hz, 1H, H_8), 6.07 (dd, *J* = 11.7, 8.8 Hz, 1H, H_3), 5.69 (dd, *J* = 11.7, 1.1 Hz, 1H, H_2), 5.48 (dt, *J* = 18.0, 1.4 Hz, 1H, H_9), 5.39 (app. q, *J* = 6.8 Hz, 1H, H_4), 4.21 – 4.12 (m, 2H, $OC\underline{H}_2CH_3$), 3.79 (app. ddd, *J* = 11.8, 6.9, 4.9 Hz, 1H, H_6), 2.53 – 2.44 (m, 1H, H_{7A}), 2.37 – 2.27 (m, 1H, H_{7B}), 1.80 (app. dt, *J* = 14.0, 7.1 Hz, 1H, H_{5A}), 1.64 – 1.55 (m, 1H, H_{5B}), 1.28 (t, *J* = 7.1 Hz, 3H, $OCH_2C\underline{H}_3$), 1.25 (s, 4xCH_3), 0.93 (t, *J* = 7.9 Hz, 9H, 3x$SiCH_2C\underline{H}_3$), 0.86 (s, 9H, $SiC(CH_3)_3$), 0.58 (q, *J* = 7.9 Hz, 6H, 3x$SiC\underline{H}_2CH_3$), 0.05 (s, 3H, Si-CH_3), 0.00 (s, 3H, Si-CH_3) ppm.

^{13}C-NMR (100.63 MHz, $CDCl_3$): δ 165.8 (s), 152.2 (d), 151.4 (d), 118.3 (d), 83.1 (s, 2x), 69.0 (d), 66.0 (d), 60.3 (t), 45.8 (t), 44.6 (t), 26.0 (q, 3x), 24.9 (q, 4x), 18.2 (s), 14.4 (q), 7.1 (q, 3x), 5.2 (t, 3x), -4.1 (q), -4.6 (q) ppm.

IR (NaCl): υ 2955 (s, C-H), 2934 (m, C-H), 2877 (m, C-H), 2858 (w, C-H), 1721 (s, C=O), 1639 (m, C=C), 1362 (s, C-O), 1085 (m, Si-O-C) cm^{-1}.

HRMS (ESI^+): calcd. for $C_{29}H_{57}BO_6Si_2Na$ ($[M+Na]^+$), 591.3679; found, 591.3680.

(2*Z*,4*S*,6*R*,8*E*,10*E*)-Ethyl 4,12-bis(*tert*-Butyldimethylsilyloxy)-6-triethylsilyloxydodeca-2,8,10-trienoate 20

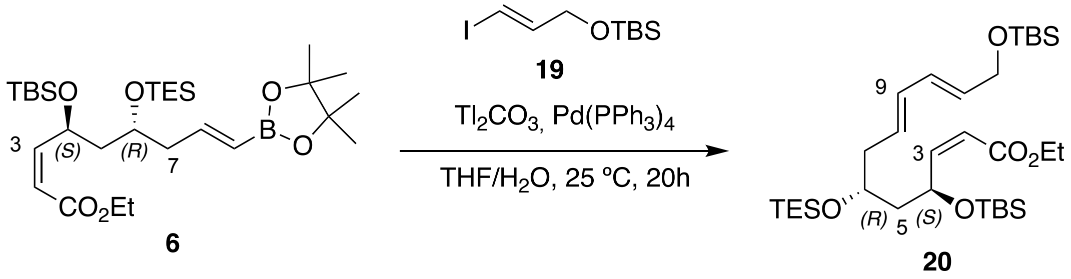

To a cooled (0 °C) solution of (2*Z*,4*S*,6*S*,8*E*)-ethyl 4-(*tert*-butyldimethylsilyloxy)-9-(4,4,5,5-tetramethyl-1,3,2-dioxaborolan-2-yl)-6-triethylsilyloxyhepta-2,8-dienoate **6** (0.30 g, 0.53 mmol) and (*E*)-3-iodo-1-(*tert*-butyldimethylsilyloxy)prop-2-ene **19** (0.24 g, 0.79 mmol) in THF-water (18 mL, 4:1 v/v), Tl_2CO_3 (0.74 g, 1.58 mmol) and $Pd(PPh_3)_4$ (0.06 g, 0.05 mmol) were added and the reaction mixture was stirred at 25 °C for 20h. Then, the reaction mixture was filtered through a mixture of Celite® and anhydrous Na_2SO_4 and the solvent was evaporated. The residue was purified by flash column chromatography (C18, from 100:0 to 60:40 (v/v) CH_3CN/CH_2Cl_2) to afford 0.27 g (82% yield) of a yellow oil, which was identified as (2*Z*,4*S*,6*S*,8*E*,10*E*)-ethyl 4,12-bis(*tert*-butyldimethylsilyloxy)-6-triethylsilyloxydodeca-2,8,10-trienoate **20**.

$[\alpha]_D$^{22} = -15.1 (*c* 0.49, CH_2Cl_2).

^{1}H-NMR (400.16 MHz, C_6D_6): δ 6.42 – 6.23 (m, 2H, $H_9 + H_{10}$), 5.98 (dd, *J* = 11.6, 8.9 Hz, 1H, H_3), 5.98 – 5.89 (m, 1H, H_8), 5.91 – 5.78 (m, 1H, H_4), 5.69 (dt, *J* = 14.8, 4.9 Hz, 1H, H_{11}), 5.66 (dd, *J* = 11.7, 1.0 Hz, 1H, H_2), 4.10 (d, *J* = 4.9 Hz, 2H, $2H_{12}$), 4.05 (app. p, *J* = 6.1, 5.5 Hz, 1H, H_6), 3.95 (app. qd, *J* = 7.1, 2.9 Hz, 2H, $OC\underline{H}_2CH_3$), 2.61 (app. dt, *J* = 12.7, 6.1 Hz, 1H, H_{7A}), 2.50 (app. dt, *J* = 13.8, 6.7 Hz, 1H, H_{7B}), 2.05 (app. dt, *J* = 13.7, 6.9 Hz, 1H, H_{5A}), 1.85 (app. dt, *J* = 13.6, 5.6 Hz, 1H, H_{5B}), 1.08 (t, *J* = 7.9 Hz, 9H, $3xSiCH_2C\underline{H}_3$), 1.01 (s, 9H, $SiC(CH_3)_3$), 0.99 (s, 9H, $SiC(CH_3)_3$), 0.94 (t, *J* = 7.1 Hz, 3H, $OCH_2C\underline{H}_3$), 0.71 (q, *J* = 8.1 Hz, 6H, $3xSiC\underline{H}_2CH_3$), 0.22 (s, 3H, $Si\text{-}CH_3$), 0.14 (s, 3H, $Si\text{-}CH_3$), 0.06 (s, 6H, $2xSi\text{-}CH_3$) ppm.

^{13}C-NMR (100.63 MHz, C_6D_6): δ 165.6 (s), 152.8 (d), 132.8 (d), 131.3 (d), 130.7 (d), 130.5 (d), 118.5 (d), 69.7 (d), 66.2 (d), 63.8 (t), 60.1 (t), 46.0 (t), 41.7 (t), 26.1 (q, 6x), 18.6 (s), 18.3 (s), 14.2 (q), 7.4 (q, 3x), 5.7 (t, 3x), -3.9 (q), -4.5 (q), -5.0 (q, 2x) ppm.

IR (NaCl): υ 2952 (s, C-H), 2856 (s, C-H), 1701 (s, C=O), 1472 (s, C=C), 1253 (s, C-O), 1006 (s, Si-O-C) cm^{-1}.

HRMS (ESI$^+$): calcd. for $C_{32}H_{64}O_5Si_3Na$ ([M+Na]$^+$), 635.3954; found, 635.3962.

(2*Z*,4*S*,6*R*,8*E*,10*E*)-4,12-bis(*tert*-Butyldimethylsilyloxy)-6-triethylsilyloxydodeca-2,8,10-trien-1-ol 21

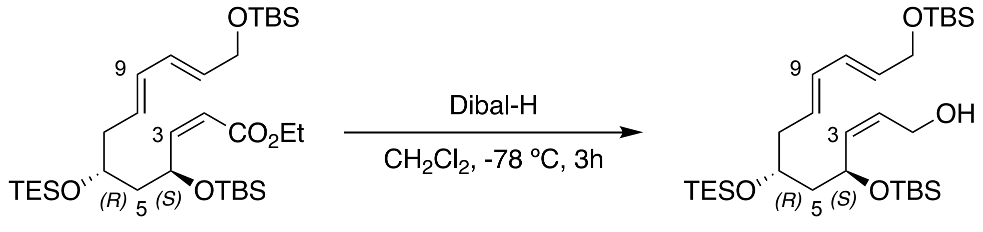

To a cooled (-78 °C) stirred solution of (2*Z*,4*S*,6*R*,8*E*,10*E*)-ethyl 4,12-bis(*tert*-butyldimethylsilyloxy)-6-triethylsilyloxydodeca-2,8,10-trienoate **20** (0.15 g, 0.24 mmol) in CH_2Cl_2 (3.2 mL), Dibal-H (0.52 mL, 1 M in hexanes, 0.52 mmol) was added. After stirring for 3h, a saturated aqueous solution of Rochelle's salt and Et_2O were added, and the resulting mixture was stirred for 2h. Then, the mixture was extracted with Et_2O (3x), the combined organic layers were washed with brine, dried over anhydrous Na_2SO_4 and the solvent was evaporated. The residue was purified by flash column chromatography (C18, from 100:0 to 50:50 (v/v) CH_3CN/CH_2Cl_2) to afford 0.12 g (88% yield) of a yellow oil, which was identified as (2*Z*,4*S*,6*R*,8*E*,10*E*)-4,12-bis(*tert*-butyldimethylsilyloxy)-6-triethylsilyloxydodeca-2,8,10-trien-1-ol **21**.

[α]$_D$^{22} = -13.4 (*c* 0.66, CH_2Cl_2).

^{1}H-NMR (400.16 MHz, C_6D_6): δ 6.39 – 6.28 (m, 1H, H_{10}), 6.16 (dd, *J* = 15.1, 10.5 Hz, 1H, H_9), 5.77 (app. dd, *J* = 15.0, 7.4 Hz, 1H, H_8), 5.72 – 5.64 (m, 1H, H_{11}), 5.54 – 5.47 (m, 1H, H_3), 5.42 (dt, *J* = 11.4, 6.0 Hz, 1H, H_2), 4.76 (app. td, *J* = 8.4, 4.8 Hz, 1H, H_4), 4.10 (d, *J* = 5.7 Hz, 2H, $2H_{12}$), 4.12 – 3.99 (m, 3H, H_6 + $2H_1$), 2.36 (app. t, *J* = 6.7 Hz, 2H, $2H_7$), 1.98 – 1.88 (m, 1H, H_{5A}), 1.70 – 1.54 (m, 1H, H_{5B}), 1.06 (t, *J* = 7.9 Hz, 9H, 3xSiCH$_2$C$\underline{H}_3$), 1.01 (s, 9H, SiC(CH$_3$)$_3$), 0.99 (s, 9H, 9H, SiC(CH$_3$)$_3$), 0.69 (q, *J* = 7.9 Hz, 6H, 3xSiC$\underline{H}_2$CH$_3$), 0.16 (s, 3H, Si-CH$_3$), 0.13 (s, 3H, Si-CH$_3$), 0.06 (s, 6H, 2xSi-CH$_3$) ppm.

^{13}C-NMR (100.63 MHz, C_6D_6): δ 135.8 (d), 132.8 (d), 131.6 (d), 130.2 (d), 130.1 (d), 129.0 (d), 69.7 (d), 66.3 (d), 63.7 (t), 58.9 (t), 46.7 (t), 41.8 (t), 26.2 (q, 3x), 26.1 (q, 3x), 18.6 (s), 18.4 (s), 7.3 (q, 3x), 5.7 (t, 3x), -3.4 (q), -4.4 (q), -5.0 (q, 2x) ppm.

IR (NaCl): υ 3565-3205 (br, O-H), 2954 (s, C-H), 2857 (s, C-H), 1462 (s, C=C), 1254 (s, C-O), 1084 (s, Si-O-C) cm^{-1}.

HRMS (ESI$^+$): calcd. for $C_{30}H_{62}O_4Si_3Na$ ([M+Na]$^+$), 593.3848; found, 593.3848.

(2*Z*,4*S*,6*R*,8*E*,10*E*)-4,12-bis(*tert*-Butyldimethylsilyloxy)-6-triethylsilyloxydodeca-2,8,10-trienal 5

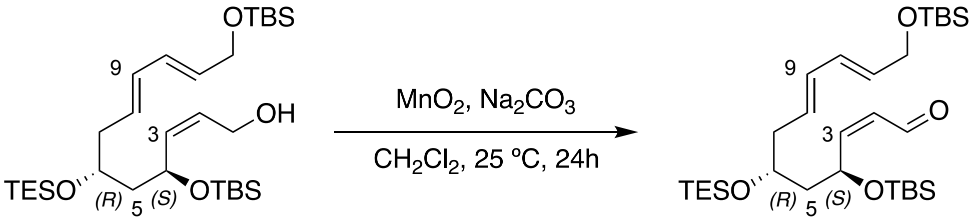

21 **5**

To a stirred suspension of MnO_2 (0.61 g, 6.30 mmol) and Na_2CO_3 (0.67 g, 6.30 mmol) in CH_2Cl_2 (7 mL), (2*Z*,4*S*,6*R*,8*E*,10*E*)-4,12-bis(*tert*-butyldimethylsilyloxy)-6-triethylsilyloxydodeca-2,8,10-trien-1-ol **21** (0.20 g, 0.35 mmol) was added and the reaction mixture was stirred at 25 °C for 24h. Then, the reaction mixture was filtered through Celite® and the solvent was evaporated to afford 0.20 g (99% yield) of a yellow oil, which was identified as (2*Z*,4*S*,6*R*,8*E*,10*E*)-4,12-bis(*tert*-butyldimethylsilyloxy)-6-triethylsilyloxydodeca-2,8,10-trienal **5**.

$[\alpha]_D$^{22} = -22.6 (*c* 0.18, CH_2Cl_2).

^{1}H-NMR (400.16 MHz, $CDCl_3$): δ 10.10 (d, *J* = 7.9 Hz, 1H, H_1), 6.53 (dd, *J* = 11.4, 9.0 Hz, 1H, H_3), 6.18 (dd, *J* = 15.1, 10.6 Hz, 1H, H_{10}), 6.05 (dd, *J* = 15.1, 10.5 Hz, 1H, H_9), 5.87 (ddd, *J* = 11.4, 7.9, 1.1 Hz, 1H, H_2), 5.66 (dt, *J* = 15.1, 5.3 Hz, 1H, H_{11}), 5.59 (app. dt, *J* = 14.7, 7.3 Hz, 1H, H_8), 5.18 (app. td, *J* = 8.6, 3.9 Hz, 1H, H_4), 4.20 (dd, *J* = 5.3, 1.7 Hz, 2H, $2H_{12}$), 3.95 – 3.87 (m, 1H, H_6), 2.32 – 2.26 (m, 2H, $2H_7$), 1.82 (ddd, *J* = 13.9, 8.4, 4.0 Hz, 1H, H_{5A}), 1.53 (ddd, *J* = 13.9, 7.6, 4.1 Hz, 1H, H_{5B}), 0.96 (t, *J* = 8.1 Hz, 9H, $3xSiCH_2C\underline{H}_3$), 0.91 (s, 9H, $SiC(CH_3)_3$), 0.88 (s, 9H, $SiC(CH_3)_3$), 0.60 (q, *J* = 8.1 Hz, 6H, $3xSiC\underline{H}_2CH_3$), 0.07 (s, 9H, $3xSi\text{-}CH_3$), 0.02 (s, 3H, $Si\text{-}CH_3$) ppm.

^{13}C-NMR (100.63 MHz, $CDCl_3$): δ 191.1 (d), 155.1 (d), 132.5 (d), 131.2 (d), 130.0 (d), 129.4 (d), 128.0 (d), 68.9 (d), 65.7 (d), 63.7 (t), 46.3 (t), 41.5 (t), 26.1 (q, 3x), 25.9 (q, 3x), 18.6 (s), 18.2 (s), 7.1 (q, 3x), 5.5 (t, 3x), -3.6 (q), -4.4 (q), -5.1 (q, 2x) ppm.

IR (NaCl): υ 2954 (s, C-H), 2879 (s, C-H), 1687 (s, C=O), 1471 (s, C=C), 1254 (s, C-O), 1088 (s, Si-O-C) cm^{-1}.

HRMS (ESI^+): calcd. for $C_{30}H_{60}O_4Si_3Na$ ($[M+Na]^+$), 591.3692; found, 591.3696.

(1*R*,2*R*,4a*S*,6*R*,8*S*,8a*S*)-8-(*tert*-Butyldimethylsilyloxy)-2-((*tert*-butyldimethylsilyloxy)methyl)-6-(triethylsilyloxy)-1,2,4a,5,6,7,8,8a-octahydronaphthalene-1-carbaldehyde *Re*-*endo*-22

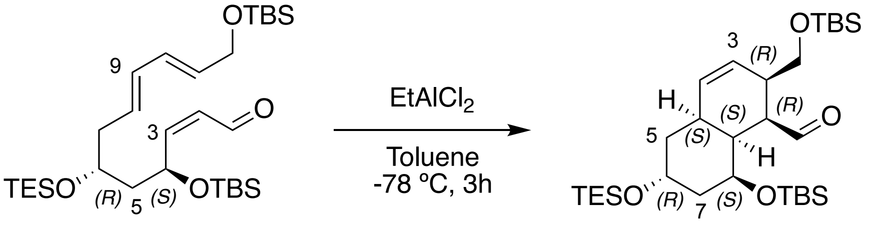

5 *Re-endo*-**22**

To a cooled (-78 °C) stirred solution of (2*Z*,4*S*,6*R*,8*E*,10*E*)-4,12-bis(*tert*-butyldimethylsilyloxy)-6-triethylsilyloxydodeca-2,8,10-trien-1-ol **5** (0.14 g, 0.25 mmol) in toluene (4.2 mL), $EtAlCl_2$ (0.27 mL, 1 M in hexanes, 0.27 mmol) was added and the reaction mixture was stirred at the same temperature for 3h. Then, a saturated aqueous solution of Rochelle's salts and $NaHCO_3$ were added and the mixture was stirred for 30 min and then, the mixture was extracted with Et_2O (3x). The combined organic layers were washed with brine, dried over anhydrous Na_2SO_4, filtered and the solvent was evaporated. The residue was purified by flash column chromatography (C18, from 90:10 to 100:0 (v/v) MeOH/H_2O) to afford 0.11 g (78% yield) of a yellow oil, which was identified as (1*R*,2*R*,4a*S*,6*R*,8*S*,8a*S*)-8-(*tert*-butyldimethylsilyloxy)-2-((*tert*-butyldimethylsilyloxy)methyl)-6-(triethylsilyloxy)-1,2,4a,5,6,7,8,8a-octahydronaphthalene-1-carbaldehyde *Re*-*endo*-**22**.

$[\alpha]_D$^{23} = -24.2 (*c* 0.44, CH_2Cl_2).

^{1}H-NMR (400.16 MHz, C_6D_6): δ 10.22 (d, *J* = 4.4 Hz, 1H, CHO), 5.70 - 5.65 (m, 1H, H_3), 5.65 – 5.61 (m, 1H, H_4), 4.53 (ddd, *J* = 11.7, 6.5, 4.9 Hz, 1H, H_8), 3.80 (app. s, 1H, H_6), 3.70 (dd, *J* = 9.8, 8.3 Hz, 1H, -OCH_{2A}), 3.66 - 3.61 (m, 1H, -OCH_{2B}), 3.60 – 3.56 (m, 1H, H_1), 2.90 – 2.80 (m, 1H, H_{4a}), 2.59 – 2.55 (m, 1H, H_2), 2.49 (app. q, *J* = 5.5 Hz, 1H, H_{8a}), 1.79 (app. d, *J* = 13.7 Hz, 1H, H_{7A}), 1.59 – 1.53 (m, 1H, H_{5A}), 1.54 – 1.42 (m, 1H, H_{7B}), 1.14 (app. td, *J* = 13.7, 2.4 Hz, 1H, H_{5B}), 1.05 (s, 9H, $SiC(CH_3)_3$), 0.99 (t, *J* = 7.9 Hz, 9H, 3x$SiCH_2C\underline{H}_3$), 0.99 (s, 9H, $SiC(CH_3)_3$), 0.55 (q, *J* = 7.9 Hz, 6H, 3x$SiC\underline{H}_2CH_3$), 0.15 (s, 3H, Si-CH_3), 0.11 (s, 3H, Si-CH_3), 0.04 (s, 3H, Si-CH_3), 0.04 (s, 3H, Si-CH_3) ppm.

^{13}C-NMR (100.63 MHz, C_6D_6): δ 204.4 (d), 131.8 (d), 126.6 (d), 67.9 (d), 67.6 (d), 64.4 (t), 46.6 (d), 45.9 (d), 43.1 (d), 39.8 (t), 36.8 (t), 31.6 (d), 26.2 (q, 3x), 26.1 (q, 3x), 18.4 (s, 2x), 7.2 (q, 3x), 5.1 (t, 3x), -4.4 (q), -4.6 (q), -5.4 (q), -5.5 (q) ppm.

IR (NaCl): υ 2953 (s, C-H), 2929 (s, C-H), 2877 (s, C-H), 2857 (s, C-H), 1717 (m, C=O), 1462 (m, C=C), 1252 (m, C-O), 1092 (s, Si-O-C) cm^{-1}.

HRMS (ESI^+): calcd. for $C_{30}H_{60}O_4Si_3Na$ ($[M+Na]^+$), 591.3692; found, 591.3710.

Benzyl 2-(Diphenoxyphosphoryl)acetate 25

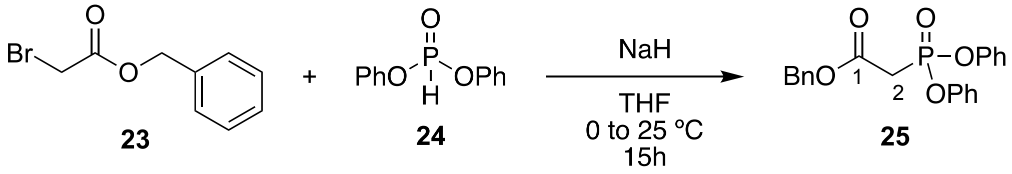

A three-necked flask equipped with a 250-mL dropping funnel was charged with NaH (5.98 g, 149.45 mmol) and THF (115 mL). Diphenyl phosphite **24** (29 mL, 149.45 mmol) was added dropwise to the stirred suspension at 0 °C during a period of 3h. After stirring for 1h at 0 °C, the gas evolution had ceased, and the reaction mixture became a clear orange solution. Subsequently benzyl bromoacetate **23** (24 mL, 149.45 mmol) was added dropwise over a period of 3h, while a white solid started to precipitate. The cooling bath was removed, and the reaction mixture was stirred at 25 °C for a further 12h. Then, a saturated aqueous solution of NH_4Cl and H_2O were added, and the mixture was extracted with Et_2O (3x). The combined organic layers were dried over anhydrous Na_2SO_4, filtered and the solvent was evaporated. The residue was purified by flash column chromatography (silica gel, 75:25 (v/v) *n*-hexane/EtOAc) to afford 39.6 g (70% yield) of a yellow oil, which was identified as benzyl 2-(diphenoxyphosphoryl)acetate **25**.

^{1}H-NMR (400.16 MHz, $CDCl_3$): δ 7.40 – 7.10 (m, 15H, ArH), 5.22 (s, 2H, OCH_2), 3.31 (d, $^2J_{P\text{-}H}$ = 21.6 Hz, 2H, $2H_2$) ppm.

^{13}C-NMR (100.63 MHz, $CDCl_3$): δ 164.8 (s, $^2J_{P\text{-}C}$ = 6.5 Hz), 150.1 (s, $^2J_{P\text{-}C}$ = 6.5 Hz, 2x), 135.2 (s), 130.0 (d, $^5J_{P\text{-}C}$ = 1.0 Hz, 2x), 129.9 (d), 128.8 (d, 2x), 128.7 (d, 2x), 125.7 (d, $^4J_{P\text{-}C}$ = 1.3 Hz, 4x), 120.8 (d, $^3J_{P\text{-}C}$ = 4.4 Hz, 4x), 67.8 (t), 34.2 (t, $J_{P\text{-}C}$ = 137.2 Hz) ppm.

^{31}P-NMR (162 MHz, $CDCl_3$): δ 12.4 ppm.

IR (NaCl): υ 3066 (s, C-H), 1738 (s, C=O), 1490 (m, C=C), 1287 (s, C-O), 1187 (s, P=O), 943 (s, P-C) cm^{-1}.

HRMS (ESI^+): calcd. for $C_{21}H_{19}O_5P$ ($[M+H]^+$), 383.1043; found, 383.1045.

2-(Diphenoxyphosphoryl)acetic Acid 26

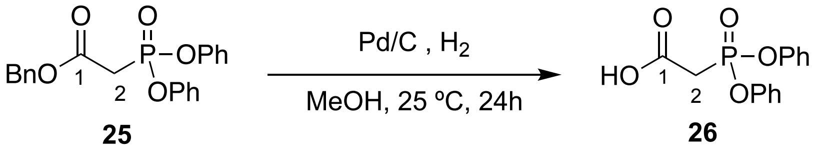

Benzyl ester **25** (3.00 g, 8.00 mmol) and Pd/C (0.30 g, 3.00 mmol) were stirred in methanol (78 mL) under H_2 at 1 atm and 25 °C for 24h. The mixture was filtered through Celite® and the solvent was

evaporated to afford 2.27 g (99% yield) of a white solid, which was identified as 2-(diphenoxyphosphoryl)acetic acid **26**.

m.p.: 137 – 140 °C (CH_2Cl_2)

^{1}H-NMR (400.16 MHz, $CDCl_3$): δ 7.40 – 7.20 (m, 10H, ArH), 3.30 (d, $^{2}J_{P-H}$ = 21.7 Hz, 2H, 2H_2) ppm.

^{13}C-NMR (100.63 MHz, $CDCl_3$): δ 167.2 (s $^{2}J_{P-C}$ = 5.8 Hz), 150.0 (s, $^{2}J_{P-C}$ = 8.6 Hz, 2x), 130.1 (d, $^{5}J_{P-C}$ = 1.0 Hz, 2x), 125.9 (d, $^{4}J_{P-C}$ = 1.4 Hz, 4x), 120.8 (d, $^{3}J_{P-C}$ = 4.4 Hz, 4x), 33.7 (t, $^{1}J_{P-C}$ = 137.0 Hz) ppm.

^{31}P-NMR (162 MHz, $CDCl_3$): δ 12.9 ppm.

IR (NaCl): υ 3446 (s, O-H), 1733 (s, C=O), 946 (m, OC-OH), 905 (s, P-C) cm^{-1}.

HRMS (ESI^+): calcd. for $C_{14}H_{13}O_5P$ ($[M+H]^+$), 293.0573; found, 293.0575.

(*E*)-3-Iodoallyl-2-(diphenoxyphosphoryl)acetate 28

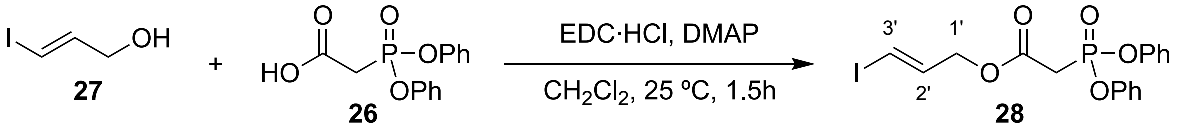

To a stirred solution of iodide **27** (1.20 g, 6.52 mmol) and 2-(diphenoxyphosphoryl)acetic acid **26** (2.29 g, 7.83 mmol) in CH_2Cl_2 (130 mL), EDC·HCl (1.75 g, 9.13 mmol) and DMAP (0.04 g, 0.33 mmol) were added and the reaction mixture was stirred at 25 °C for 1.5h. Then, the reaction mixture was filtered, and the solvent was evaporated. The residue was purified by flash column chromatography (silica gel, from 98:2 (v/v) *n*-hexane/Et_3N to 70:30 (v/v) *n*-hexane/EtOAc) to afford 2.08 g (70% yield) of a colourless oil, which was identified as (*E*)-3-iodoallyl-2-(diphenoxyphosphoryl)acetate **28**.

^{1}H-NMR (400.16 MHz, C_6D_6): δ 7.28 (d, *J* = 7.7 Hz, 4H, ArH), 6.97 (t, *J* = 8.0 Hz, 4H, ArH), 6.85 – 6.77 (m, 2H, ArH), 6.15 (dt, *J* = 14.7, 5.9 Hz, 1H, $H_{2'}$), 6.03 (d, J=14.6 Hz, 1H, $H_{3'}$), 3.98 (d, *J* = 5.9 Hz, 2H, 2$H_{1'}$), 2.84 (d, $^{2}J_{P-H}$ =21.6 Hz, 2H, 2H_2) ppm.

^{13}C-NMR (100.63 MHz, C_6D_6): δ 164.14 (s, $^{2}J_{P-C}$ = 6.4 Hz), 150.7 (s, $^{2}J_{P-C}$ = 8.3 Hz), 139.0 (d), 130.1 (d, 2x), 125.6 (d, 4x), 121.1 (d, $^{3}J_{P-C}$ = 4.5 Hz, 4x), 81.7 (d), 66.4 (t), 34.0 (t, $^{1}J_{P-C}$ = 136.0 Hz) ppm.

^{31}P-NMR (162 MHz, C_6D_6): δ 12.0 ppm.

IR (NaCl): υ 3650 – 3000 (br, OH), 1642 (s, C=C), 1187 (s, P=O), 940 (s, P-C), 772 (s, C-I), 688 (m, C-I) cm^{-1}.

HRMS (ESI^+): calcd. for $C_{17}H_{16}IO_5P$ ($[M+H]^+$), 458.9853; found, 458.9855.

(2*E*,4*E*,7*R*,9*S*)-9-(*tert*-Butyldimethylsilyloxy)-10-hydroxy-7-(triethylsilyloxy)deca-2,4-dien-1-yl 2-(diphenoxyphosphoryl)acetate 29

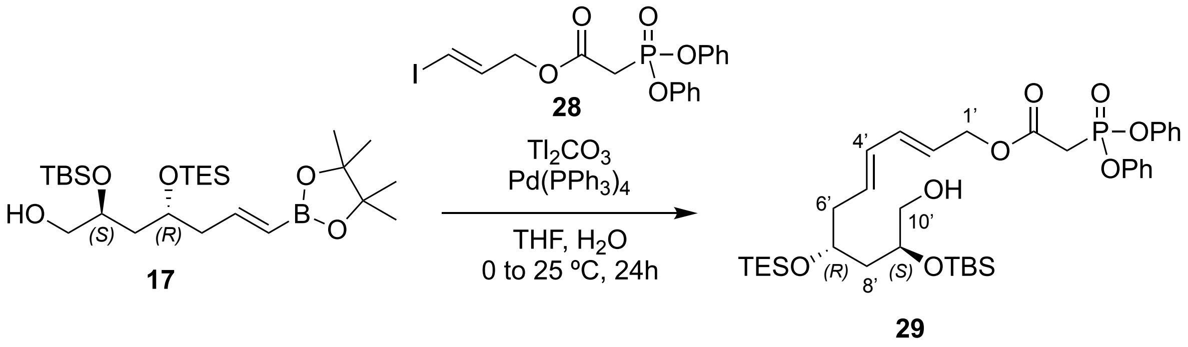

To a cooled (0 °C) solution of (*E*)-3-iodoallyl-2-(diphenoxyphosphoryl)acetate **28** (0.45 g, 0.99 mmol) and (2*S*,4*R*,6*E*)-2-(*tert*-butyldimethylsilyloxy)-7-(4,4,5,5-tetramethyl-1,3,2-dioxaborolan-2-yl)-4-triethylsilyloxyhept-6-enal **17** (0.33 g, 0.66 mmol) in THF–H_2O (22 mL, 4:1 v/v), Tl_2CO_3 (0.93 g, 1.98 mmol) and $Pd(PPh_3)_4$ (0.08 mg, 0.07 mmol) were added and the mixture was stirred at 25 °C for 20h. Then, water was added, and the mixture was extracted with Et_2O (3x). The combined organic layers were washed with a saturated aqueous solution of $NaHCO_3$, brine, dried over anhydrous Na_2SO_4, filtered and the solvent was evaporated. The residue was purified by flash column chromatography (C18, from 70:30 to 100:0 (v/v) CH_3CN/H_2O) to afford 0.38 g (81% yield) of a yellow oil, which was identified as (2*E*,4*E*,7*R*,9*S*)-9-(*tert*-butyldimethylsilyloxy)-10-hydroxy-7-(triethylsilyloxy)deca-2,4-dien-1-yl-2-(diphenoxyphosphoryl)acetate **29**.

[α]$_D$^{21} = -17.5 (*c* 0.06, CH_2Cl_2).

^{1}H-NMR (400.16 MHz, C_6D_6): δ 7.35 – 7.25 (m, 4H, ArH), 7.00 – 6.95 (m, 4H, ArH), 6.90 – 6.80 (m, 2H, ArH), 6.10 (t, *J* = 12.4 Hz, 1H, $H_{3'}$), 5.94 (t, *J* = 14.2 Hz, 1H, $H_{4'}$), 5.75 – 5.65 (m, 1H, $H_{5'}$), 5.55 – 5.45 (m, 1H, $H_{2'}$), 4.44 (d, *J* = 6.5 Hz, 2H, $2H_{1'}$), 4.00 – 3.85 (m, 2H, $H_{7'}$ + $H_{9'}$), 3.53 (dd, *J* = 10.9, 4.2 Hz, 1H, $H_{10'A}$), 3.45 (dd, *J* = 11.0, 5.5 Hz, 1H, $H_{10'B}$), 2.94 (d, $^2J_{P\text{-}H}$ = 21.6 Hz, 2H, $2H_2$), 2.31 – 2.22 (m, 2H, $2H_{6'}$), 1.82 – 1.71 (m, 2H, $2H_{8'}$), 0.99 (t, *J* = 7.9 Hz, 9H, 3xSiCH$_2$C$\underline{H}_3$), 0.94 (s, 9H, SiC(CH_3)$_3$), 0.63 (q, *J* = 7.8 Hz, 6H, 3xSiC$\underline{H}_2$CH$_3$), 0.12 (s, 3H, Si-CH_3), 0.10 (s, 3H, Si-CH_3) ppm.

^{13}C-NMR (100.63 MHz, C_6D_6): δ 164.6 (s, $^2J_{P\text{-}C}$ = 6.6 Hz), 150.9 (s, $^2J_{P\text{-}C}$ = 4.5 Hz), 135.1 (d), 132.2 (d, 2x), 130.0 (d, 4x), 125.5 (d, 2x), 124.7 (d), 121.2 (d, $^3J_{P\text{-}C}$ = 4.5 Hz), 71.7 (d), 70.0 (d), 67.3 (t), 66.0 (t), 42.6 (t), 41.7 (t), 34.2 (t, $^1J_{P\text{-}C}$ = 135.8 Hz), 26.1 (q, 3x), 18.3 (s), 7.2 (q, 3x), 5.7 (t, 3x), -4.1 (q), -4.3 (q) ppm.

^{31}P-NMR (162 MHz, C_6D_6): δ 25.8 ppm.

IR (NaCl): υ 3610 – 3280 (br, OH), 2953 (s, C-H), 2933 (s, C-H), 2877 (m, C-H), 2856 (m, C-H), 1741 (m, C=O), 1490 (s, C=C), 1256 (m, C-O), 1071 (m, Si-O-C) cm^{-1}.

HRMS (ESI^+): calcd. for $C_{36}H_{58}O_8PSi_2$ ($[M+H]^+$), 705.3402; found, 705.3403.

(2*E*,4*E*,7*R*,9*S*)-9-(*tert*-Butyldimethylsilyloxy)-10-oxo-7-(triethylsilyloxy)deca-2,4-dien-1-yl 2-(diphenoxyphosphoryl)acetate 30

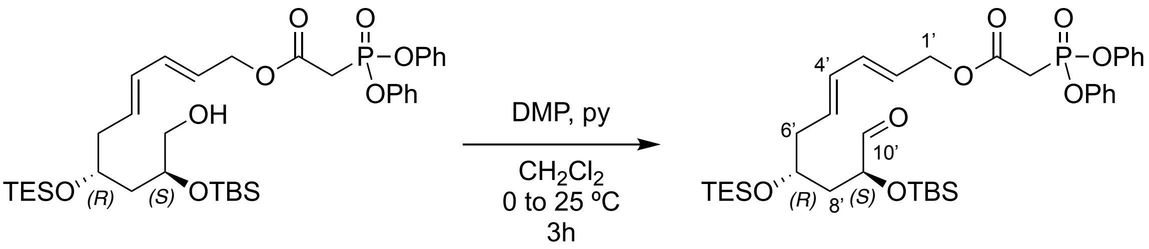

29 **30**

To a cooled (0 °C) solution of (2*E*,4*E*,7*R*,9*S*)-9-(*tert*-butyldimethylsilyloxy)-10-hydroxy-7-(triethylsilyloxy)deca-2,4-dien-1-yl-2-(diphenoxyphosphoryl)acetate **29** (0.35 g, 0.50 mmol) in CH_2Cl_2 (5.6 mL), pyridine (0.20 mL, 2.51 mmol) and Dess-Martin Periodinane (0.43 g, 1.00 mmol) were added, and the reaction mixture was stirred at 25 °C for 3 h. Then, a saturated aqueous solution of $NaHCO_3$ and $Na_2S_2O_3$ was added, and the mixture was extracted with Et_2O (3x). The combined organic layers were washed with brine, dried over anhydrous Na_2SO_4, filtered and the solvent was evaporated. The residue was purified by flash column chromatography (C18, from 70:30 to 100:0 (v/v) CH_3CN/H_2O) to afford 0.29 g (83% yield) of a yellow oil, which was identified as (2*E*,4*E*,7*R*,9*S*)-9-(*tert*-butyldimethylsilyloxy)-10-oxo-7-(triethylsilyloxy)deca-2,4-dien-1-yl 2-(diphenoxyphosphoryl)acetate **30**.

$[\alpha]_D^{22}$ = -5.2 (*c* 0.23, CH_2Cl_2).

¹H-NMR (400.16 MHz, C_6D_6): δ 9.45 (d, *J* = 1.5 Hz, 1H, $H_{10'}$), 7.34 (dd, *J* =7.5, 1.2 Hz, 4H, ArH), 6.98 (t, *J* = 7.8 Hz, 4H, ArH), 6.83 (td, *J* = 7.5, 1.0 Hz, 2H, ArH), 6.10 (dd, *J* = 15.0, 10.6 Hz, 1H, $H_{3'}$), 5.93 (dd, *J* = 15.2, 10.4 Hz, $H_{4'}$), 5.63 (dt, *J* = 14.9, 7.3 Hz, 1H, $H_{5'}$), 5.48 (dt, *J* = 15.2, 6.5 Hz, 1H, $H_{2'}$), 4.45 (d, *J* = 6.1 Hz, 2H, $2H_{1'}$), 4.20 – 4.10 (m, 1H, $H_{9'}$), 4.00 – 3.90 (m, 1H, $H_{7'}$), 2.92 (d, $^2J_{P\text{-}H}$ = 21.7 Hz, 2H, $2H_2$), 2.30 – 2.20 (m, 2H, $2H_{6'}$), 1.90 – 1.80 (m, 1H, $H_{8'A}$), 1.80 – 1.68 (m, 1H, $H_{8'B}$), 1.01 (t, *J* = 7.9 Hz, 9H, $3xSiCH_2C\underline{H}_3$), 0.95 (s, 9H, $SiC(CH_3)_3$), 0.62 (q, *J* = 7.9 Hz, 6H, $3xSiC\underline{H}_2CH_3$), 0.08 (s, 3H, Si-CH_3), 0.06 (s, 3H, Si-CH_3) ppm.

¹³C-NMR (100.63 MHz, C_6D_6): δ 201.7 (d), 164.6 (s, $^2J_{P\text{-}C}$ = 6.5 Hz), 150.9 (s, $^2J_{P\text{-}C}$ = 8.1 Hz, 2x), 134.9 (d), 132.4 (d), 131.8 (d), 130.0 (d, 4x), 125.5 (d, 2x), 124.9 (d), 121.2 (d, $^3J_{P\text{-}C}$ = 4.6 Hz, 4x), 76.3 (d),

69.1 (d), 65.9 (t), 41.5 (t), 40.2 (t), 34.2 (t, $^{1}J_{P\text{-}C}$ = 135.8 Hz), 26.0 (q, 3x), 18.4 (s), 7.2 (q, 3x), 5.7 (t, 3x), -4.0 (q), -4.6 (q) ppm.

^{31}P-NMR (162 MHz, C_6D_6): δ 12.7 ppm.

IR (NaCl): υ 2954 (s, C-H), 1720 (s, C=O), 1491 (m, C=C), 1288.22 (s, C-O), 1189 (s, P=O), 1111 (s, Si-O-C), 772 cm^{-1}.

HRMS (ESI$^+$): calcd. for $C_{36}H_{56}O_8PSi_2$ ($[M+H]^+$), 703.3246; found, 703.3247.

(2*Z*,4*S*,6*R*,8*E*,10*E*,15*Z*,17*S*,19*R*,21*E*,23*E*)-4,17-Bis(*tert*-butyldimethylsilyloxy)-6,19-bis(triethylsilyloxy)-13,26-dioxacyclohexacosa-2,8,10,15,21,23-hexaene-1,14-dione 31*Z* and (2*Z*,4*S*,6*R*,8*E*,10*E*,15*E*,17*S*,19*R*,21*E*,23*E*)-4,17-Bis(*tert*-butyldimethylsilyloxy)-6,19-bis(triethylsilyloxy)-13,26-dioxacyclohexacosa-2,8,10,15,21,23-hexaene-1,14-dione 31*E*

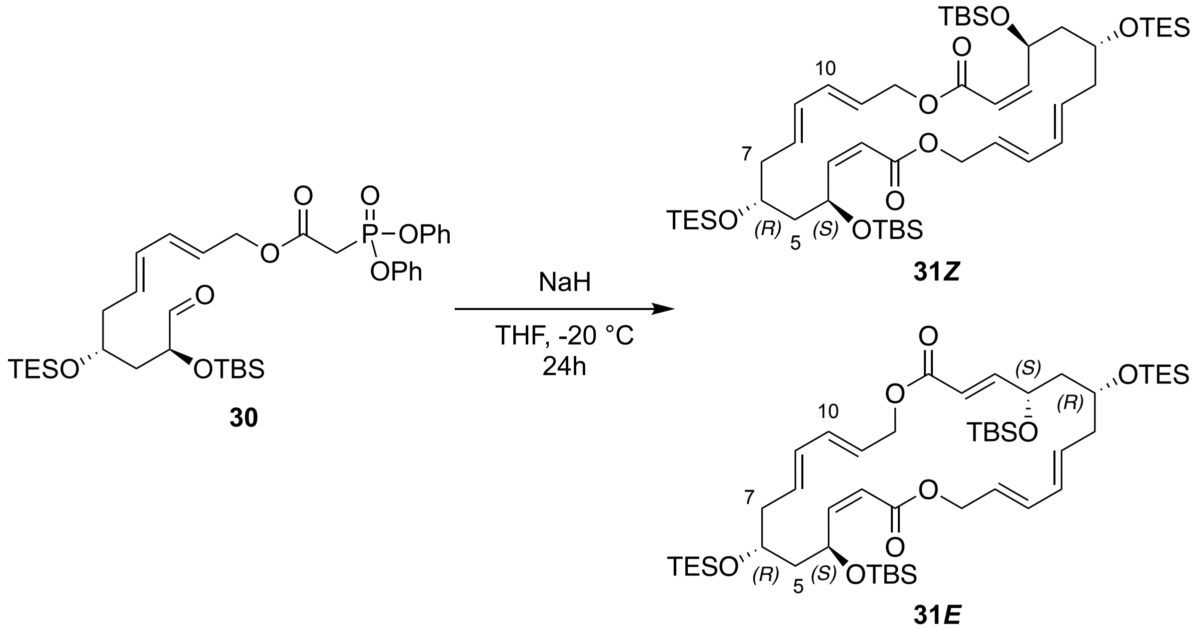

To a cooled (-20 °C) stirred suspension of NaH (0.03 g, 60% in mineral oil, 0.77 mmol) in THF (385 mL), (2*E*,4*E*,7*R*,9*S*)-9-(*tert*-butyldimethylsilyloxy)-10-oxo-7-(triethylsilyloxy)deca-2,4-dien-1-yl 2-(diphenoxyphosphoryl)acetate **30** (0.27 g, 0.38 mmol) was added, and the reaction mixture was stirred at the same temperature for 24h. Then, a buffer solution (pH= 7) was added, and the mixture was extracted with EtOAc (3x). The combined organic layers were washed with brine, dried over anhydrous Na_2SO_4, filtered and the solvent was evaporated. The residue was purified by flash column chromatography (silica gel, from 98:2 (v/v) *n*-hexane/Et_3N to 95:5 (v/v) *n*-hexane/EtOAc) to afford 88 mg (50% yield) of a yellow oil, which was identified as (2*Z*,4*S*,6*R*,8*E*,10*E*,15*Z*,17*S*,19*R*,21*E*,23*E*)-4,17-bis(*tert*-butyldimethylsilyloxy)-6,19-bis(triethylsilyloxy)-13,26-dioxacyclohexacosa-2,8,10,15,21,23-hexaene-1,14-dione **31*Z*** and 7 mg (8% yield) of a yellow oil, which was identified as (2*Z*,4*S*,6*R*,8*E*,10*E*,15*E*,17*S*,19*R*,21*E*,23*E*)-4,17-

bis(*tert*-butyldimethylsilyloxy)-6,19-bis(triethylsilyloxy)-13,26-dioxacyclohexacosa-2,8,10,15,21,23-hexaene-1,14-dione **31*E***.

Data for (2*Z*,4*S*,6*R*,8*E*,10*E*,15*Z*,17*S*,19*R*,21*E*,23*E*)-4,17-bis(*tert*-butyldimethylsilyloxy)-6,19-bis(triethylsilyloxy)-13,26-dioxacyclohexacosa-2,8,10,15,21,23-hexaene-1,14-dione **31*Z***:

[α]D^{24} = +84.5 (*c* 0.18, CH_2Cl_2).

^{1}H-NMR (400.16 MHz, $(CD_3)_2CO$, data for the monomer): δ 6.41 (dd, *J* = 15.2, 10.4 Hz, 1H, H_{10}), 6.20 – 6.15 (m, 2H, H_9 + H_3), 5.92 – 5.79 (m, 2H, H_8 + H_2), 5.78 – 5.65 (m, 1H, H_{11}), 5.41 (app. td, *J* = 9.5, 4.4 Hz, 1H, H_4), 5.01 (dd, *J* = 12.5, 4.4 Hz, 1H, H_{12A}), 4.43 (dd, *J* = 12.5, 8.3 Hz, 1H, H_{12B}), 3.70 – 3.60 (m, 1H, H_6), 2.60 – 2.50 (m, 1H, H_{7A}), 2.17 (app. dt, *J* = 14.5, 7.5 Hz, 1H, H_{7B}), 1.93 – 1.82 (m, 1H, H_{5A}), 1.82 – 1.71 (m, 1H, H_{5B}), 0.96 (t, *J* = 8.2 Hz, 9H, 3xSiCH$_2$C$\underline{H}_3$), 0.91 (s, 9H, $SiC(CH_3)_3$), 0.60 (q, *J* = 8.2 Hz, 6H, 3xSiC$\underline{H}_2$CH$_3$), 0.11 (s, 3H, Si-CH_3), 0.07 (s, 3H, Si-CH_3) ppm ppm.

^{13}C-NMR (100.63 MHz, $(CD_3)_2CO$, data for the monomer): δ 166.2 (s), 151.4 (d), 136.7 (d), 134.5 (d), 132.1 (d), 125.4 (d), 120.0 (d), 69.9 (d), 66.0 (d), 65.6 (t), 47.1 (t), 40.7 (t), 26.2 (q, 3x), 18.6 (s), 7.3 (q, 3x), 5.6 (t, 3x), -4.2 (q), -4.5 (q) ppm.

IR (NaCl): υ 2954 (s, C-H), 1720 (s, C=O), 1411 (m, C=C), 1254 (s, C-O), 1189 (s, P=O), 1074 (s, Si-O-C) cm^{-1}.

HRMS (ESI$^+$): calcd. for $C_{48}H_{88}O_8Si_4Na$ ([M+Na]$^+$), 927.5448; found, 927.5446.

Data for (2*Z*,4*S*,6*R*,8*E*,10*E*,15*E*,17*S*,19*R*,21*E*,23*E*)-4,17-bis(*tert*-butyldimethylsilyloxy)-6,19-bis(triethylsilyloxy)-13,26-dioxacyclohexacosa-2,8,10,15,21,23-hexaene-1,14-dione **31*E***:

[α]D^{24} = +42.9 (*c* 0.65, CH_2Cl_2).

^{1}H-NMR (400.16 MHz, C_6D_6): δ 7.03 (dd, *J* = 15.7, 4.9 Hz, 1H), 6.28 – 5.91 (m, 7H), 5.88 – 5.53 (m, 6H), 4.99 (dd, *J* = 12.5, 6.0 Hz, 1H), 4.57 (dd, *J* = 5.9, 4.0 Hz, 2H), 4.45 – 4.36 (m, 1H), 4.11 (app. p, *J* = 5.5 Hz, 1H), 4.04 (dd, *J* = 12.5, 7.7 Hz, 1H), 3.91 – 3.81 (m, 1H), 2.75 (ddd, *J* = 14.0, 7.1, 3.1 Hz, 1H), 2.36 (dt, *J* = 12.1, 6.0 Hz, 1H), 2.29 – 2.18 (m, 2H), 2.05 – 1.89 (m, 2H), 1.80 (t, *J* = 5.5 Hz, 2H), 1.07 – 1.04 (m, 18H), 1.01 (s, 9H), 0.95 (s, 9H), 0.71 – 0.58 (m, 12H), 0.20 (s, 3H), 0.16 (s, 3H), 0.06 (s, 3H), 0.02 (s, 3H) ppm.

^{13}C-NMR (100.63 MHz, C_6D_6): δ 165.6 (s), 165.5 (s), 152.3 (d), 151.3 (d), 135.8 (d), 134.5 (d), 133.1 (d), 132.4 (d), 132.3 (d), 131.9 (d), 125.2 (d, 2x), 120.5 (d), 118.9 (d), 69.5 (d, 2x), 68.1 (d), 65.8 (d), 64.7 (t), 64.5 (t), 47.1 (t), 44.4 (t), 41.1 (t), 26.1 (q, 3x), 26.1 (q, 3x), 18.4 (s), 18.3 (s), 7.3 (q, 6x), 5.7 (t, 3x), 5.6 (t, 3x), -4.0 (q), -4.5 (q), -4.6 (q), -4.7 (q) ppm.

IR (NaCl): υ 2954 (s, C-H), 2932 (s, C-H), 2877 (m, C-H), 2857 (m, C-H), 1722 (s, C=O), 1462 (w, C=C), 1254 (m, C-O), 1082 (s, Si-O-C) cm^{-1}.

HRMS (ESI$^+$): calcd. for $C_{48}H_{88}O_8Si_4Na$ ([M+Na]$^+$), 927.5448; found, 927.5457.

(*1aS*,1b*R*,2*S*,4*R*,5a*S*,7a*R*,10a*S*,10b*R*,11*S*,13*R*,14a*S*,16a*R*)-Macrodiolide 33

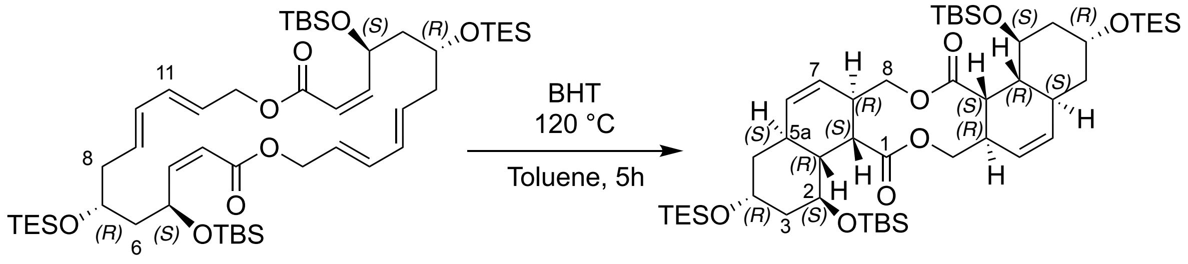

31*Z* **33**

To a solution of (2*Z*,4*S*,6*R*,8*E*,10*E*,15*Z*,17*S*,19*R*,21*E*,23*E*)-4,17-bis(*tert*-butyldimethylsilyloxy)-6,19-bis(triethylsilyloxy)-13,26-dioxacyclohexacosa-2,8,10,15,21,23-hexaene-1,14-dione **31*Z*** (48 mg, 0.05 mmol) in toluene (3.7 mL), one drop to a 0.1 M solution of BHT in toluene was added. The solution mixture was degassed and warmed up to 120 °C for 5h. Then, the solvent was evaporated and the residue was purified by flash column chromatography (silica gel, from 100:0 to 95:10 (v/v) *n*-hexane/EtOAc) to afford 28 mg (58% yield) of a white solid, which was identified as (*1aS*,1b*R*,2*S*,4*R*,5a*S*,7a*R*,10a*S*,10b*R*,11*S*,13*R*,14a*S*,16a*R*)-macrodiolide **33**.

[α]$_D$^{22} = -24.6 (*c* 0.18, CH_2Cl_2).

^{1}H-NMR (400.16 MHz, C_6D_6, data for the monomer): δ 5.60 (ddd, *J* = 9.8, 3.6, 1.9 Hz, 1H, H_7), 5.12 (dt, *J* = 9.8, 2.6 Hz, 1H, H_6), 4.85 (dd, *J* = 10.3, 4.2 Hz, 1H, H_{8A}), 4.49 (app. td, *J* = 10.5, 4.4 Hz, 1H, H_2), 4.02 (app. s, 1H, H_4), 3.38 (dd, *J* = 7.2, 4.2 Hz, 1H, H_{1a}), 3.21 (dd, *J* = 12.2, 10.3 Hz, 1H, H_{8B}), 3.17 (app. t, *J* = 12.6 Hz, 1H, H_{5a}), 2.68 – 2.55 (m, 1H, H_{7a}), 2.18 (app. d, *J* = 12.3 Hz, 1H, H_{3A}), 1.83 (app. d, *J* = 13.2 Hz, 1H, H_{5a}), 1.38 – 1.32 (m, 1H, H_{3B}), 1.27 (td, *J* = 10.6, 7.2 Hz, 1H, H_{1b}), 1.12 (s, 9H, $SiC(CH_3)_3$), 1.06 (t, *J* = 7.9 Hz, 9H, 3x$SiCH_2C\underline{H}_3$), 1.02 – 0.90 (m, 1H, H_{5B}), 0.59 (q, *J* = 8.1 Hz, 6H, 3x$SiC\underline{H}_2CH_3$), 0.23 (s, 3H, Si-CH_3), 0.22 (s, 3H, Si-CH_3) ppm.

^{13}C-NMR (100.63 MHz, C_6D_6): δ 176.5 (s), 136.9 (d), 123.1 (d), 68.5 (d), 67.7 (d), 67.1 (t), 48.6 (d), 44.8 (d), 44.2 (t), 40.5 (t), 39.6 (d), 29.2 (d), 26.5 (q, 3x), 18.5 (s), 7.2 (q, 3x), 5.3 (t, 3x), -2.5 (q), -4.7 (q) ppm.

IR (NaCl): υ 2954 (s, C-H), 2928 (s, C-H), 2877 (s, C-H), 2857 (s, C-H), 1737 (s, C=O), 1462 (m, C=C), 1255 (m, C-O) cm^{-1}.

HRMS (ESI$^+$): calcd. for $C_{48}H_{88}O_8Si_4Na$ ([M+Na]$^+$), 927.5448; found, 927.5485.

(1a*S*,1b*R*,2*S*,4*R*,5a*S*,7a*R*)-2-(*tert*-Butyldimethylsilyloxy)-4-hydroxy-7a-(hydroxymethyl)-1a,1b,2,3,4,5,5a,7a-octahydronaphthalene-1-carboxylic Acid 34

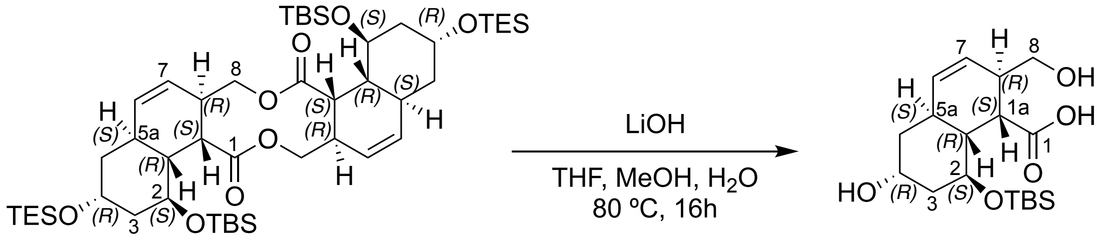

33 **34**

To a stirred solution of (*1aS*,1b*R*,2*S*,4*R*,5a*S*,7a*R*,10a*S*,10b*R*,11*S*,13*R*,14a*S*,16a*R*)-macrodiolide **33** (12 mg, 0.01 mmol) in THF–MeOH-H_2O (0.44 mL, 4:2:1 v/v/v), LiOH (96 mg, 1.94 mmol) was added, and the reaction mixture was stirred at 80 °C for 16h. Then, the reaction mixture was diluted with water and CH_2Cl_2. A 10% aqueous solution of citric acid was added until pH= 5 and the mixture was extracted with CH_2Cl_2 (3x). The combined organic layers were dried over anhydrous Na_2SO_4, filtered and the solvent was evaporated. A 10% aqueous solution of K_2CO_3 was added and the mixture was washed with Et_2O (3x). Then, A 10% aqueous solution of citric acid was added until pH= 5 and the mixture was extracted with CH_2Cl_2 (3x). The combined organic layers were dried over anhydrous Na_2SO_4, filtered and the solvent was evaporated to afford 4 mg (85% yield) of a white solid, which was identified as (1a*S*,1b*R*,2*S*,4*R*,5a*S*,7a*R*)-2-(*tert*-butyldimethylsilyloxy)-4-hydroxy-7a-(hydroxymethyl)-1a,1b,2,3,4,5,5a,7a-octahydronaphthalene-1-carboxylic acid **34**.

$[\alpha]_D$^{24} = +4.4 (*c* 0.20, $CHCl_3$).

^{1}H-NMR (400.16 MHz, CD_3OD): δ 5.61 – 5.54 (m, 1H, H_7), 5.54 (app. dt, *J* = 10.0, 1.4 Hz, 1H, H_6), 4.36 (app. td, *J* = 10.5, 4.6 Hz, 1H, H_2), 4.14 (app. p, *J* = 3.1 Hz, 1H, H_4), 3.56 (dd, *J* = 10.6, 5.6 Hz, 1H, H_{8A}), 3.37 (dd, *J* = 10.6, 8.9 Hz, 1H, H_{8B}), 3.26 (app. d, *J* = 3.5 Hz, 1H, H_{1a}), 2.76 (app. t, *J* = 12.5 Hz, 1H, H_{5a}), 2.73 – 2.66 (m, 1H, H_{7a}), 2.20 – 2.11 (m, 1H, H_{3A}), 1.82 (app. dq, *J* = 13.7, 3.0 Hz, 1H, H_{5A}), 1.40 (ddd, *J* = 13.4, 10.8, 2.9 Hz, 1H, H_{3B}), 1.31 (td, *J* = 10.6, 3.6 Hz, 1H, H_{1b}), 1.18 (app. td, *J* = 13.3, 2.7 Hz, 1H, H_{5B}), 0.91 (s, 9H, $SiC(CH_3)_3$), 0.11 (s, 3H, Si-CH_3), 0.10 (s, 3H, Si-CH_3).

^{13}C-NMR (100.63 MHz, CD_3OD): δ 177.7 (s), 134.6 (d), 126.5 (d), 68.6 (d), 67.9 (d), 65.6 (t), 46.8 (d), 44.4 (t), 42.8 (d), 40.2 (t), 39.3 (d), 31.0 (d), 26.6 (q, 3x), 19.0 (s), -3.7 (q), -4.6 (q) ppm.

IR (NaCl): υ 3602 – 3138 (br, O-H), 2953 (s, C-H), 2926 (s, C-H), 2855 (s, C-H), 1716 (s, C=O), 1254 (s, C-O), 1086 (m, Si-O-C) cm^{-1}.

HMRS (ESI^+): calcd. for $C_{18}H_{33}O_5Si$ ($[M+H]^+$), 357.2092; found, 357.2093.

(1a*S*,1b*R*,2*S*,4*R*,5a*S*,7a*R*)-2,4-Dihydroxy-7a-(hydroxymethyl)-1a,1b,2,3,4,5,5a,7a-octahydronaphthalene-1-carboxylic Acid 35

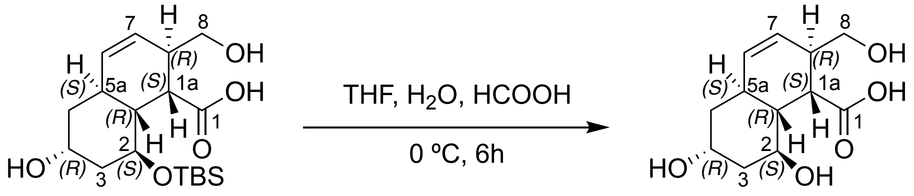

34 **35**

To a cooled (0 °C) solution of (1a*S*,1b*R*,2*S*,4*R*,5a*S*,7a*R*)-2-(*tert*-butyldimethylsilyloxy)-4-hydroxy-7a-(hydroxymethyl)-1a,1b,2,3,4,5,5a,7a-octahydronaphthalene-1-carboxylic acid **34** (8 mg, 0.02 mmol) in THF (0.57 mL), a cooled (0 °C) solution of HCOOH (0.10 mL) and H_2O (0.29 mL) was added and the mixture was stirred at the same temperature for 6h. Then, the solvent was evaporated and the residue was triturated with 90:10 (v/v) *n*-hexane/Et_2O to afford 4 mg (81% yield) of a white solid, which was identified as (1a*S*,1b*R*,2*S*,4*R*,5a*S*,7a*R*)-2,4-dihydroxy-7a-(hydroxymethyl)-1a,1b,2,3,4,5,5a,7a-octahydronaphthalene-1-carboxylic acid **35**.

$[\alpha]_D$ ^{21} = +46.4 (*c* 0.20, MeOH).

^{1}H-NMR (400.16 MHz, CD_3OD): δ 5.64 – 5.51 (m, 2H, H_6 + H_7), 4.16 (br s, 2H, H_2 + H_4), 3.56 (dd, *J* = 10.8, 5.0 Hz, 1H, H_{8A}), 3.38 (dd, *J* = 10.8, 8.9 Hz, 1H, H_{8B}), 3.26 (br s, 1H, H_{1a}), 2.66 (br s, 1H, H_{7a}), 2.59 (app. t, *J* = 11.3 Hz, 1H, H_{5a}), 2.16 (app. d, *J* = 13.2 Hz, 1H, H_{3A}), 1.83 (app. dd, *J* = 13.7, 2.9 Hz, 1H, H_{5A}), 1.43 (app. t, *J* = 11.2 Hz, 1H, H_{3B}), 1.35 – 1.29 (m, 1H, H_{1b}), 1.19 (app. td, *J* = 13.3, 2.6 Hz, 1H, H_{5B}) ppm.

^{13}C-NMR (100.63 MHz, CD_3OD): δ 177.9 (s), 134.2 (d), 126.7 (d), 67.7 (d), 67.0 (d), 65.5 (t), 46.4 (d), 43.3 (t), 42.6 (d), 40.1 (t and d, 2x), 31.1 (d) ppm.

IR (NaCl): υ 3614 – 3103 (br, O-H), 2925 (s, C-H), 2854 (m, C-H), 1724 (m, C=O), 1259 (m, C-O) cm^{-1}.

HMRS (ESI^+): calcd. for $C_{12}H_{18}O_5Na$ ($[M+Na]^+$), 265.1046; found, 265.1048.

8. X-Ray structure of compound 33

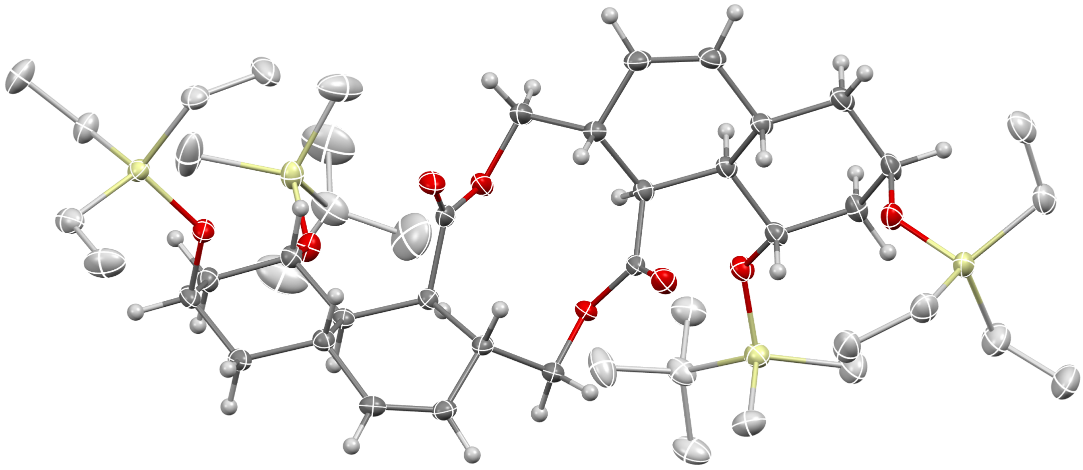

FIGURE S2. X-ray crystallographic structure of **33** (Displacement ellipsoids are drawn at the 50% probability level).

Crystals were obtained by precipitation of a solution of **33** in MeCN/Et_2O 9:1 *v/v*.

TABLE 1. Crystal data and structure refinement for **33**.

Empirical formula	C48 H88 O8 Si4	
Formula weight	905.54	
Temperature	100.00 K	
Wavelength	0.71073 Å	
Crystal system	Orthorhombic	
Space group	P212121	
Unit cell dimensions	a = 14.3694(6) Å	α = 90°
	b = 14.5349(6) Å	β = 90°
	c = 26.1503(12) Å	γ = 90°
Volume	5461.7(4) $Å^3$	
Z	4	
Density (calculated)	1.101 Mg/m^3	
Absorption coefficient	0.154 mm^{-1}	
F(000)	1984	
Crystal size	0.212 x 0.191 x 0.079 mm^3	
Theta range for data collection	2.095 to 28.311°.	
Index ranges	-19≤h≤18, -19≤k≤16, -34≤l≤29	
Reflections collected	90518	

Independent reflections	13574 [R(int) = 0.0375]
Completeness to theta = 25.242°	99.9 %
Absorption correction	Semi-empirical from equivalents
Max. and min. transmission	0.7457 and 0.6913
Refinement method	Full-matrix least-squares on F^2
Data / restraints / parameters	13574 / 0 / 568
Goodness-of-fit on F^2	1.125
Final R indices [I>2sigma(I)]	R1 = 0.0397, wR2 = 0.0879
R indices (all data)	R1 = 0.0433, wR2 = 0.0896
Absolute structure parameter	-0.015(19)
Extinction coefficient	n/a
Largest diff. peak and hole	0.312 and -0.227 e.$Å^{-3}$

9. DFT computational methods and computations on the octahydronaphthalene diastereomers

DFT-based structural optimization was carried out using the Gaussian 16 suite of programs[21] at the B3LYP/6-31+G**-PCM(MeOH) level. In the case of conformationally flexible compounds, the conformational search was done using CREST 3.0[22] and with methanol as solvent. All conformers within 5 kcal/mol of the lowest energy conformer were subjected to further optimization at the same level of theory.[23–25] The conformations within 2 kcal/mol from the B3LYP/6-31+G**-PCM(MeOH) global minima were subjected to NMR calculations using the gauge including atomic orbitals (GIAO).[26,27] The unscaled chemical shifts (δ_u) were computed using TMS as reference standard according to $\delta_u = \sigma_{TMS} - \sigma_x$, where σ_x is the Boltzmann averaged shielding tensor (over all significantly populated conformations) and σ_{TMS} is the shielding tensor of TMS computed at the same level of theory employed for σ_x. Scaled chemical shifts were calculated following the statistical equation δ_s = (δ_u-intercept)/slope). sDP4+, uDP4+ and DP4+ probabilities (%) were calculated using the tool develop by Sarotti.[28]

The corrected mean absolute error (CMAE) computed for proton and carbon are shown in Figure S2. Being the hydroxyl group placed at the axial position, H15 on sagamilactam model **M1** (**3*R***) is deshielded when compared to epimer **M2** (**3*S***). The same trend was exhibited by the signals on the natural product sagamilactam **1** (Figure S1). Moreover, a similar chemical shift value for both carbinol hydrogens was experimentally observed (and computed) for synthetic compound **35**, which is likely reflecting in this case the effect of the axial carboxylic acid in its proximity.

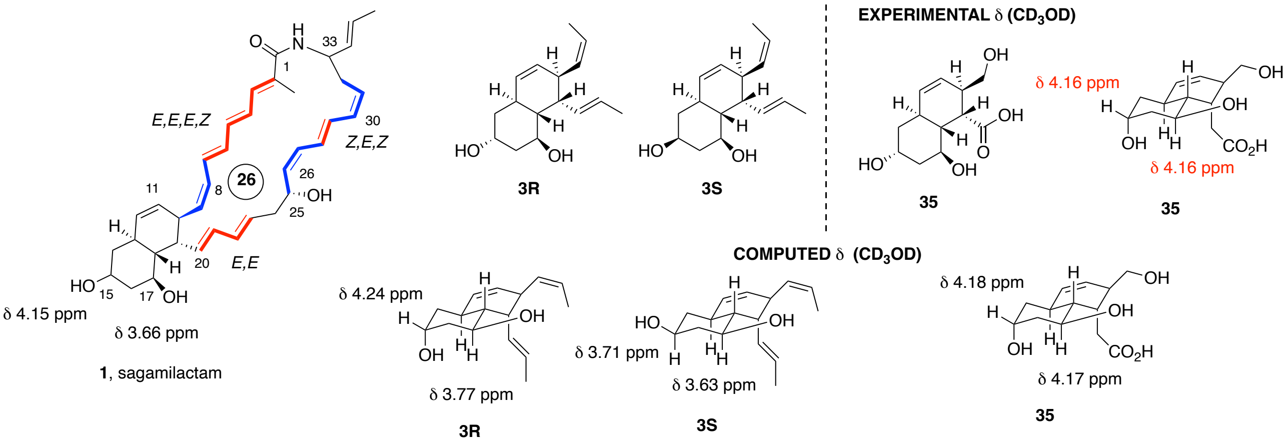

Figure S2. Selected experimental (**1** and **35**) and calculated ^{1}H-NMR chemical shifts (**3*R***, **3*S*** and **35**) to confirm the structural assignment of the relative configuration at C15 of sagamilactam (**1**).

Scaled chemical shifts (δ_s) calculated for the B3LYP/6-31+G-PCM(MeOH) computed geometries of 3*R* and 3*S* models and comparison with the reported NMR data.**

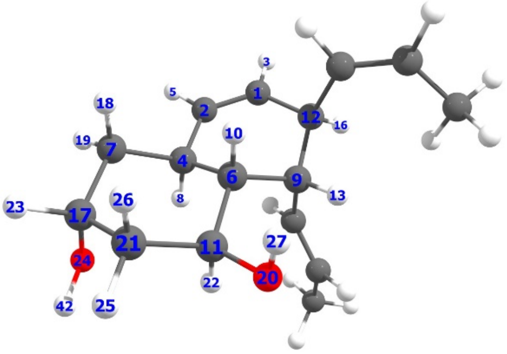

		**3*R***
**1**	C	131.22
**2**	C	132.38
**3**	H	5.54
**4**	C	28.84
**5**	H	5.49
**6**	C	49.09
**7**	C	40.15
**8**	H	2.56
**9**	C	43.31
**10**	H	1.23
**11**	C	68.39
**12**	C	39.82
**13**	H	2.70
**15**	C	135.02
**16**	H	3.04
**17**	C	70.18
**18**	H	1.35
**19**	H	1.72
**21**	C	43.69
**22**	H	3.78
**23**	H	4.24
**25**	H	2.05
**26**	H	1.39

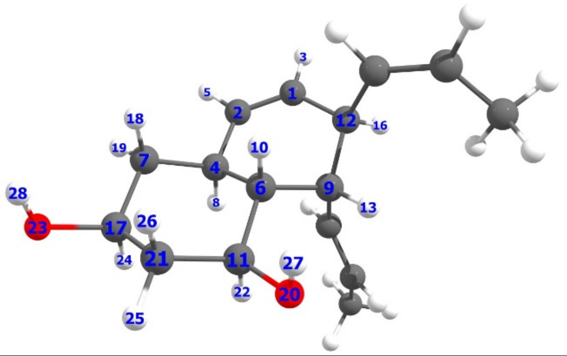

		3S
**1**	C	129.99
**2**	C	130.39
**3**	H	5.69
**4**	C	29.66
**5**	H	5.65
**6**	C	46.21
**7**	C	41.77
**8**	H	2.11
**9**	C	41.70
**10**	H	1.35
**11**	C	69.20
**12**	C	38.46
**13**	H	2.84
**15**	C	133.39
**16**	H	3.18
**17**	C	70.16
**18**	H	1.12
**19**	H	2.12
**21**	C	46.44
**22**	H	3.64
**24**	H	3.73
**25**	H	2.35
**26**	H	1.28

sDP4+, uDP4+ and DP4+ probabilities (%) for 3R and 3S models computed at the B3LYP/6-31+G-PCM level.**

Parameters	3*R*	3*S*
sDP4+ (H data)	99.98%	0.02%
sDP4+ (C data)	99.86%	0.14%
sDP4+ (all data)	100.00%	0.00%
uDP4+ (H data)	37.56%	62.44%
uDP4+ (C data)	86.86%	13.14%
uDP4+ (all data)	79.91%	20.09%
DP4+ (H data)	99.97%	0.03%
DP4+ (C data)	99.98%	0.02%
DP4+ (all data)	100.00%	0.00%

Z-matrix for B3LYP/6-31+G**-PCM(MeOH) geometries of 3*R* and 3*S* models

3R_conformer 1

Num. Imaginary Frequencies: 0

C	-1.068526	-1.366614	-1.888779
C	0.251982	-1.395740	-1.968301
H	-1.672106	-1.873598	-2.630747
C	1.155119	-0.723076	-0.979794
H	0.741295	-1.904913	-2.788154
C	0.392927	-0.427763	0.312141
C	2.404324	-1.553325	-0.678745
H	1.505572	0.224518	-1.408201
C	-0.934635	0.283941	0.001093
H	0.146751	-1.381083	0.797158
C	1.285461	0.373490	1.267571
C	-1.838543	-0.686463	-0.792934
H	-1.418581	0.530270	0.951559
C	-0.734714	1.553301	-0.770958
C	-2.453303	-1.722235	0.116011
H	-2.644024	-0.100422	-1.250537
C	3.318608	-0.792030	0.283864
H	2.127471	-2.509907	-0.232467
H	2.952628	-1.744840	-1.602439
O	0.621843	0.727725	2.463753
C	2.560995	-0.426605	1.565036
H	1.546583	1.329648	0.801185
H	4.171440	-1.440104	0.553053
O	3.805238	0.350041	-0.394991
H	3.202121	0.157552	2.230256
H	2.291532	-1.350175	2.083265
H	0.348149	-0.083565	2.912184
C	-0.844856	2.764350	-0.250032
H	-0.501904	1.434260	-1.821402
C	-0.653728	4.037821	-1.005916
H	-1.078938	2.876391	0.801715
H	0.156033	4.618573	-0.564318
H	-0.417305	3.840591	-2.048892
H	-1.557948	4.644435	-0.956878
C	-3.521226	-1.552229	0.881820
H	-1.945287	-2.678321	0.130985
H	-3.854998	-2.378510	1.498654
C	-4.347358	-0.314817	1.009853
H	-4.280349	0.068480	2.028201
H	-4.023624	0.464900	0.327323
H	-5.394403	-0.546581	0.815553
H	4.348067	0.868235	0.211275

3R_conformer 2

Num. Imaginary Frequencies: 0

C	-1.077678	-1.349908	-1.902259
C	0.242674	-1.374176	-1.984016
H	-1.679411	-1.858484	-2.644361
C	1.144992	-0.698984	-0.992923
H	0.733753	-1.879945	-2.804653
C	0.380232	-0.422275	0.301532
C	2.396260	-1.530720	-0.701619
H	1.465774	0.258258	-1.426629
C	-0.947517	0.292151	-0.005743
H	0.133703	-1.383043	0.769761
C	1.265836	0.362716	1.278014
C	-1.849604	-0.675005	-0.804568
H	-1.429430	0.533178	0.946764
C	-0.744844	1.565733	-0.771473
C	-2.464368	-1.716252	0.098491
H	-2.655673	-0.088433	-1.260342
C	3.317640	-0.818892	0.299248
H	2.106417	-2.498053	-0.287525
H	2.952111	-1.707330	-1.624810
O	0.591386	0.682797	2.477686
C	2.545038	-0.435047	1.568606
H	1.511809	1.339456	0.845260
H	4.133861	-1.497196	0.577642
O	3.984168	0.285463	-0.277533
H	3.194845	0.151565	2.221601
H	2.272917	-1.352842	2.095524
H	0.355600	-0.140148	2.926152
C	-0.793590	2.772809	-0.231713
H	-0.561880	1.453833	-1.832929
C	-0.594250	4.049491	-0.979891
H	-0.980128	2.877618	0.830193
H	-0.407125	3.860012	-2.034382
H	-1.477368	4.681277	-0.886461
H	0.248708	4.601659	-0.564306
C	-3.528347	-1.547568	0.869963
H	-1.960569	-2.674616	0.103779
H	-3.862561	-2.377012	1.482185
C	-4.348031	-0.307458	1.011591
H	-4.263018	0.075301	2.028841
H	-4.033262	0.471746	0.324194
H	-5.398829	-0.535763	0.834737
H	3.328661	0.943195	-0.540899

3R_conformer 3

Num. Imaginary Frequencies: 0

C	-1.070520	-1.339250	-1.907212
C	0.250312	-1.373140	-1.978396
H	-1.671092	-1.834739	-2.659260
C	1.149925	-0.718273	-0.974186
H	0.741610	-1.875860	-2.801300
C	0.380543	-0.426922	0.313808
C	2.385974	-1.564565	-0.662954
H	1.506064	0.235662	-1.385668
C	-0.940823	0.293672	-0.003771
H	0.125245	-1.380874	0.792706
C	1.271348	0.363965	1.280470
C	-1.843839	-0.668847	-0.807931
H	-1.428082	0.539342	0.944940
C	-0.726607	1.564405	-0.770097

C	-2.462050	-1.712970	0.089154
H	-2.647766	-0.078325	-1.262484
C	3.303169	-0.809857	0.307125
H	2.088911	-2.511452	-0.208492
H	2.928252	-1.788008	-1.585174
O	0.596969	0.707537	2.473575
C	2.543808	-0.438453	1.581834
H	1.545430	1.321932	0.826880
H	4.149253	-1.462849	0.585016
O	3.809000	0.383127	-0.261325
H	3.194280	0.156031	2.225494
H	2.276015	-1.356352	2.110434
H	0.342181	-0.108540	2.924833
C	-0.794635	2.773426	-0.237618
H	-0.518157	1.448734	-1.826204
C	-0.583648	4.047647	-0.986761
H	-1.003666	2.882126	0.819641
H	-0.385893	3.854141	-2.038455
H	-1.464098	4.684564	-0.903547
H	0.259182	4.594663	-0.564266
C	-3.527175	-1.546728	0.859557
H	-1.959301	-2.671964	0.091350
H	-3.863813	-2.378745	1.467031
C	-4.345846	-0.306404	1.005207
H	-4.273054	0.064102	2.027892
H	-4.019932	0.479838	0.331235
H	-5.394852	-0.529906	0.811840
H	4.285911	0.164905	-1.071815

3R_conformer 4

Num. Imaginary Frequencies: 0

C	-1.364322	-1.268608	-1.554526
C	-0.075566	-1.471715	-1.774505
H	-2.109089	-1.746230	-2.177873
C	1.012227	-0.851857	-0.951418
H	0.247873	-2.098323	-2.595145
C	0.455556	-0.403689	0.400315
C	2.192534	-1.804023	-0.748656
H	1.404498	0.025018	-1.481439
C	-0.812299	0.446397	0.207172
H	0.175305	-1.298634	0.969668
C	1.545077	0.333577	1.189516
C	-1.916848	-0.409560	-0.450679
H	-1.156355	0.776693	1.193610
C	-0.549431	1.662371	-0.630920
C	-2.601186	-1.292214	0.557696
H	-2.653581	0.272102	-0.886499
C	3.298339	-1.108260	0.047999
H	1.871625	-2.699350	-0.213526
H	2.596142	-2.102880	-1.717288
O	1.081812	0.825479	2.430550
C	2.751586	-0.594664	1.384399
H	1.850165	1.226700	0.633620
H	4.101838	-1.837400	0.254090
O	3.817914	-0.065300	-0.754480
H	3.530769	-0.056977	1.930760
H	2.449744	-1.454082	1.988167
H	0.786201	0.076456	2.965121
C	-0.455607	2.891594	-0.151359
H	-0.448168	1.486289	-1.694247
C	-0.196316	4.109826	-0.974964
H	-0.557954	3.061012	0.913634
H	-1.010264	4.824879	-0.856496
H	0.720565	4.597765	-0.644543
H	-0.100145	3.855682	-2.027906
C	-3.882733	-1.290012	0.894373
H	-1.946474	-2.010286	1.035405
H	-4.216434	-2.002167	1.640838
C	-4.972645	-0.414540	0.368990
H	-5.458373	0.102772	1.195982
H	-4.607582	0.325275	-0.336617
H	-5.727963	-1.025700	-0.125489
H	4.489646	0.412207	-0.252973

3R_conformer 5

Num. Imaginary Frequencies: 0

C	-1.425848	-1.231630	-1.500689
C	-0.146734	-1.458992	-1.750455
H	-2.192942	-1.706808	-2.097840
C	0.971592	-0.845370	-0.959832
H	0.146862	-2.103193	-2.568359
C	0.450096	-0.394750	0.405014
C	2.142172	-1.816436	-0.788707
H	1.334678	0.033614	-1.509369
C	-0.802287	0.484359	0.236489
H	0.159369	-1.288846	0.969351
C	1.564455	0.310983	1.190129
C	-1.938151	-0.345777	-0.398162
H	-1.116121	0.822609	1.230017
C	-0.527326	1.693696	-0.608732
C	-2.637048	-1.197995	0.626380
H	-2.661070	0.351794	-0.832182
C	3.283628	-1.180804	0.017343
H	1.801872	-2.714226	-0.269534
H	2.527005	-2.113352	-1.766511
O	1.123003	0.781777	2.447283
C	2.762064	-0.635225	1.353499
H	1.864719	1.221512	0.658749
H	4.043504	-1.944958	0.223444
O	3.985432	-0.204104	-0.724076
H	3.569080	-0.108274	1.867104
H	2.459160	-1.481478	1.974986
H	0.868096	0.022518	2.988087
C	-0.335740	2.911459	-0.128511
H	-0.503530	1.523671	-1.677987
C	-0.062754	4.121282	-0.959912
H	-0.363638	3.075899	0.941665
H	-0.829083	4.876426	-0.786420
H	0.896532	4.559069	-0.684060
H	-0.046619	3.873162	-2.018625

C	-3.933911	-1.232093	0.895785
H	-1.980936	-1.859097	1.178283
H	-4.278666	-1.913908	1.665030
C	-5.028125	-0.438137	0.261091
H	-5.714021	-1.108763	-0.256949
H	-5.597079	0.083990	1.029795
H	-4.653796	0.291044	-0.450755
H	3.382656	0.518801	-0.937953

3R_conformer 6

Num. Imaginary Frequencies: 0

C	-1.515127	-1.148134	-1.419757
C	-0.256143	-1.442370	-1.700416
H	-2.321595	-1.584303	-1.994383
C	0.911640	-0.886368	-0.943978
H	-0.018779	-2.103619	-2.523222
C	0.454184	-0.391933	0.428536
C	2.036580	-1.911946	-0.791448
H	1.331246	-0.041188	-1.503541
C	-0.758828	0.544597	0.286905
H	0.139730	-1.263306	1.016891
C	1.627766	0.272445	1.159675
C	-1.951112	-0.232588	-0.307709
H	-1.029265	0.907123	1.284938
C	-0.449731	1.732409	-0.575975
C	-2.662323	-1.046558	0.739955
H	-2.652424	0.497897	-0.724263
C	3.223701	-1.285459	-0.056858
H	1.685548	-2.781456	-0.233117
H	2.370711	-2.241841	-1.776322
O	1.258988	0.796944	2.419043
C	2.778831	-0.732458	1.301369
H	1.963392	1.142113	0.584595
H	3.988120	-2.064009	0.114125
O	3.767903	-0.281455	-0.892028
H	3.617553	-0.244951	1.805020
H	2.451904	-1.568118	1.925116
H	0.943874	0.070019	2.972599
C	-0.236389	2.953625	-0.114581
H	-0.419136	1.542968	-1.641423
C	0.070846	4.143101	-0.963332
H	-0.268232	3.137170	0.952560
H	-0.676537	4.920759	-0.806808
H	1.040376	4.559008	-0.689378
H	0.087455	3.876329	-2.017455
C	-3.970699	-1.158509	0.916631
H	-2.006613	-1.609877	1.391310
H	-4.327741	-1.798029	1.715968
C	-5.061325	-0.506477	0.131718
H	-5.651940	-1.268629	-0.377106
H	-5.728907	0.032226	0.803738
H	-4.678683	0.185871	-0.611870
H	4.493575	0.153681	-0.428515

3R_conformer 7

Num. Imaginary Frequencies: 0

C	-1.362777	-1.264395	-1.558467
C	-0.073334	-1.467351	-1.774017
H	-2.105046	-1.740935	-2.185586
C	1.011497	-0.851245	-0.943390
H	0.251671	-2.093960	-2.594275
C	0.450327	-0.401741	0.405518
C	2.185756	-1.808331	-0.728181
H	1.403688	0.031535	-1.465276
C	-0.816069	0.449197	0.205511
H	0.167012	-1.294937	0.976012
C	1.537694	0.336238	1.198482
C	-1.918717	-0.406313	-0.455976
H	-1.162165	0.781061	1.190527
C	-0.548371	1.663642	-0.633569
C	-2.607584	-1.288697	0.549561
H	-2.653648	0.275845	-0.894011
C	3.293994	-1.107723	0.066915
H	1.855406	-2.690587	-0.176826
H	2.577623	-2.140703	-1.692740
O	1.064903	0.821254	2.438484
C	2.748093	-0.585210	1.396575
H	1.849238	1.229792	0.648098
H	4.097138	-1.834302	0.282753
O	3.841770	-0.011230	-0.639826
H	3.533818	-0.031802	1.913483
H	2.458015	-1.437429	2.015781
H	0.798500	0.066005	2.979911
C	-0.424879	2.889380	-0.151998
H	-0.469549	1.490079	-1.699392
C	-0.157885	4.105658	-0.975837
H	-0.503989	3.056017	0.915310
H	0.782983	4.564154	-0.671312
H	-0.102206	3.856896	-2.033038
H	-0.946573	4.842801	-0.826430
C	-3.889753	-1.282238	0.883835
H	-1.955890	-2.008658	1.028541
H	-4.227309	-1.993646	1.629240
C	-4.975531	-0.402003	0.357798
H	-4.607232	0.335630	-0.348395
H	-5.734014	-1.009944	-0.135827
H	-5.458422	0.118459	1.184459
H	4.182797	-0.324769	-1.486814

3R_conformer 8

Num. Imaginary Frequencies: 0

C	-1.475624	-1.171792	-1.463008
C	-0.207056	-1.441320	-1.724668
H	-2.264074	-1.616010	-2.056286
C	0.937899	-0.874647	-0.940857
H	0.054471	-2.091129	-2.549528
C	0.449473	-0.394769	0.425648
C	2.072098	-1.886688	-0.766220
H	1.351407	-0.013225	-1.481464
C	-0.776973	0.521229	0.266322
H	0.140425	-1.272663	1.006847

C	1.599774	0.286466	1.179432
C	-1.946011	-0.274557	-0.350722
H	-1.068486	0.875718	1.261112
C	-0.473736	1.716051	-0.589193
C	-2.654748	-1.109647	0.681354
H	-2.655185	0.445139	-0.771938
C	3.240855	-1.239326	-0.013813
H	1.720275	-2.751607	-0.200866
H	2.411857	-2.238159	-1.743687
O	1.195387	0.792303	2.435329
C	2.769411	-0.693754	1.334848
H	1.935591	1.164325	0.618249
H	4.016890	-2.002627	0.172213
O	3.812152	-0.168517	-0.741076
H	3.599091	-0.180665	1.823909
H	2.459862	-1.532114	1.963505
H	0.911834	0.050207	2.986136
C	-0.254352	2.933006	-0.119722
H	-0.451802	1.535656	-1.656583
C	0.050067	4.128364	-0.961024
H	-0.276649	3.107045	0.949102
H	1.028935	4.530177	-0.699297
H	0.045632	3.873259	-2.018159
H	-0.685763	4.912262	-0.782744
C	-3.959857	-1.183383	0.897841
H	-1.999197	-1.723070	1.286156
H	-4.312475	-1.845481	1.680674
C	-5.053309	-0.459708	0.182780
H	-4.673676	0.248196	-0.547564
H	-5.693053	-1.177857	-0.330642
H	-5.672132	0.076248	0.901976
H	4.108440	-0.497074	-1.599118

3R_conformer 9

Num. Imaginary Frequencies: 0

C	-1.045713	-1.426336	-1.848996
C	0.274711	-1.446041	-1.927397
H	-1.644309	-1.956343	-2.578859
C	1.170928	-0.742202	-0.954752
H	0.767970	-1.971232	-2.735079
C	0.409619	-0.411948	0.328192
C	2.420790	-1.558717	-0.620679
H	1.511755	0.199649	-1.405965
C	-0.931374	0.268456	0.007115
H	0.180784	-1.340492	0.862525
C	1.295568	0.436306	1.244356
C	-1.821414	-0.730158	-0.768508
H	-1.411165	0.512915	0.959186
C	-0.760164	1.527954	-0.786395
C	-2.424246	-1.753604	0.161362
H	-2.633646	-0.163181	-1.238279
C	3.336946	-0.751669	0.305891
H	2.139233	-2.487194	-0.121090
H	2.957198	-1.815941	-1.537696
O	0.572346	0.724159	2.427182
C	2.586997	-0.326162	1.568908
H	1.550729	1.374191	0.726802
H	4.192399	-1.382203	0.605904
O	3.826503	0.420429	-0.319528
H	3.246086	0.308145	2.166129
H	2.333330	-1.212793	2.152642
H	1.130237	1.254519	3.008868
C	-0.939601	2.745204	-0.300326
H	-0.491344	1.396028	-1.826907
C	-0.781371	4.008494	-1.080999
H	-1.215398	2.869778	0.739830
H	-0.508289	3.797695	-2.112227
H	-1.711551	4.576418	-1.070923
H	-0.009303	4.633900	-0.632836
C	-3.503707	-1.586135	0.911022
H	-1.894872	-2.696750	0.209888
H	-3.824992	-2.402101	1.547770
C	-4.355668	-0.362940	1.000150
H	-5.398908	-0.622150	0.820826
H	-4.288734	0.056045	2.004346
H	-4.050766	0.399400	0.289896
H	4.288958	0.171968	-1.129637

3R_conformer 10

Num. Imaginary Frequencies: 0

C	1.169514	0.341724	2.047469
C	-0.144544	0.413832	2.179069
H	1.814726	0.497977	2.902541
C	-1.097280	0.203536	1.039217
H	-0.591057	0.655570	3.134127
C	-0.407579	-0.581424	-0.077162
C	-2.365826	-0.520163	1.498637
H	-1.375668	1.193698	0.651491
C	0.928364	0.090019	-0.451027
H	-0.173089	-1.584223	0.300293
C	-1.356965	-0.740962	-1.273332
C	1.875584	0.010433	0.764852
H	1.370553	-0.490958	-1.268552
C	0.723231	1.486201	-0.968733
C	2.484047	-1.367312	0.874541
H	2.683125	0.735115	0.614653
C	-3.340446	-0.752206	0.336330
H	-2.097831	-1.486660	1.929420
H	-2.871606	0.066967	2.267965
O	-0.748182	-1.411997	-2.357731
C	-2.633370	-1.464717	-0.824727
H	-1.610951	0.244557	-1.679243
H	-4.167496	-1.384002	0.683647
O	-3.979939	0.439036	-0.073875
H	-3.319157	-1.542739	-1.671119
H	-2.373036	-2.475558	-0.502004
H	-0.525270	-2.309481	-2.077553
C	0.880373	2.616182	-0.298398
H	0.413863	1.530284	-2.006639
C	0.656730	3.975077	-0.877243

H	1.185243	2.603941	0.739509
H	0.341456	3.911979	-1.916260
H	-0.106302	4.505594	-0.307575
H	1.573089	4.562669	-0.823179
C	3.531909	-1.813662	0.197466
H	1.989618	-2.033056	1.570753
H	3.861857	-2.832476	0.363956
C	4.338775	-1.062387	-0.809804
H	5.393248	-1.092122	-0.536167
H	4.240798	-1.536046	-1.786815
H	4.023860	-0.026993	-0.896747
H	-3.310104	1.064627	-0.376590

TMS

Num. Imaginary Frequencies: 0

Si	0.000052	0.000353	-0.000012
C	-1.408936	1.257563	-0.107862
C	1.657021	0.890297	-0.200322
C	-0.052633	-0.862982	1.681940
C	-0.195476	-1.285175	-1.373774
H	-2.385378	0.776023	0.011584
H	-1.323675	2.023225	0.670658
H	-1.413990	1.772604	-1.074438
H	2.497657	0.191124	-0.136614
H	1.725687	1.397447	-1.168639
H	1.801240	1.648018	0.577239
H	-1.147756	-1.819807	-1.290313
H	-0.166690	-0.820367	-2.365100
H	0.604180	-2.032804	-1.339370
H	-0.999672	-1.392675	1.830658
H	0.752840	-1.598581	1.780520
H	0.054968	-0.147372	2.504082

3R_conformer 1

Num. Imaginary Frequencies: 0

C	-1.068526	-1.366614	-1.888779
C	0.251982	-1.395740	-1.968301
H	-1.672106	-1.873598	-2.630747
C	1.155119	-0.723076	-0.979794
H	0.741295	-1.904913	-2.788154
C	0.392927	-0.427763	0.312141
C	2.404324	-1.553325	-0.678745
H	1.505572	0.224518	-1.408201
C	-0.934635	0.283941	0.001093
H	0.146751	-1.381083	0.797158
C	1.285461	0.373490	1.267571
C	-1.838543	-0.686463	-0.792934
H	-1.418581	0.530270	0.951559
C	-0.734714	1.553301	-0.770958
C	-2.453303	-1.722235	0.116011
H	-2.644024	-0.100422	-1.250537
C	3.318608	-0.792030	0.283864
H	2.127471	-2.509907	-0.232467
H	2.952628	-1.744840	-1.602439
O	0.621843	0.727725	2.463753
C	2.560995	-0.426605	1.565036
H	1.546583	1.329648	0.801185
H	4.171440	-1.440104	0.553053
O	3.805238	0.350041	-0.394991
H	3.202121	0.157552	2.230256
H	2.291532	-1.350175	2.083265
H	0.348149	-0.083565	2.912184
C	-0.844856	2.764350	-0.250032
H	-0.501904	1.434260	-1.821402
C	-0.653728	4.037821	-1.005916
H	-1.078938	2.876391	0.801715
H	0.156033	4.618573	-0.564318
H	-0.417305	3.840591	-2.048892
H	-1.557948	4.644435	-0.956878
C	-3.521226	-1.552229	0.881820
H	-1.945287	-2.678321	0.130985
H	-3.854998	-2.378510	1.498654
C	-4.347358	-0.314817	1.009853
H	-4.280349	0.068480	2.028201
H	-4.023624	0.464900	0.327323
H	-5.394403	-0.546581	0.815553
H	4.348067	0.868235	0.211275

3R_conformer 2

Num. Imaginary Frequencies: 0

C	-1.077678	-1.349908	-1.902259
C	0.242674	-1.374176	-1.984016
H	-1.679411	-1.858484	-2.644361
C	1.144992	-0.698984	-0.992923
H	0.733753	-1.879945	-2.804653
C	0.380232	-0.422275	0.301532
C	2.396260	-1.530720	-0.701619
H	1.465774	0.258258	-1.426629
C	-0.947517	0.292151	-0.005743
H	0.133703	-1.383043	0.769761
C	1.265836	0.362716	1.278014
C	-1.849604	-0.675005	-0.804568
H	-1.429430	0.533178	0.946764
C	-0.744844	1.565733	-0.771473
C	-2.464368	-1.716252	0.098491
H	-2.655673	-0.088433	-1.260342
C	3.317640	-0.818892	0.299248
H	2.106417	-2.498053	-0.287525
H	2.952111	-1.707330	-1.624810
O	0.591386	0.682797	2.477686
C	2.545038	-0.435047	1.568606
H	1.511809	1.339456	0.845260
H	4.133861	-1.497196	0.577642
O	3.984168	0.285463	-0.277533
H	3.194845	0.151565	2.221601
H	2.272917	-1.352842	2.095524
H	0.355600	-0.140148	2.926152
C	-0.793590	2.772809	-0.231713
H	-0.561880	1.453833	-1.832929
C	-0.594250	4.049491	-0.979891
H	-0.980128	2.877618	0.830193
H	-0.407125	3.860012	-2.034382
H	-1.477368	4.681277	-0.886461
H	0.248708	4.601659	-0.564306
C	-3.528347	-1.547568	0.869963
H	-1.960569	-2.674616	0.103779
H	-3.862561	-2.377012	1.482185
C	-4.348031	-0.307458	1.011591
H	-4.263018	0.075301	2.028841
H	-4.033262	0.471746	0.324194
H	-5.398829	-0.535763	0.834737
H	3.328661	0.943195	-0.540899

3R_conformer 3

Num. Imaginary Frequencies: 0

C	-1.070520	-1.339250	-1.907212
C	0.250312	-1.373140	-1.978396
H	-1.671092	-1.834739	-2.659260
C	1.149925	-0.718273	-0.974186
H	0.741610	-1.875860	-2.801300
C	0.380543	-0.426922	0.313808
C	2.385974	-1.564565	-0.662954
H	1.506064	0.235662	-1.385668
C	-0.940823	0.293672	-0.003771
H	0.125245	-1.380874	0.792706
C	1.271348	0.363965	1.280470
C	-1.843839	-0.668847	-0.807931
H	-1.428082	0.539342	0.944940
C	-0.726607	1.564405	-0.770097
C	-2.462050	-1.712970	0.089154
H	-2.647766	-0.078325	-1.262484
C	3.303169	-0.809857	0.307125
H	2.088911	-2.511452	-0.208492

H	2.928252	-1.788008	-1.585174
O	0.596969	0.707537	2.473575
C	2.543808	-0.438453	1.581834
H	1.545430	1.321932	0.826880
H	4.149253	-1.462849	0.585016
O	3.809000	0.383127	-0.261325
H	3.194280	0.156031	2.225494
H	2.276015	-1.356352	2.110434
H	0.342181	-0.108540	2.924833
C	-0.794635	2.773426	-0.237618
H	-0.518157	1.448734	-1.826204
C	-0.583648	4.047647	-0.986761
H	-1.003666	2.882126	0.819641
H	-0.385893	3.854141	-2.038455
H	-1.464098	4.684564	-0.903547
H	0.259182	4.594663	-0.564266
C	-3.527175	-1.546728	0.859557
H	-1.959301	-2.671964	0.091350
H	-3.863813	-2.378745	1.467031
C	-4.345846	-0.306404	1.005207
H	-4.273054	0.064102	2.027892
H	-4.019932	0.479838	0.331235
H	-5.394852	-0.529906	0.811840
H	4.285911	0.164905	-1.071815

3R_conformer 4

Num. Imaginary Frequencies: 0

C	-1.364322	-1.268608	-1.554526
C	-0.075566	-1.471715	-1.774505
H	-2.109089	-1.746230	-2.177873
C	1.012227	-0.851857	-0.951418
H	0.247873	-2.098323	-2.595145
C	0.455556	-0.403689	0.400315
C	2.192534	-1.804023	-0.748656
H	1.404498	0.025018	-1.481439
C	-0.812299	0.446397	0.207172
H	0.175305	-1.298634	0.969668
C	1.545077	0.333577	1.189516
C	-1.916848	-0.409560	-0.450679
H	-1.156355	0.776693	1.193610
C	-0.549431	1.662371	-0.630920
C	-2.601186	-1.292214	0.557696
H	-2.653581	0.272102	-0.886499
C	3.298339	-1.108260	0.047999
H	1.871625	-2.699350	-0.213526
H	2.596142	-2.102880	-1.717288
O	1.081812	0.825479	2.430550
C	2.751586	-0.594664	1.384399
H	1.850165	1.226700	0.633620
H	4.101838	-1.837400	0.254090
O	3.817914	-0.065300	-0.754480
H	3.530769	-0.056977	1.930760
H	2.449744	-1.454082	1.988167
H	0.786201	0.076456	2.965121
C	-0.455607	2.891594	-0.151359
H	-0.448168	1.486289	-1.694247
C	-0.196316	4.109826	-0.974964
H	-0.557954	3.061012	0.913634
H	-1.010264	4.824879	-0.856496
H	0.720565	4.597765	-0.644543
H	-0.100145	3.855682	-2.027906
C	-3.882733	-1.290012	0.894373
H	-1.946474	-2.010286	1.035405
H	-4.216434	-2.002167	1.640838
C	-4.972645	-0.414540	0.368990
H	-5.458373	0.102772	1.195982
H	-4.607582	0.325275	-0.336617
H	-5.727963	-1.025700	-0.125489
H	4.489646	0.412207	-0.252973

3R_conformer 5

Num. Imaginary Frequencies: 0

C	-1.425848	-1.231630	-1.500689
C	-0.146734	-1.458992	-1.750455
H	-2.192942	-1.706808	-2.097840
C	0.971592	-0.845370	-0.959832
H	0.146862	-2.103193	-2.568359
C	0.450096	-0.394750	0.405014
C	2.142172	-1.816436	-0.788707
H	1.334678	0.033614	-1.509369
C	-0.802287	0.484359	0.236489
H	0.159369	-1.288846	0.969351
C	1.564455	0.310983	1.190129
C	-1.938151	-0.345777	-0.398162
H	-1.116121	0.822609	1.230017
C	-0.527326	1.693696	-0.608732
C	-2.637048	-1.197995	0.626380
H	-2.661070	0.351794	-0.832182
C	3.283628	-1.180804	0.017343
H	1.801872	-2.714226	-0.269534
H	2.527005	-2.113352	-1.766511
O	1.123003	0.781777	2.447283
C	2.762064	-0.635225	1.353499
H	1.864719	1.221512	0.658749
H	4.043504	-1.944958	0.223444
O	3.985432	-0.204104	-0.724076
H	3.569080	-0.108274	1.867104
H	2.459160	-1.481478	1.974986
H	0.868096	0.022518	2.988087
C	-0.335740	2.911459	-0.128511
H	-0.503530	1.523671	-1.677987
C	-0.062754	4.121282	-0.959912
H	-0.363638	3.075899	0.941665
H	-0.829083	4.876426	-0.786420
H	0.896532	4.559069	-0.684060
H	-0.046619	3.873162	-2.018625
C	-3.933911	-1.232093	0.895785
H	-1.980936	-1.859097	1.178283
H	-4.278666	-1.913908	1.665030
C	-5.028125	-0.438137	0.261091

H	-5.714021	-1.108763	-0.256949
H	-5.597079	0.083990	1.029795
H	-4.653796	0.291044	-0.450755
H	3.382656	0.518801	-0.937953

3R_conformer 6

Num. Imaginary Frequencies: 0

C	-1.515127	-1.148134	-1.419757
C	-0.256143	-1.442370	-1.700416
H	-2.321595	-1.584303	-1.994383
C	0.911640	-0.886368	-0.943978
H	-0.018779	-2.103619	-2.523222
C	0.454184	-0.391933	0.428536
C	2.036580	-1.911946	-0.791448
H	1.331246	-0.041188	-1.503541
C	-0.758828	0.544597	0.286905
H	0.139730	-1.263306	1.016891
C	1.627766	0.272445	1.159675
C	-1.951112	-0.232588	-0.307709
H	-1.029265	0.907123	1.284938
C	-0.449731	1.732409	-0.575975
C	-2.662323	-1.046558	0.739955
H	-2.652424	0.497897	-0.724263
C	3.223701	-1.285459	-0.056858
H	1.685548	-2.781456	-0.233117
H	2.370711	-2.241841	-1.776322
O	1.258988	0.796944	2.419043
C	2.778831	-0.732458	1.301369
H	1.963392	1.142113	0.584595
H	3.988120	-2.064009	0.114125
O	3.767903	-0.281455	-0.892028
H	3.617553	-0.244951	1.805020
H	2.451904	-1.568118	1.925116
H	0.943874	0.070019	2.972599
C	-0.236389	2.953625	-0.114581
H	-0.419136	1.542968	-1.641423
C	0.070846	4.143101	-0.963332
H	-0.268232	3.137170	0.952560
H	-0.676537	4.920759	-0.806808
H	1.040376	4.559008	-0.689378
H	0.087455	3.876329	-2.017455
C	-3.970699	-1.158509	0.916631
H	-2.006613	-1.609877	1.391310
H	-4.327741	-1.798029	1.715968
C	-5.061325	-0.506477	0.131718
H	-5.651940	-1.268629	-0.377106
H	-5.728907	0.032226	0.803738
H	-4.678683	0.185871	-0.611870
H	4.493575	0.153681	-0.428515

3R_conformer 7

Num. Imaginary Frequencies: 0

C	-1.362777	-1.264395	-1.558467
C	-0.073334	-1.467351	-1.774017
H	-2.105046	-1.740935	-2.185586
C	1.011497	-0.851245	-0.943390
H	0.251671	-2.093960	-2.594275
C	0.450327	-0.401741	0.405518
C	2.185756	-1.808331	-0.728181
H	1.403688	0.031535	-1.465276
C	-0.816069	0.449197	0.205511
H	0.167012	-1.294937	0.976012
C	1.537694	0.336238	1.198482
C	-1.918717	-0.406313	-0.455976
H	-1.162165	0.781061	1.190527
C	-0.548371	1.663642	-0.633569
C	-2.607584	-1.288697	0.549561
H	-2.653648	0.275845	-0.894011
C	3.293994	-1.107723	0.066915
H	1.855406	-2.690587	-0.176826
H	2.577623	-2.140703	-1.692740
O	1.064903	0.821254	2.438484
C	2.748093	-0.585210	1.396575
H	1.849238	1.229792	0.648098
H	4.097138	-1.834302	0.282753
O	3.841770	-0.011230	-0.639826
H	3.533818	-0.031802	1.913483
H	2.458015	-1.437429	2.015781
H	0.798500	0.066005	2.979911
C	-0.424879	2.889380	-0.151998
H	-0.469549	1.490079	-1.699392
C	-0.157885	4.105658	-0.975837
H	-0.503989	3.056017	0.915310
H	0.782983	4.564154	-0.671312
H	-0.102206	3.856896	-2.033038
H	-0.946573	4.842801	-0.826430
C	-3.889753	-1.282238	0.883835
H	-1.955890	-2.008658	1.028541
H	-4.227309	-1.993646	1.629240
C	-4.975531	-0.402003	0.357798
H	-4.607232	0.335630	-0.348395
H	-5.734014	-1.009944	-0.135827
H	-5.458422	0.118459	1.184459
H	4.182797	-0.324769	-1.486814

3R_conformer 8

Num. Imaginary Frequencies: 0

C	-1.475624	-1.171792	-1.463008
C	-0.207056	-1.441320	-1.724668
H	-2.264074	-1.616010	-2.056286
C	0.937899	-0.874647	-0.940857
H	0.054471	-2.091129	-2.549528
C	0.449473	-0.394769	0.425648
C	2.072098	-1.886688	-0.766220
H	1.351407	-0.013225	-1.481464
C	-0.776973	0.521229	0.266322
H	0.140425	-1.272663	1.006847
C	1.599774	0.286466	1.179432
C	-1.946011	-0.274557	-0.350722
H	-1.068486	0.875718	1.261112
C	-0.473736	1.716051	-0.589193

C	-2.654748	-1.109647	0.681354
H	-2.655185	0.445139	-0.771938
C	3.240855	-1.239326	-0.013813
H	1.720275	-2.751607	-0.200866
H	2.411857	-2.238159	-1.743687
O	1.195387	0.792303	2.435329
C	2.769411	-0.693754	1.334848
H	1.935591	1.164325	0.618249
H	4.016890	-2.002627	0.172213
O	3.812152	-0.168517	-0.741076
H	3.599091	-0.180665	1.823909
H	2.459862	-1.532114	1.963505
H	0.911834	0.050207	2.986136
C	-0.254352	2.933006	-0.119722
H	-0.451802	1.535656	-1.656583
C	0.050067	4.128364	-0.961024
H	-0.276649	3.107045	0.949102
H	1.028935	4.530177	-0.699297
H	0.045632	3.873259	-2.018159
H	-0.685763	4.912262	-0.782744
C	-3.959857	-1.183383	0.897841
H	-1.999197	-1.723070	1.286156
H	-4.312475	-1.845481	1.680674
C	-5.053309	-0.459708	0.182780
H	-4.673676	0.248196	-0.547564
H	-5.693053	-1.177857	-0.330642
H	-5.672132	0.076248	0.901976
H	4.108440	-0.497074	-1.599118

3R_conformer 9

Num. Imaginary Frequencies: 0

C	-1.045713	-1.426336	-1.848996
C	0.274711	-1.446041	-1.927397
H	-1.644309	-1.956343	-2.578859
C	1.170928	-0.742202	-0.954752
H	0.767970	-1.971232	-2.735079
C	0.409619	-0.411948	0.328192
C	2.420790	-1.558717	-0.620679
H	1.511755	0.199649	-1.405965
C	-0.931374	0.268456	0.007115
H	0.180784	-1.340492	0.862525
C	1.295568	0.436306	1.244356
C	-1.821414	-0.730158	-0.768508
H	-1.411165	0.512915	0.959186
C	-0.760164	1.527954	-0.786395
C	-2.424246	-1.753604	0.161362
H	-2.633646	-0.163181	-1.238279
C	3.336946	-0.751669	0.305891
H	2.139233	-2.487194	-0.121090
H	2.957198	-1.815941	-1.537696
O	0.572346	0.724159	2.427182
C	2.586997	-0.326162	1.568908
H	1.550729	1.374191	0.726802
H	4.192399	-1.382203	0.605904
O	3.826503	0.420429	-0.319528
H	3.246086	0.308145	2.166129
H	2.333330	-1.212793	2.152642
H	1.130237	1.254519	3.008868
C	-0.939601	2.745204	-0.300326
H	-0.491344	1.396028	-1.826907
C	-0.781371	4.008494	-1.080999
H	-1.215398	2.869778	0.739830
H	-0.508289	3.797695	-2.112227
H	-1.711551	4.576418	-1.070923
H	-0.009303	4.633900	-0.632836
C	-3.503707	-1.586135	0.911022
H	-1.894872	-2.696750	0.209888
H	-3.824992	-2.402101	1.547770
C	-4.355668	-0.362940	1.000150
H	-5.398908	-0.622150	0.820826
H	-4.288734	0.056045	2.004346
H	-4.050766	0.399400	0.289896
H	4.288958	0.171968	-1.129637

3R_conformer 10

Num. Imaginary Frequencies: 0

C	1.169514	0.341724	2.047469
C	-0.144544	0.413832	2.179069
H	1.814726	0.497977	2.902541
C	-1.097280	0.203536	1.039217
H	-0.591057	0.655570	3.134127
C	-0.407579	-0.581424	-0.077162
C	-2.365826	-0.520163	1.498637
H	-1.375668	1.193698	0.651491
C	0.928364	0.090019	-0.451027
H	-0.173089	-1.584223	0.300293
C	-1.356965	-0.740962	-1.273332
C	1.875584	0.010433	0.764852
H	1.370553	-0.490958	-1.268552
C	0.723231	1.486201	-0.968733
C	2.484047	-1.367312	0.874541
H	2.683125	0.735115	0.614653
C	-3.340446	-0.752206	0.336330
H	-2.097831	-1.486660	1.929420
H	-2.871606	0.066967	2.267965
O	-0.748182	-1.411997	-2.357731
C	-2.633370	-1.464717	-0.824727
H	-1.610951	0.244557	-1.679243
H	-4.167496	-1.384002	0.683647
O	-3.979939	0.439036	-0.073875
H	-3.319157	-1.542739	-1.671119
H	-2.373036	-2.475558	-0.502004
H	-0.525270	-2.309481	-2.077553
C	0.880373	2.616182	-0.298398
H	0.413863	1.530284	-2.006639
C	0.656730	3.975077	-0.877243
H	1.185243	2.603941	0.739509
H	0.341456	3.911979	-1.916260
H	-0.106302	4.505594	-0.307575
H	1.573089	4.562669	-0.823179

C	3.531909	-1.813662	0.197466
H	1.989618	-2.033056	1.570753
H	3.861857	-2.832476	0.363956
C	4.338775	-1.062387	-0.809804
H	5.393248	-1.092122	-0.536167
H	4.240798	-1.536046	-1.786815
H	4.023860	-0.026993	-0.896747
H	-3.310104	1.064627	-0.376590

TMS

Num. Imaginary Frequencies: 0

Si	0.000052	0.000353	-0.000012
C	-1.408936	1.257563	-0.107862
C	1.657021	0.890297	-0.200322
C	-0.052633	-0.862982	1.681940
C	-0.195476	-1.285175	-1.373774
H	-2.385378	0.776023	0.011584
H	-1.323675	2.023225	0.670658
H	-1.413990	1.772604	-1.074438
H	2.497657	0.191124	-0.136614
H	1.725687	1.397447	-1.168639
H	1.801240	1.648018	0.577239
H	-1.147756	-1.819807	-1.290313
H	-0.166690	-0.820367	-2.365100
H	0.604180	-2.032804	-1.339370
H	-0.999672	-1.392675	1.830658
H	0.752840	-1.598581	1.780520
H	0.054968	-0.147372	2.504082

10. References

(1) Inanaga, J.; Hirata, K.; Saeki, H.; Katsuki, T.; Yamaguchi, M. Esterification by Means of Mixed Anhydride and Its Application to Large-Ring Lactonization. *Bull. Chem. Soc. Jpn.* **1979**, *52*, 1989–1993.

(2) Fleming, I.; Trost, B. M.; Paquette, L. A. Roush, W. R., Intramolecular Diels-Alder Reactions. In *Comprehensive Organic Synthesis*; Eds. Pergamon Press: Oxford, 1991; Vol. 5, pp 513–550.

(3) Takao, K. I.; Munakata, R.; Tadano, K. I. Recent Advances in Natural Product Synthesis by Using Intramolecular Diels-Alder Reactions. *Chem. Rev.* **2005**, *105*, 4779–4807.

(4) Juhl, M.; Tanner, D. Recent Applications of Intramolecular Diels–Alder Reactions to Natural Product Synthesis. *Chem. Soc. Rev.* **2009**, *38*, 2983–2992.

(5) Heravi, M. M.; Vavsari, V. F. Recent Applications of Intramolecular Diels-Alder Reaction in Total Synthesis of Natural Products. *RSC Adv.* **2015**, *5*, 50890–50912.

(6) Nicolaou, K. C.; Shah, A. A.; Korman, H.; Khan, T.; Shi, L.; Worawalai, W.; Theodorakis, E. A. Total Synthesis and Structural Revision of Antibiotic CJ-16,264. *Angew. Chem. Int. Ed.* **2015**, *127*, 9335–9340.

(7) Nicolaou, K. C.; Pulukuri, K. K.; Rigol, S.; Buchman, M.; Shah, A. A.; Cen, N.; McCurry, M. D.; Beabout, K.; Shamoo, Y. Enantioselective Total Synthesis of Antibiotic CJ-16,264, Synthesis and Biological Evaluation of Designed Analogues, and Discovery of Highly Potent and Simpler Antibacterial Agents. *J. Am. Chem. Soc.* **2017**, *139*, 15868–15877.

(8) Boden, E. P.; Keck, G. E. Proton-Transfer Steps in Steglich Esterification: A Very Practical New Method for Macrolactonization. *J. Org. Chem.* **1985**, *50*, 2394–2395.

(9) Shiina, I.; Kubota, M.; Ibuka, R. A Novel and Efficient Macrolactonization of V-Hydroxycarboxylic Acids Using 2-Methyl-6-Nitrobenzoic Anhydride (MNBA). *Tetrahedron Lett.* **2002**, *43*, 7535–7539.

(10) Rezgui, F.; Moncef, M.; Gaied, E. DMAP-Catalyzed Hydroxymethylation of 2-Cyciohexenones in Aqueous Medium Through Baylis-Hiilman Reaction. *Tetrahedron Lett.* **1998**, *39*, 5965–5966.

(11) Hill, J. S.; Isaacs, N. S. Functionalisation of the α Position of Acrylate Systems by the Addition of Carbonyl Compounds: Highly Pressure-Dependent Reactions. *Tetrahedron Lett.* **1986**, *27*, 5007–5010.

(12) Marsault, E.; As Toro A, A.; Nowak, P.; Deslongchamps, P. The Transannular Diels–Alder Strategy: Applications to Total Synthesis. *Tetrahedron* **2001**, *57*, 4243–4260.

(13) Herbert C. Brown; Krishna S. Bhat. Chiral Synthesis via Organoboranes. 7. Diastereoselective and Enantioselective Synthesis of Erythro- and Threo-Beta-Methylhomoallyl Alcohols via Enantiomeric (*Z*)- and (*E*)-Crotylboranes. *J. Am. Chem. Soc.* **1986**, *108*, 5919–5923.

(14) Kim, I. S.; Ngai, M. Y.; Krische, M. J. Enantioselective Iridium-Catalyzed Carbonyl Allylation from the Alcohol or Aldehyde Oxidation Level via Transfer Hydrogenative Coupling of Allyl Acetate: Departure from Chirally Modified Allyl Metal Reagents in Carbonyl Addition. *J. Am. Chem. Soc.* **2008**, *130*, 14891–14899.

(15) Rychnovsky, S. D.; Rogers, B.; Yang, G. Analysis of Two 13C NMR Correlations for Determining the Stereochemistry of 1,3-Diol Acetonides. *J. Am. Chem. Soc* **1993**, *58*, 671–677.

(16) Garrais, S.; Turkington, J.; Goldring, W. P. D. Synthesis of Isomeric Polyacetylenes Based on Natural Hydroxy Matricaria Esters. *Tetrahedron* **2009**, *65*, 8418–8427.

(17) Raghavan, S.; Nyalata, S. A Short Convergent Synthesis of the [3.2.1]Dioxabicyclooctane Subunit of Sorangicin A via Regioselective Epoxide Opening. *Tetrahedron* **2018**, *74*, 1071–1077.

(18) Sharma, B. M.; Gontala, A.; Kumar, P. Enantioselective Modular Total Synthesis of Macrolides Sch725674 and C-4-Epi-Sch725674. *Eur. J. Org. Chem.* **2016**, 1215–1226.

(19) Takemura, A.; Fujiwara, K.; Shimawaki, K.; Murai, A.; Kawai, H.; Suzuki, T. Synthesis of the EF-Ring Segment of Ciguatoxin CTX1B Based on Novel Regioselective Reduction of Unsaturated Cyanohydrins and Ring-Closing Olefin Metathesis. *Tetrahedron* **2005**, *61*, 7392–7419.

(20) Kirsch, S. F.; Klahn, P.; Menz, H. The use of COP-OAc in the catalyst-controlled synthesis of 1,3-polyols. *Synthesis* **2011**, *22*, 3592–3603.

(21) Gaussian 16, Revision C.01, M. J. Frisch, G. W. Trucks, H. B. Schlegel, G. E. Scuseria, M. A. Robb, J. R. Cheeseman, G. Scalmani, V. Barone, G. A. Petersson, H. Nakatsuji, X. Li, M. Caricato, A. V. Marenich, J. Bloino, B. G. Janesko, R. Gomperts, B. Mennucci, H. P. Hratchian, J. V. Ortiz, A. F. Izmaylov, J. L. Sonnenberg, D. Williams-Young, F. Ding, F. Lipparini, F. Egidi, J. Goings, B. Peng, A. Petrone, T. Henderson, D. Ranasinghe, V. G. Zakrzewski, J. Gao, N. Rega, G. Zheng, W. Liang, M. Hada, M. Ehara, K. Toyota, R. Fukuda, J. Hasegawa, M. Ishida, T. Nakajima, Y. Honda, O. Kitao, H. Nakai, T. Vreven, K. Throssell, J. A. Montgomery, Jr., J. E. Peralta, F. Ogliaro, M. J. Bearpark, J. J. Heyd, E. N. Brothers, K. N. Kudin, V. N. Staroverov, T. A. Keith, R. Kobayashi, J. Normand, K. Raghavachari, A. P. Rendell, J. C. Burant, S. S. Iyengar, J. Tomasi, M. Cossi, J. M. Millam, M. Klene, C. Adamo, R. Cammi, J. W. Ochterski, R. L. Martin, K. Morokuma, O. Farkas, J. B. Foresman, and D. J. Fox, Gaussian, Inc., Wallingford CT, 2016.

(22) Pracht, P.; Grimme, S.; Bannwarth, C.; Bohle, F.; Ehlert, S.; Feldmann, G.; Gorges, J.; Müller, M.; Neudecker, T.; Plett, C.; Spicher, S.; Steinbach, P.; Wesołowski, P. A.; Zeller, F. CREST—A Program for the Exploration of Low-Energy Molecular Chemical Space. *J. Chem. Phys.* **2024**, *160*, 114110.

(23) Becke, A. D. Density-Functional Thermochemistry. III. The Role of Exact Exchange. *J. Chem. Phys.* **1993**, *98*, 5648–5652.

(24) Stephens, P. J.; Devlin, F. J.; Chabalowski, C. F.; Frisch, M. J. *Ab Initio Calculation of Vibrational Absorption and Circular Dichroism Spectra Using Density Functional Force Fields*; 1994; Vol. 98. https://pubs.acs.org/sharingguidelines.

(25) Tomasi, J.; Mennucci, B.; Cammi, R. Quantum Mechanical Continuum Solvation Models. *Chem. Rev.* **2005**, *105*, 2999–3093.

(26) Ditchfield, R. Self-Consistent Perturbation Theory of Diamagnetism I. A Gauge-Invariant LCAO Method for N.M.R. Chemical Shifts. *Mol. Phys.* **1974**, *27*, 789–807.

(27) Wolinskv, K.; Hinton, J. F.; Pulay, P. *Efficient Implementation of the Gauge-Independent Atomic Orbital Method for NMR Chemical Shift Calculations*; 1990; Vol. 112. https://pubs.acs.org/sharingguidelines.

(28) Grimblat, N.; Zanardi, M. M.; Sarotti, A. M. Beyond DP4: An Improved Probability for the Stereochemical Assignment of Isomeric Compounds Using Quantum Chemical Calculations of NMR Shifts. *J. Org. Chem.* **2015**, *80*, 12526–12534.

12. Copies of NMR spectra

^{1}H-NMR (400.16 MHz, CDCl$_3$)

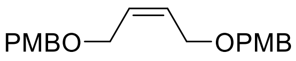

S8

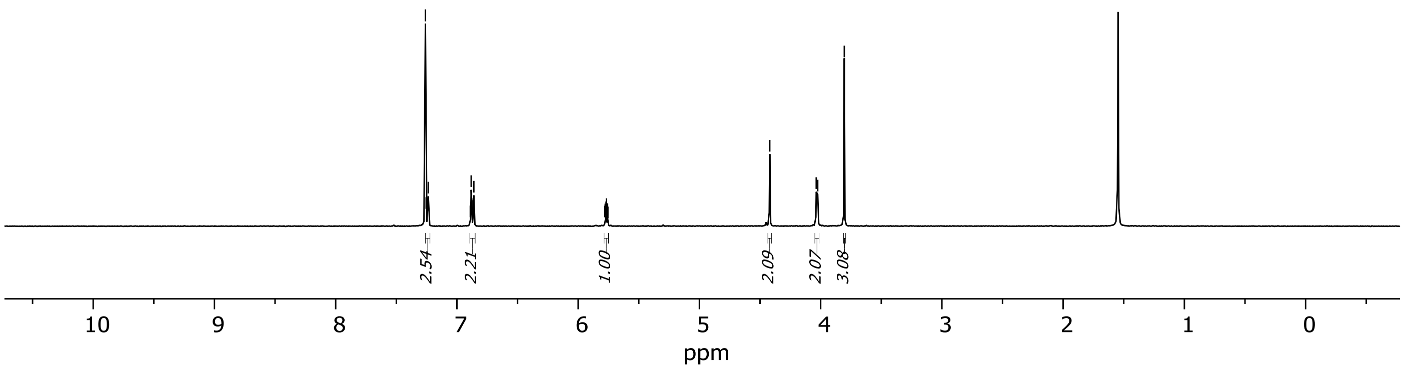

^{13}C-NMR (100.63 MHz, CDCl$_3$)

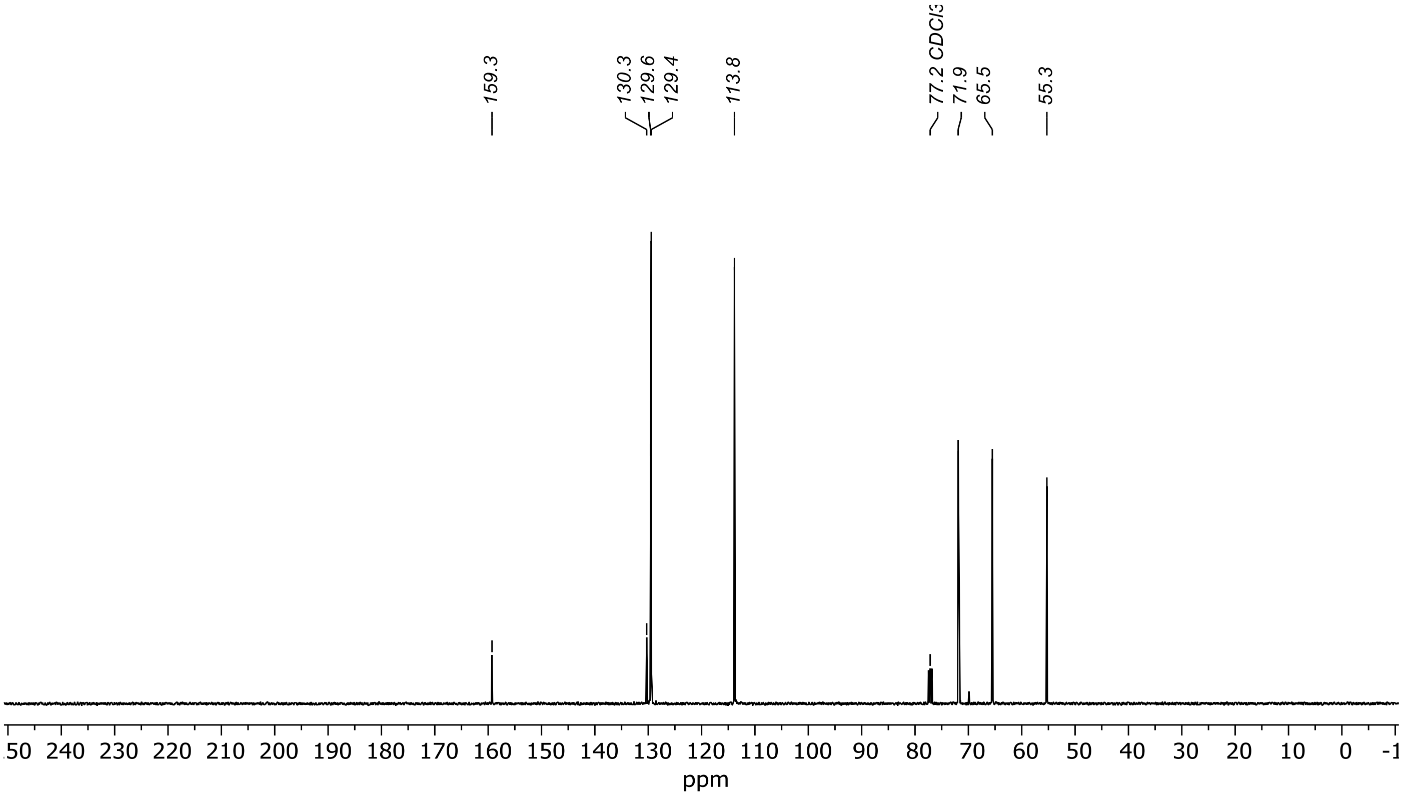

^{1}H-NMR (400.16 MHz, CDCl$_3$)

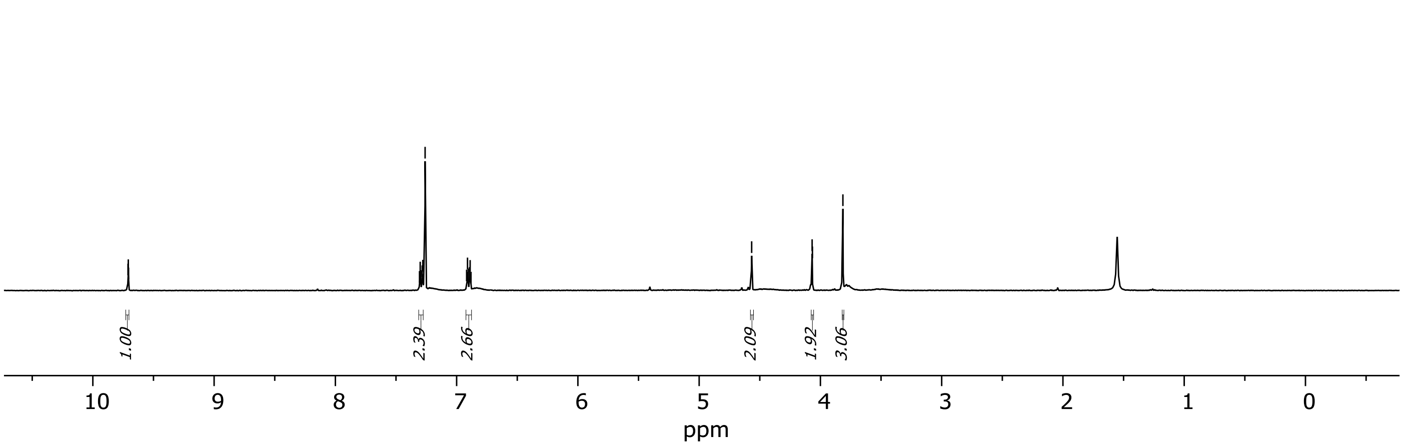

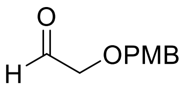

S9

^{13}C-NMR (100.63 MHz, CDCl$_3$)

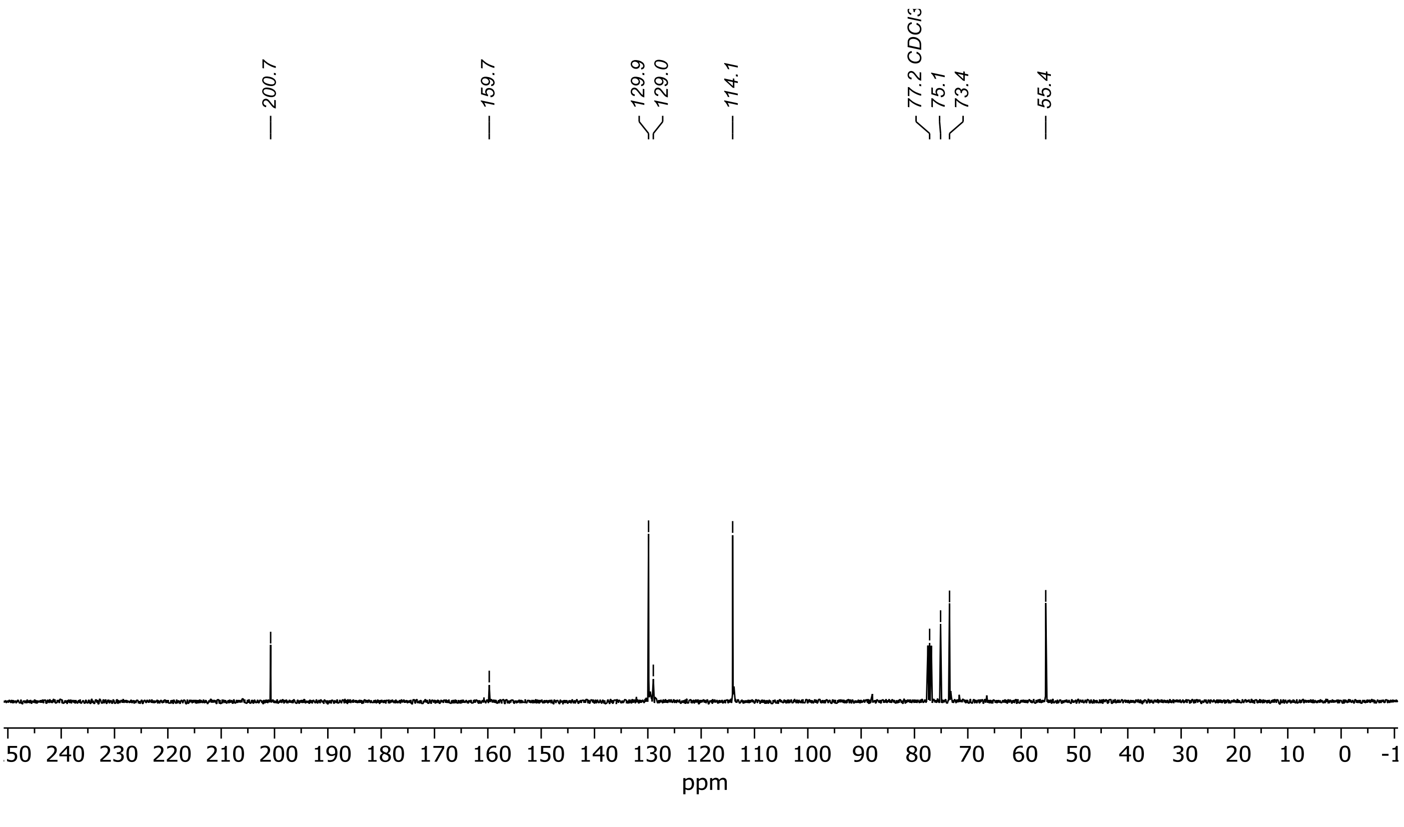

^{1}H-NMR (400.16 MHz, CDCl$_3$)

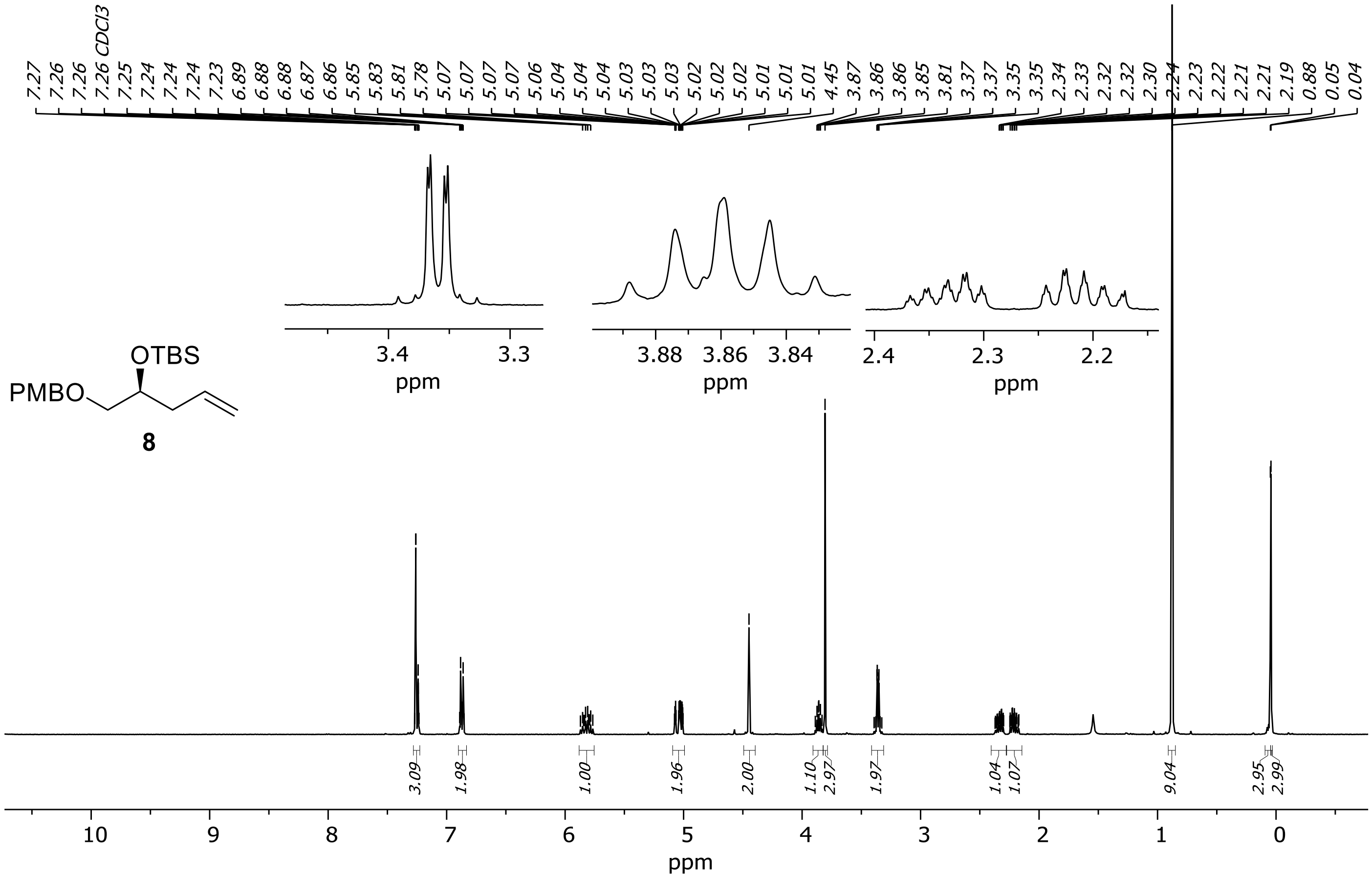

8

^{13}C-NMR (100.63 MHz, CDCl$_3$)

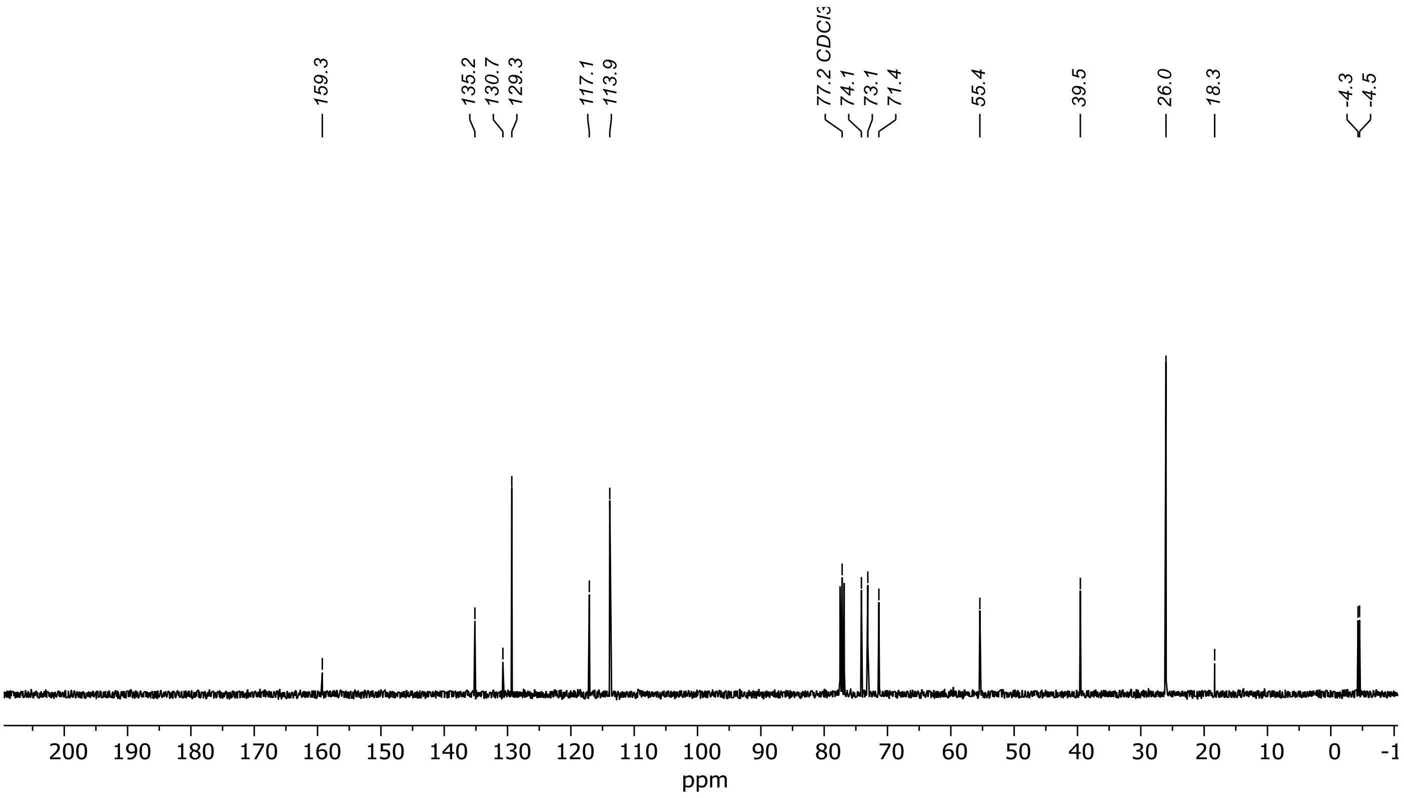

^{1}H-NMR (400.16 MHz, $CDCl_3$)

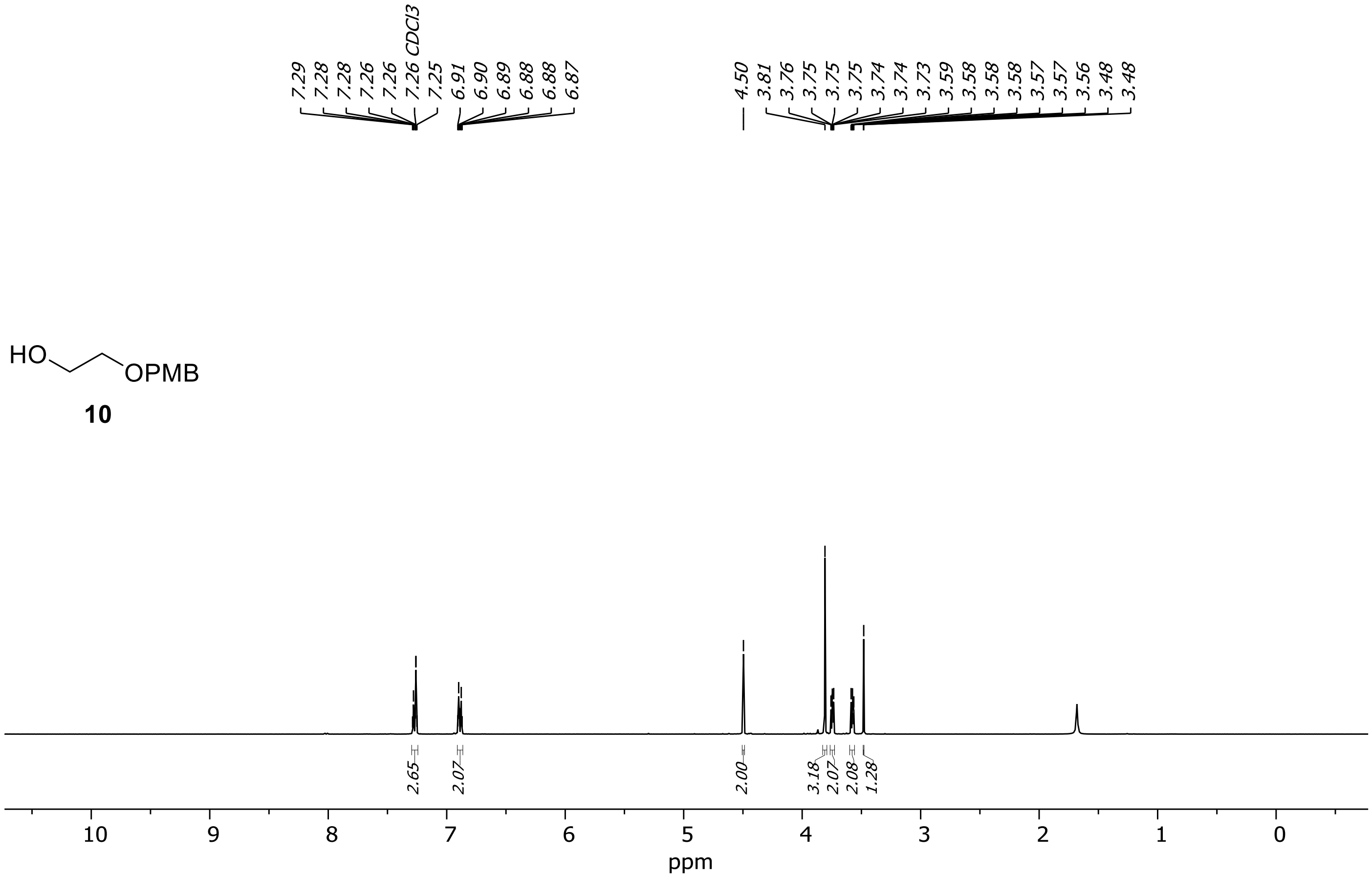

10

^{13}C-NMR (100.63 MHz, $CDCl_3$)

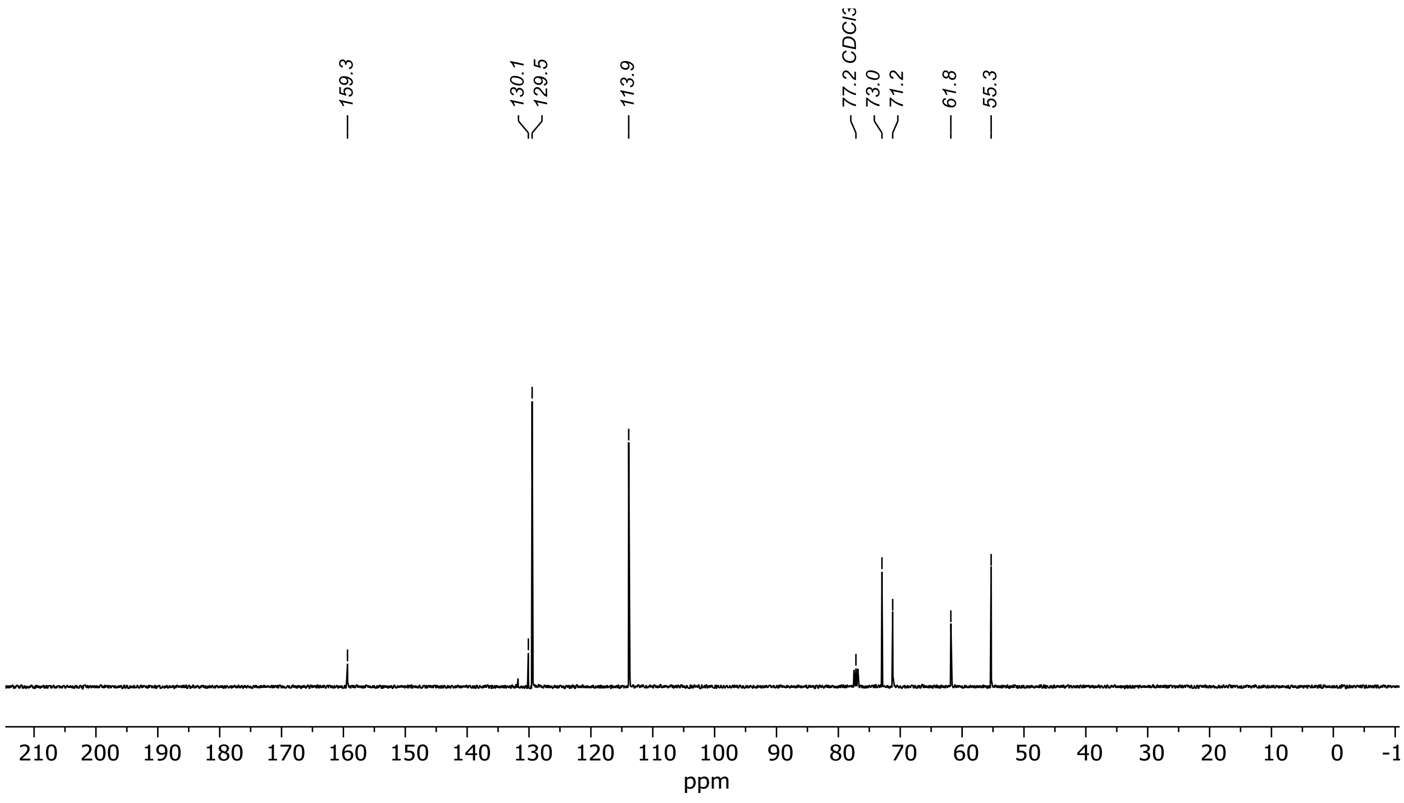

^{1}H-NMR (400.16 MHz, $CDCl_3$)

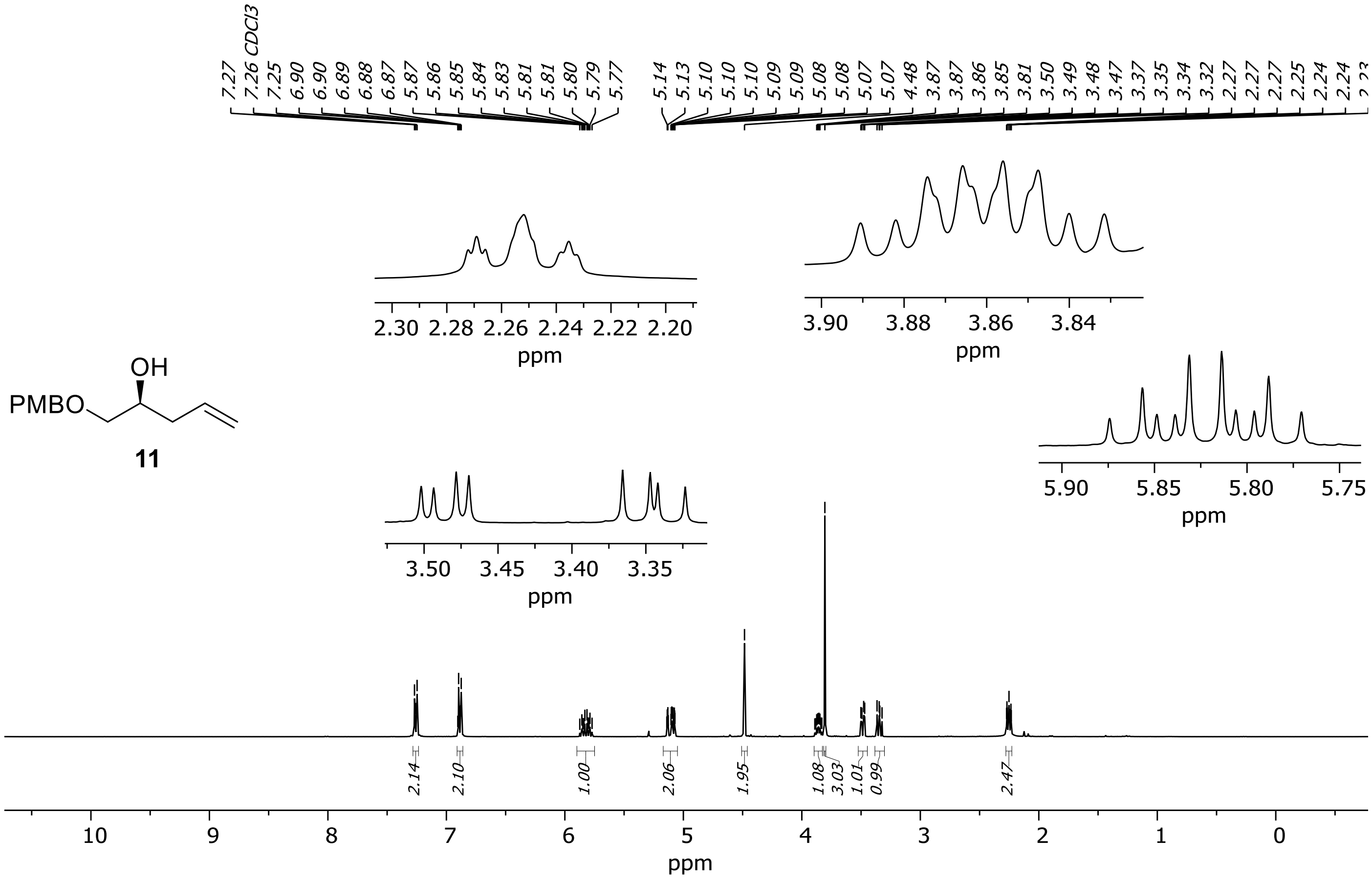

11

^{13}C-NMR (100.63 MHz, $CDCl_3$)

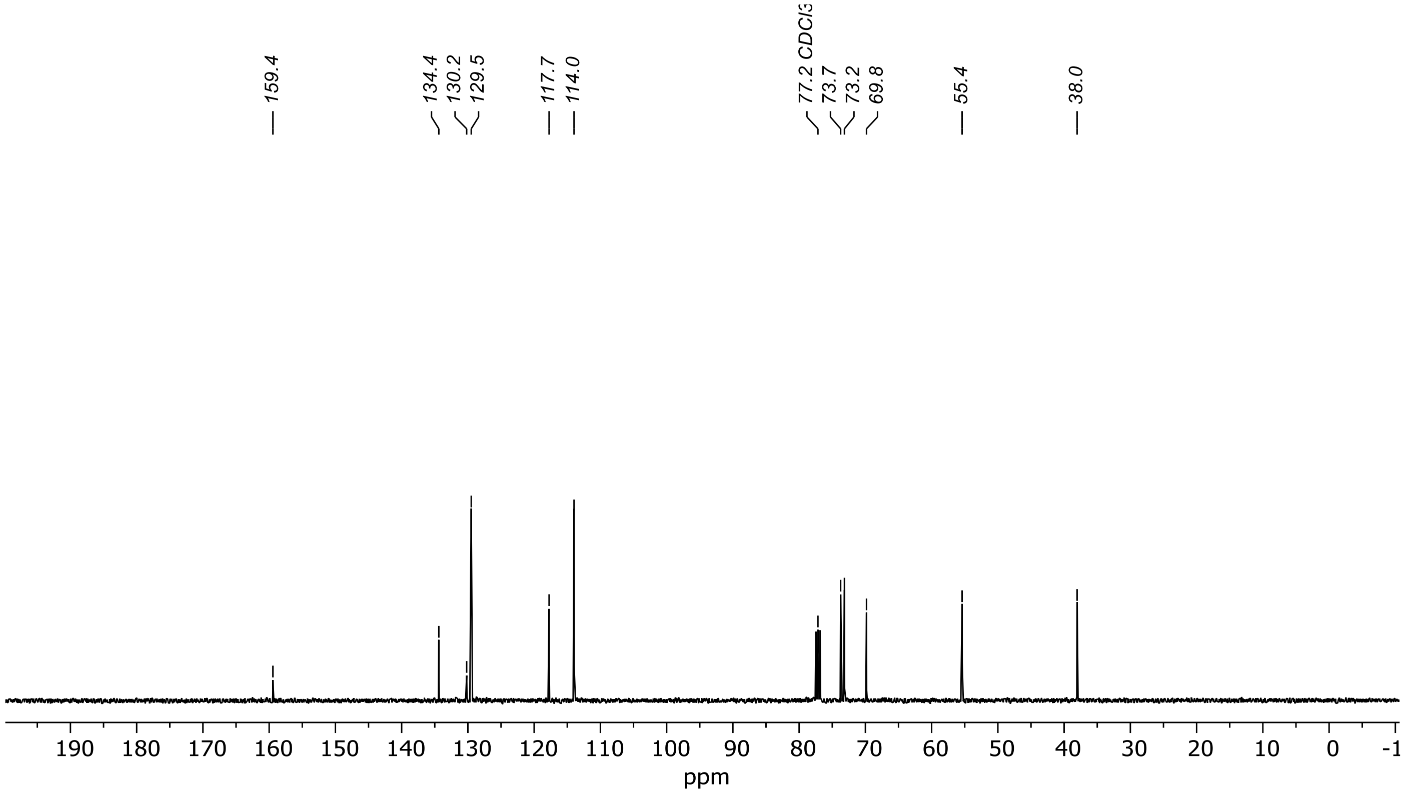

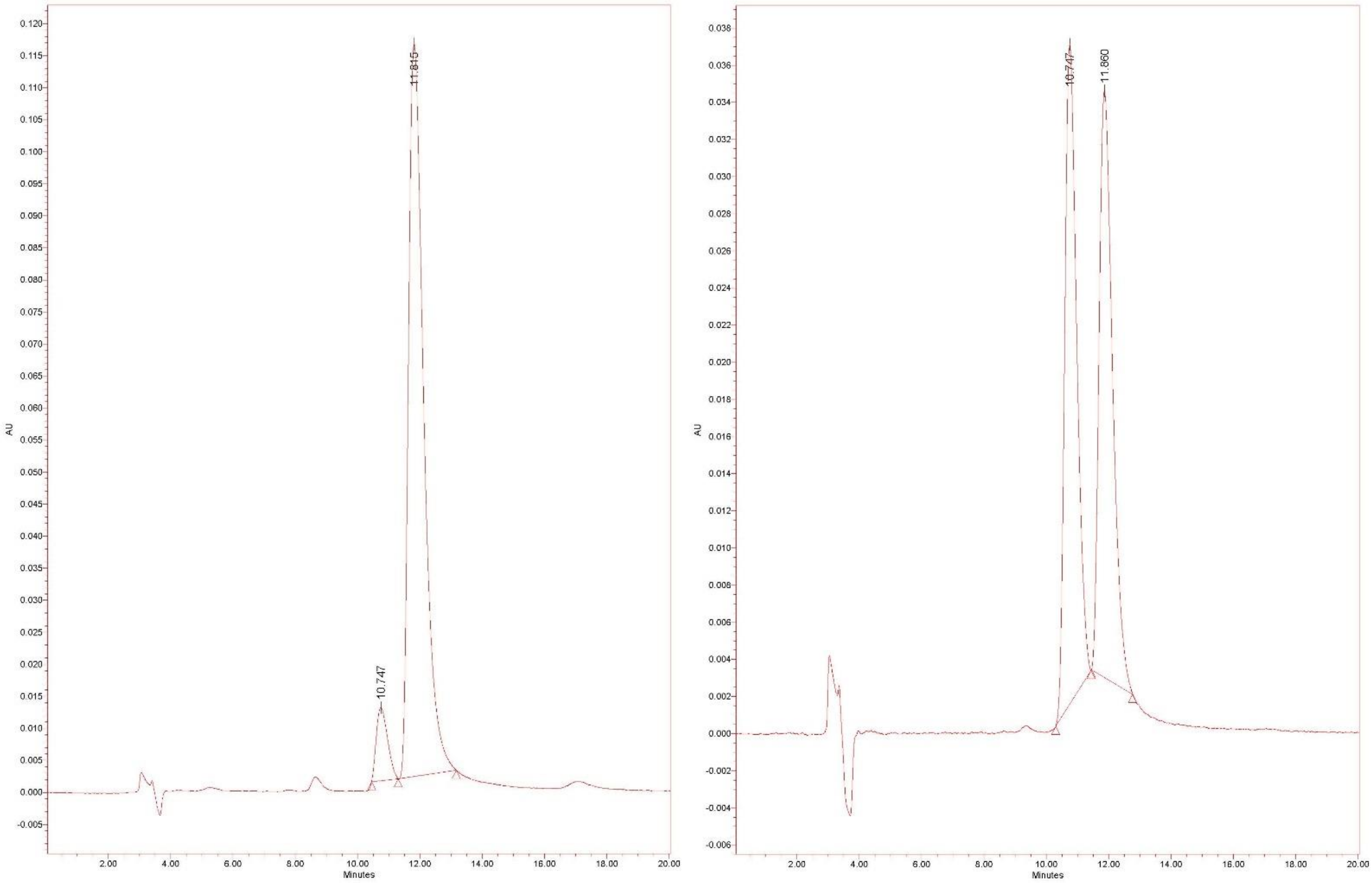

Figure S3. (Chiralcel OD-H, *n*-hexane/^{i}PrOH/CH_2Cl_2 95.5:4:0.5 v/v, 0.7 mL/min, 275 nm), t_R = 10.7 min for the *S*-enantiomer and t_R = 11.9 min for the *R*-enantiomer; *e.e.* = 87%)

^{1}H-NMR (400.16 MHz, CDCl$_3$)

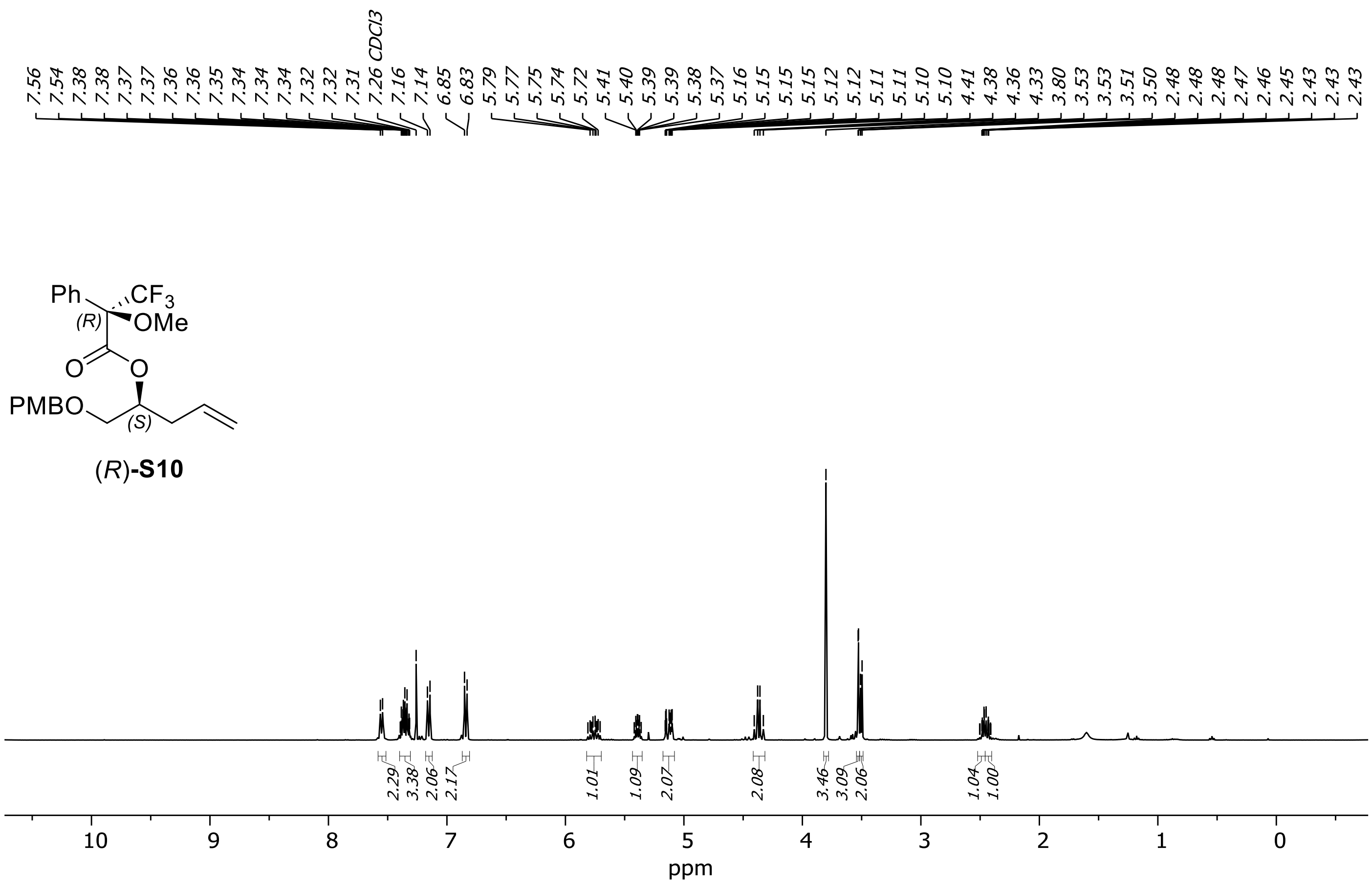

(*R*)-**S10**

^{13}C-NMR (100.63 MHz, CDCl$_3$)

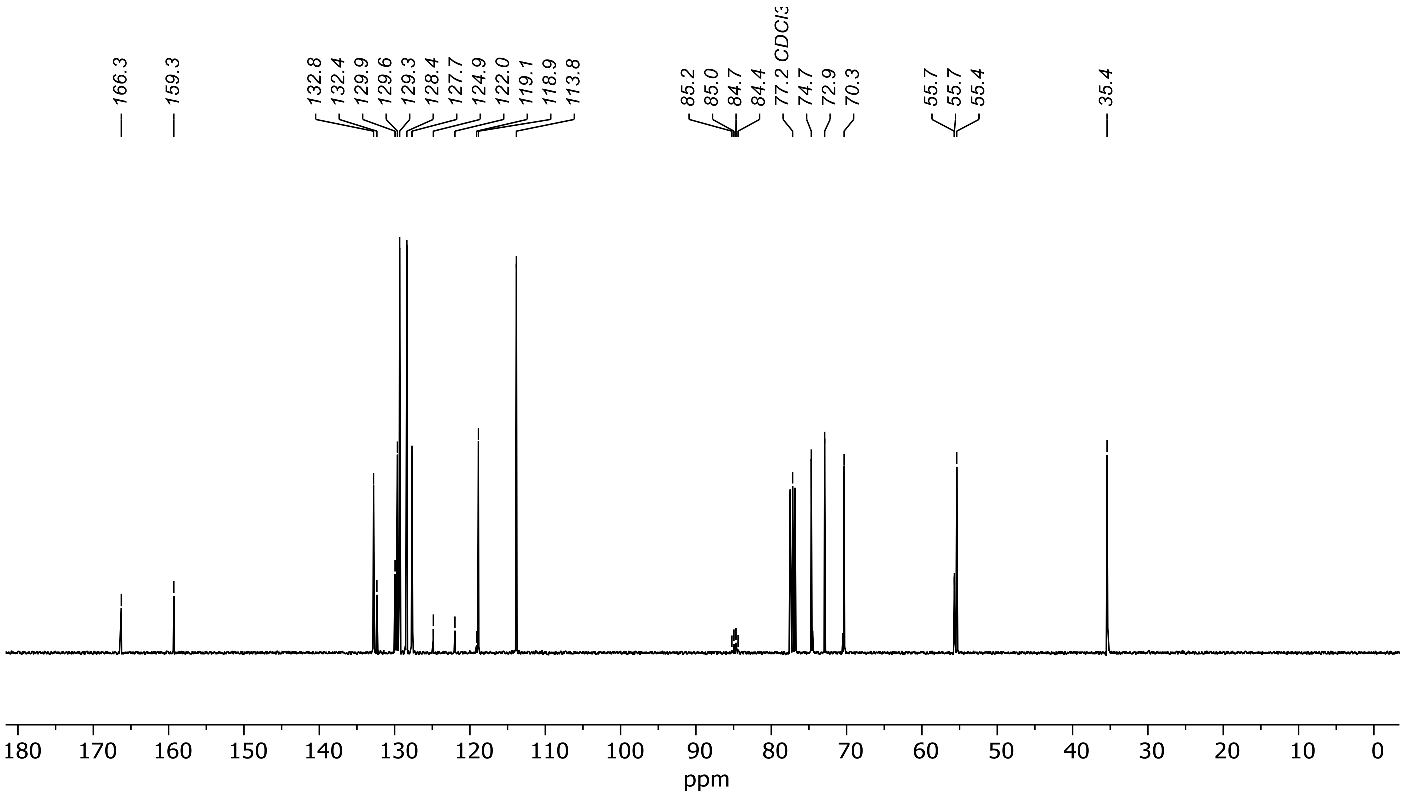

^{1}H-NMR (400.16 MHz, $CDCl_3$)

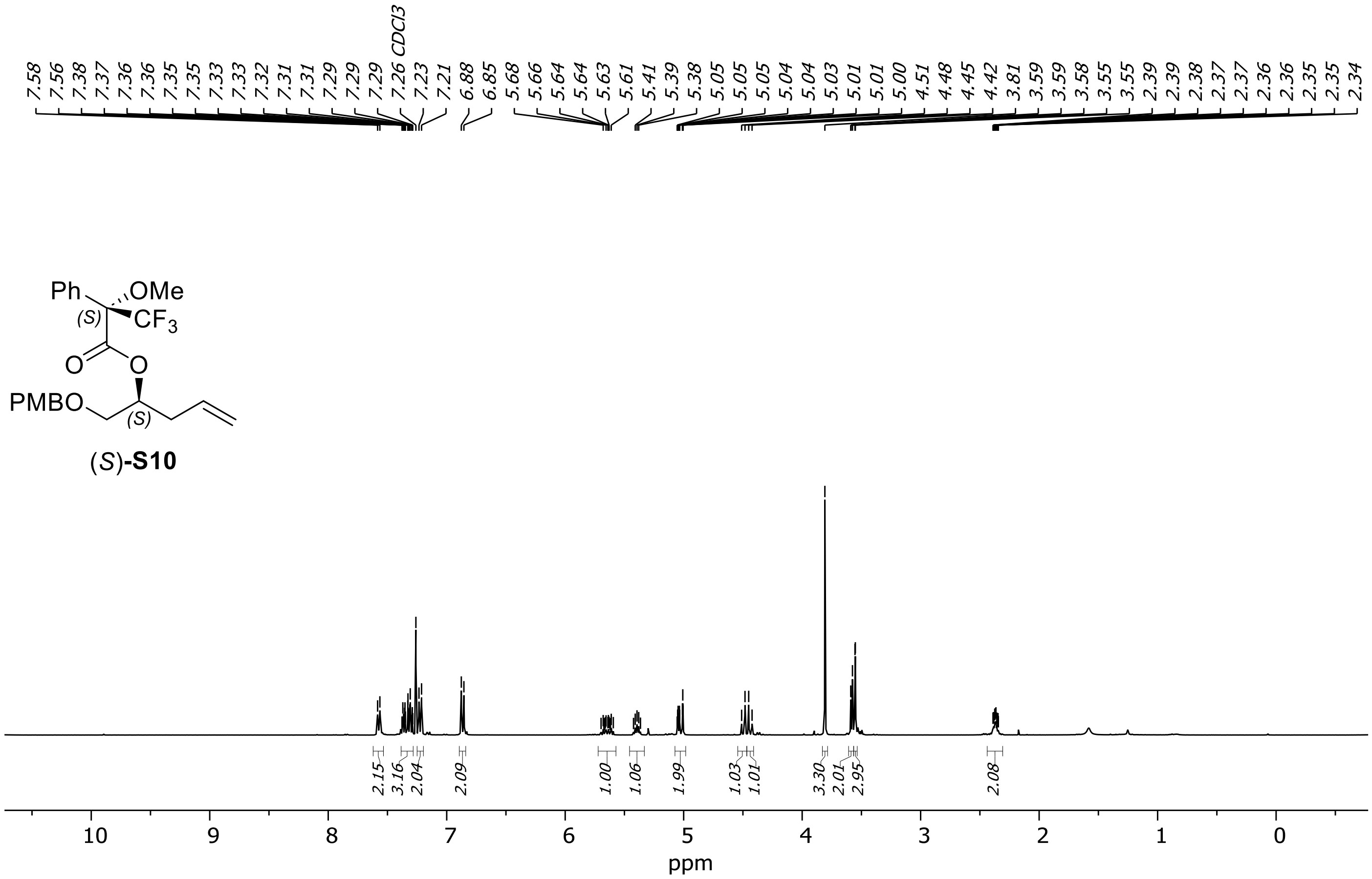

(*S*)-**S10**

^{13}C-NMR (100.63 MHz, $CDCl_3$)

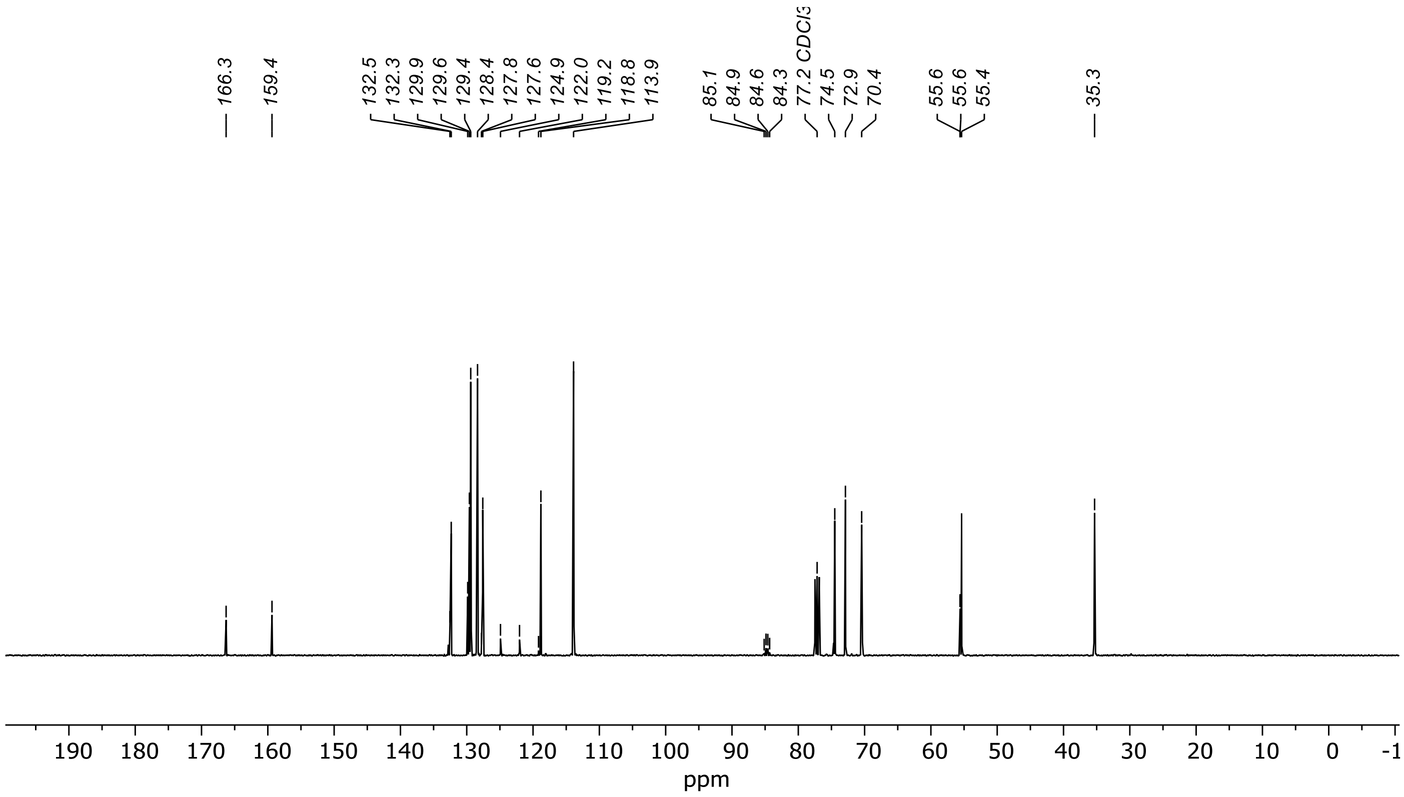

^{1}H-NMR (400.16 MHz, $CDCl_3$)

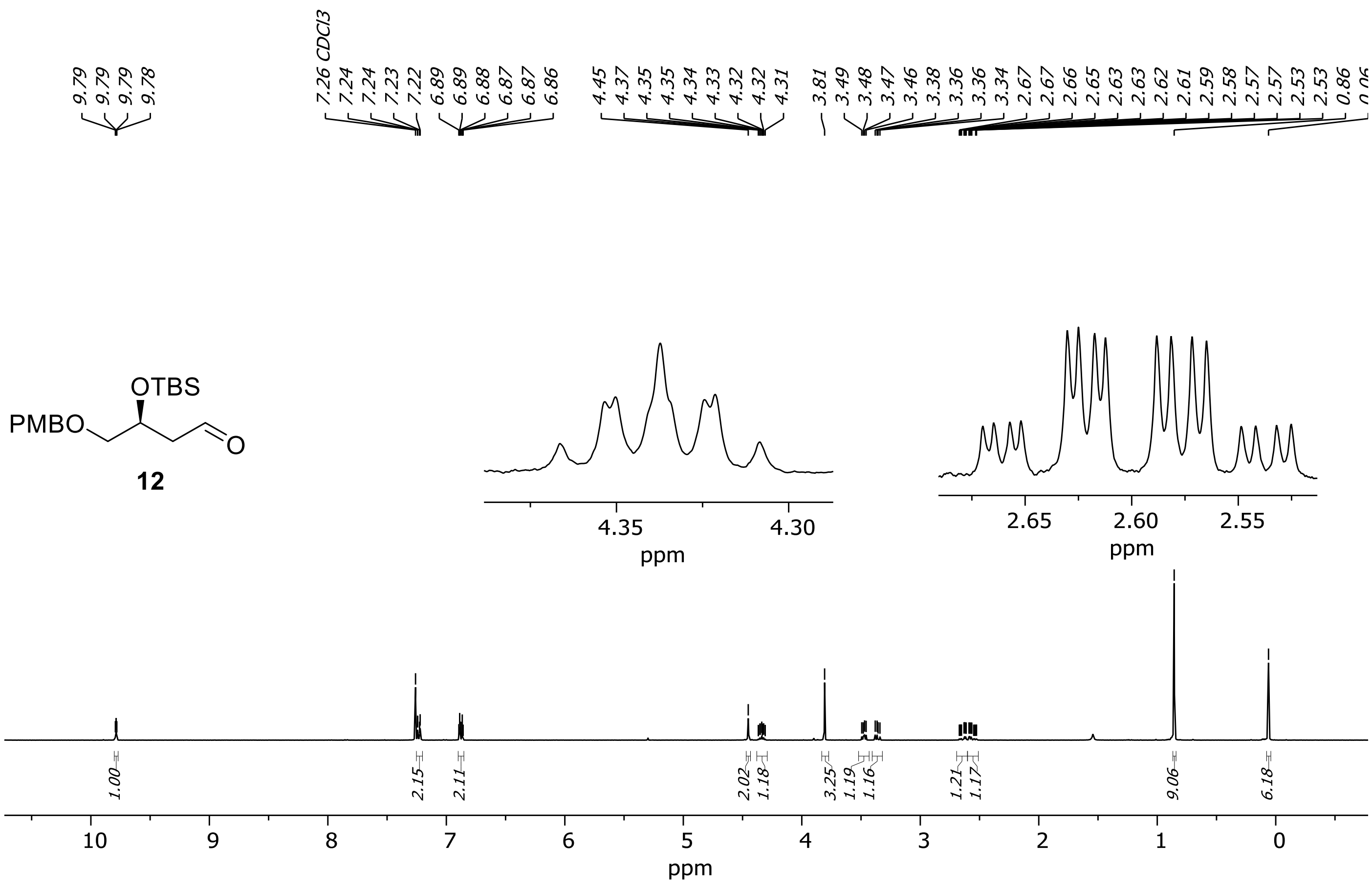

12

^{13}C-NMR (100.63 MHz, $CDCl_3$)

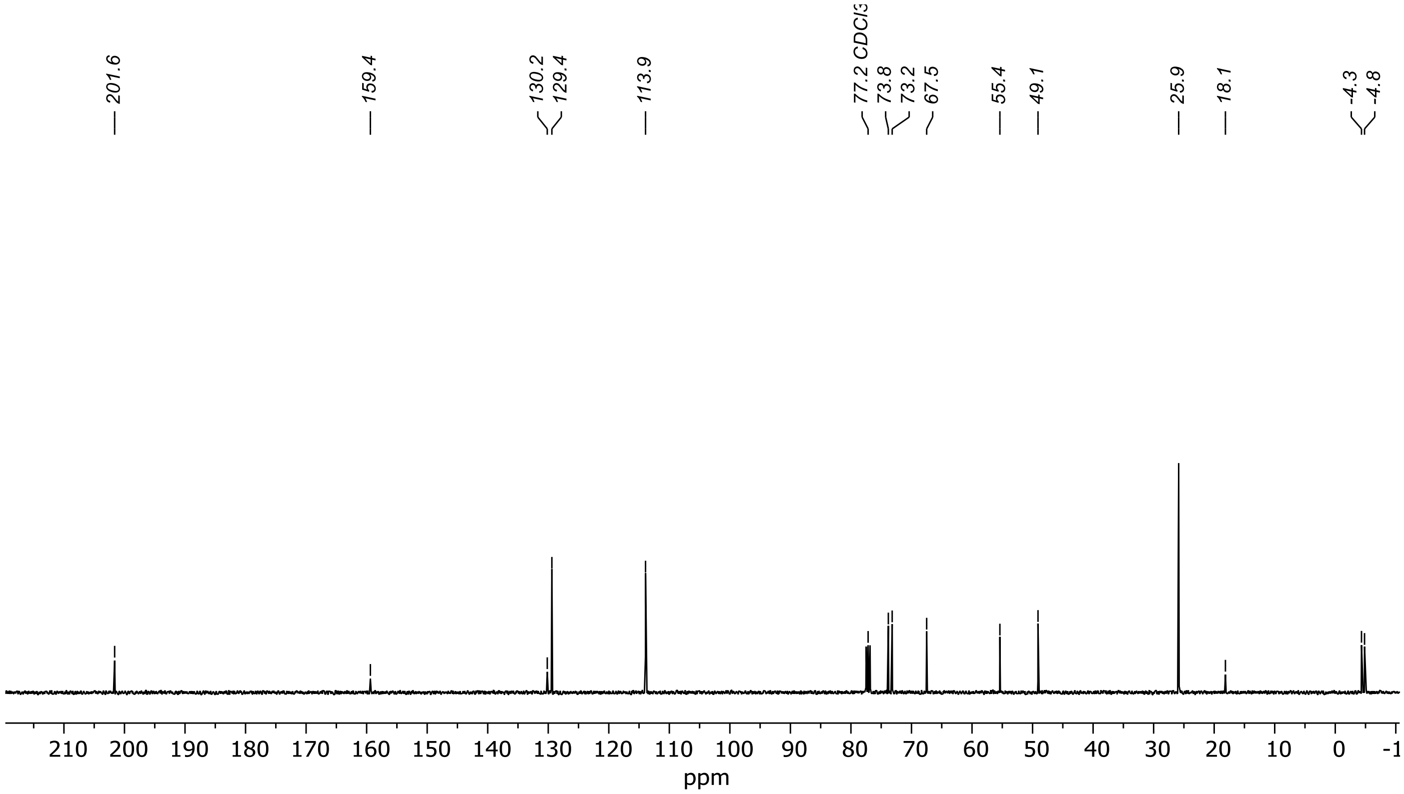

^{1}H-NMR (400.16 MHz, CDCl$_3$)

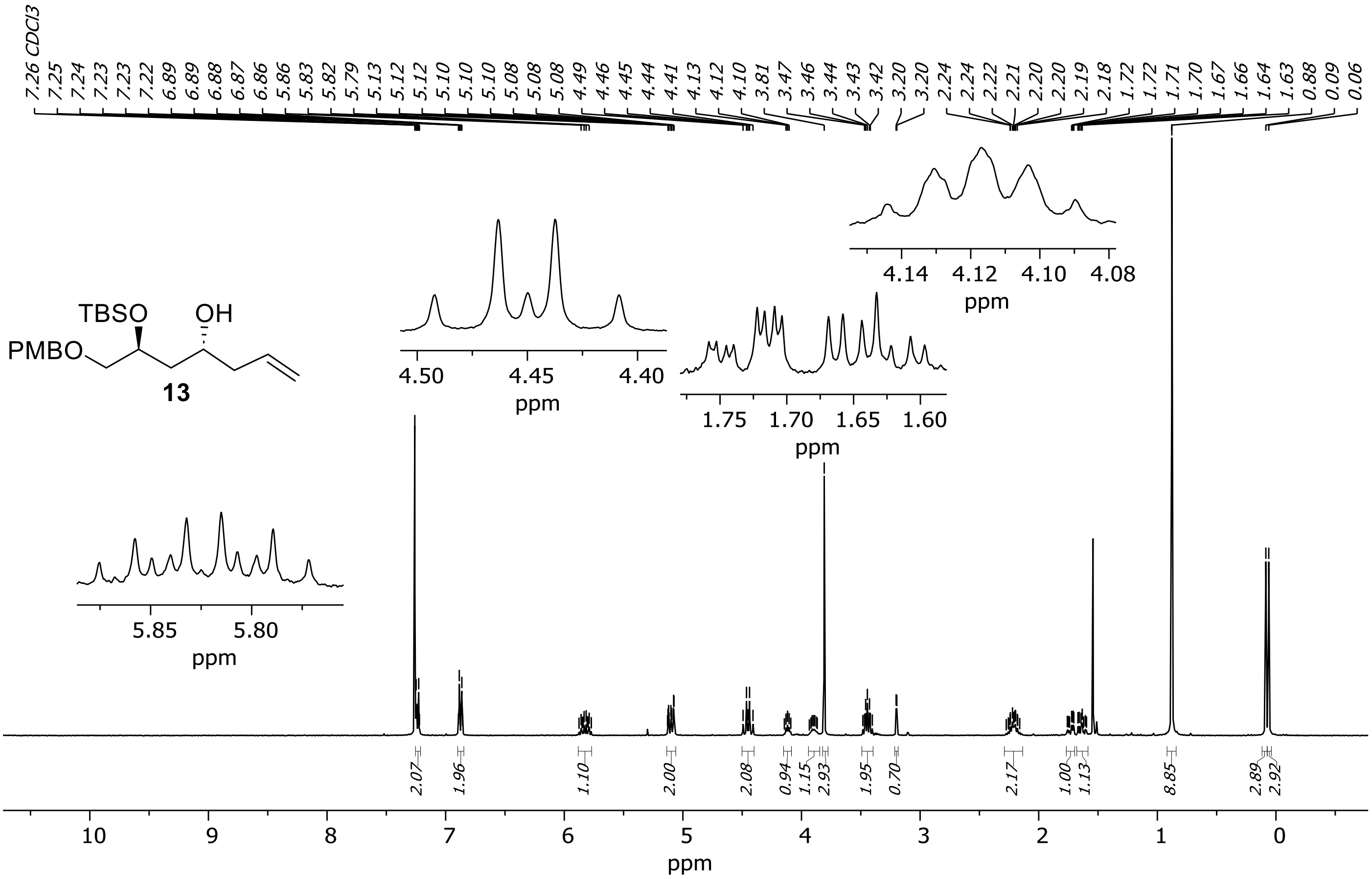

^{13}C-NMR (100.63 MHz, CDCl$_3$)

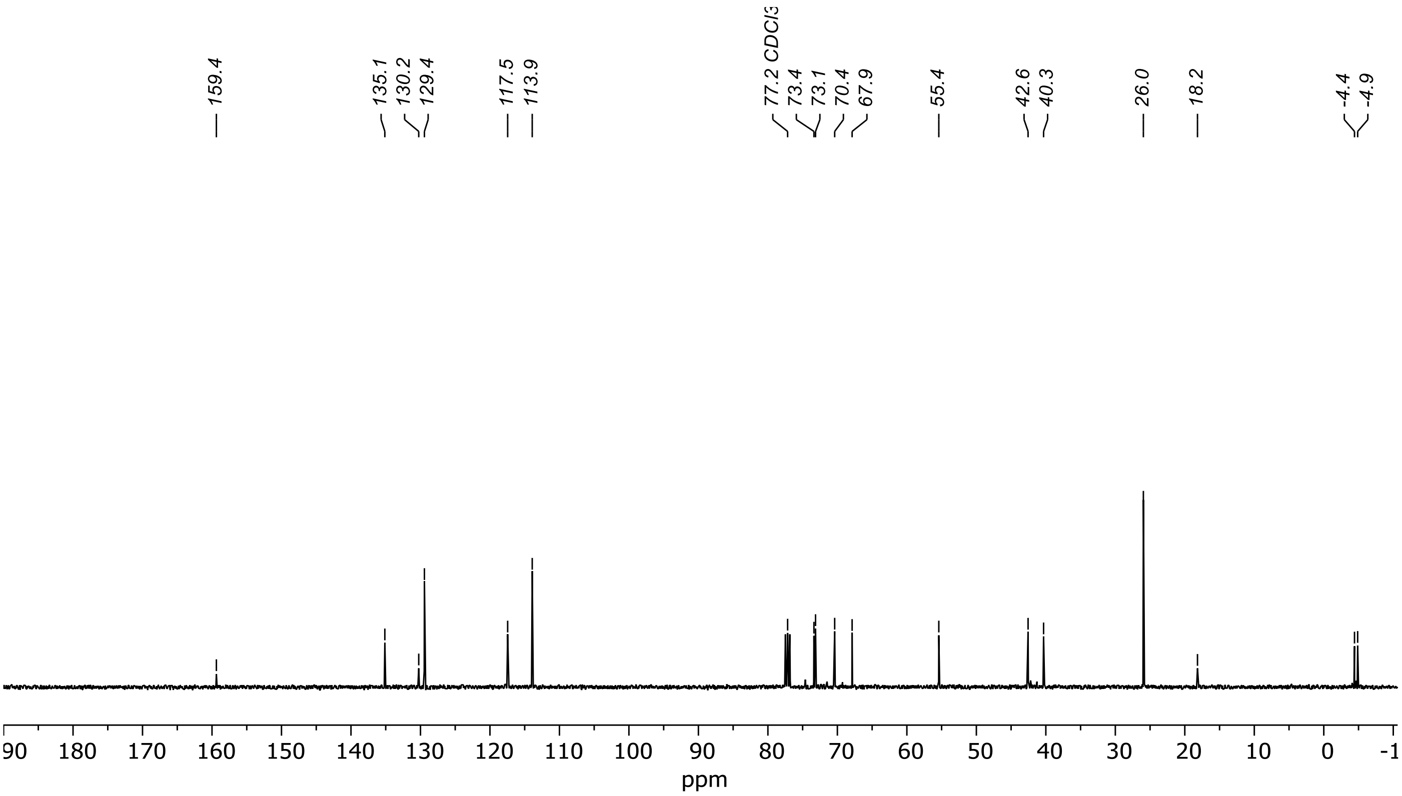

^{1}H-NMR (400.16 MHz, CDCl$_3$)

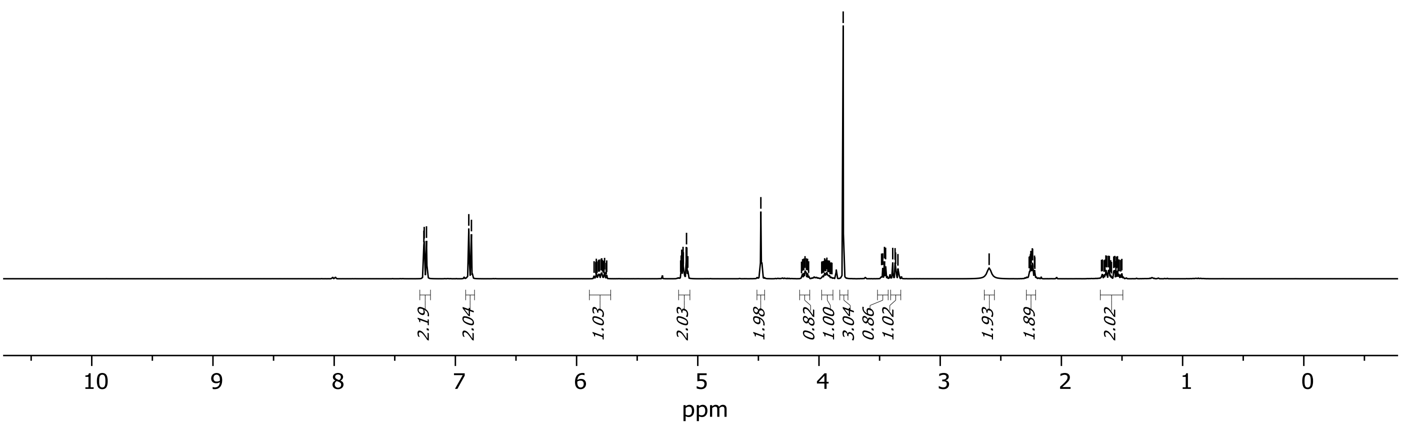

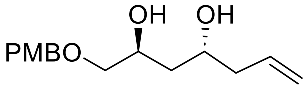

S12

^{13}C-NMR (100.63 MHz, CDCl$_3$)

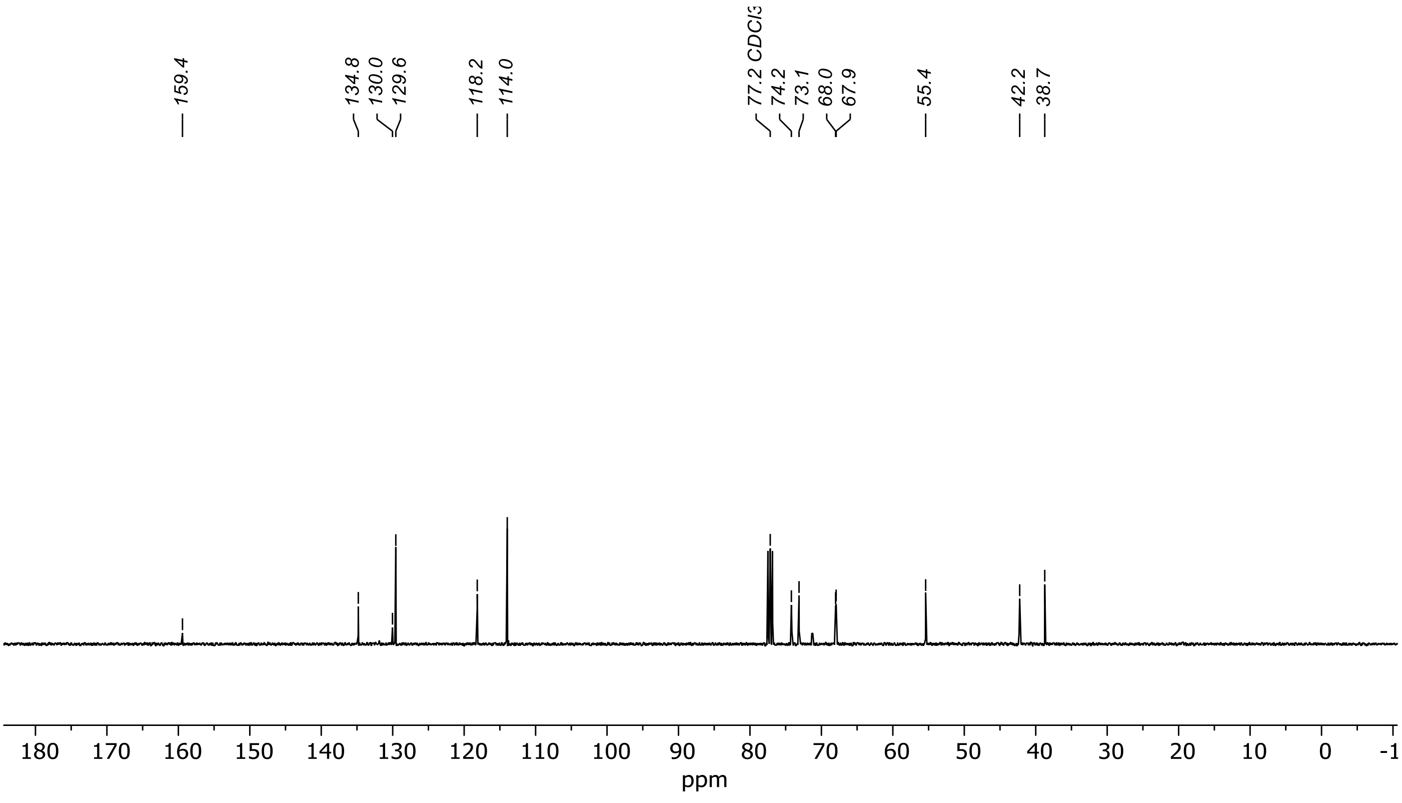

^{1}H-NMR (400.16 MHz, $CDCl_3$)

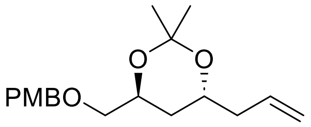

S11

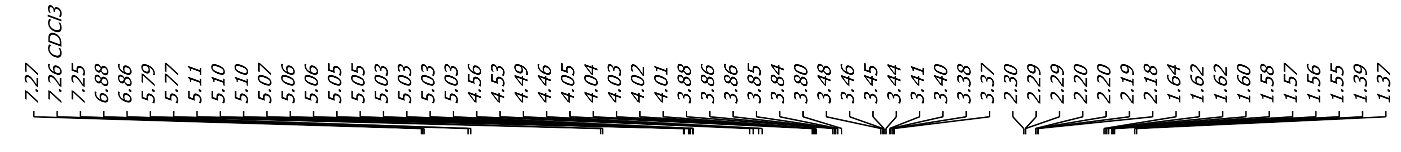
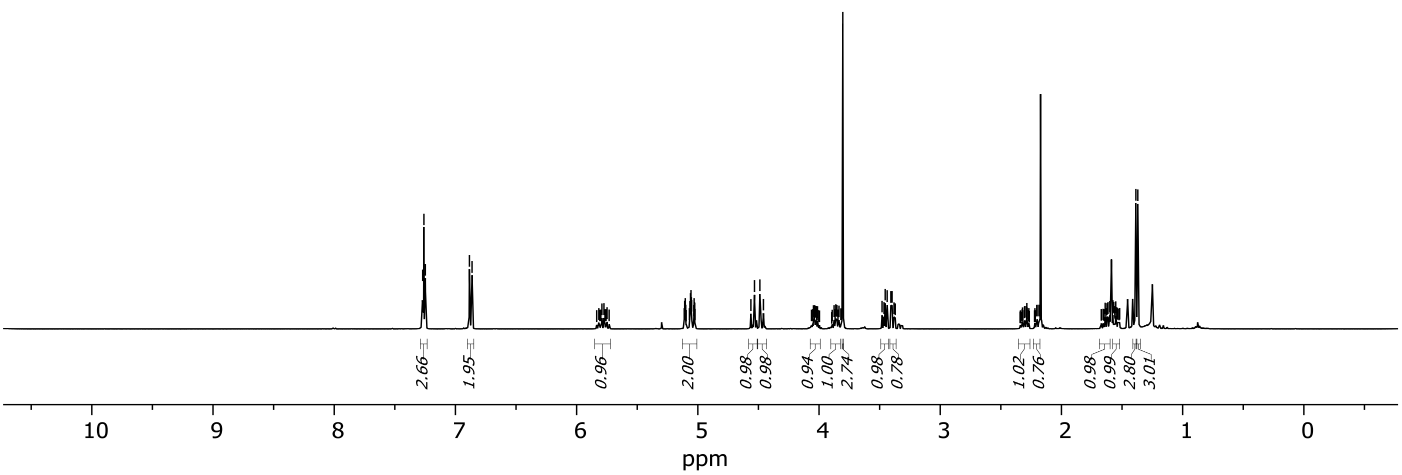

^{13}C-NMR (100.63 MHz, $CDCl_3$)

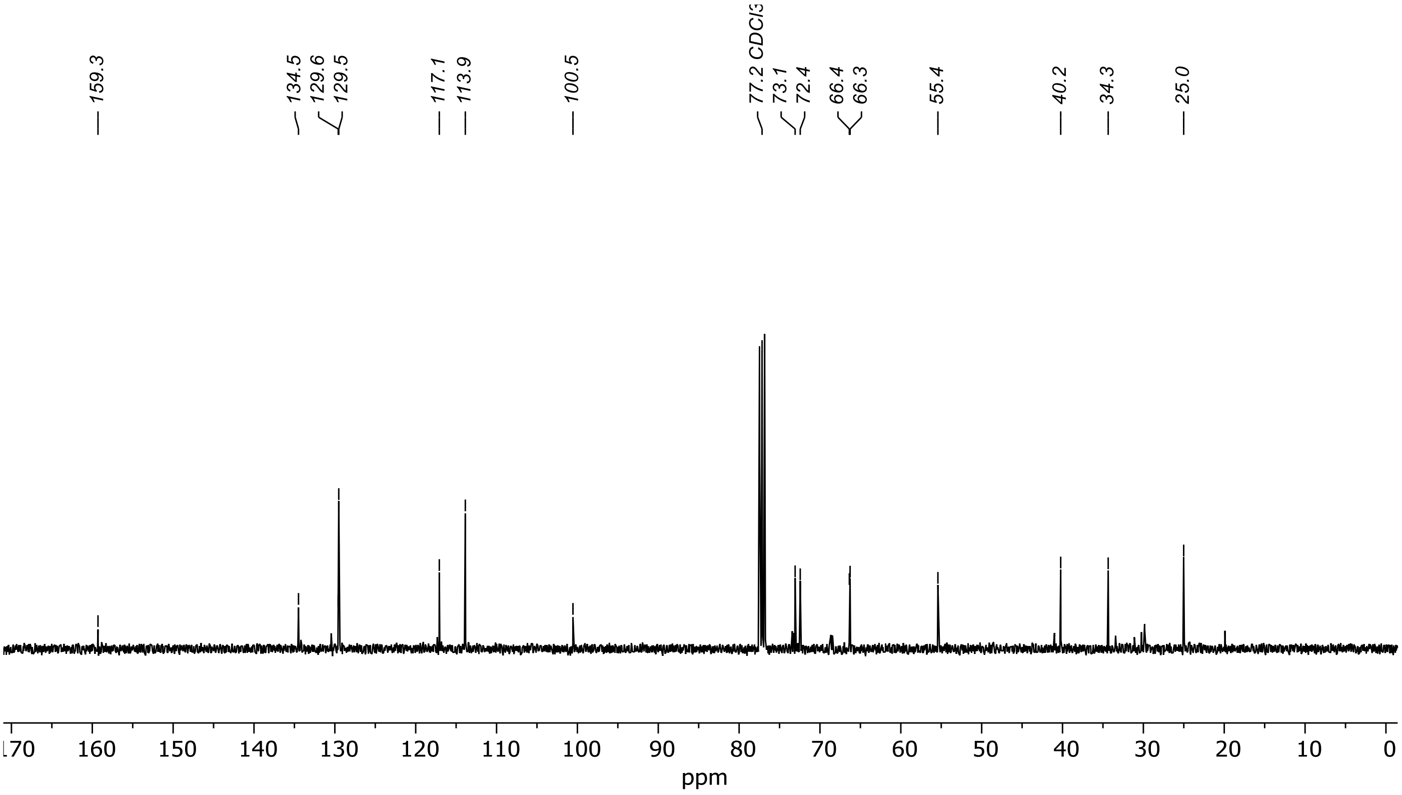

^{1}H-NMR (400.16 MHz, 323K $CDCl_3$)

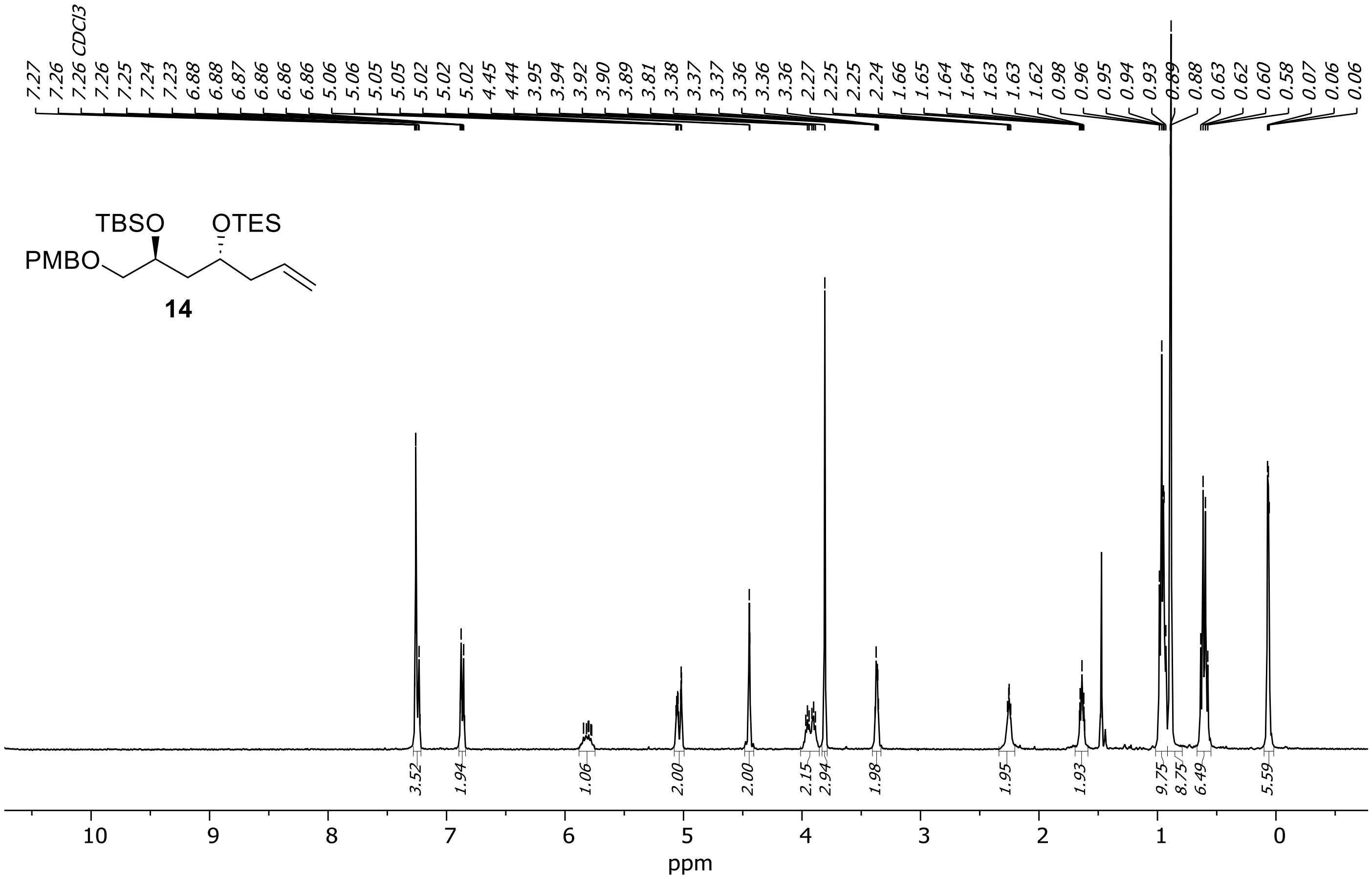

14

^{13}C-NMR (100.63 MHz, 323 K, $CDCl_3$)

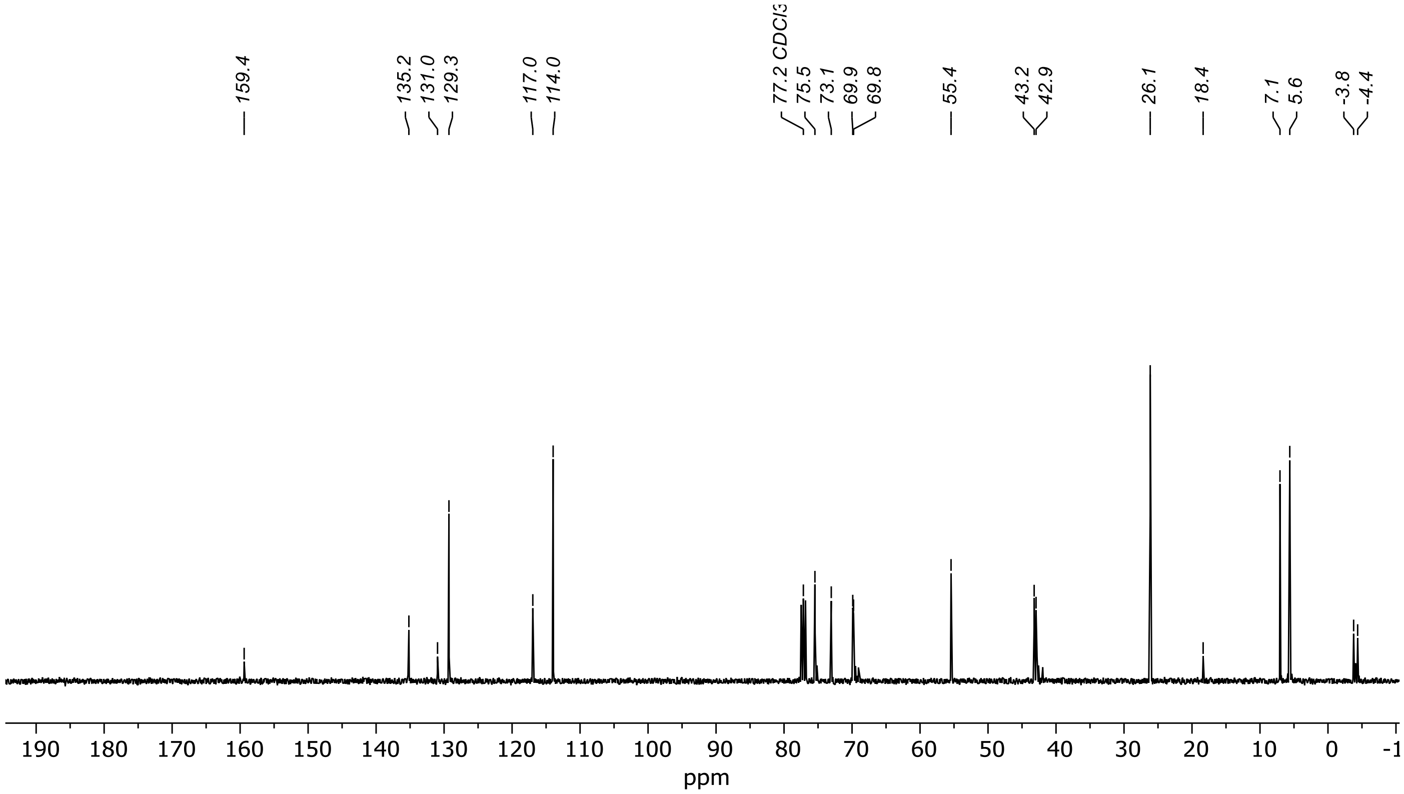

^{1}H-NMR (400.16 MHz, CDCl$_3$)

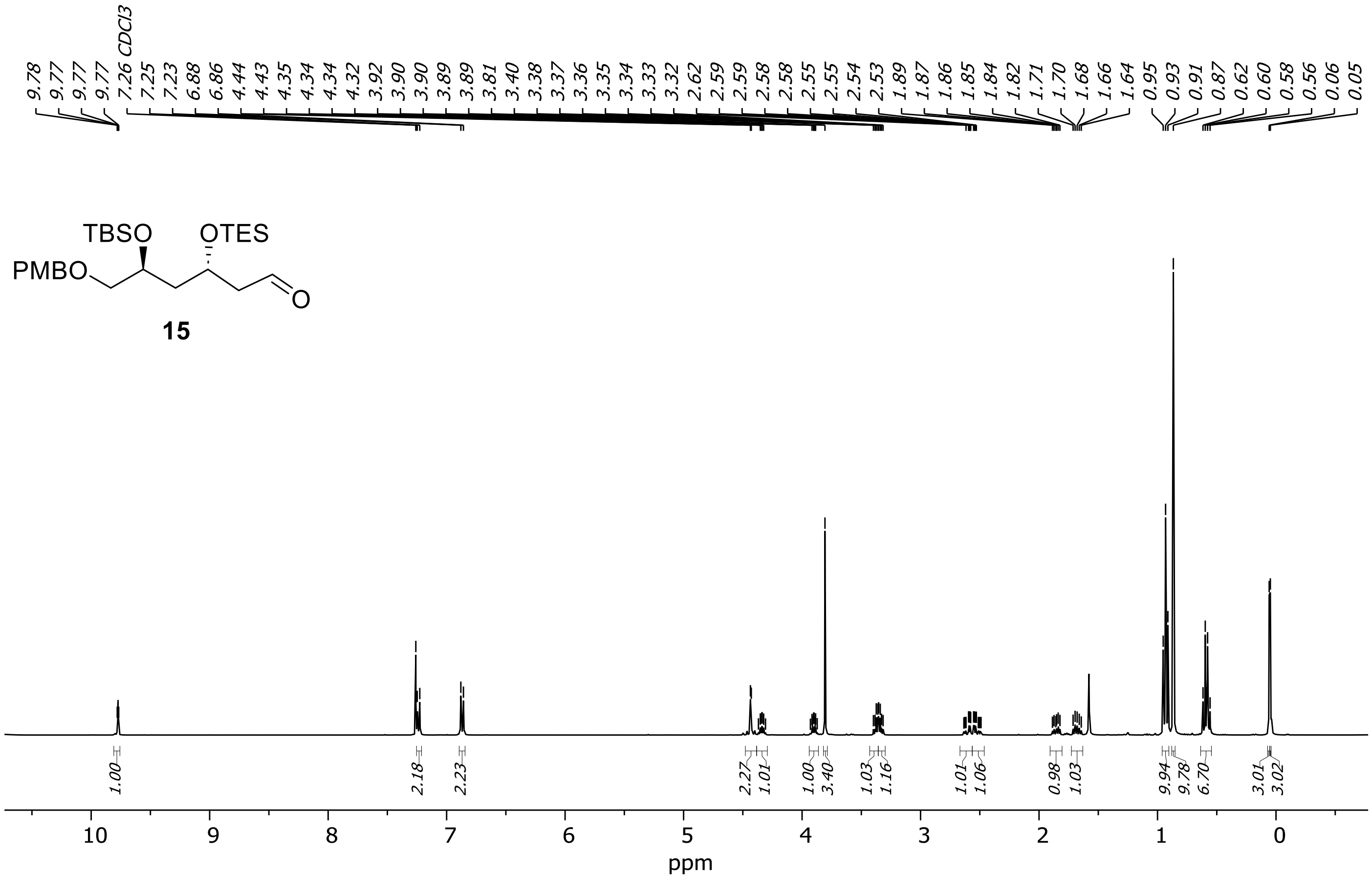

^{13}C-NMR (100.63 MHz, CDCl$_3$)

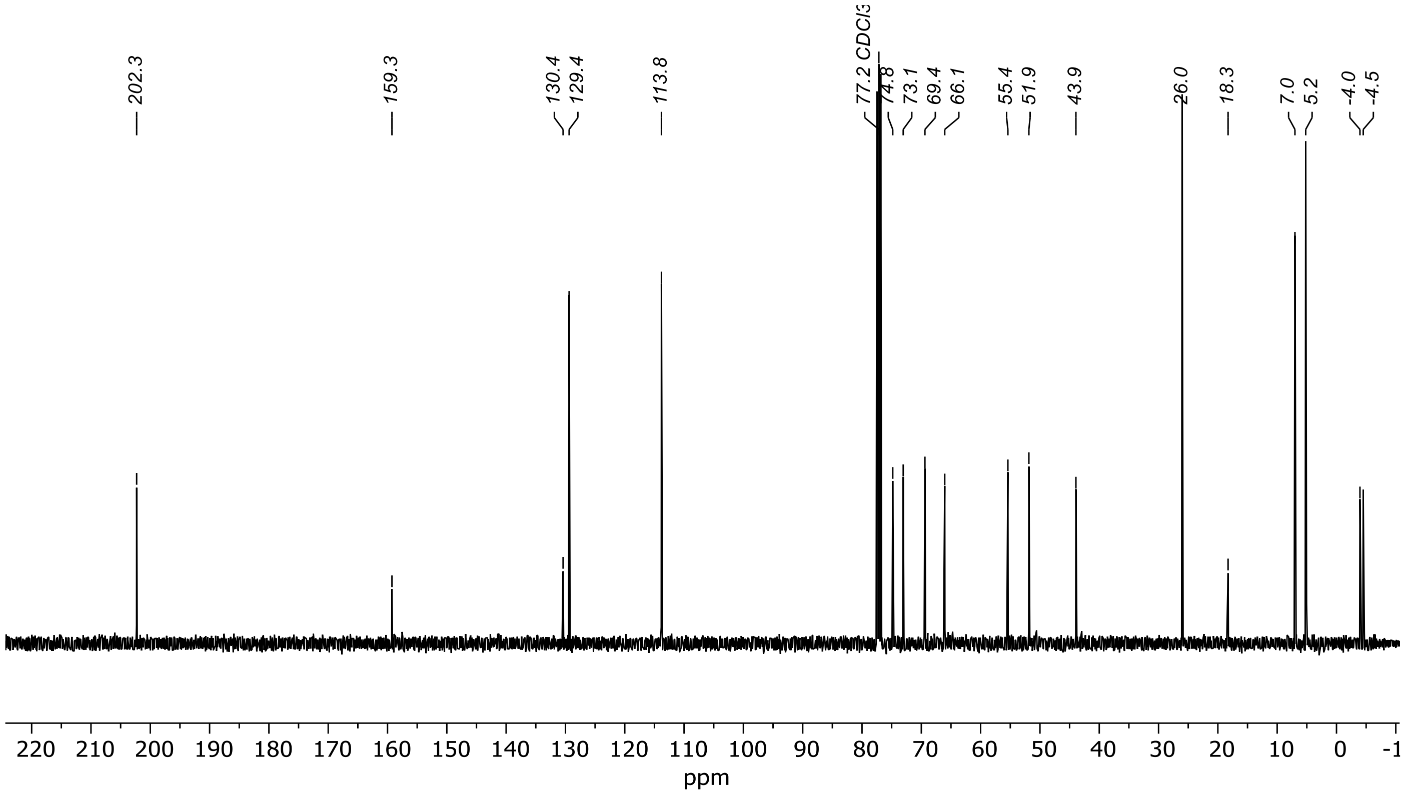

^{1}H-NMR (400.16 MHz, C_6D_6)

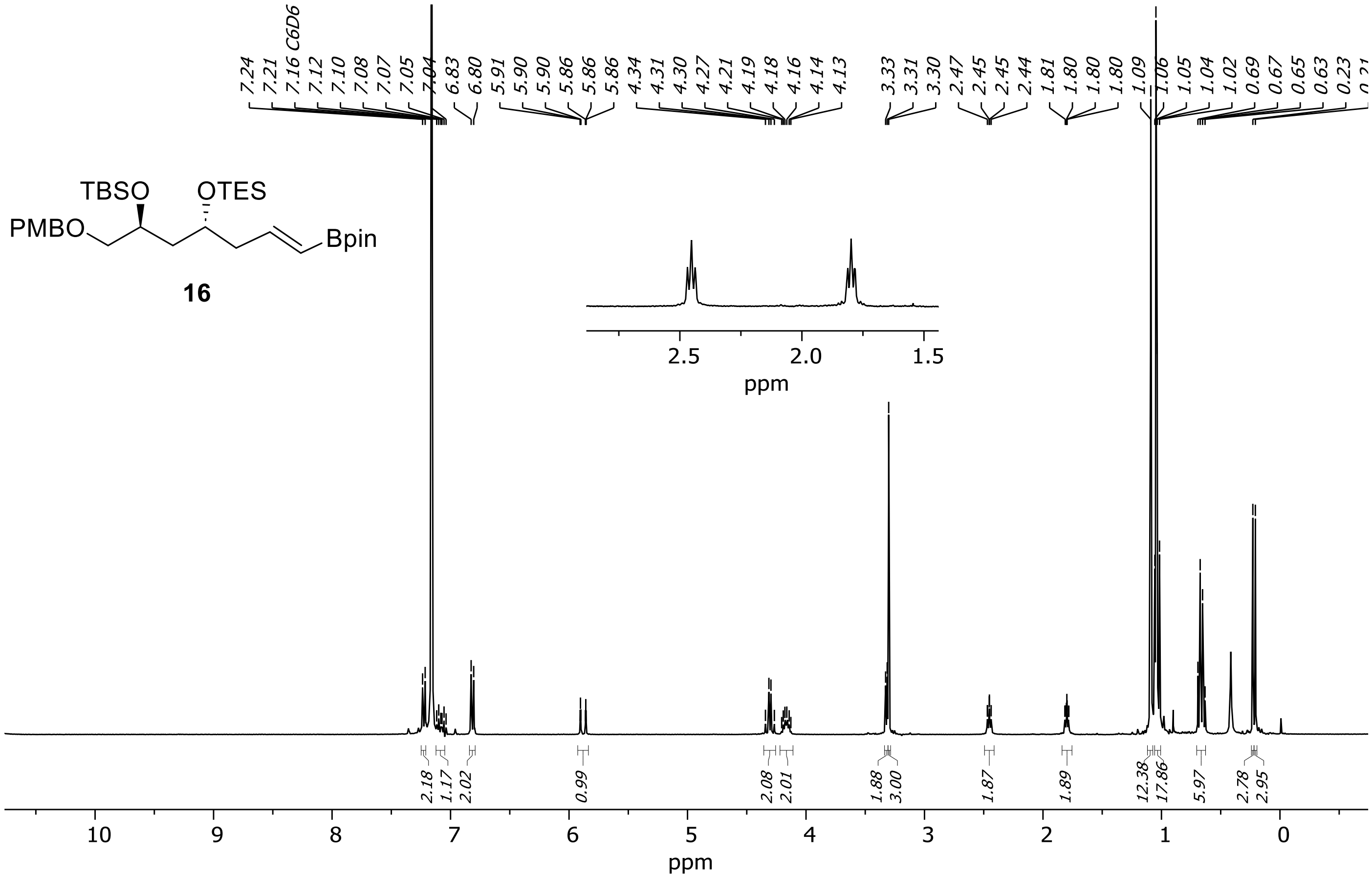

^{13}C-NMR (100.63 MHz, C_6D_6)

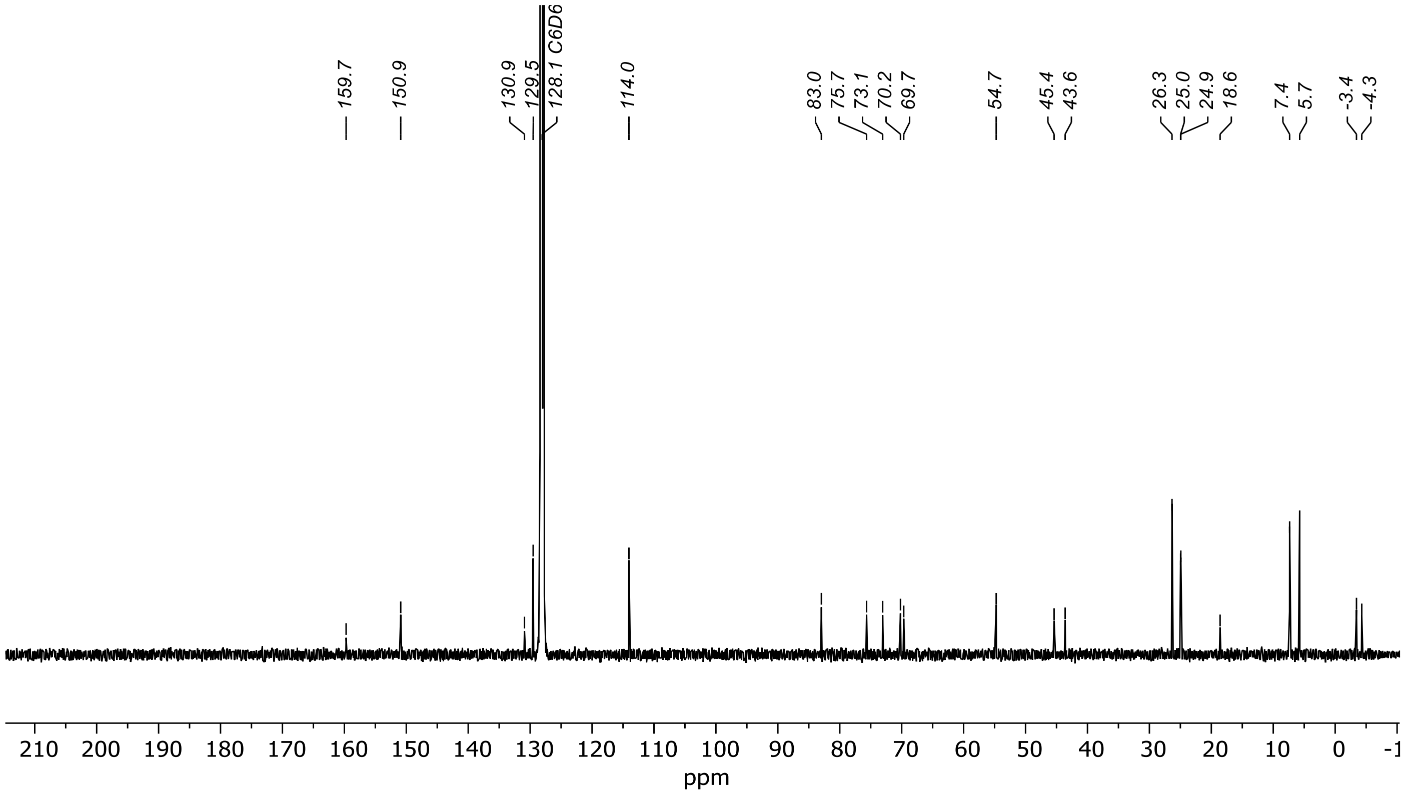

^{1}H-NMR (400.16 MHz, C_6D_6)

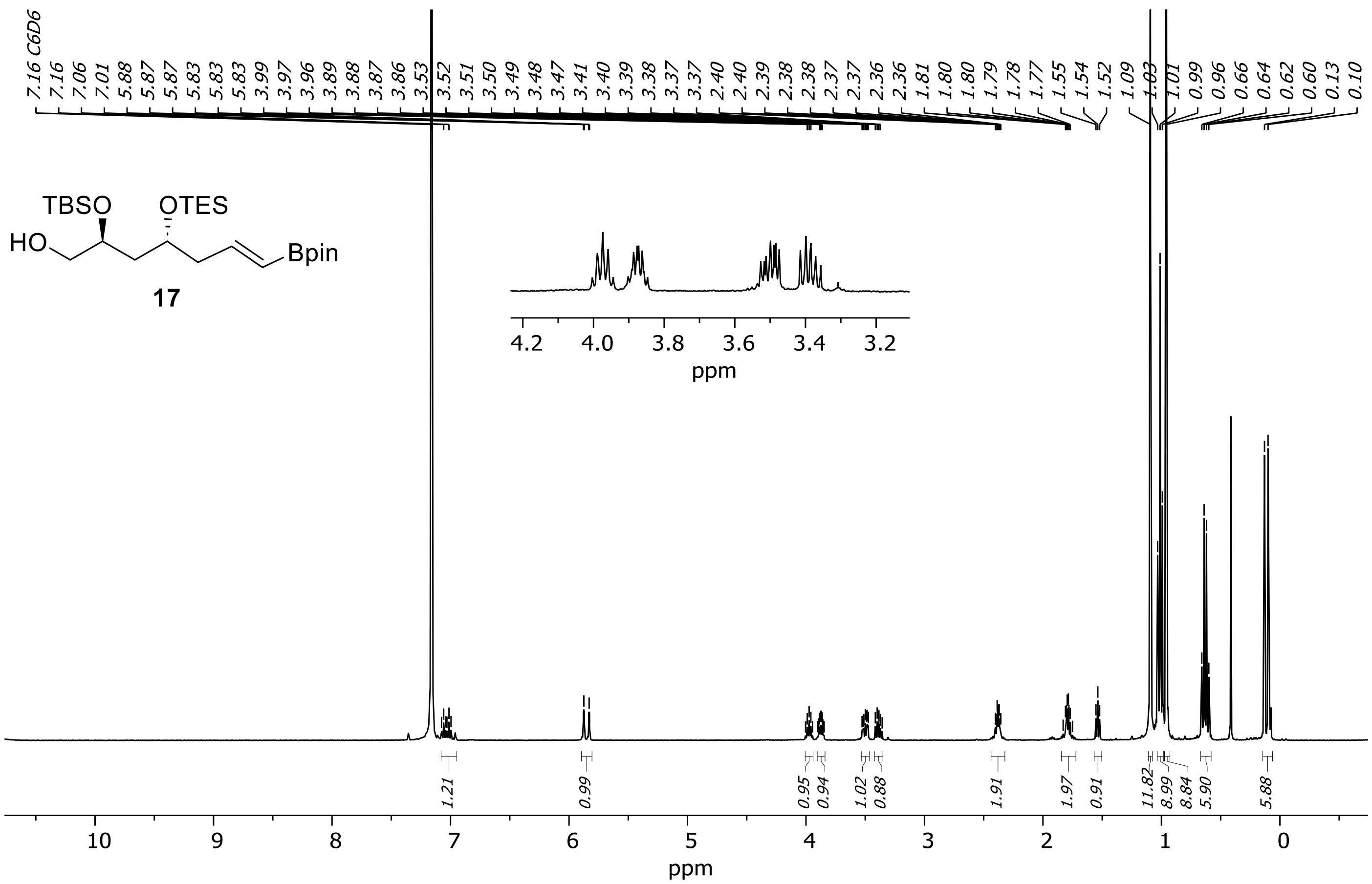

^{13}C-NMR (100.63 MHz, C_6D_6)

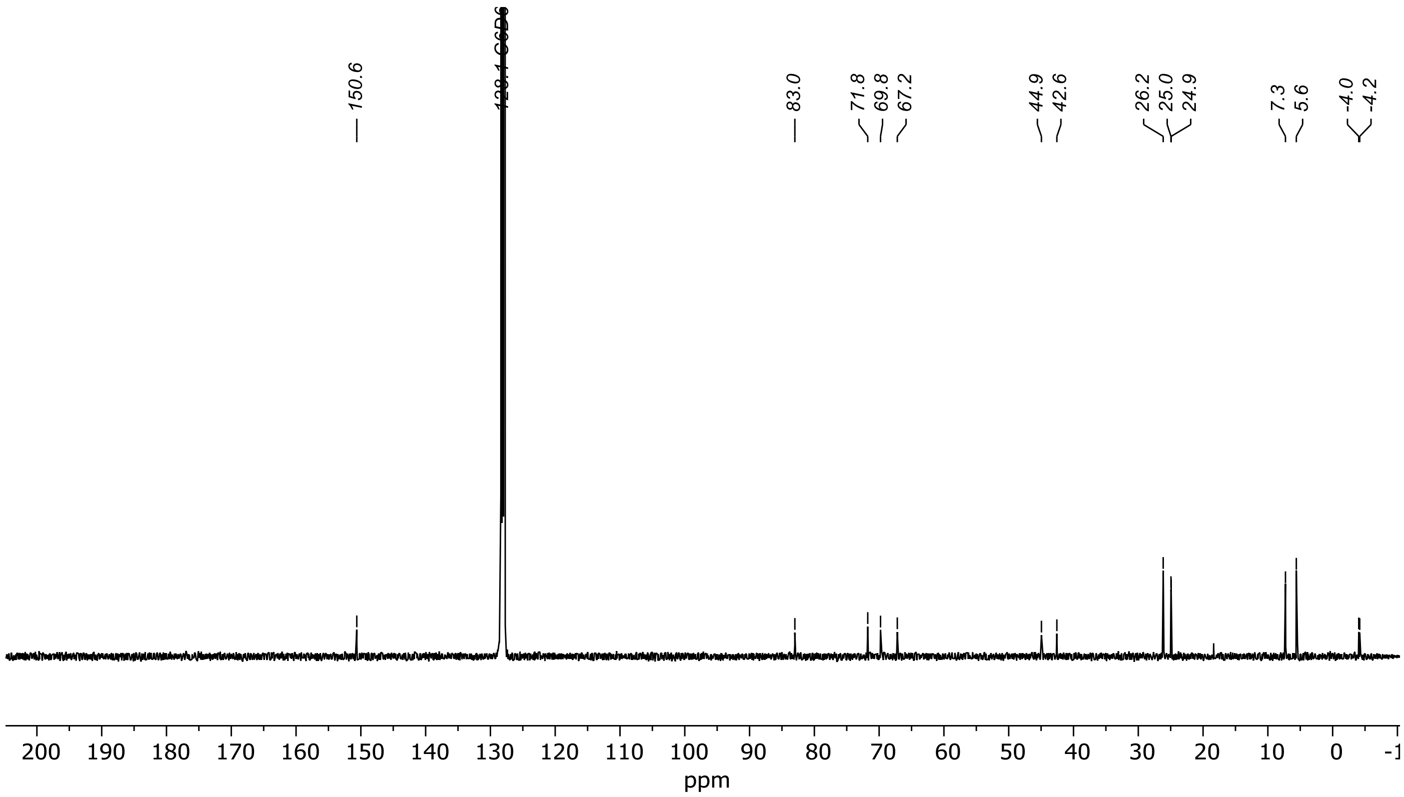

^{1}H-NMR (400.16 MHz, C_6D_6)

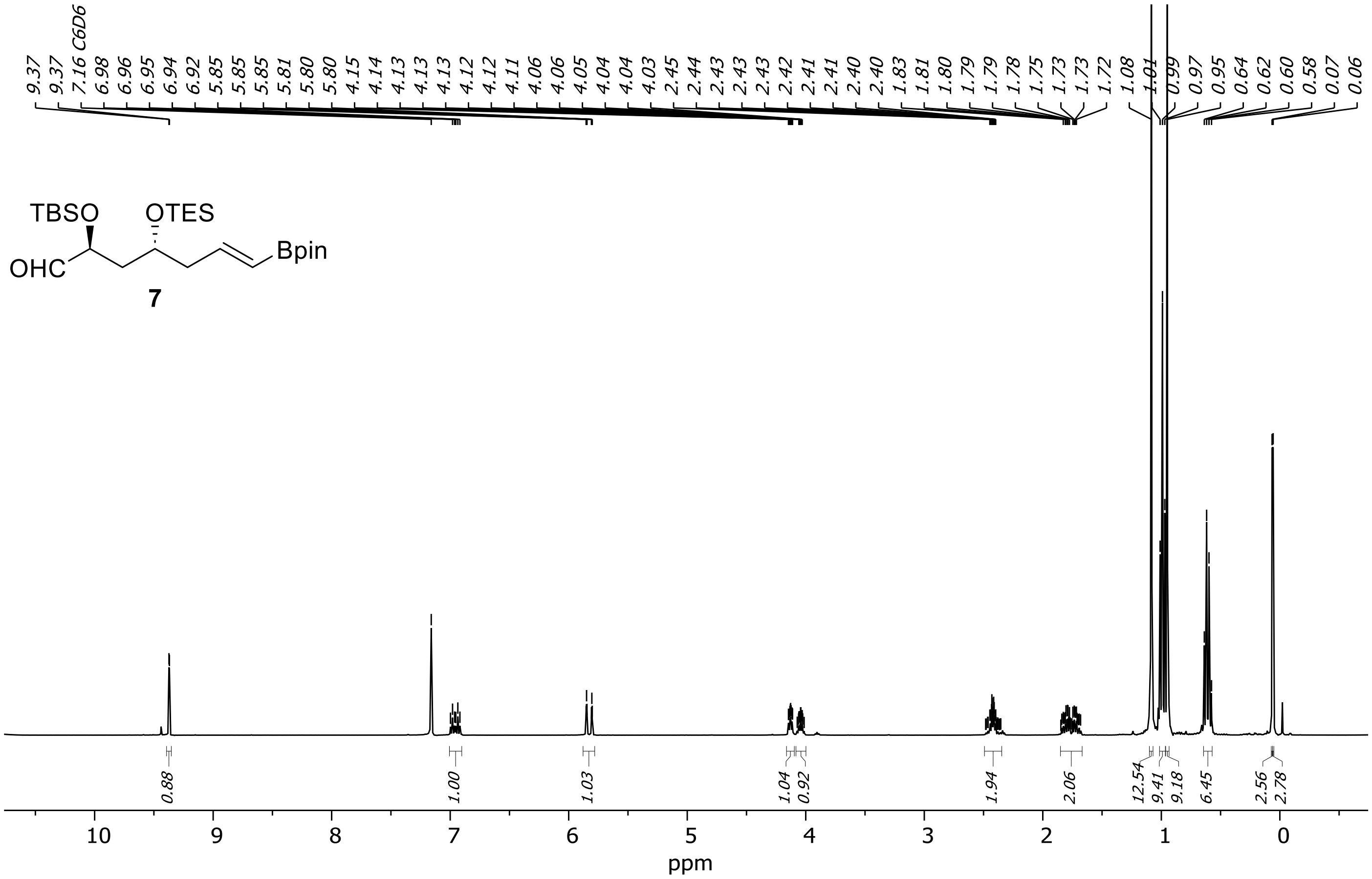

^{13}C-NMR (100.63 MHz, C_6D_6)

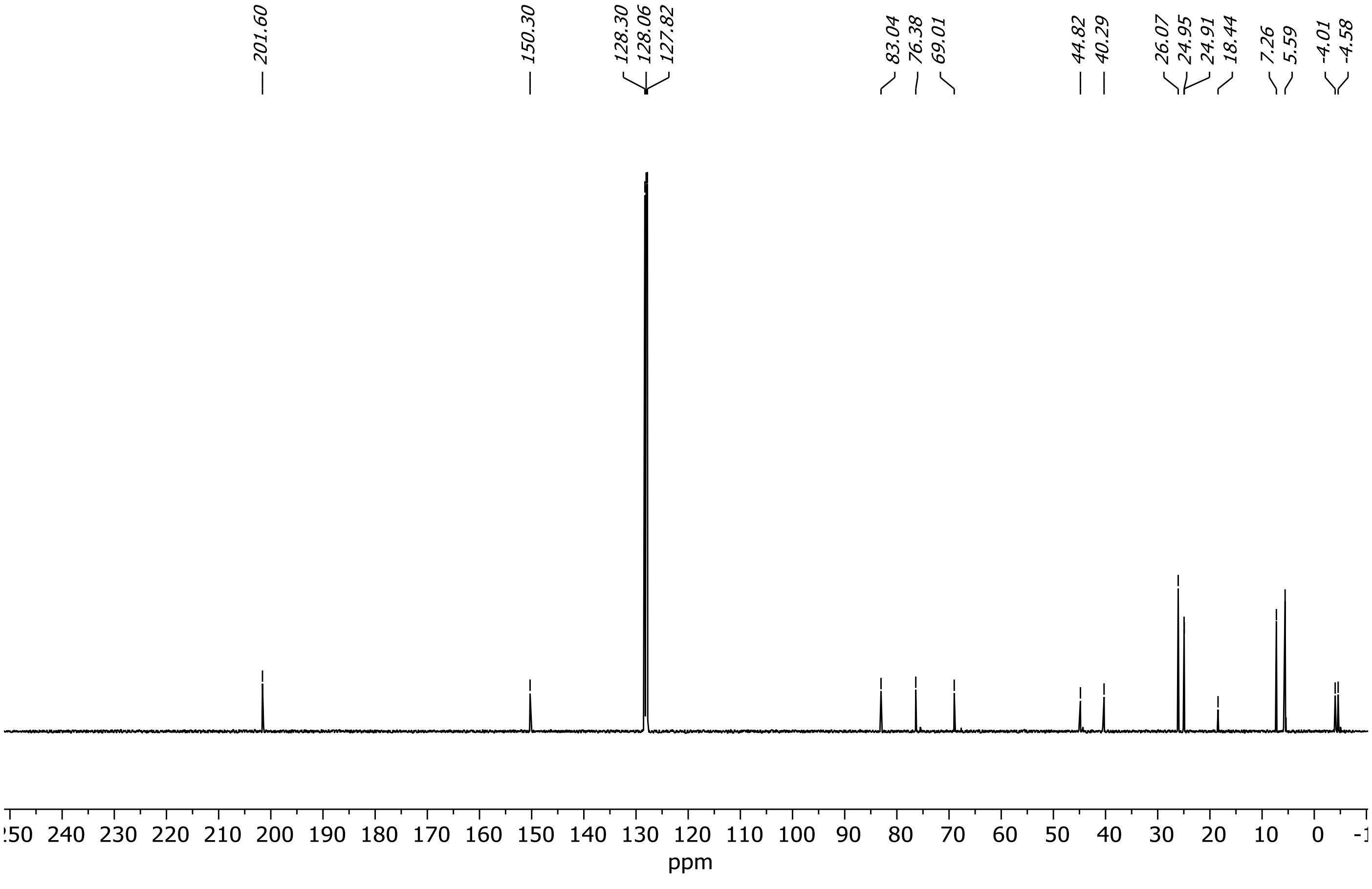

^{1}H-NMR (400.16 MHz, $CDCl_3$)

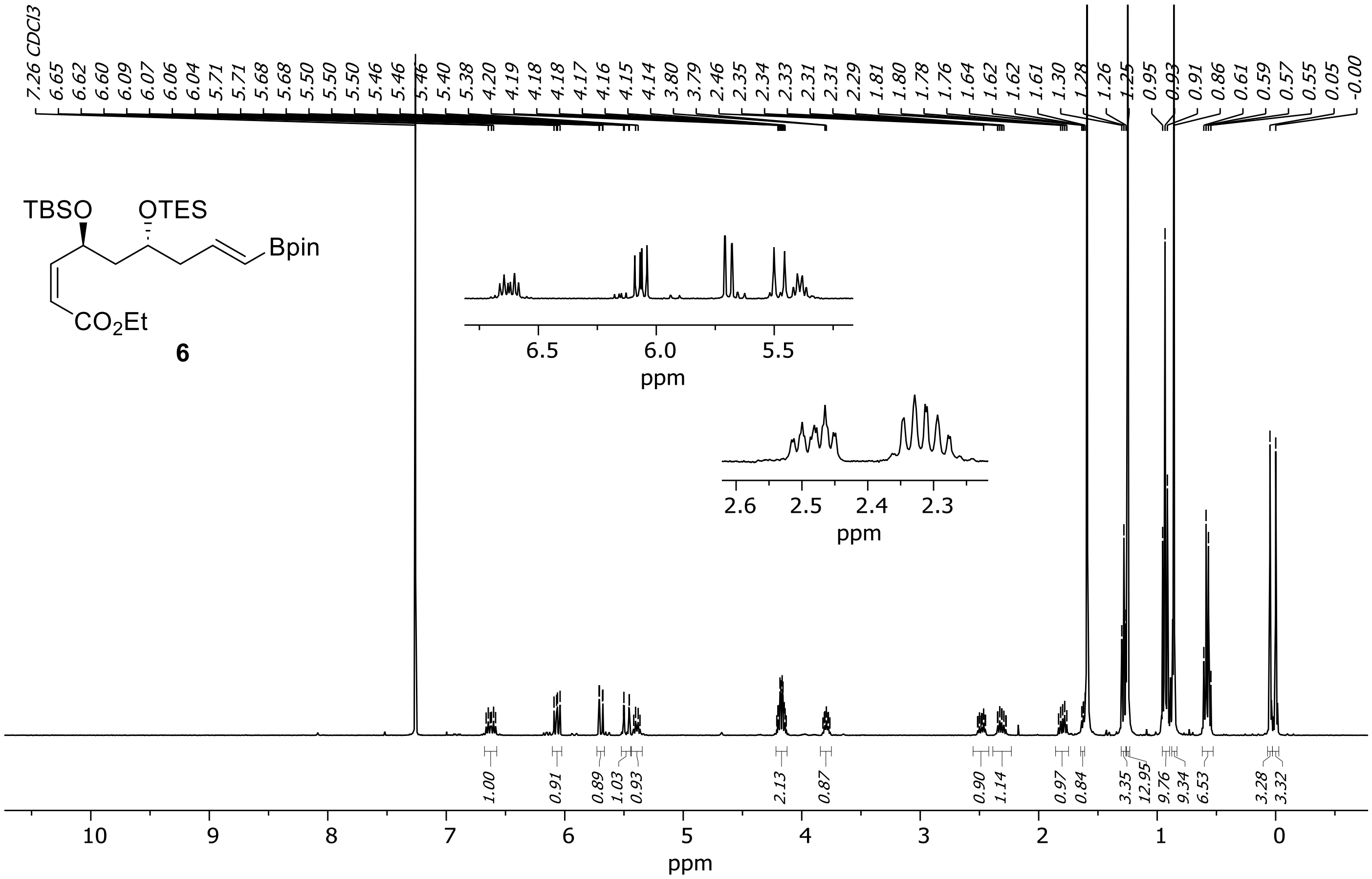

^{13}C-NMR (100.63 MHz, $CDCl_3$)

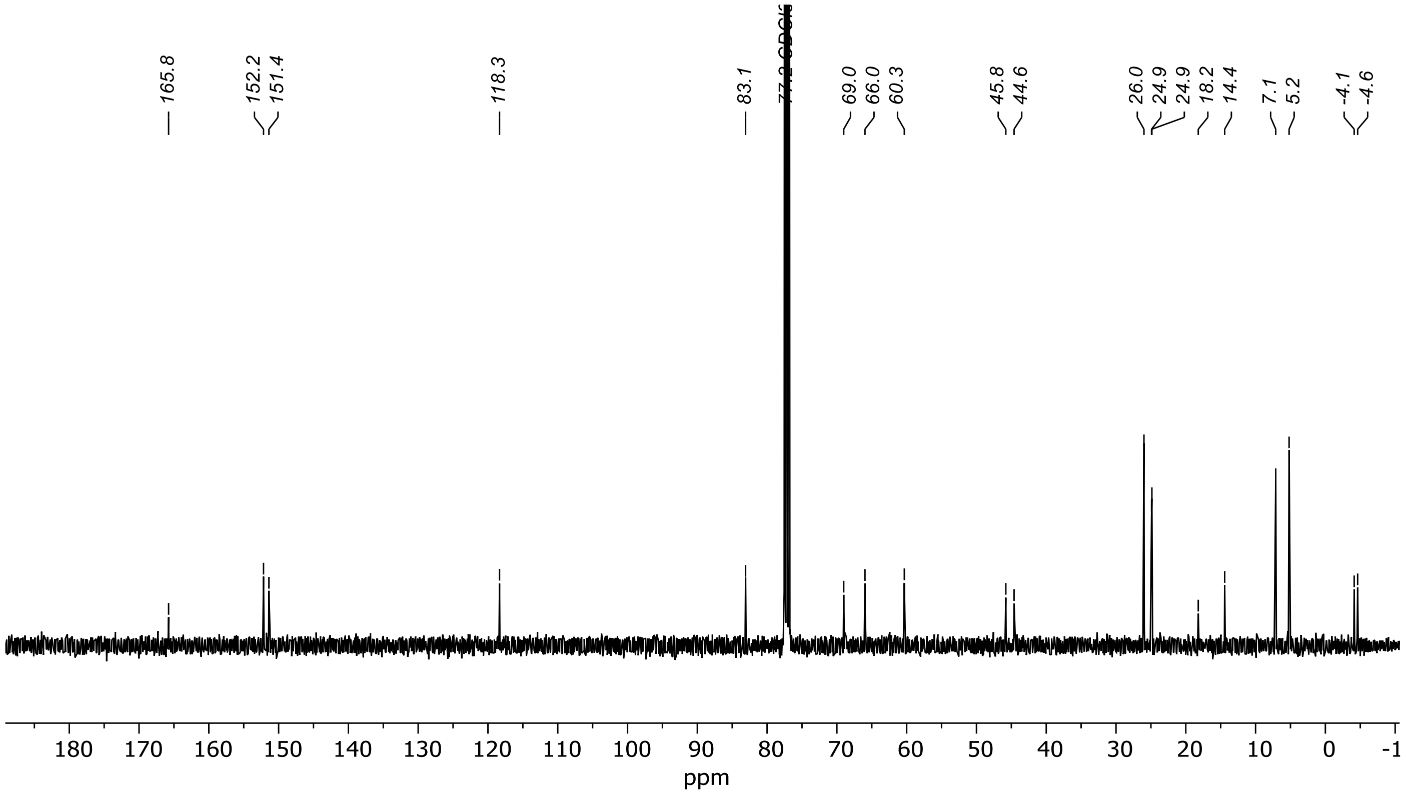

^{1}H-NMR (400.16 MHz, C_6D_6)

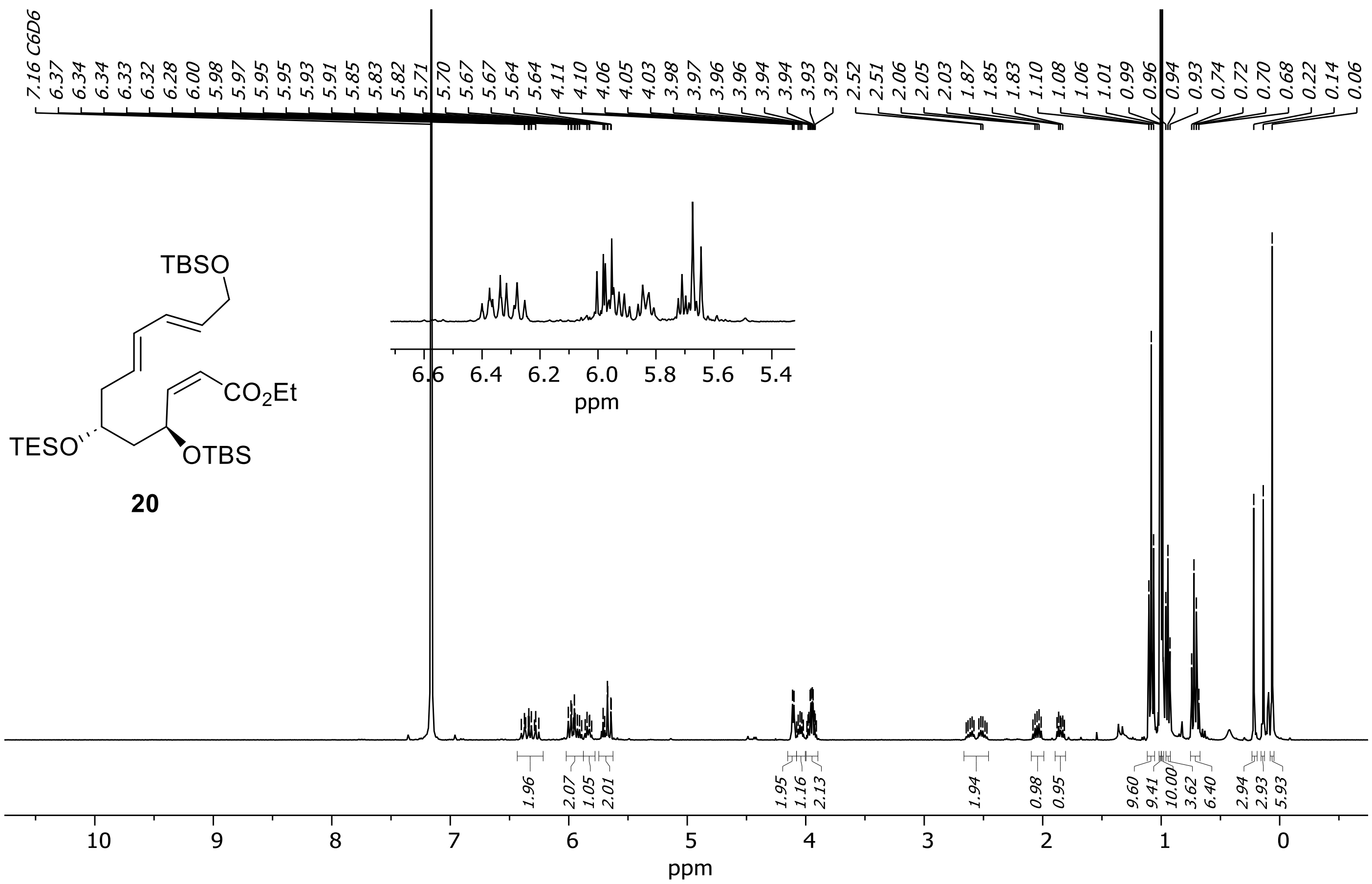

^{13}C-NMR (100.63 MHz, C_6D_6)

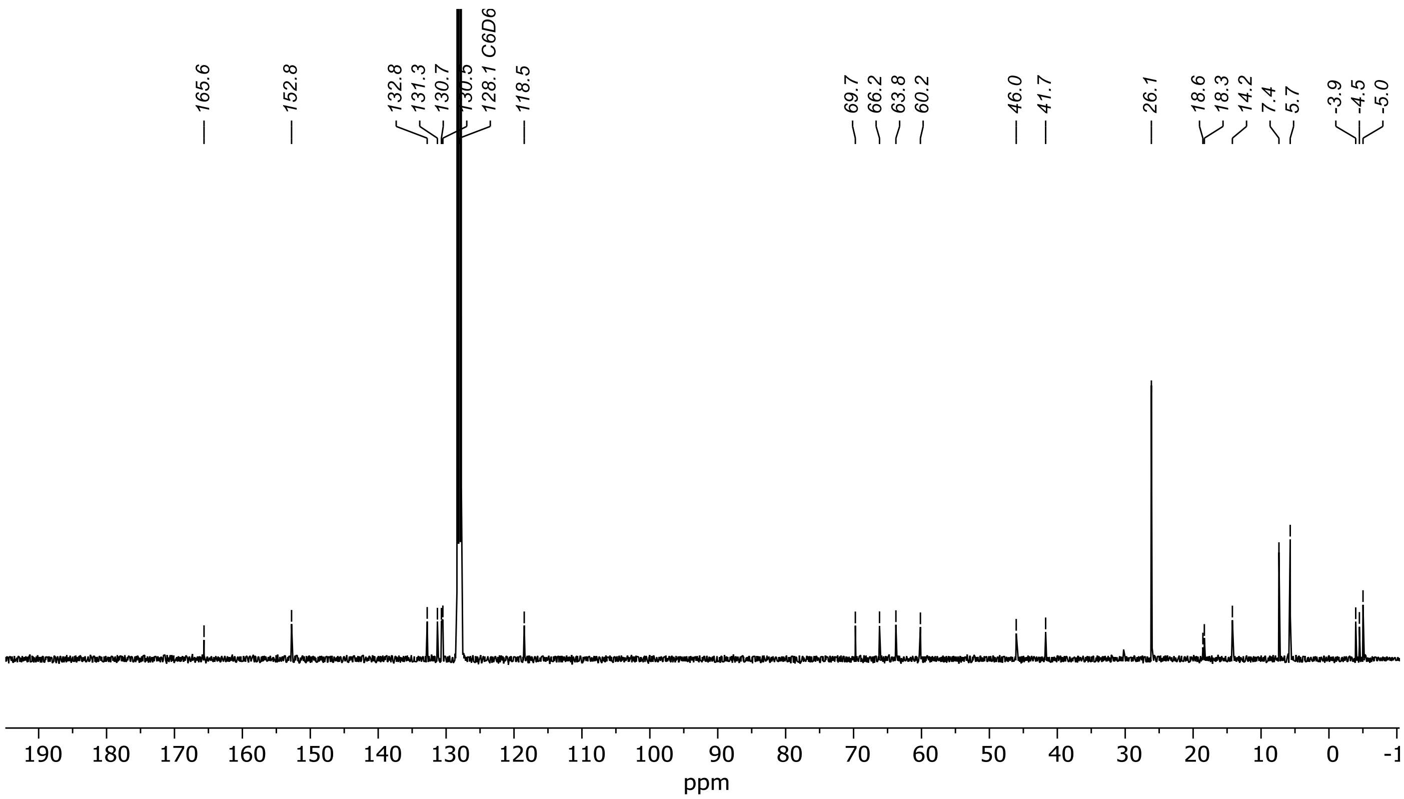

^{1}H-NMR (400.16 MHz, C_6D_6)

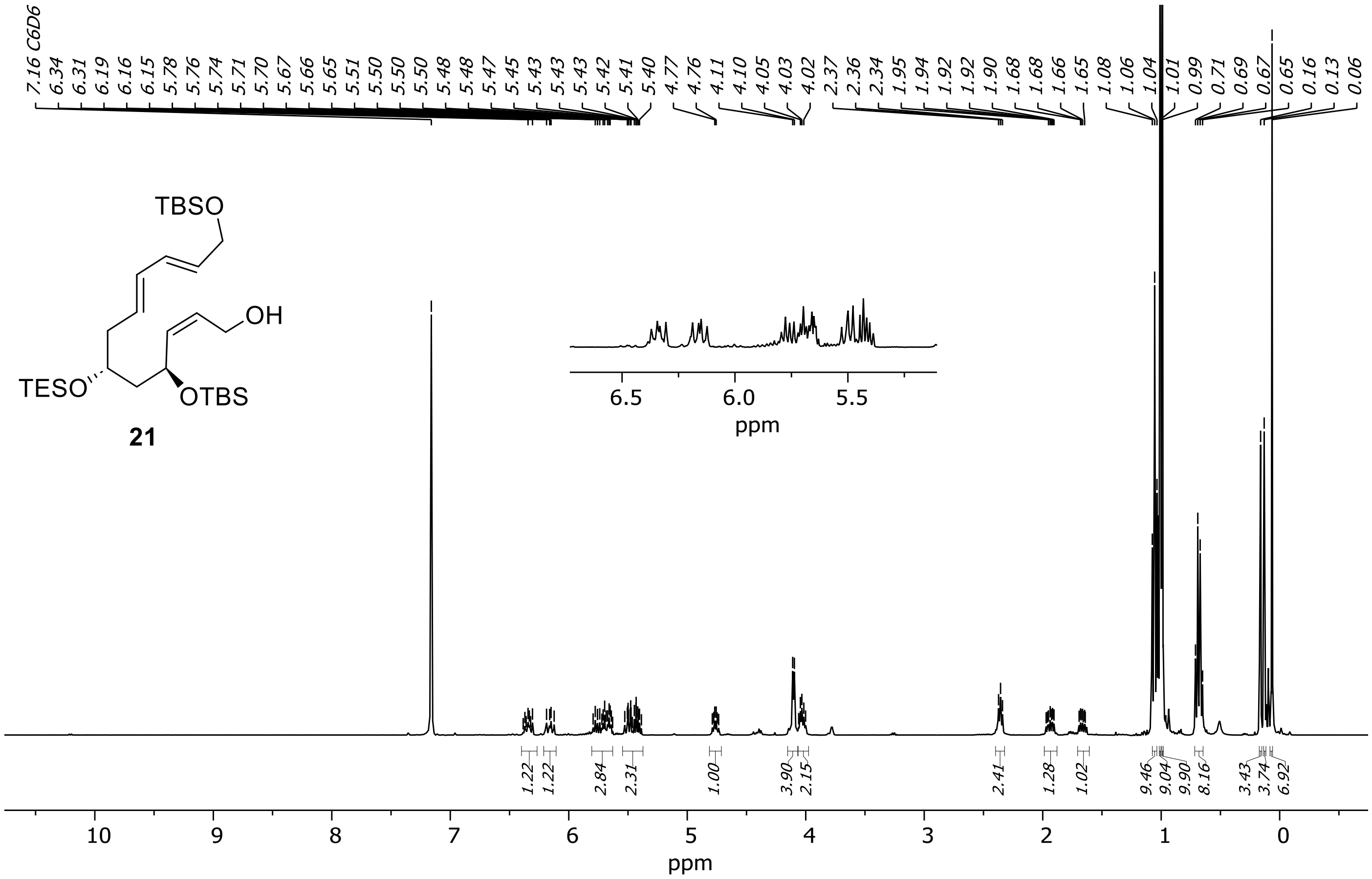

^{13}C-NMR (100.63 MHz, C_6D_6)

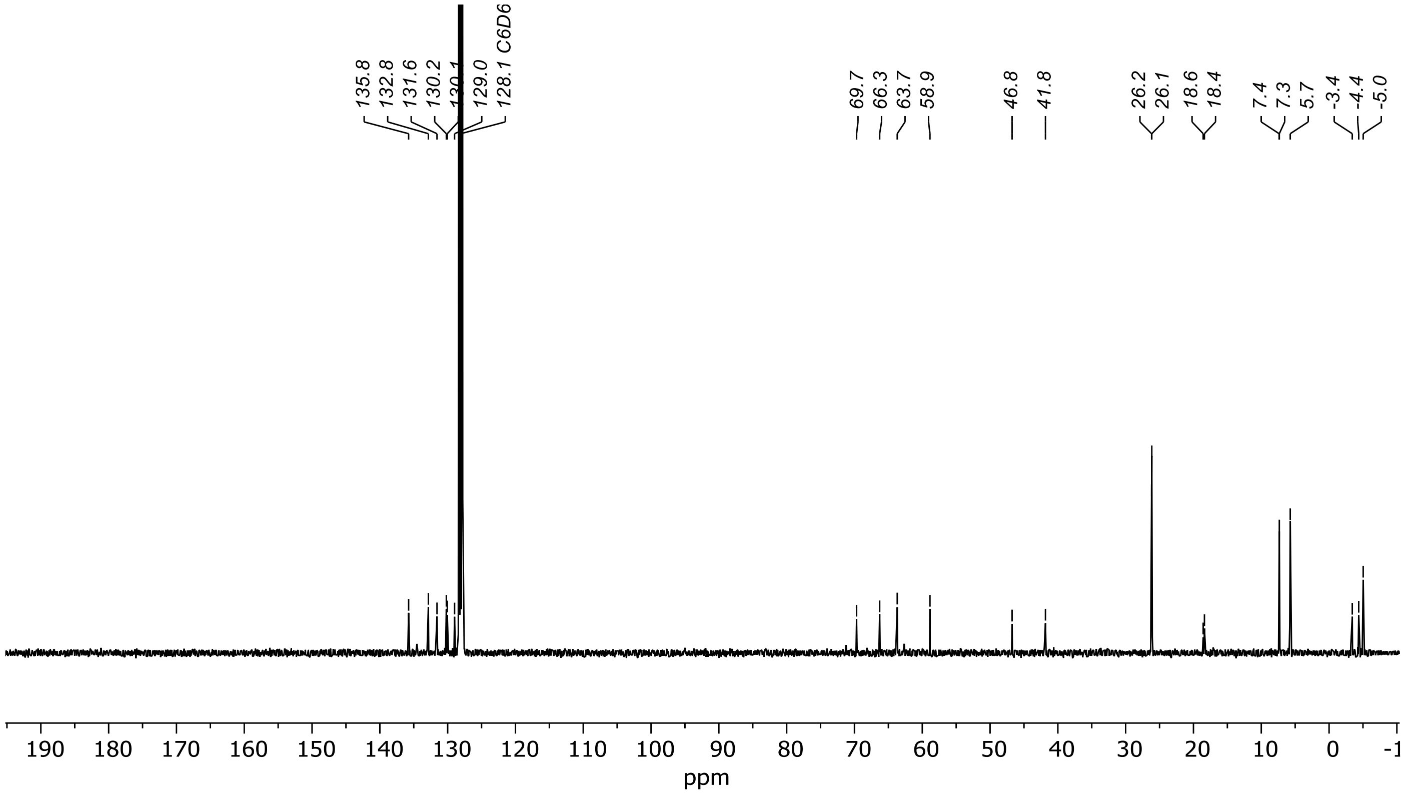

^{1}H-NMR (400.16 MHz, $CDCl_3$)

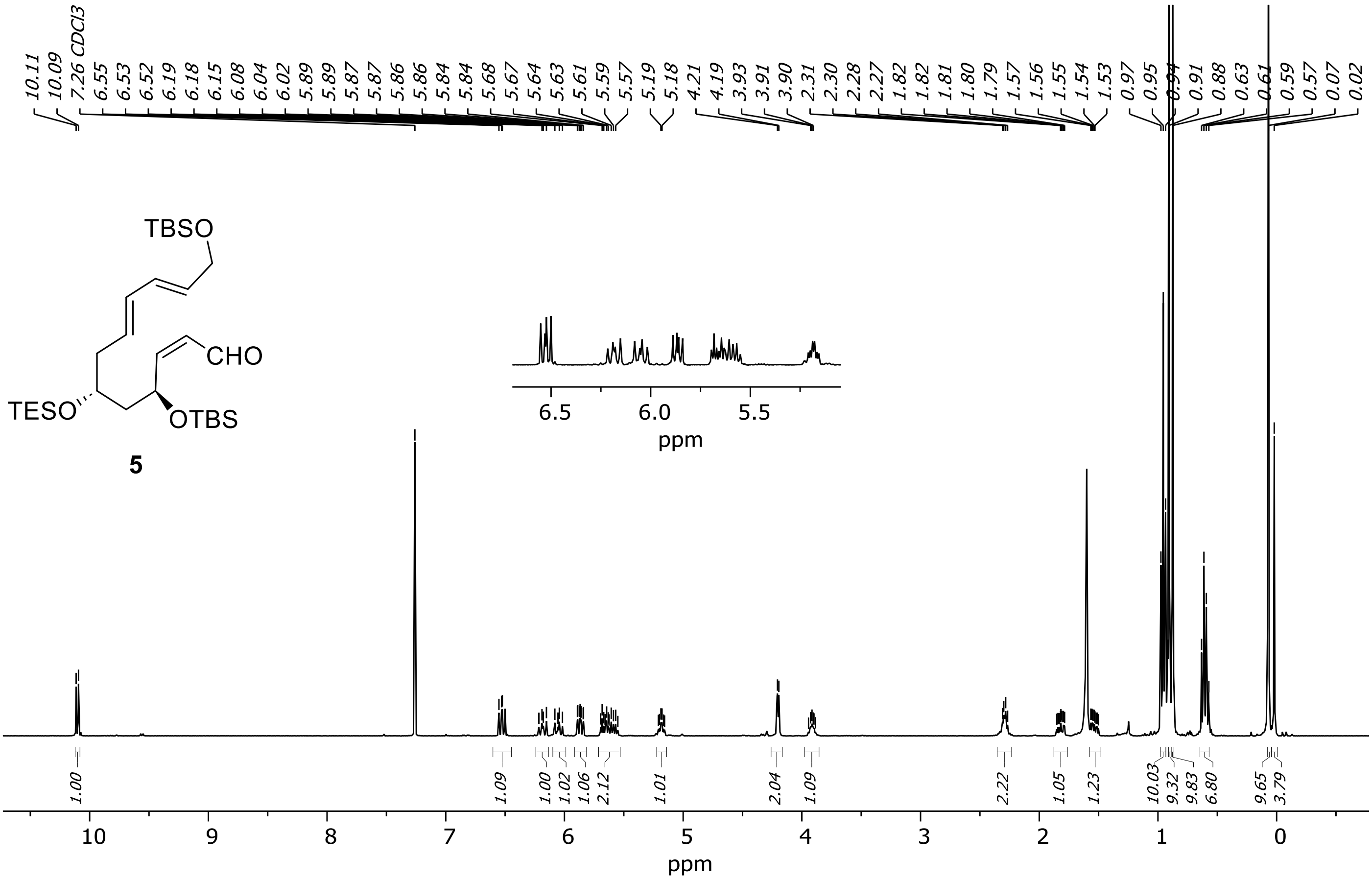

5

^{13}C-NMR (100.63 MHz, $CDCl_3$)

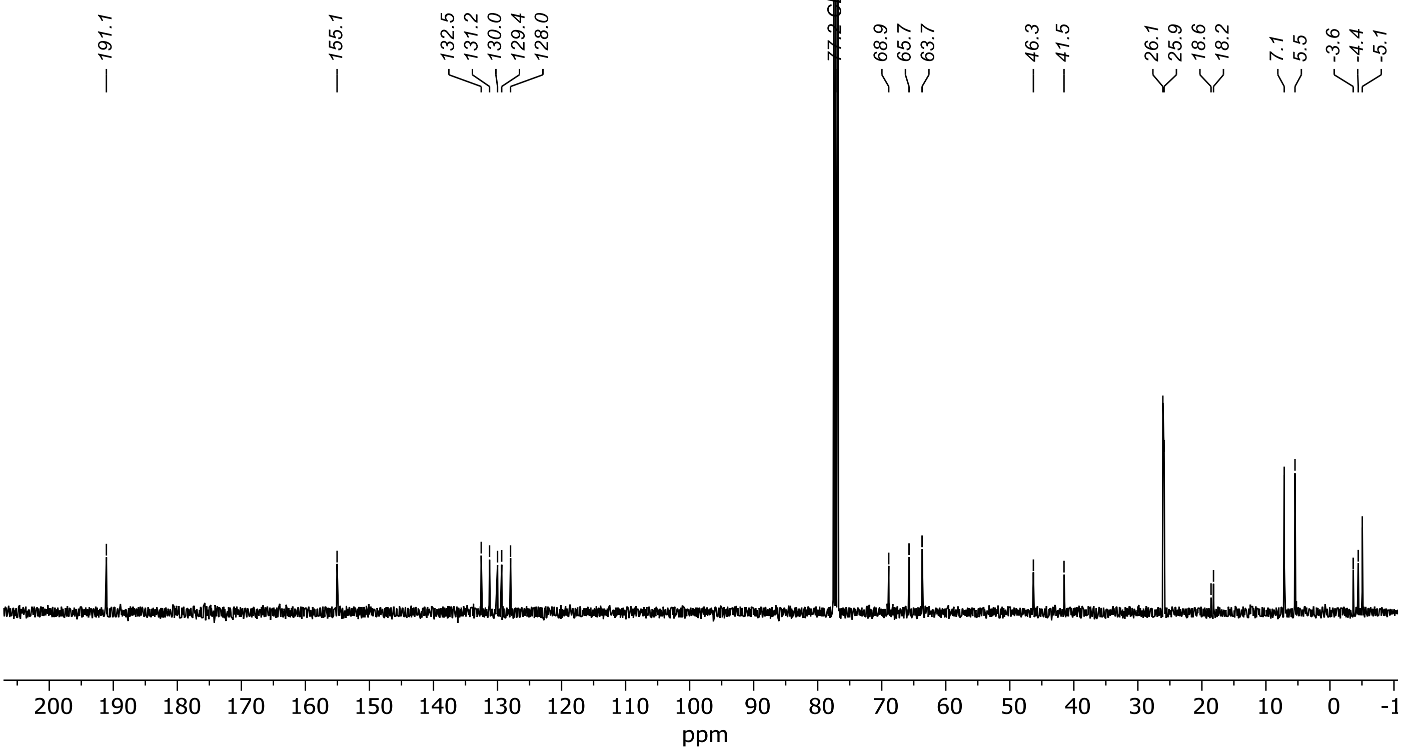

^{1}H-NMR (400.16 MHz, C_6D_6)

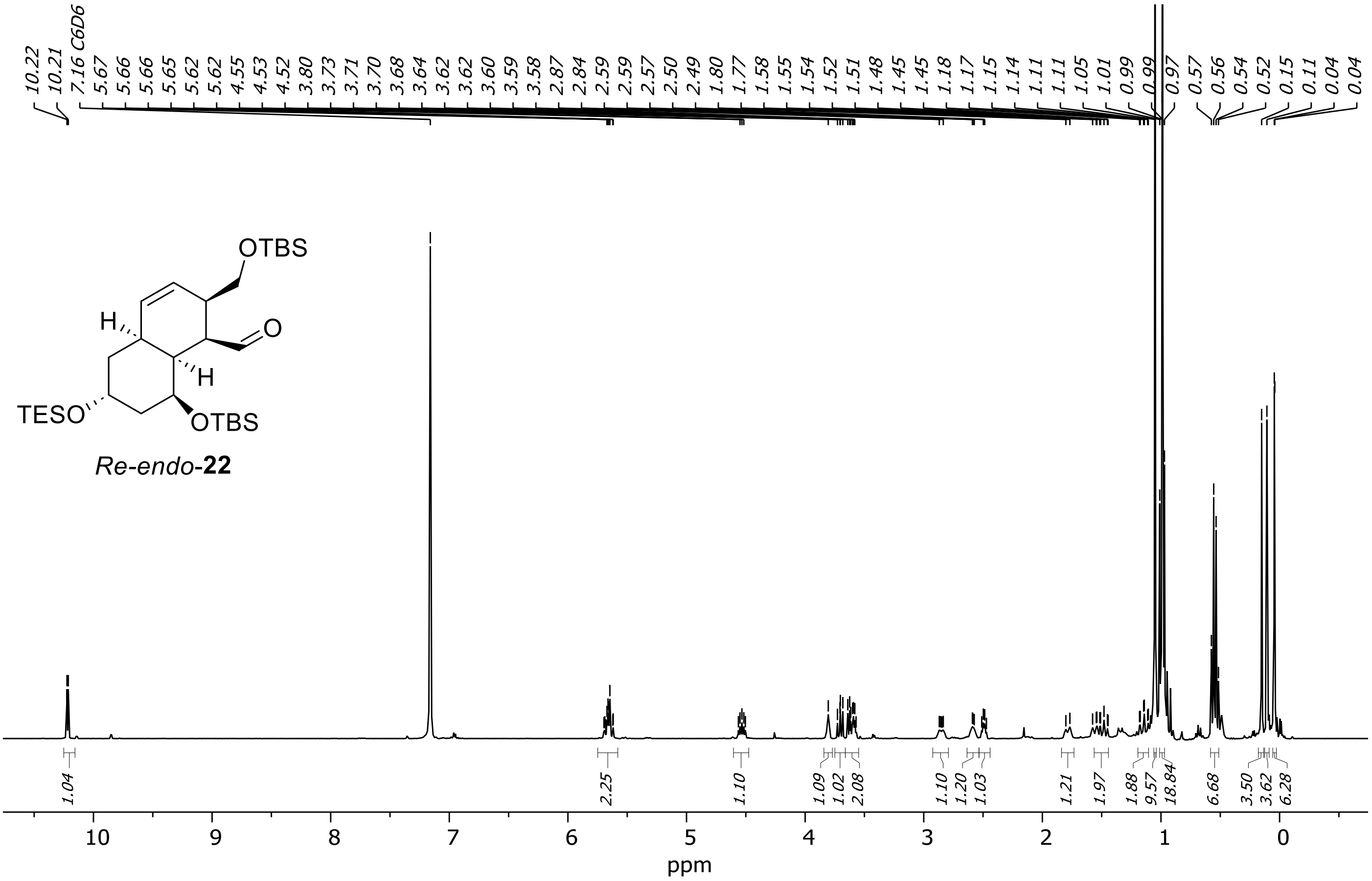

^{13}C-NMR (100.63 MHz, C_6D_6)

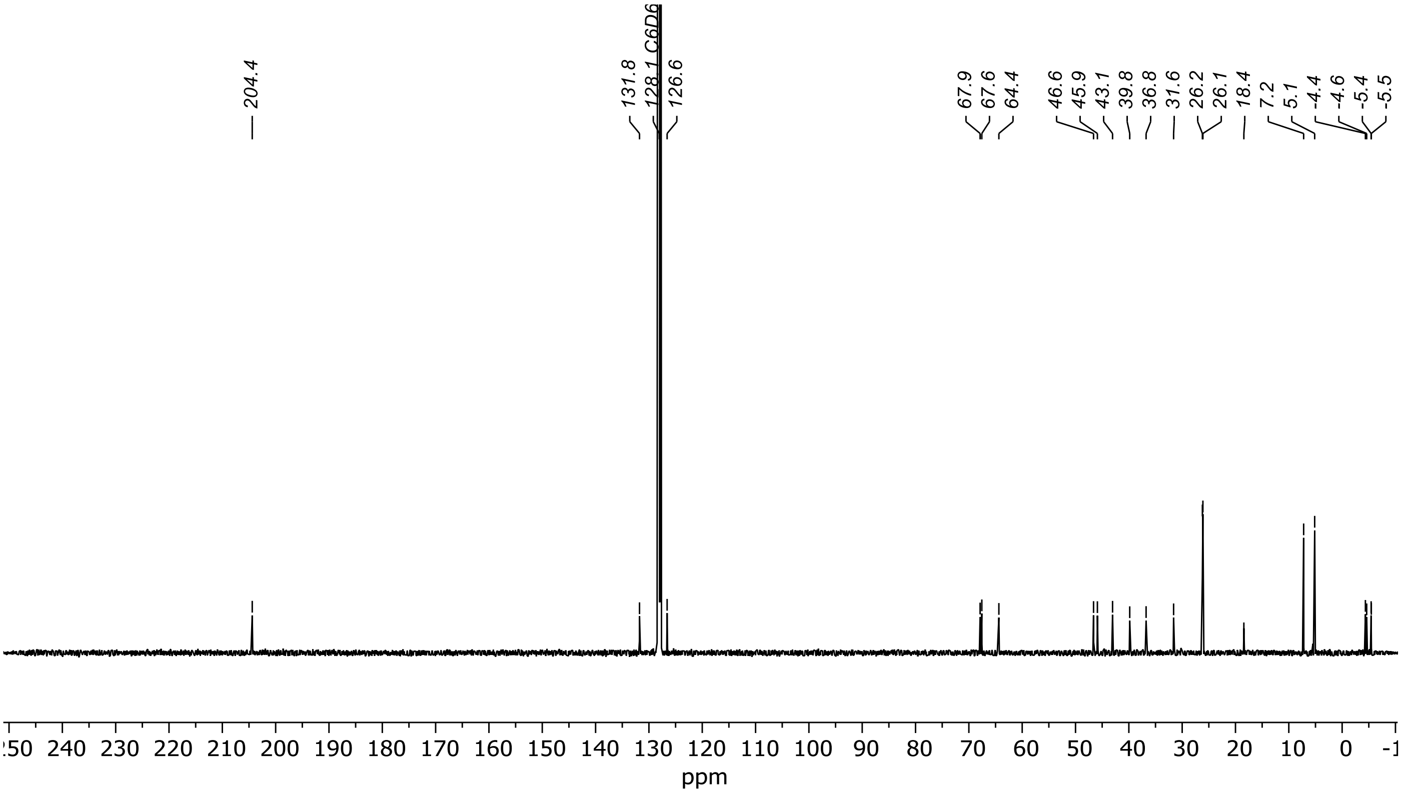

NOE-1D (400.16 MHz, freq. 4.53 ppm, C_6D_6)

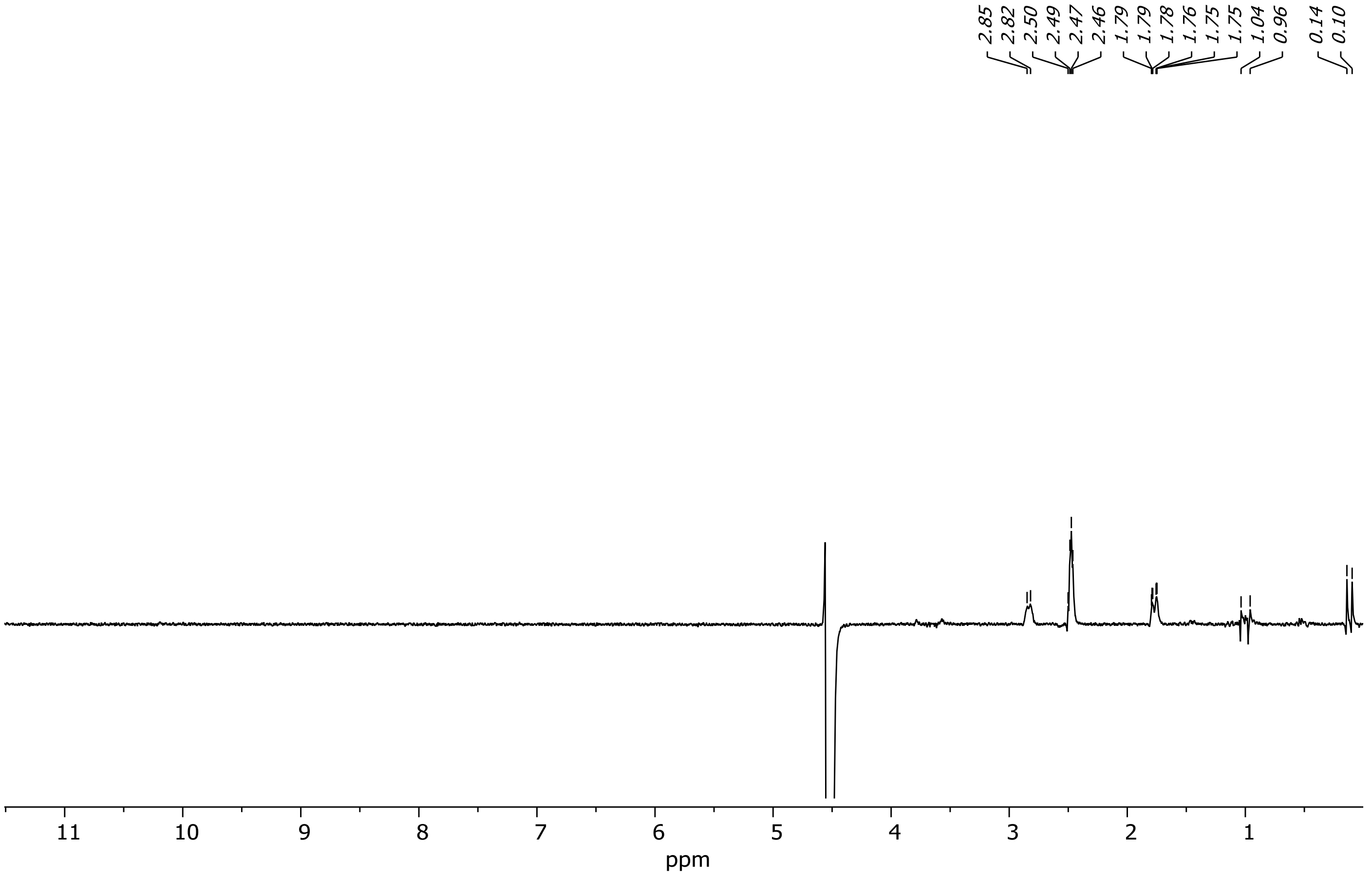

NOE-1D (400.16 MHz, freq. 3.80 ppm, C_6D_6)

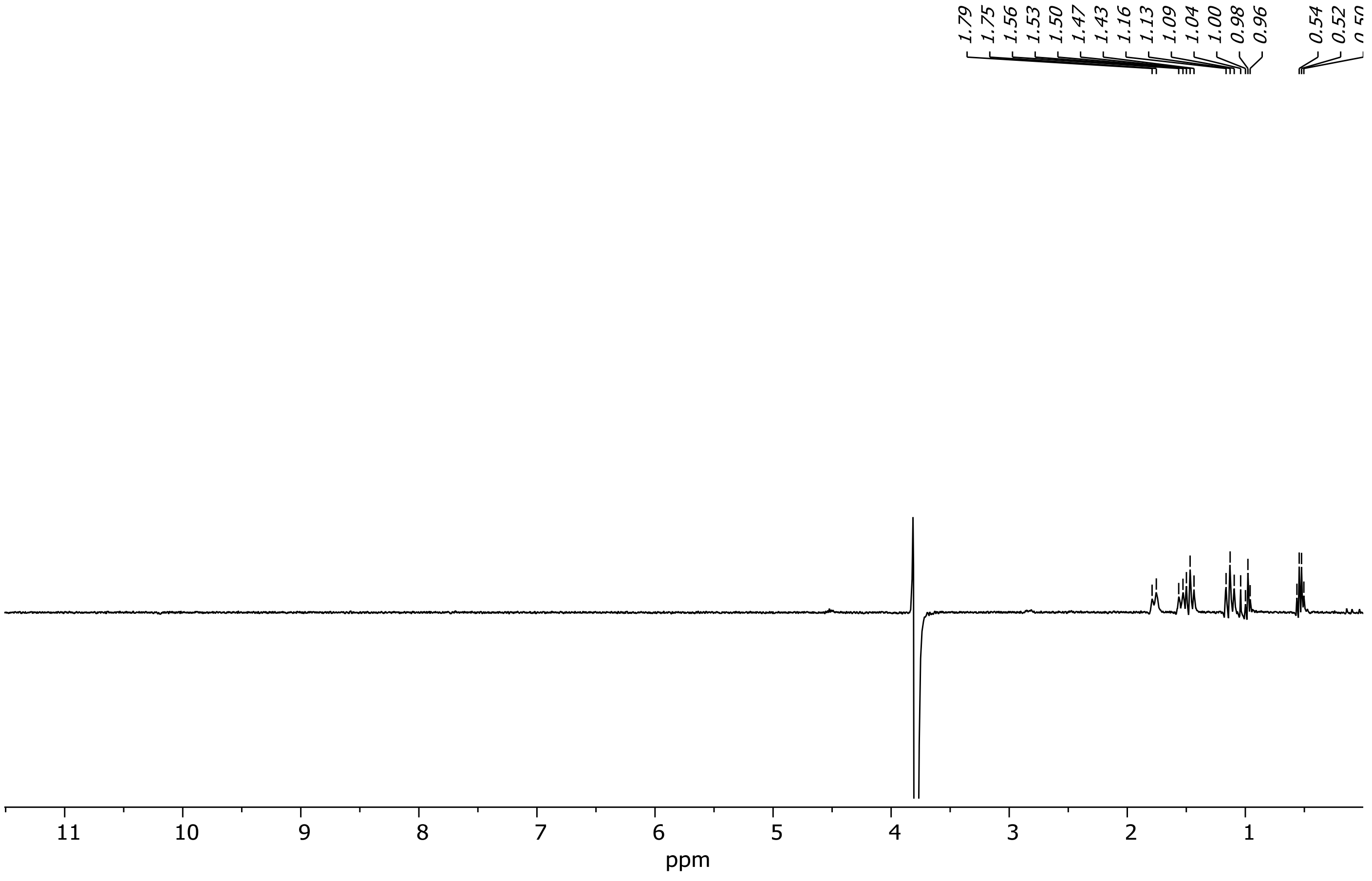

NOE-1D (400.16 MHz, freq. 2.85 ppm, C_6D_6)

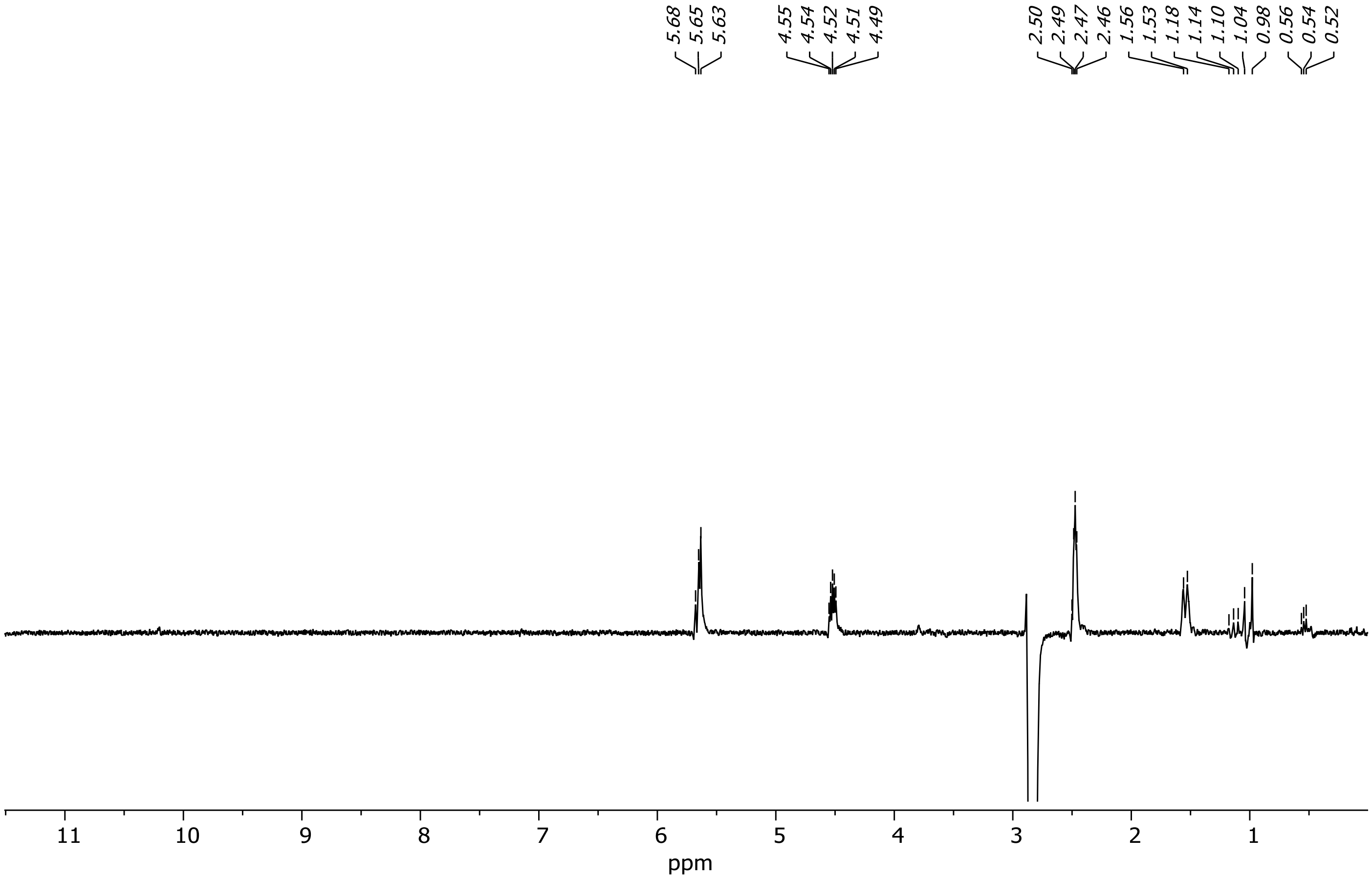

NOE-1D (400.16 MHz, freq. 2.49 ppm, C_6D_6)

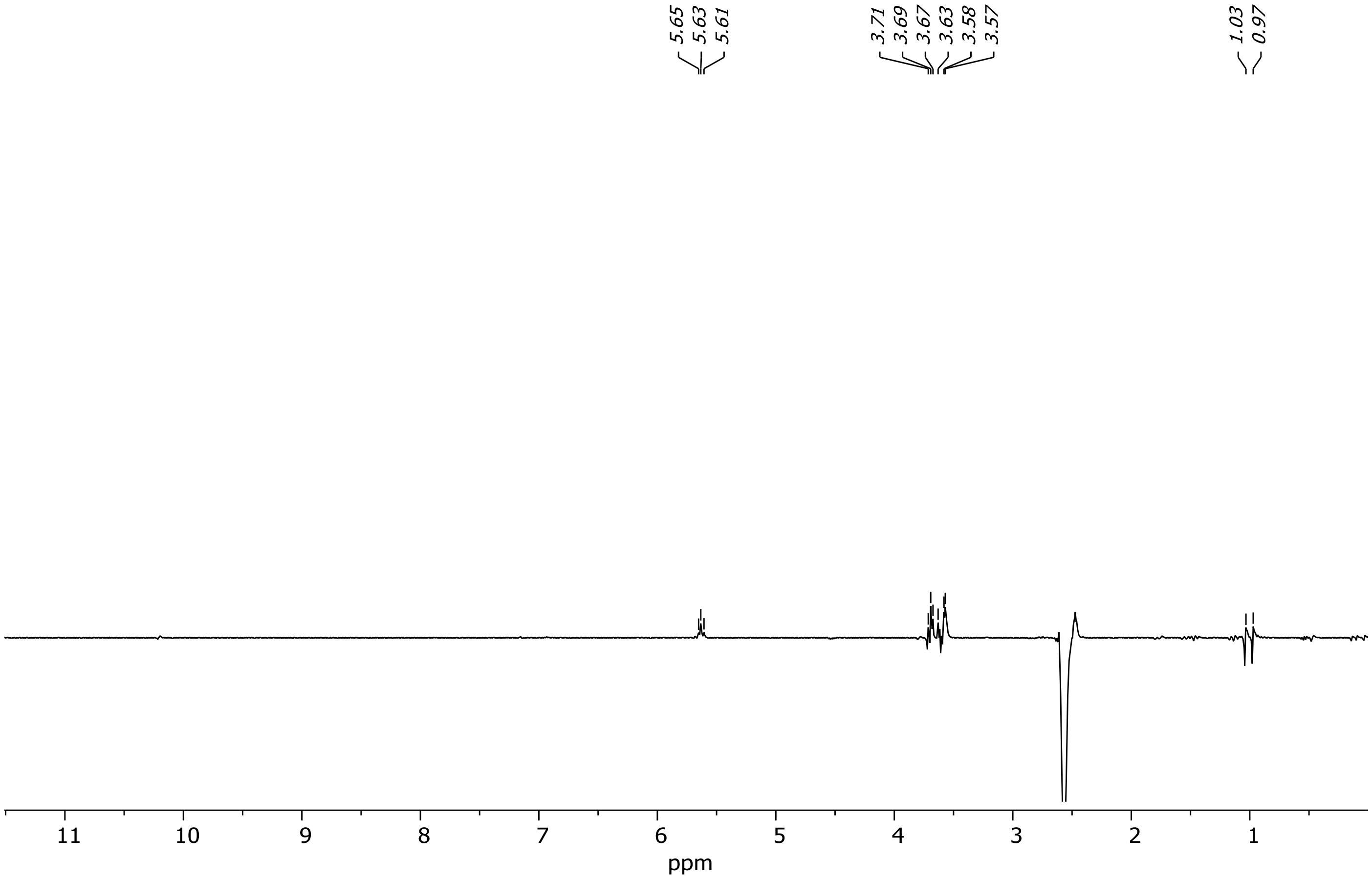

NOE-1D (400.16 MHz, freq. 2.49 ppm, C_6D_6)

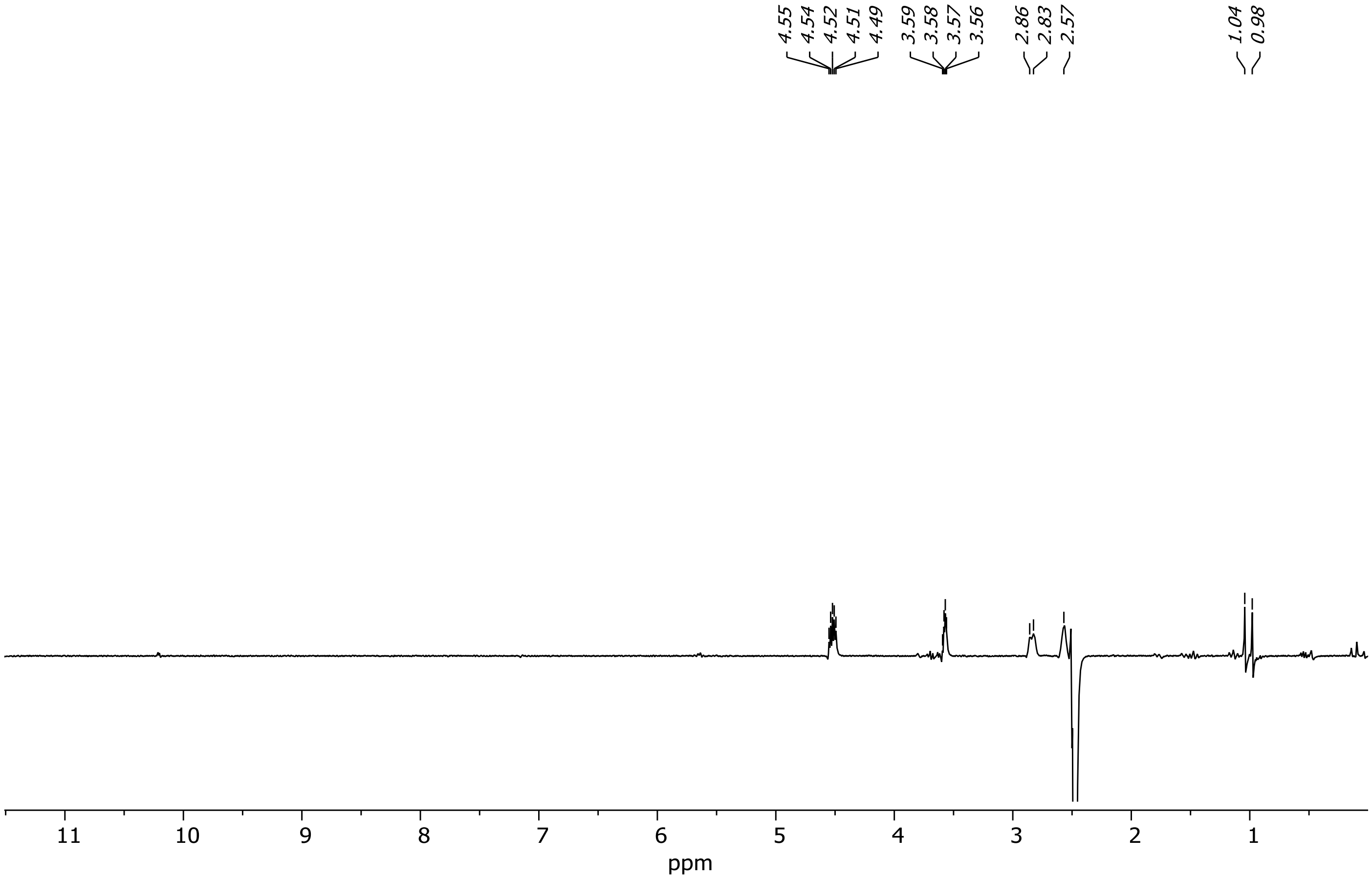

^{1}H-NMR (400.16 MHz, $CDCl_3$)

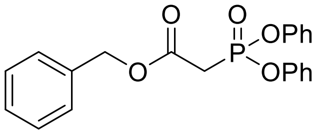

25

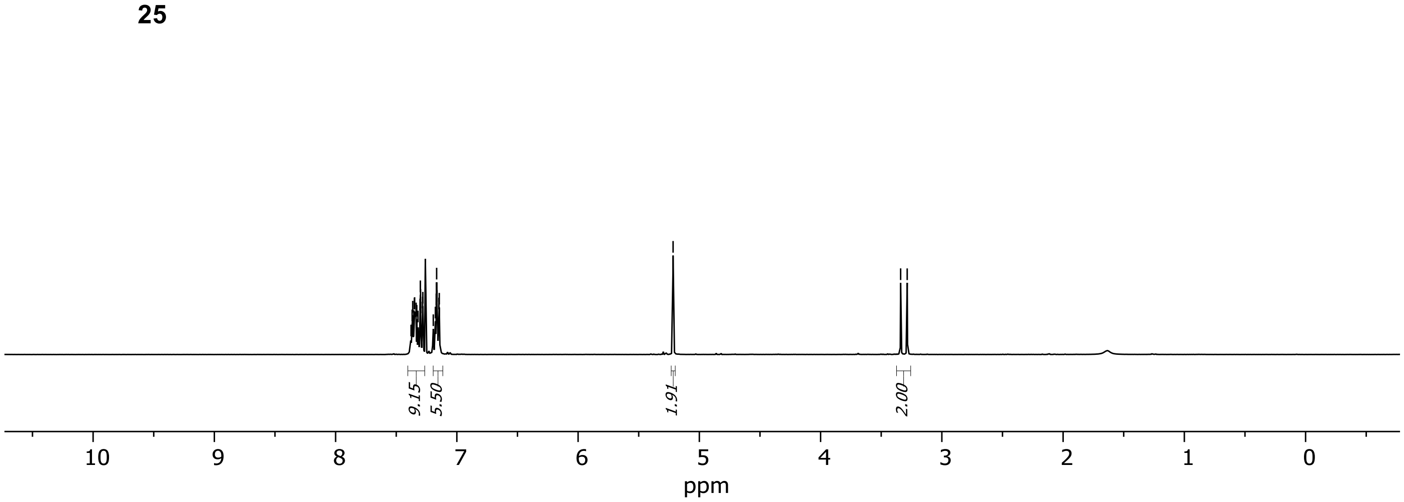

^{13}C-NMR (100.63 MHz, $CDCl_3$)

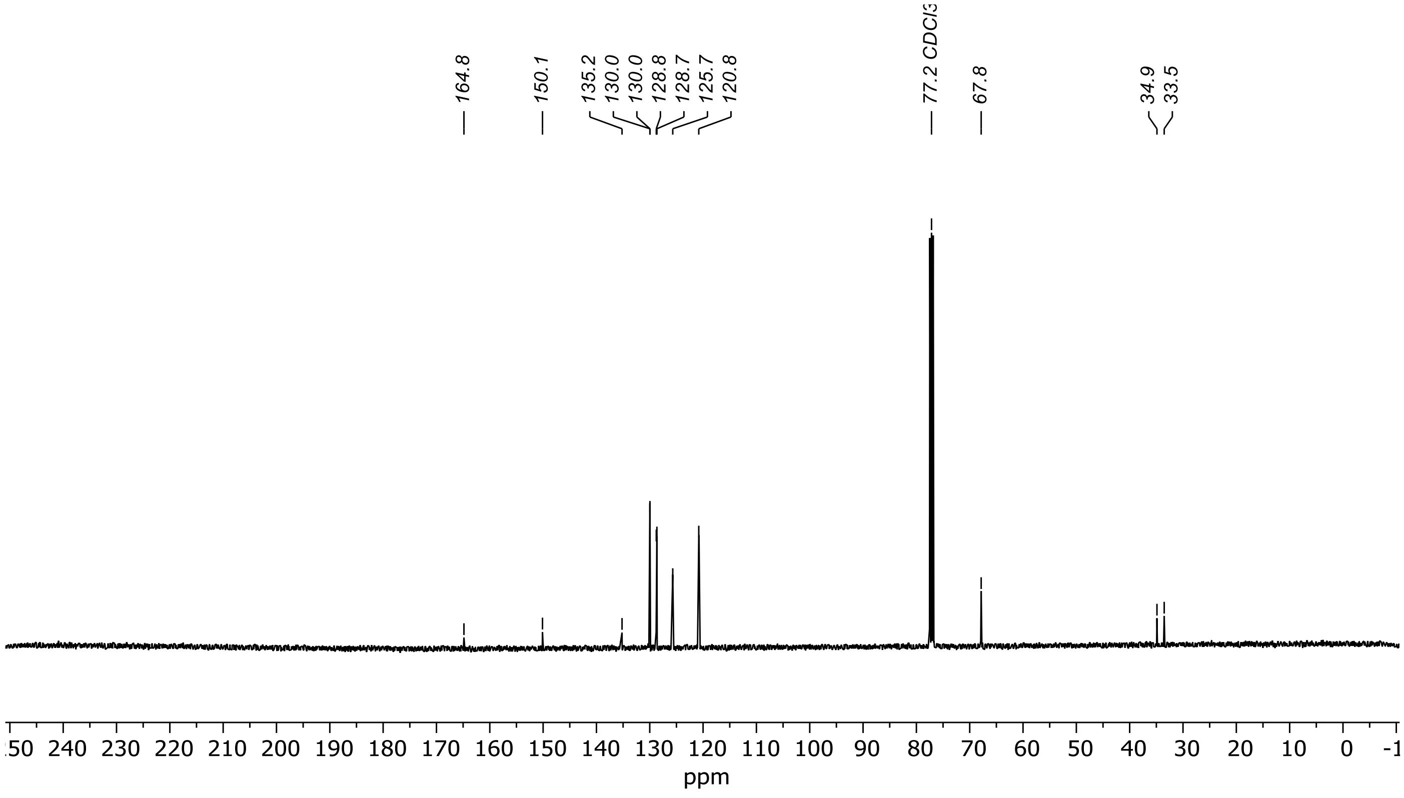

^{31}P-NMR (162.0 MHz, $CDCl_3$)

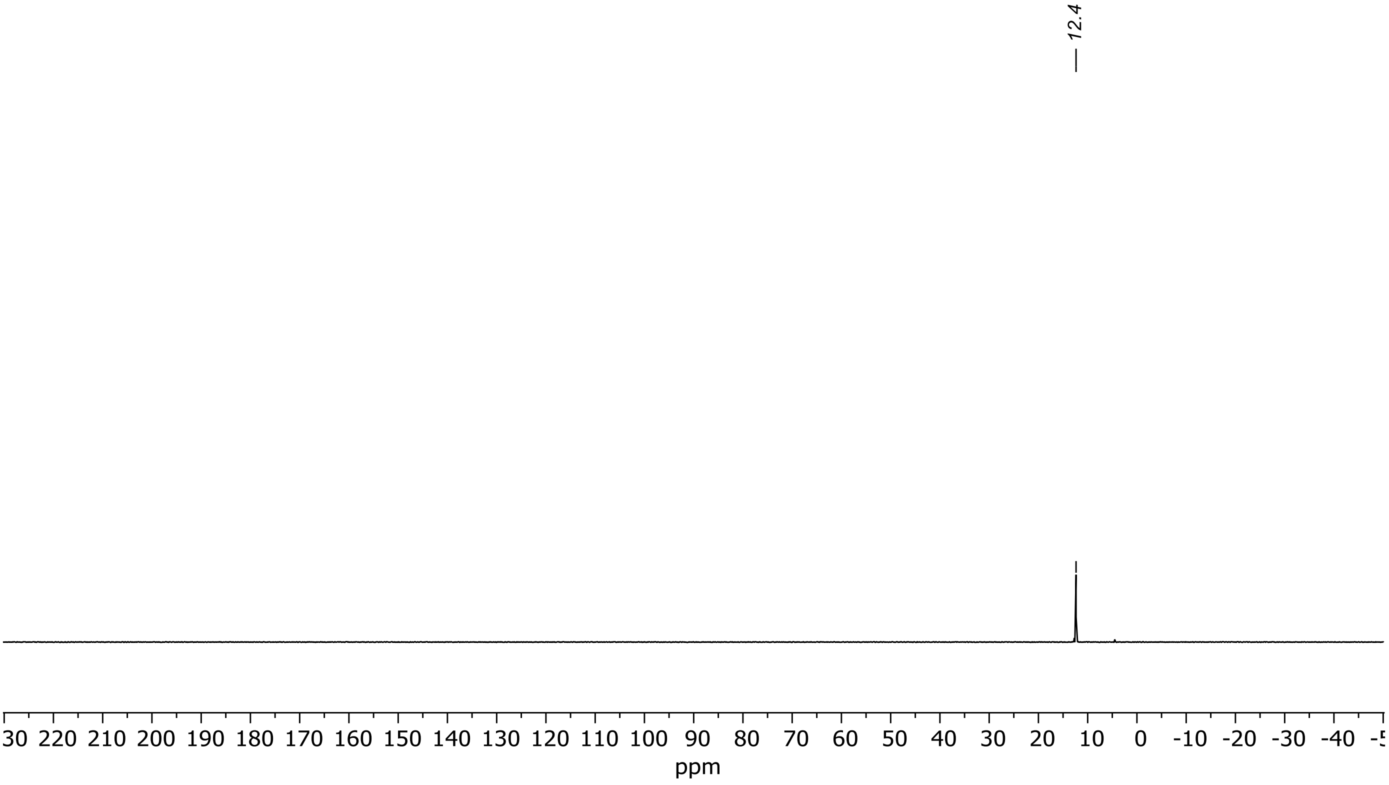

^{1}H-NMR (400.16 MHz, $CDCl_3$)

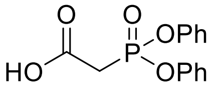

26

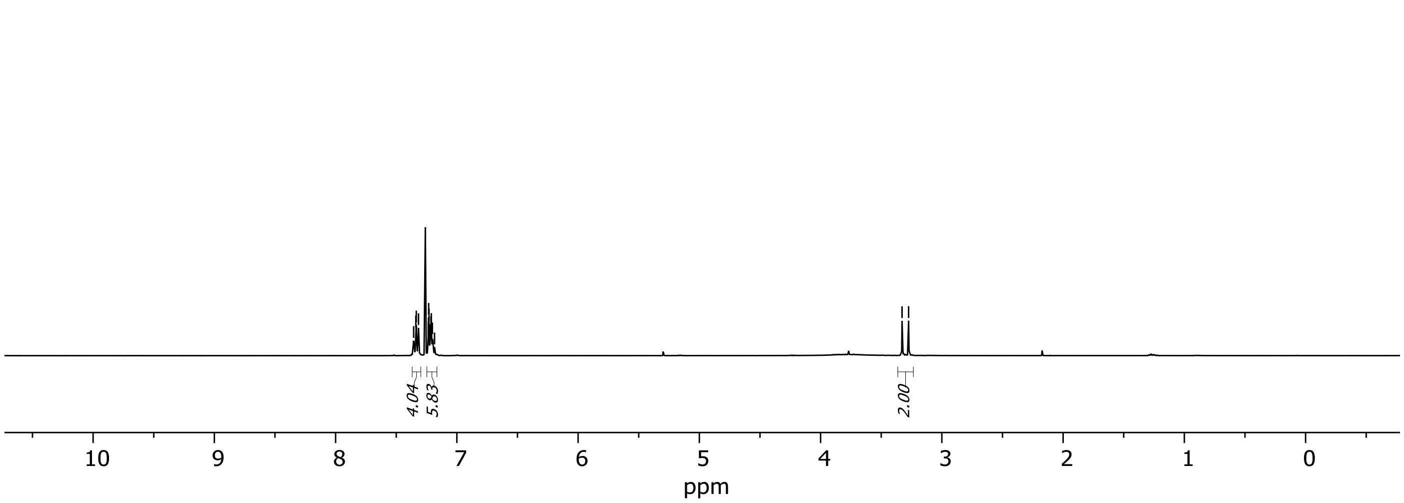

^{13}C-NMR (100.63 MHz, $CDCl_3$)

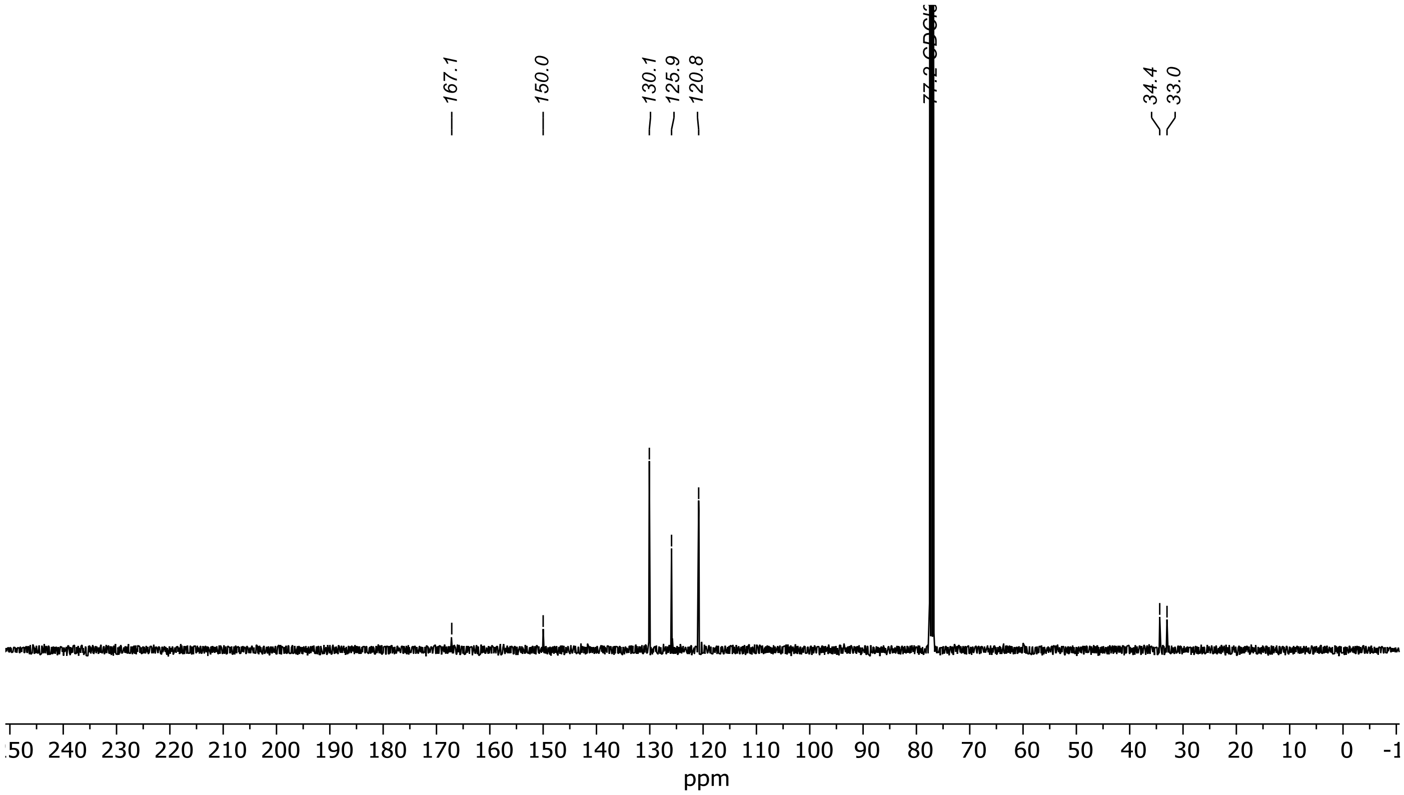

^{31}P-NMR (162.0 MHz, $CDCl_3$)

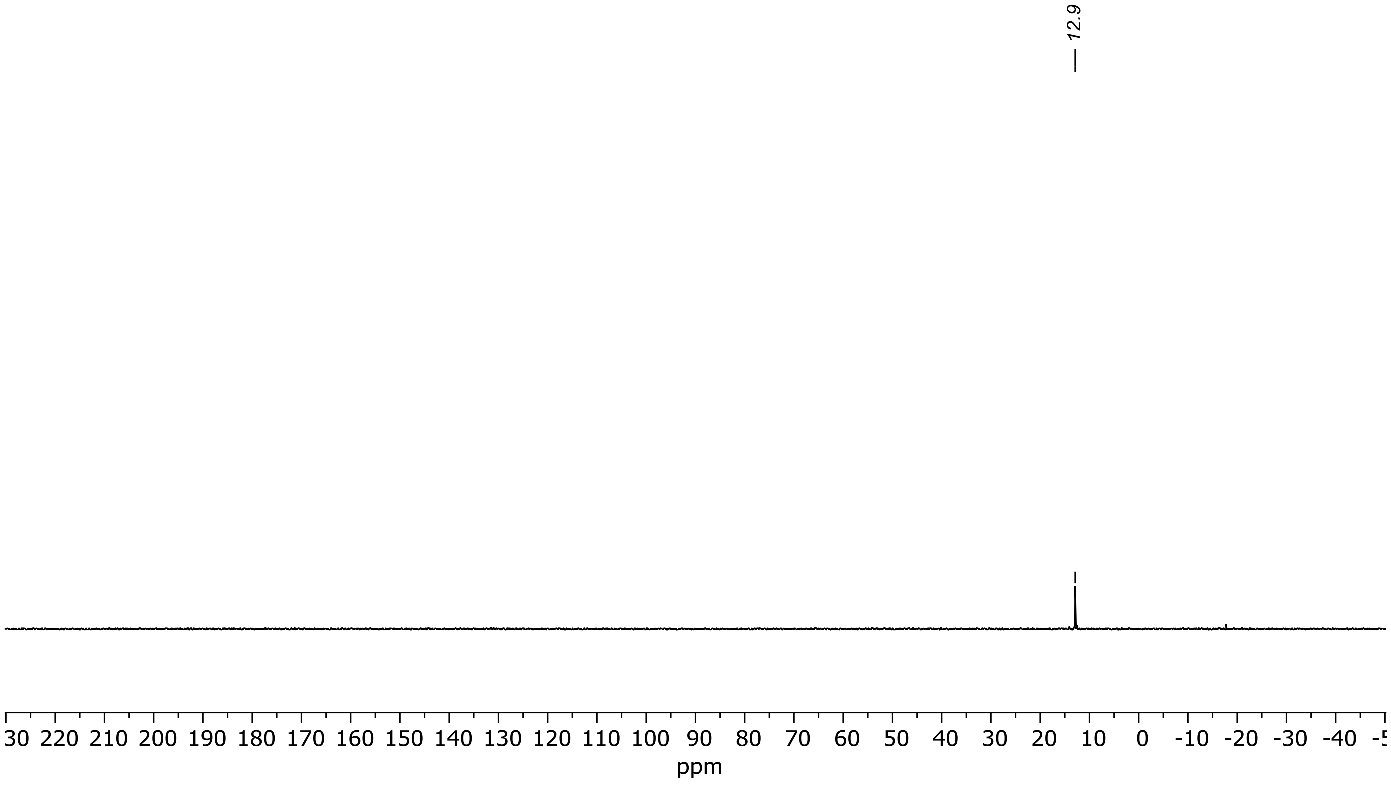

^{1}H-NMR (400.16 MHz, $CDCl_3$)

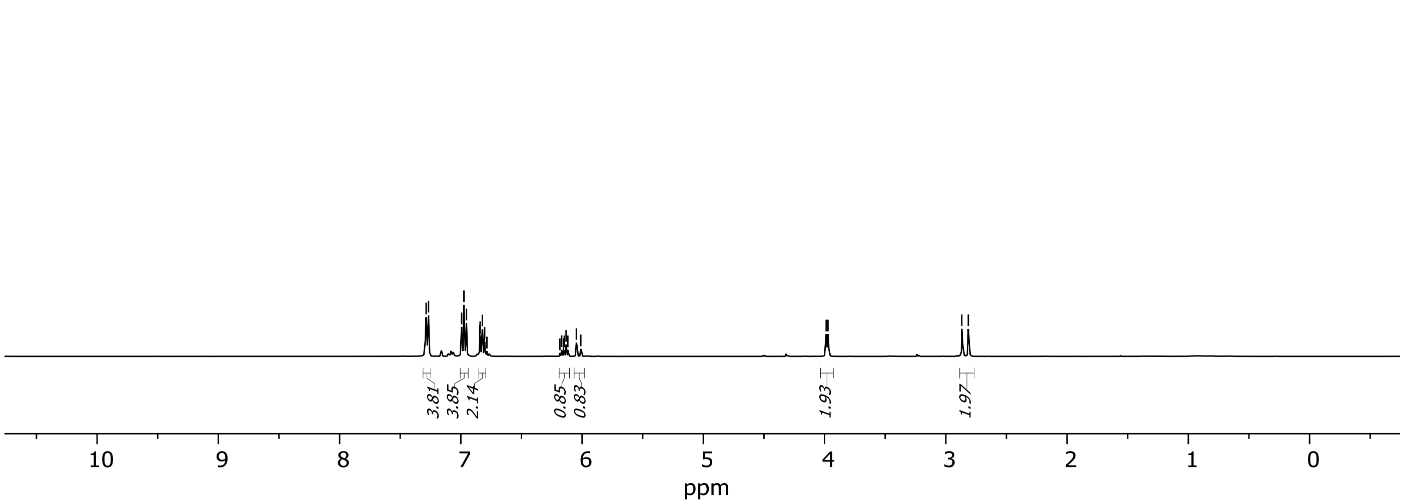

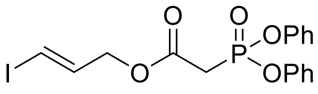

28

^{13}C-NMR (100.63 MHz, $CDCl_3$)

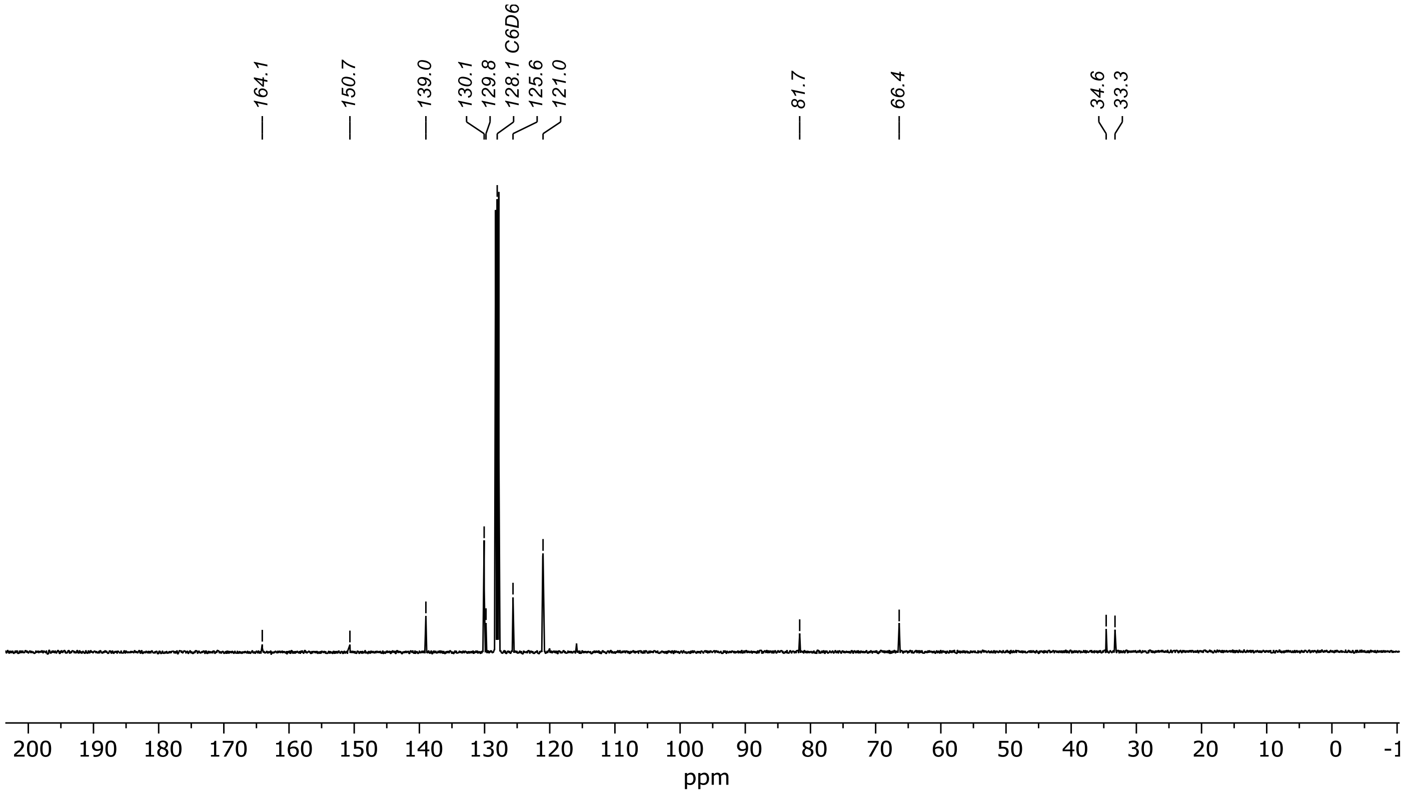

^{31}P-NMR (162.0 MHz, $CDCl_3$)

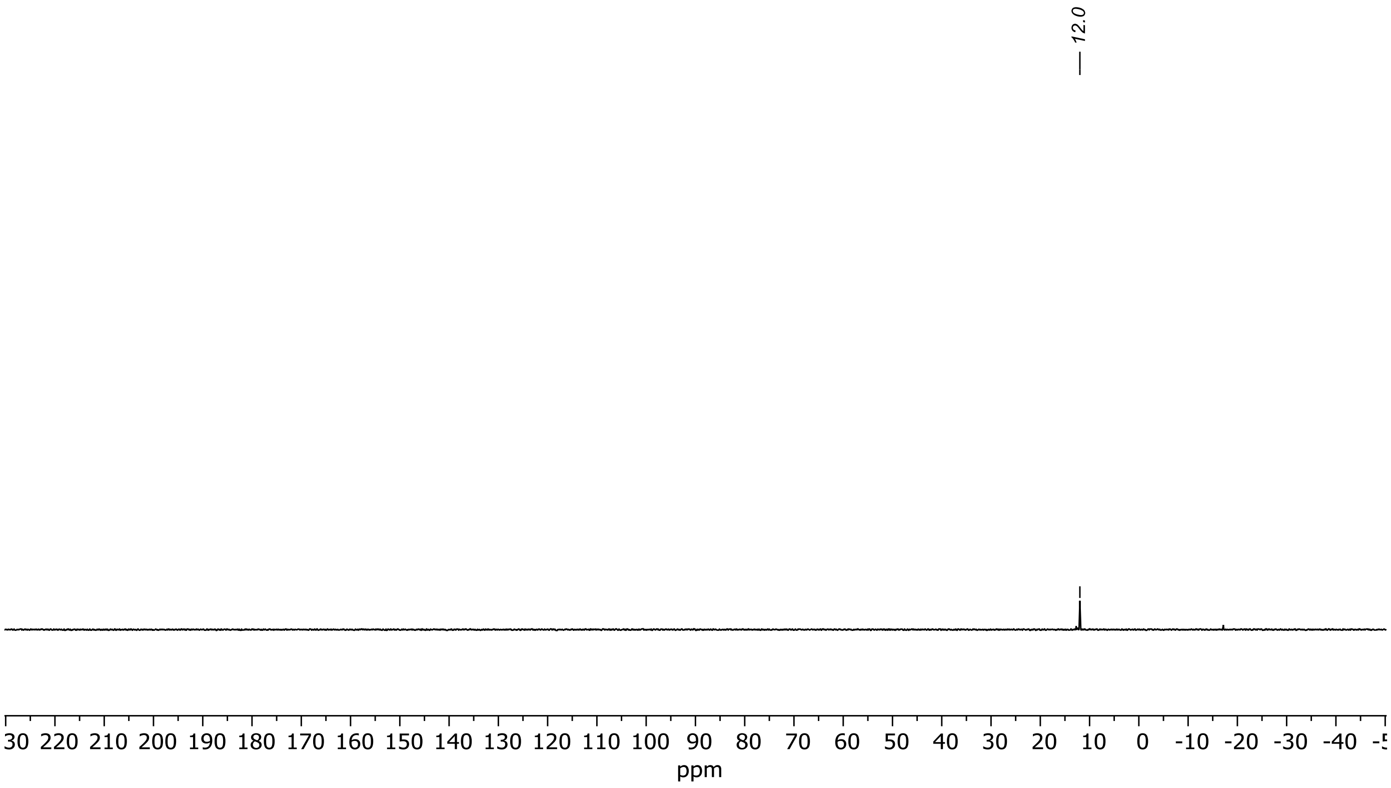

^{1}H-NMR (400.16 MHz, C_6D_6)

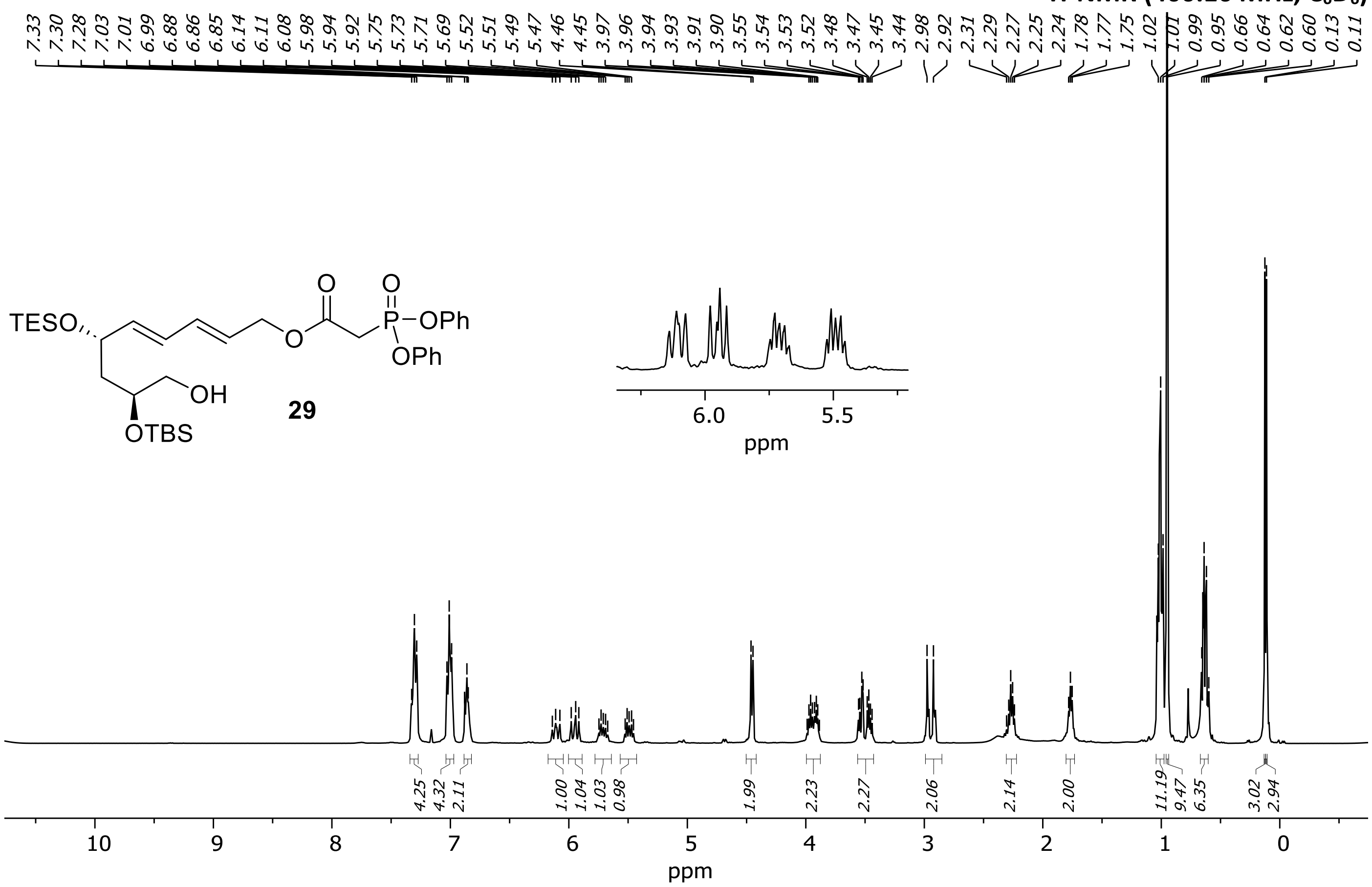

^{13}C-NMR (100.63 MHz, C_6D_6)

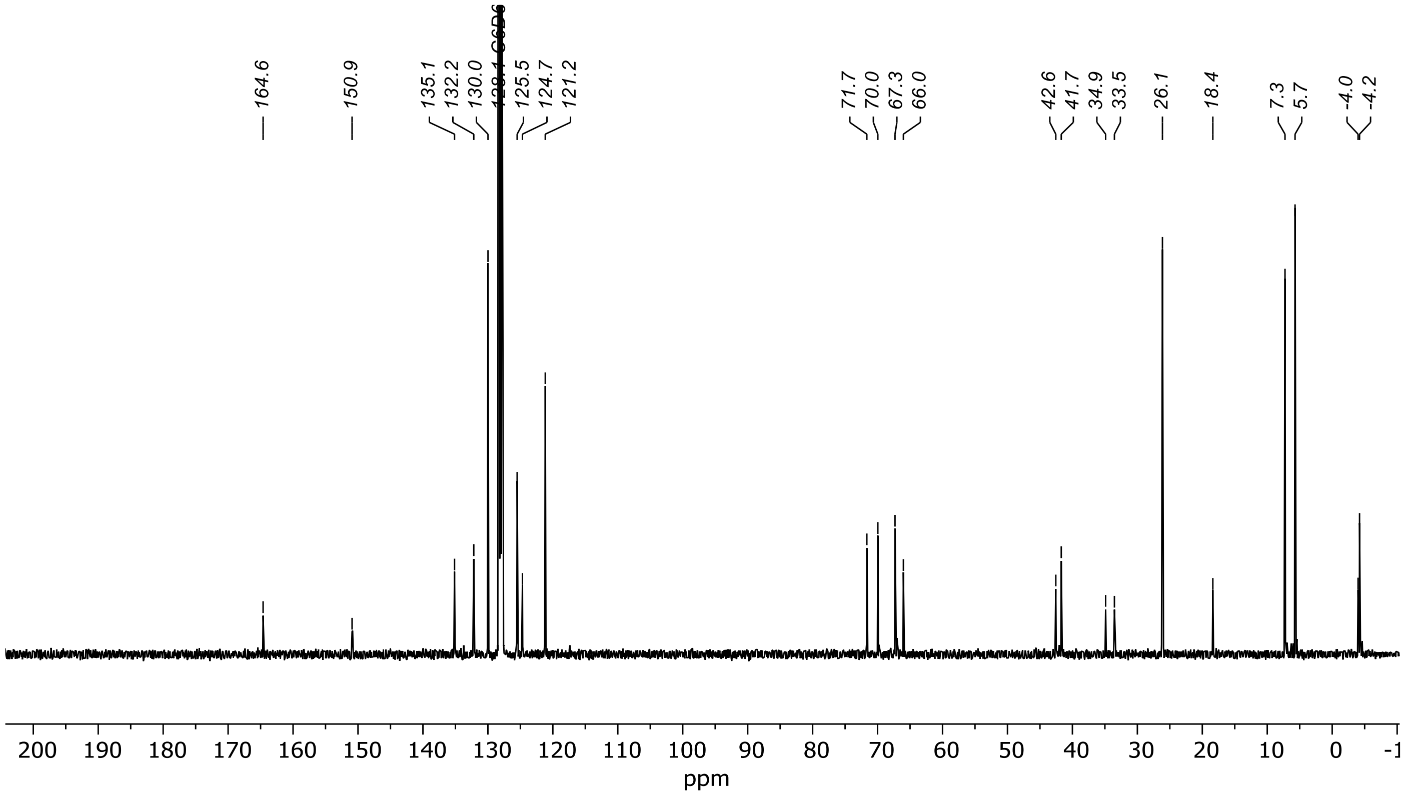

^{31}P-NMR (162.0 MHz, C_6D_6)

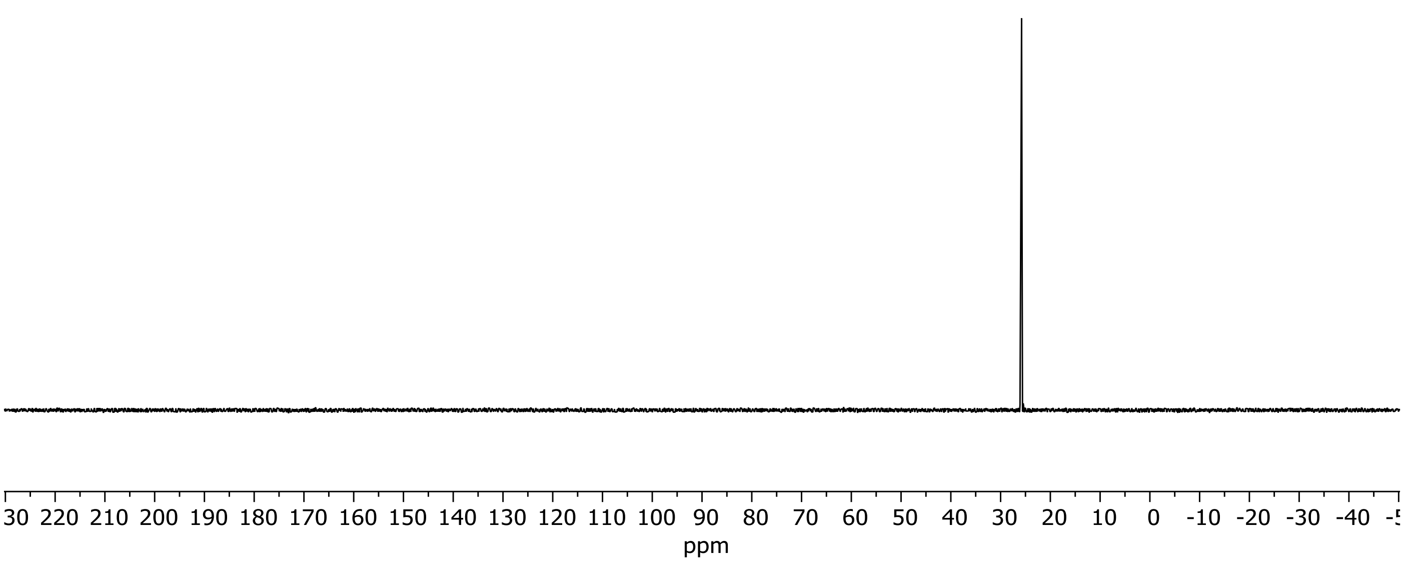

COSY (C_6D_6)

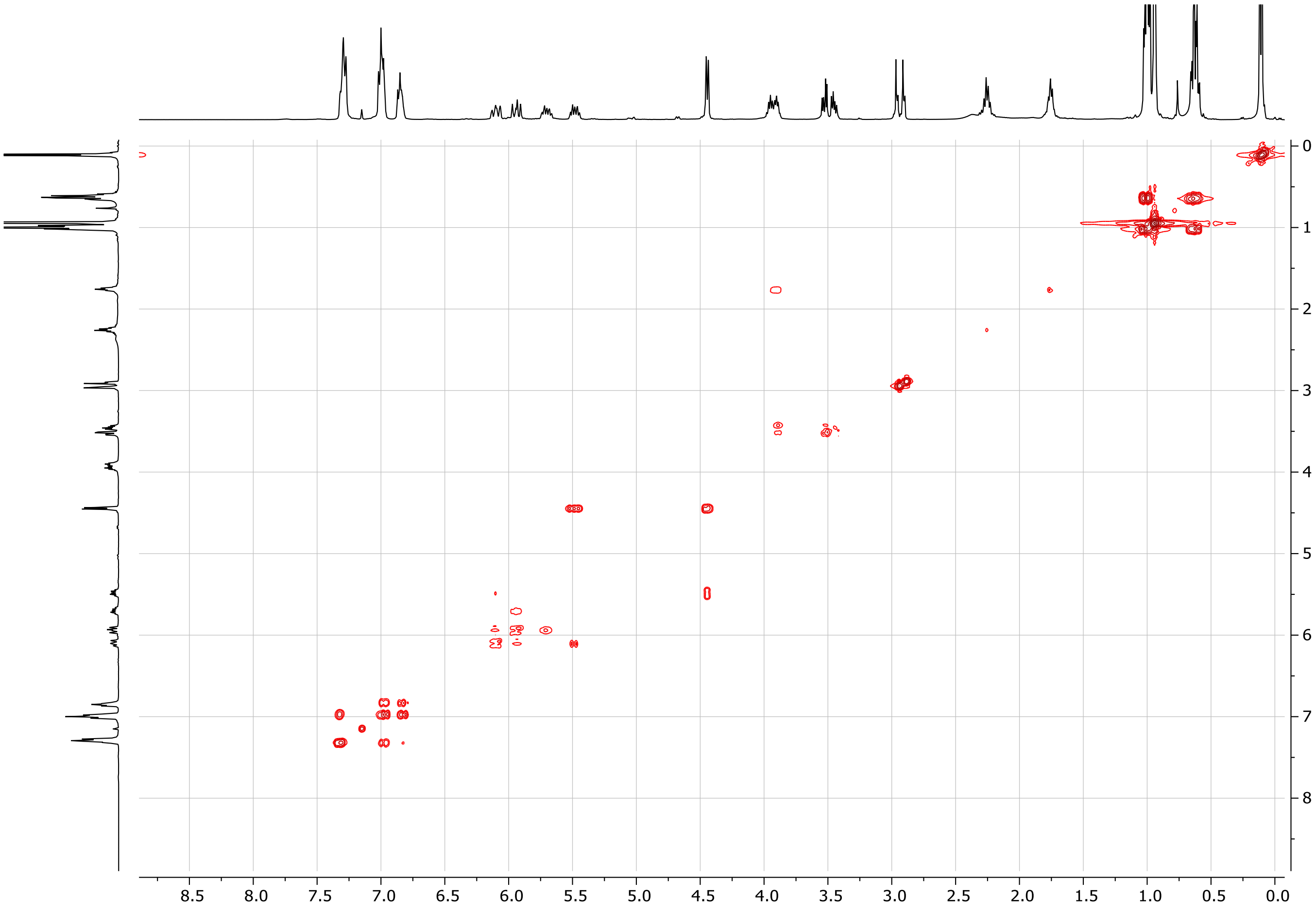

^{1}H-NMR (400.16 MHz, C_6D_6)

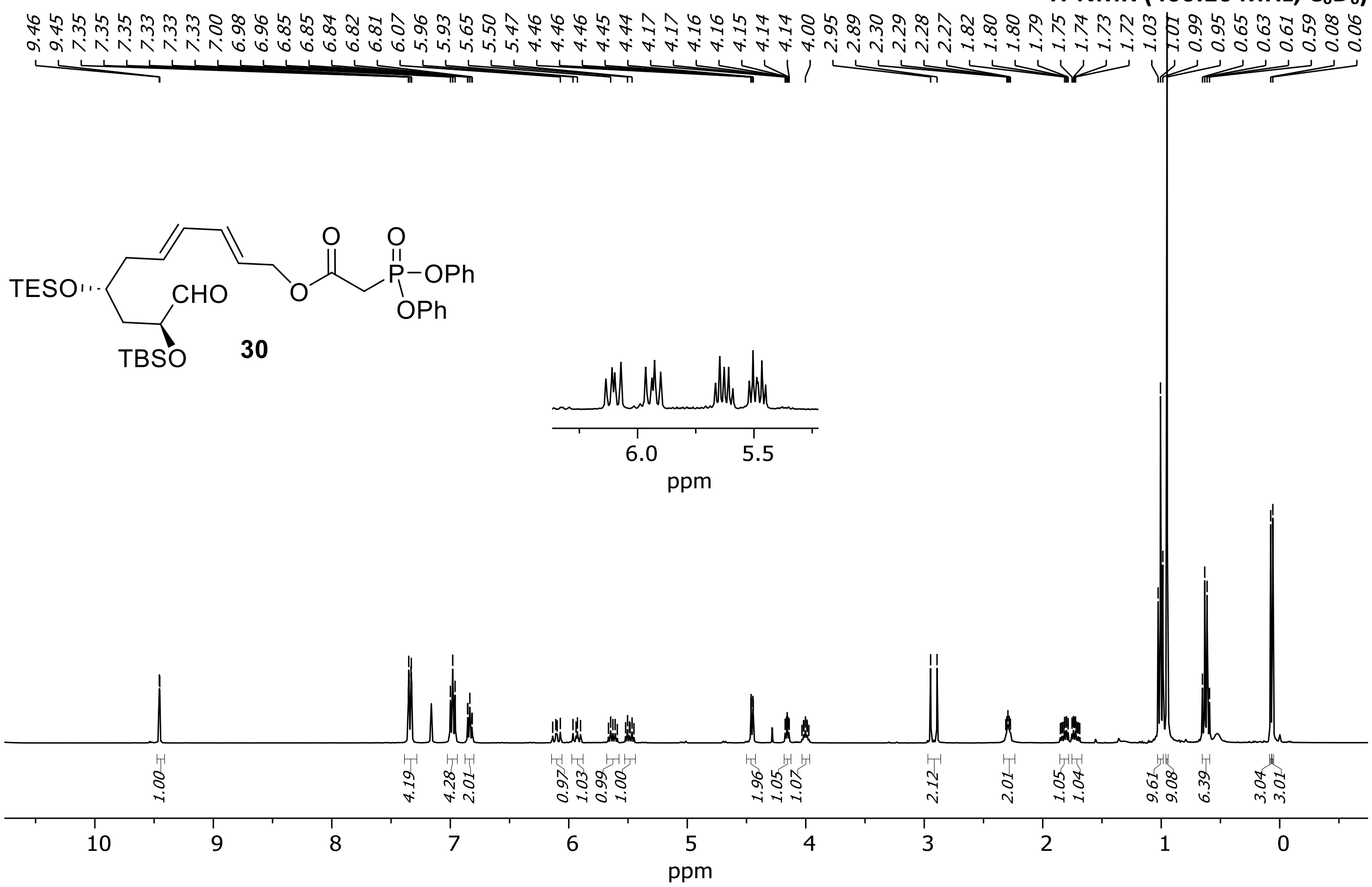

^{13}C-NMR (100.63 MHz, C_6D_6)

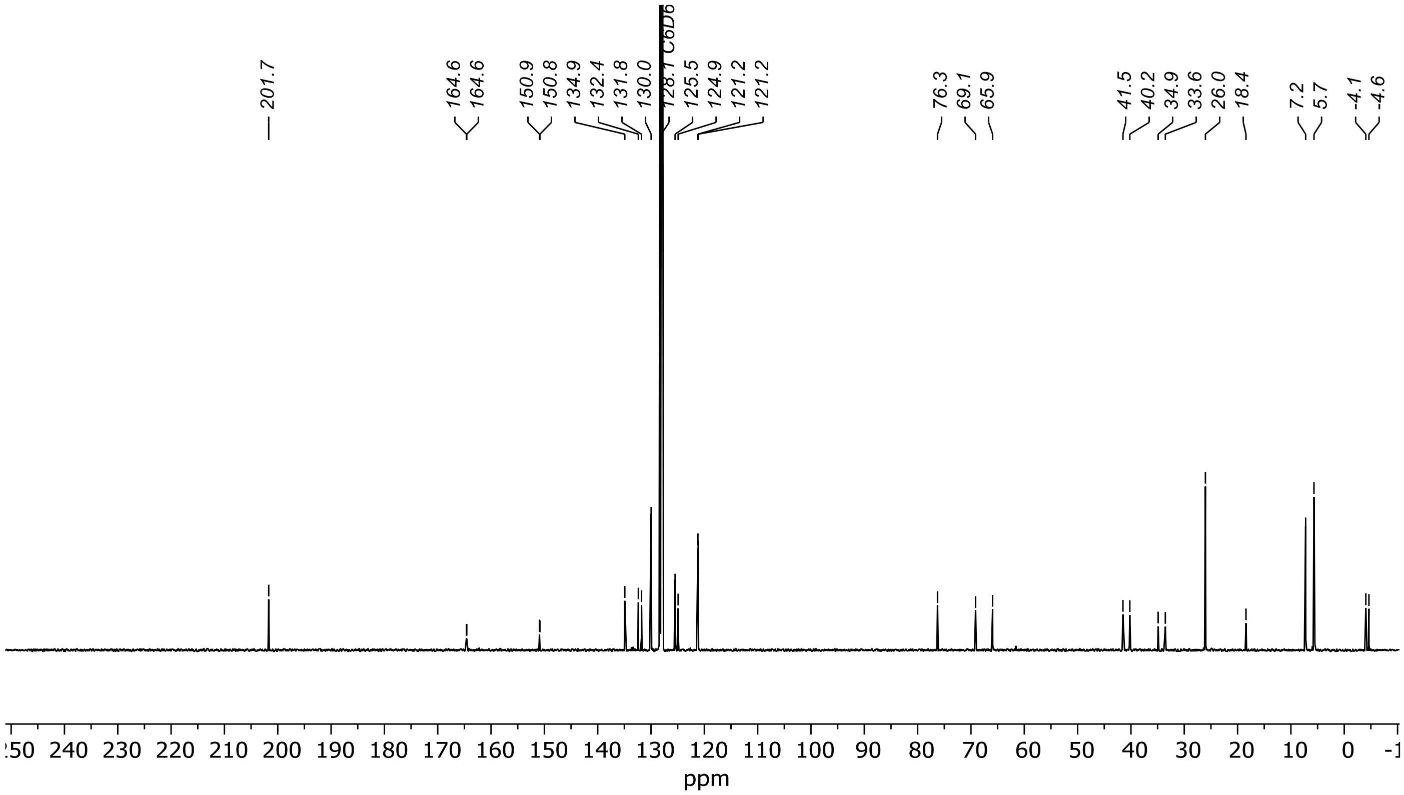

^{31}P-NMR (162.0 MHz, C_6D_6)

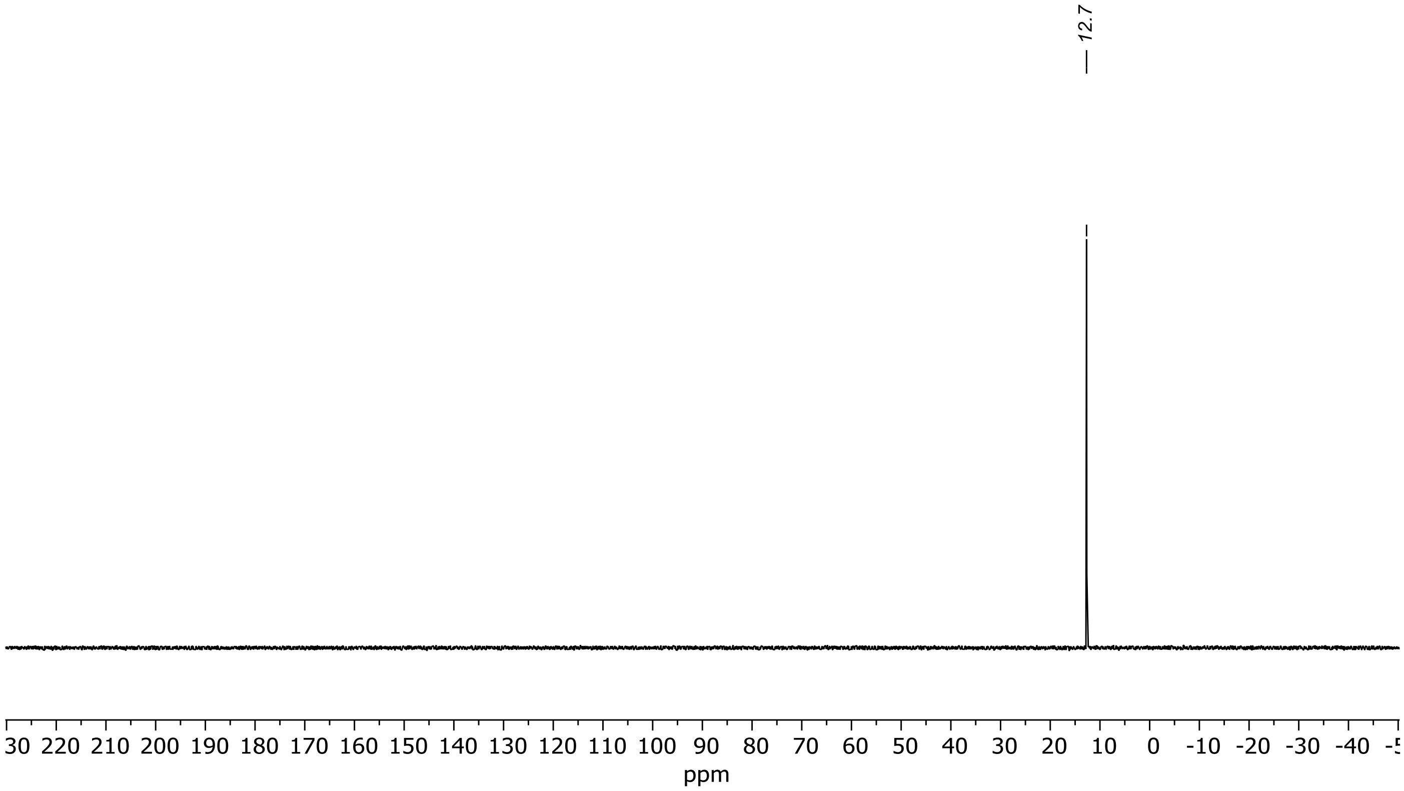

COSY (C_6D_6)

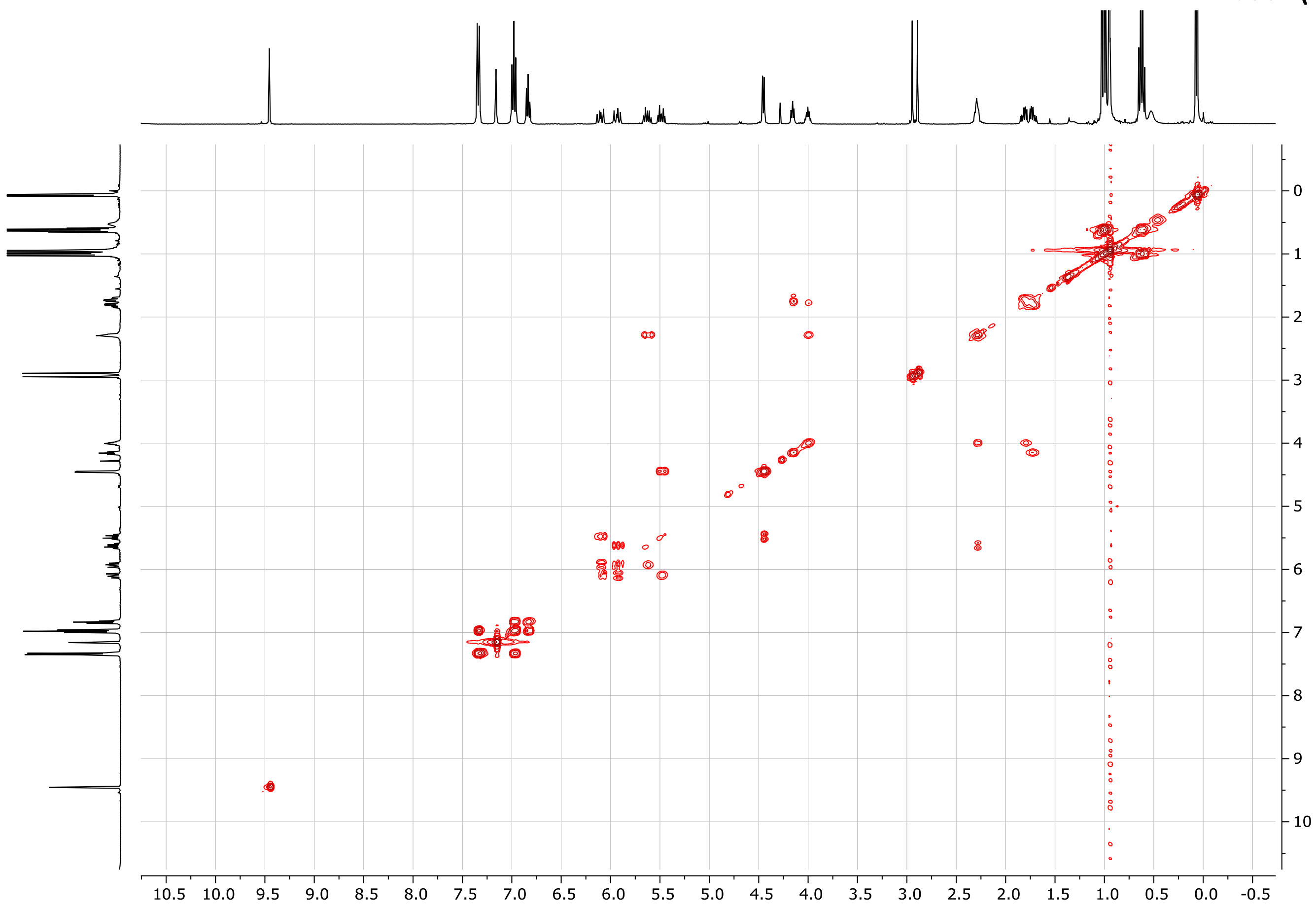

^{1}H-NMR (400.16 MHz, $(CD_3)_2CO$)

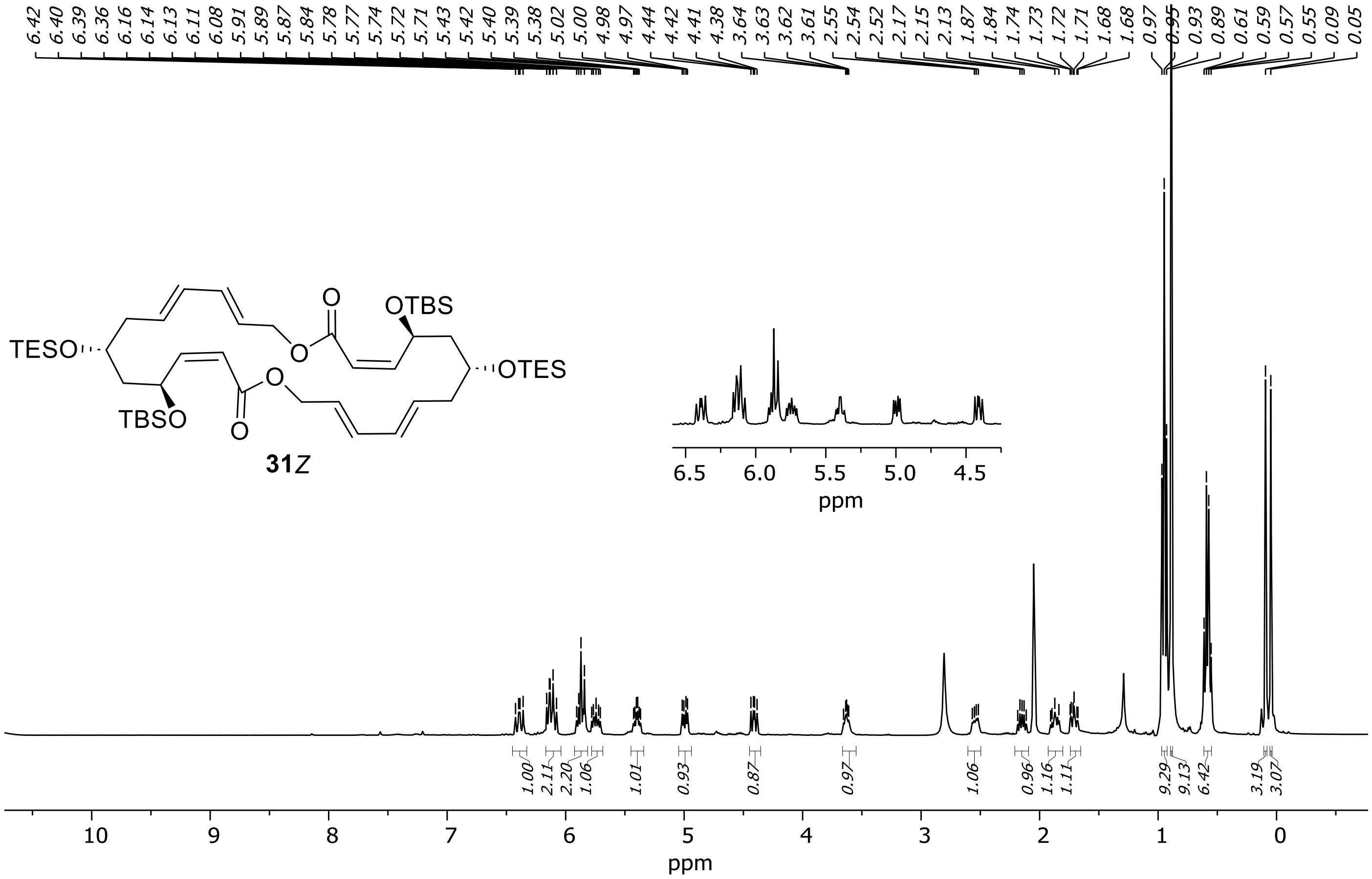

31*Z*

^{13}C-NMR (100.63 MHz, $(CD_3)_2CO$)

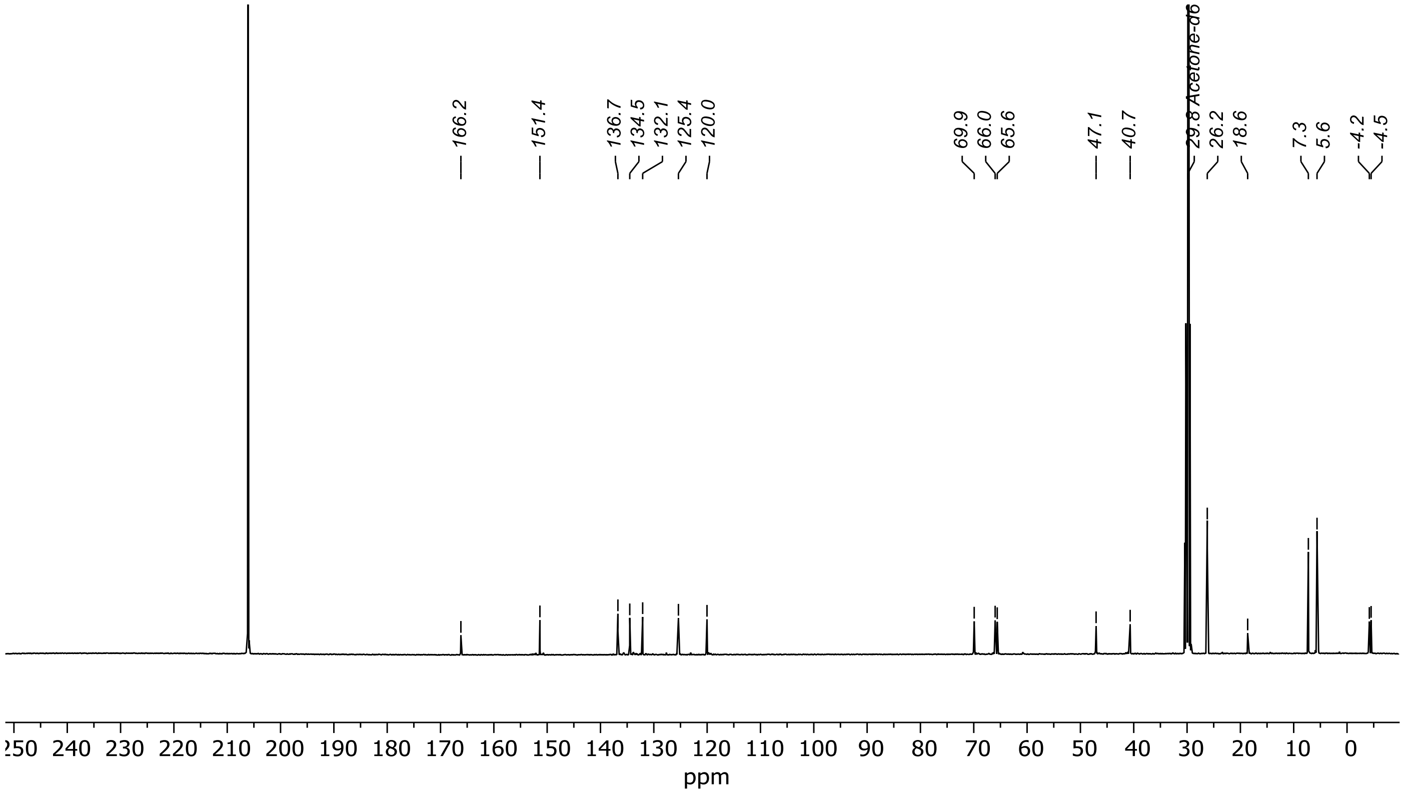

COSY ((CD$_3$)$_2$CO)

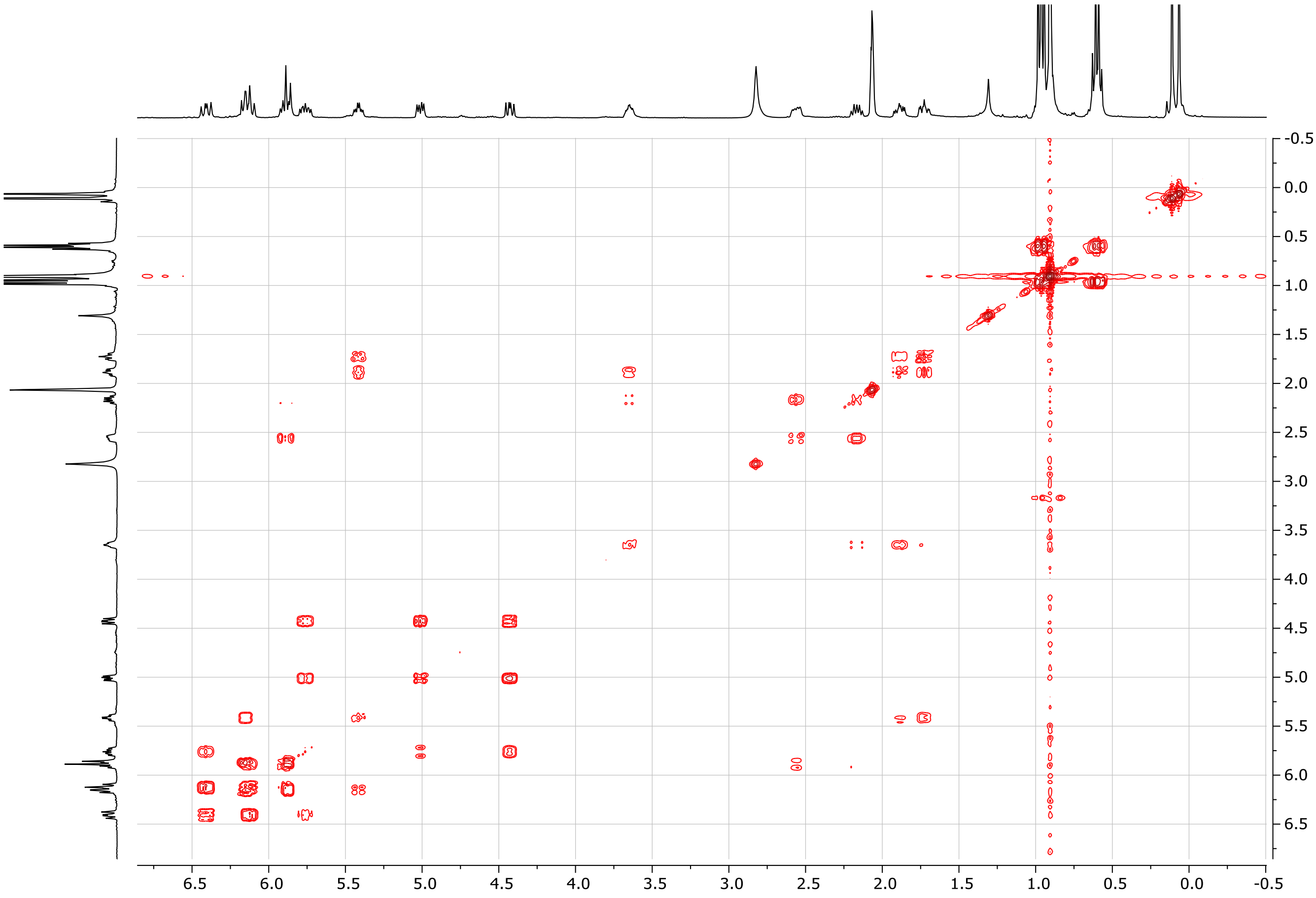

^{1}H-NMR (400.16 MHz, C_6D_6)

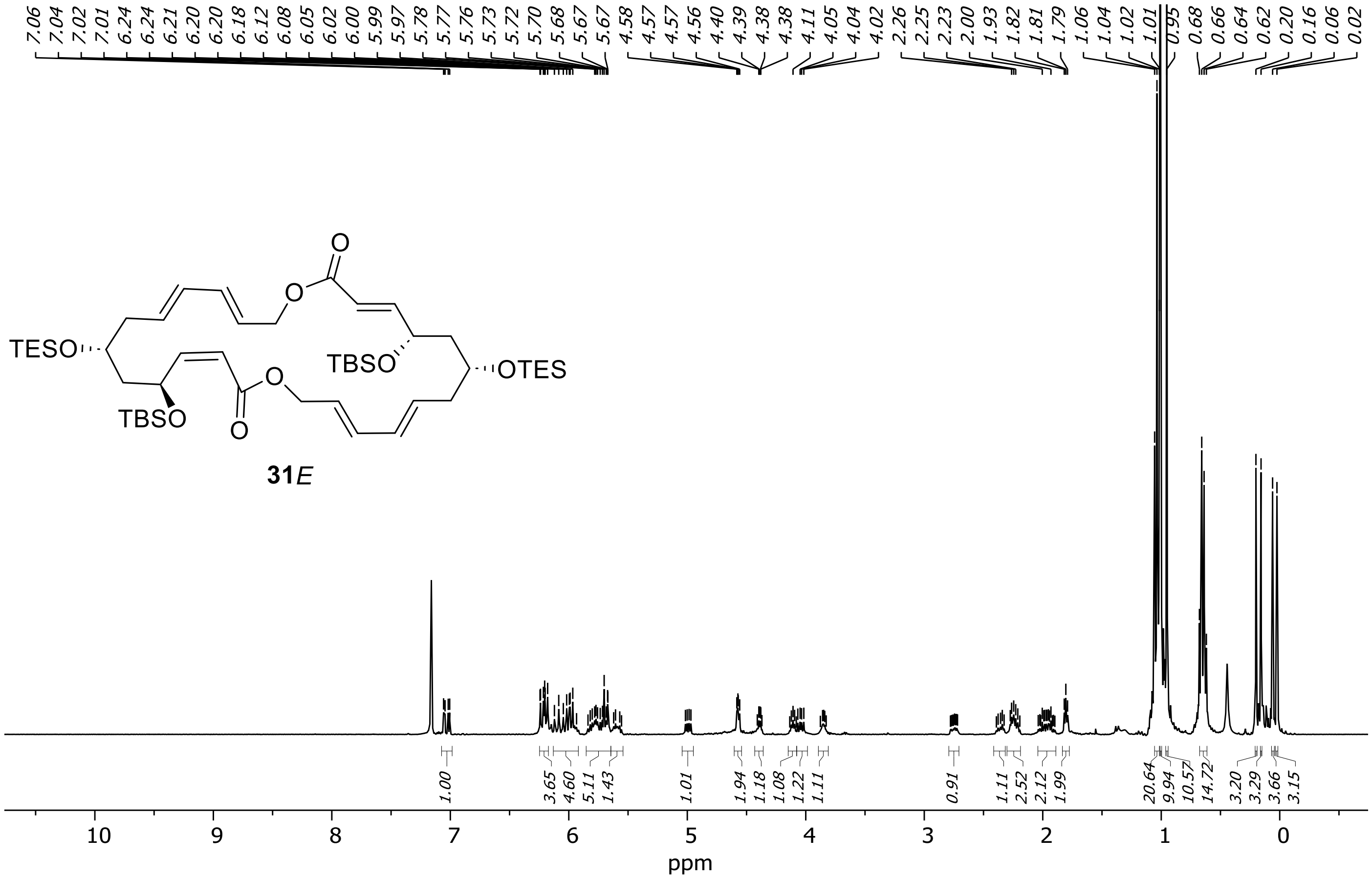

31*E*

^{13}C-NMR (100.63 MHz, C_6D_6)

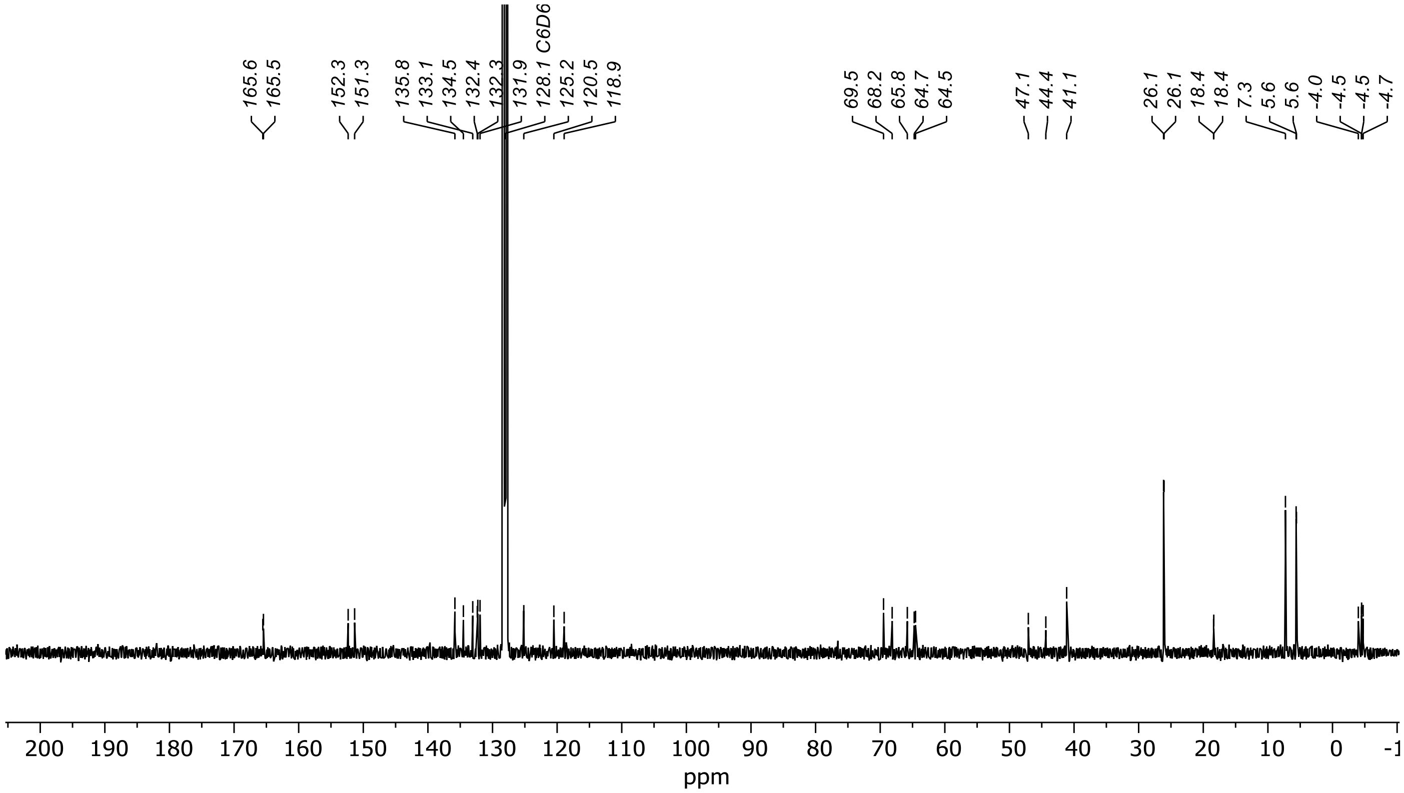

^{1}H-NMR (400.16 MHz, C_6D_6)

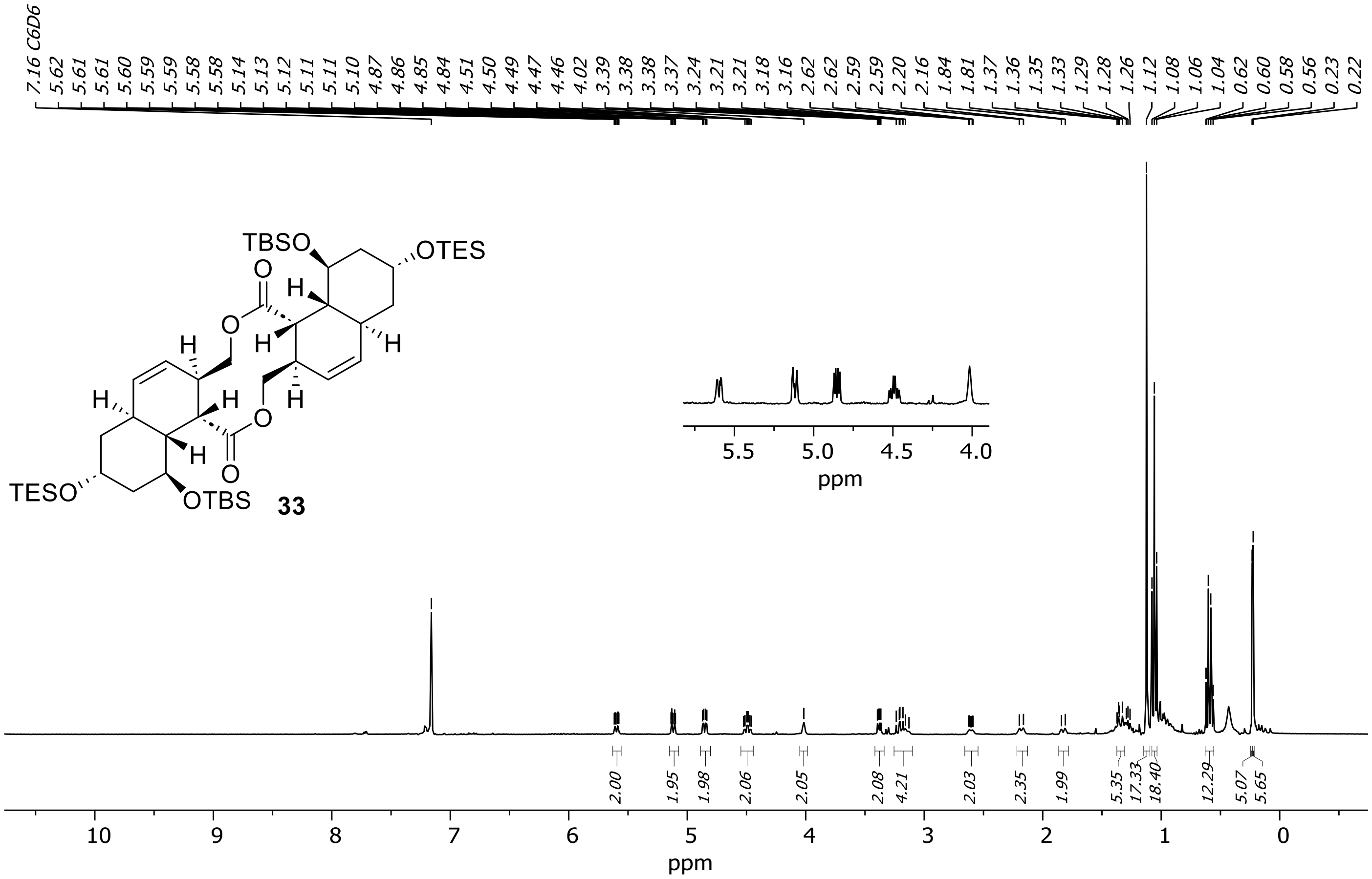

^{13}C-NMR (100.63 MHz, C_6D_6)

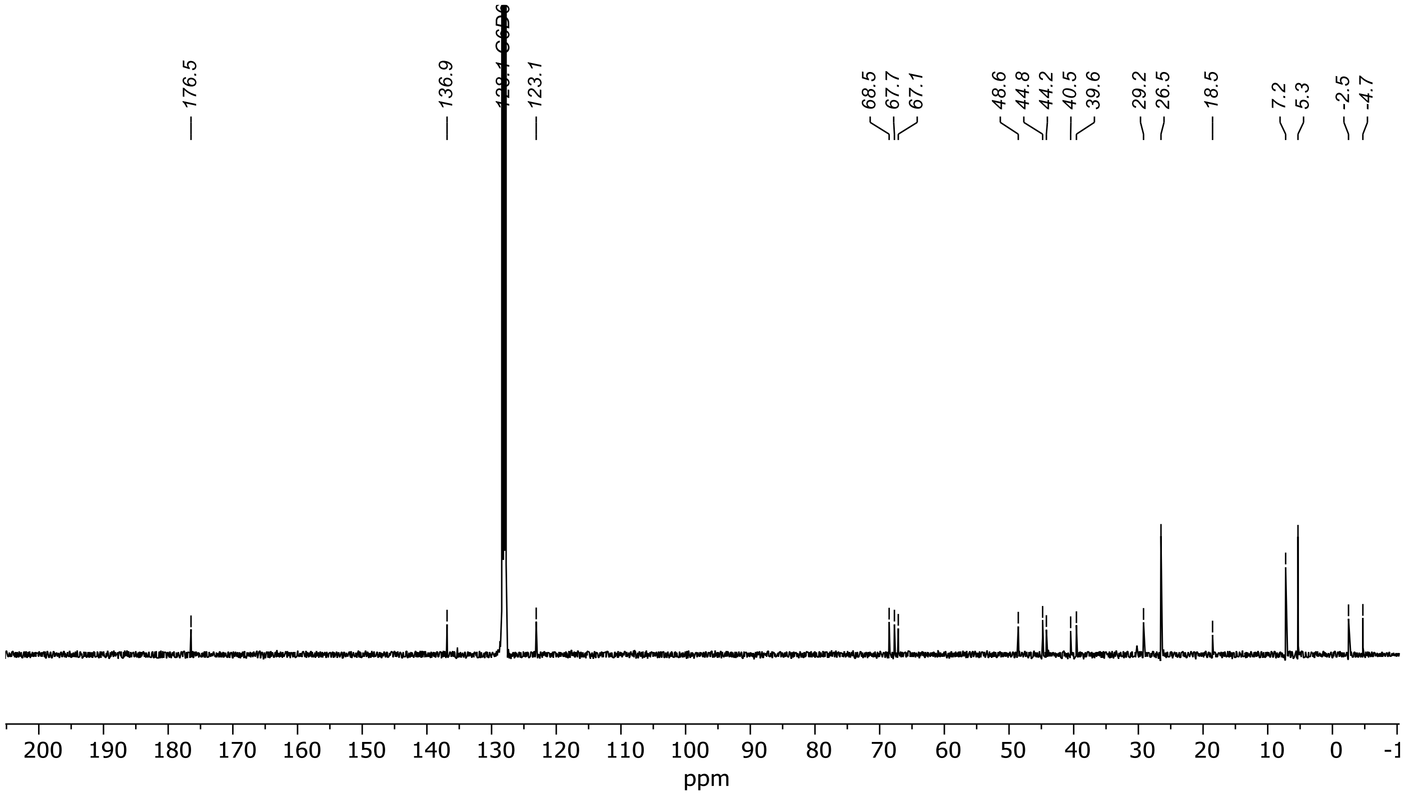

COSY (C_6D_6)

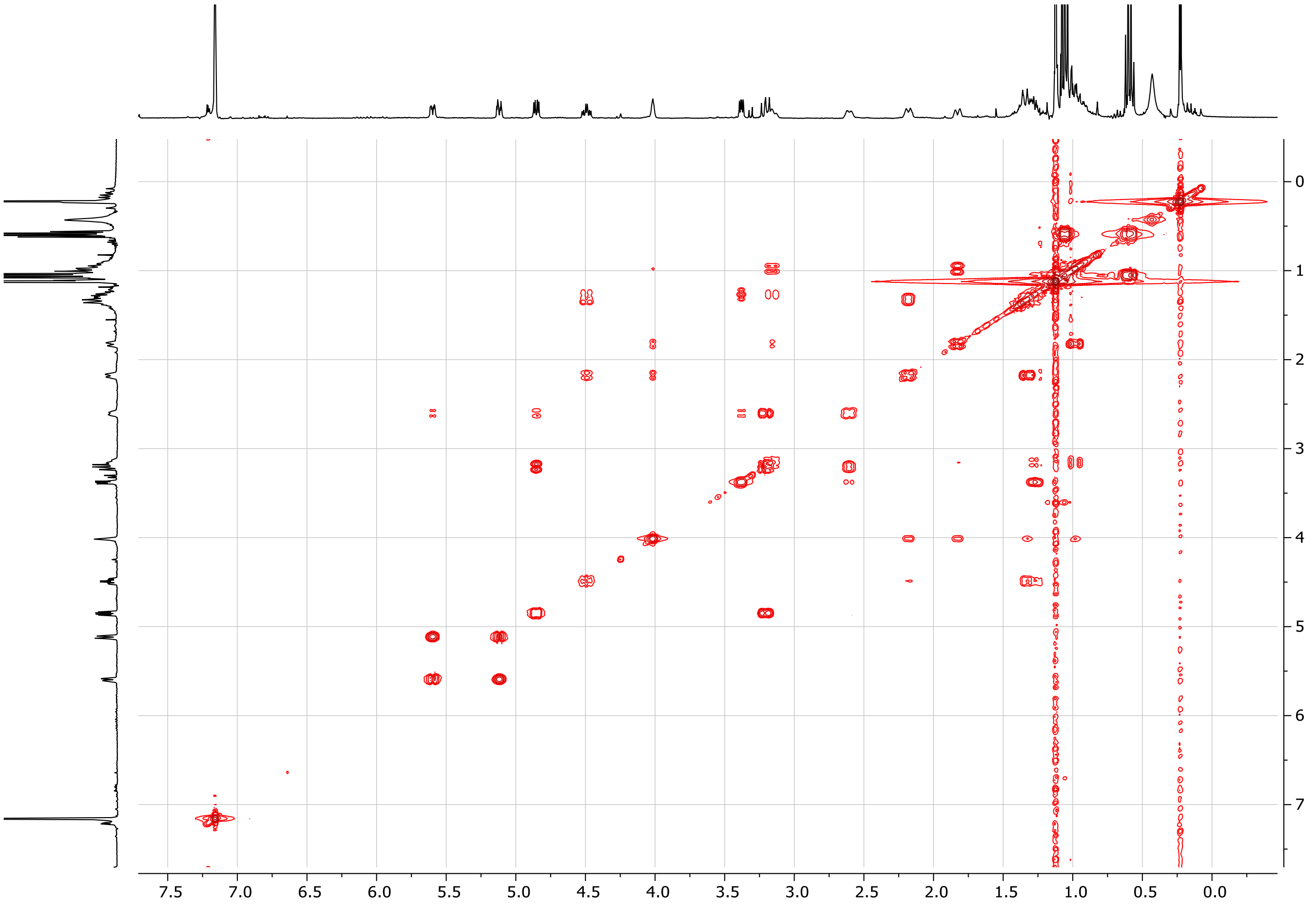

HMBC (C_6D_6)

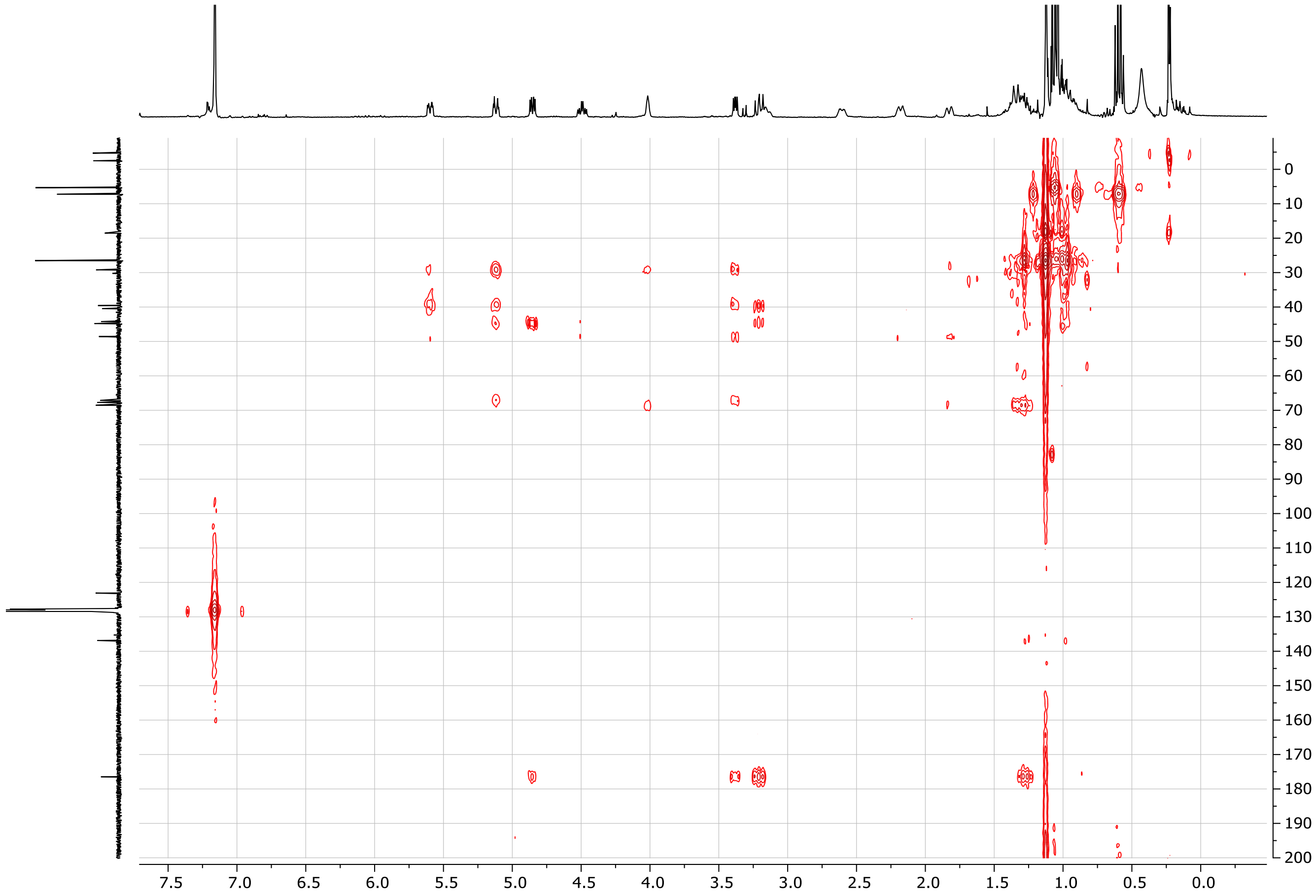

NOE-1D (400.16 MHz, freq. 5.12 ppm, C_6D_6)

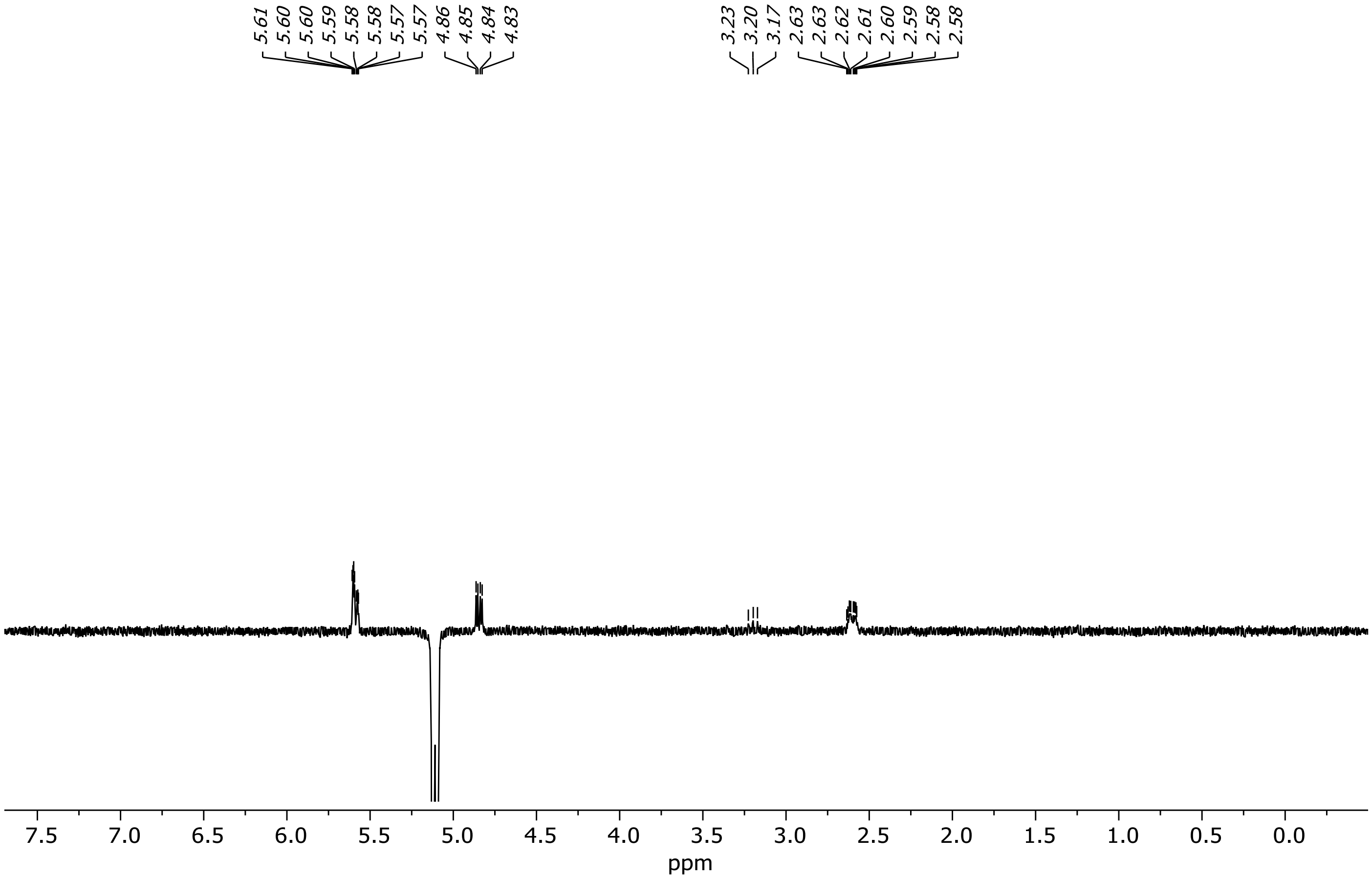

NOE-1D (400.16 MHz, freq. 4.85 ppm, C_6D_6)

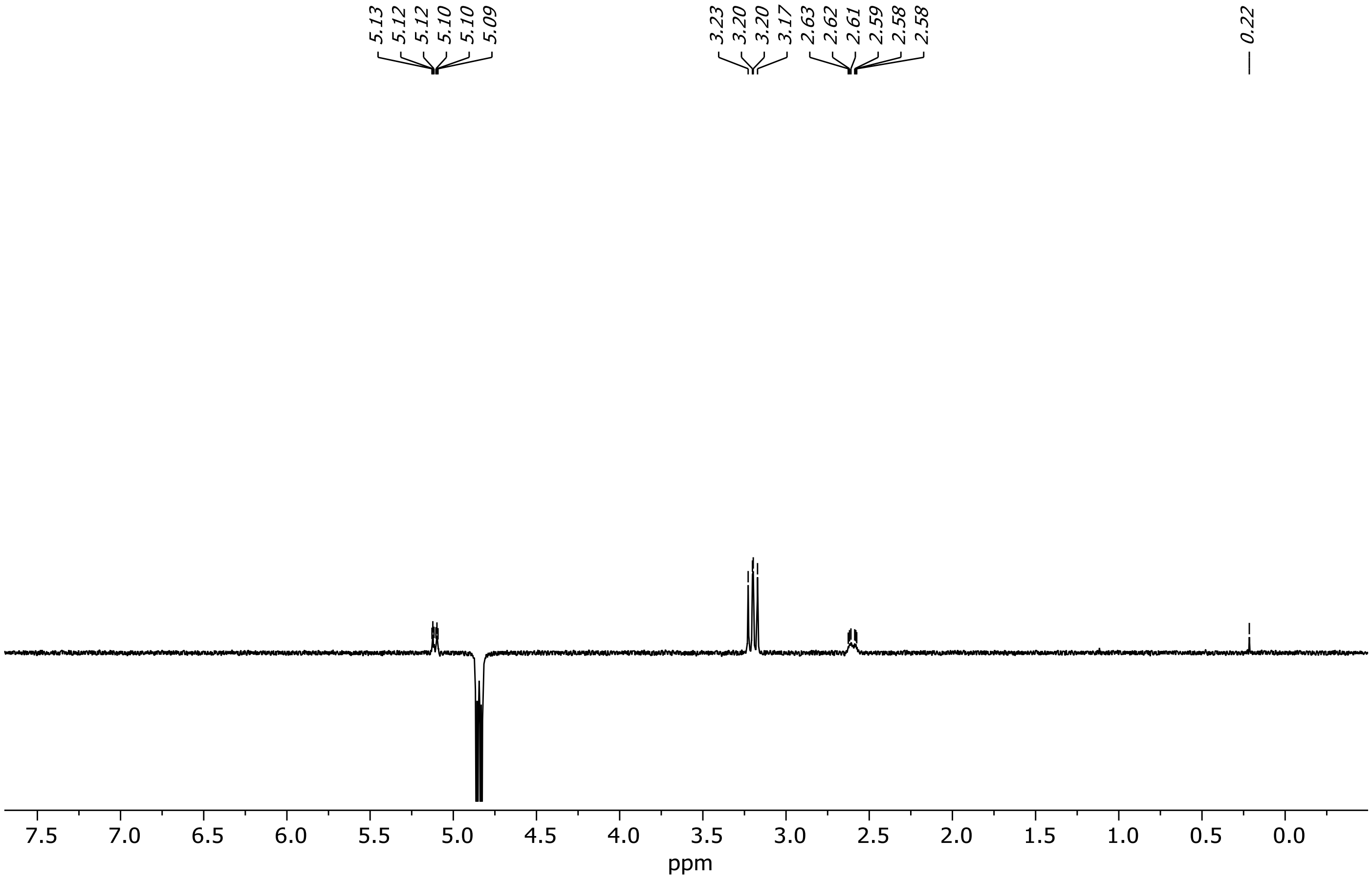

NOE-1D (400.16 MHz, freq. 4.49 ppm, C_6D_6)

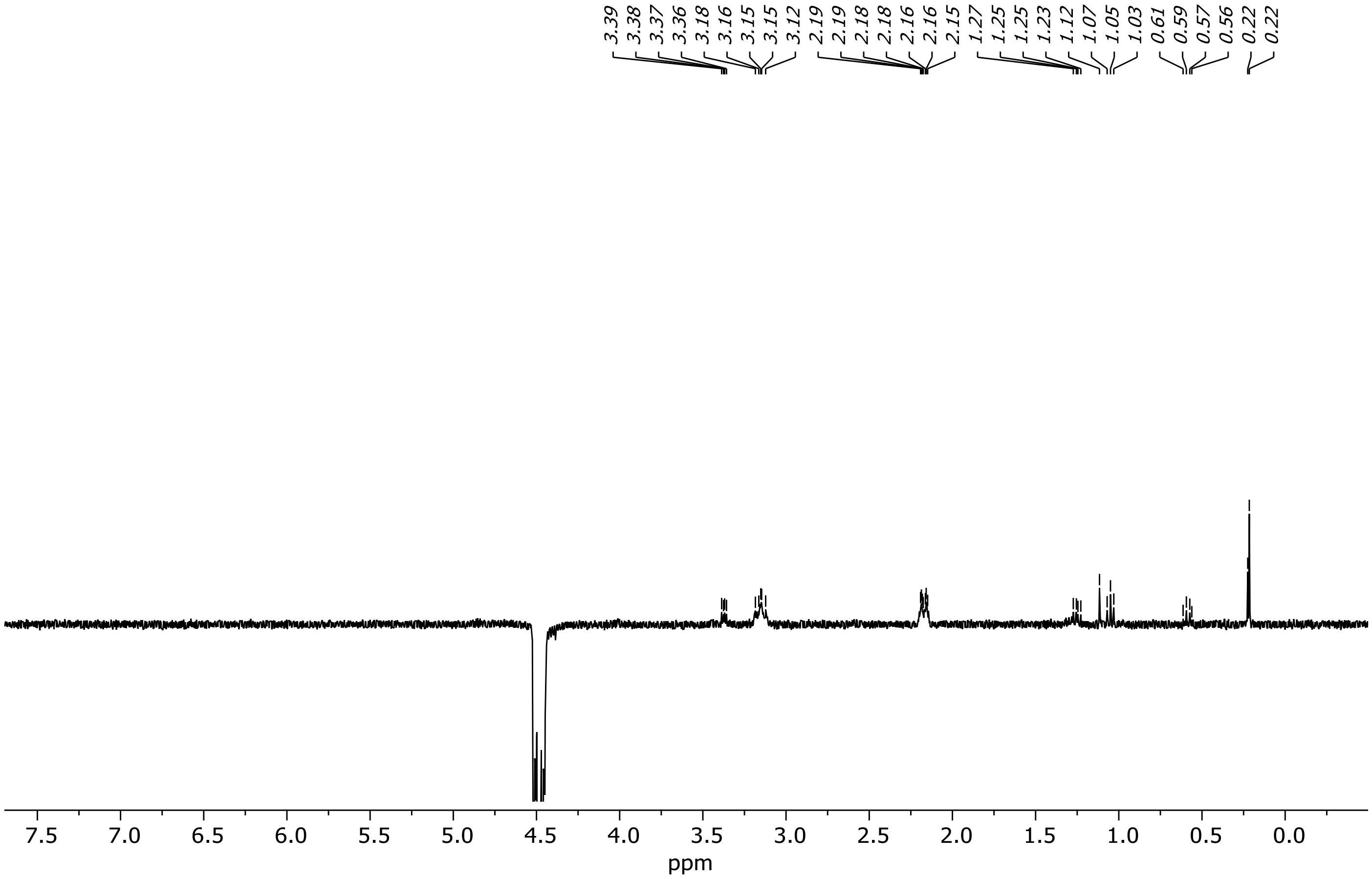

NOE-1D (400.16 MHz, freq. 4.05 ppm, C_6D_6)

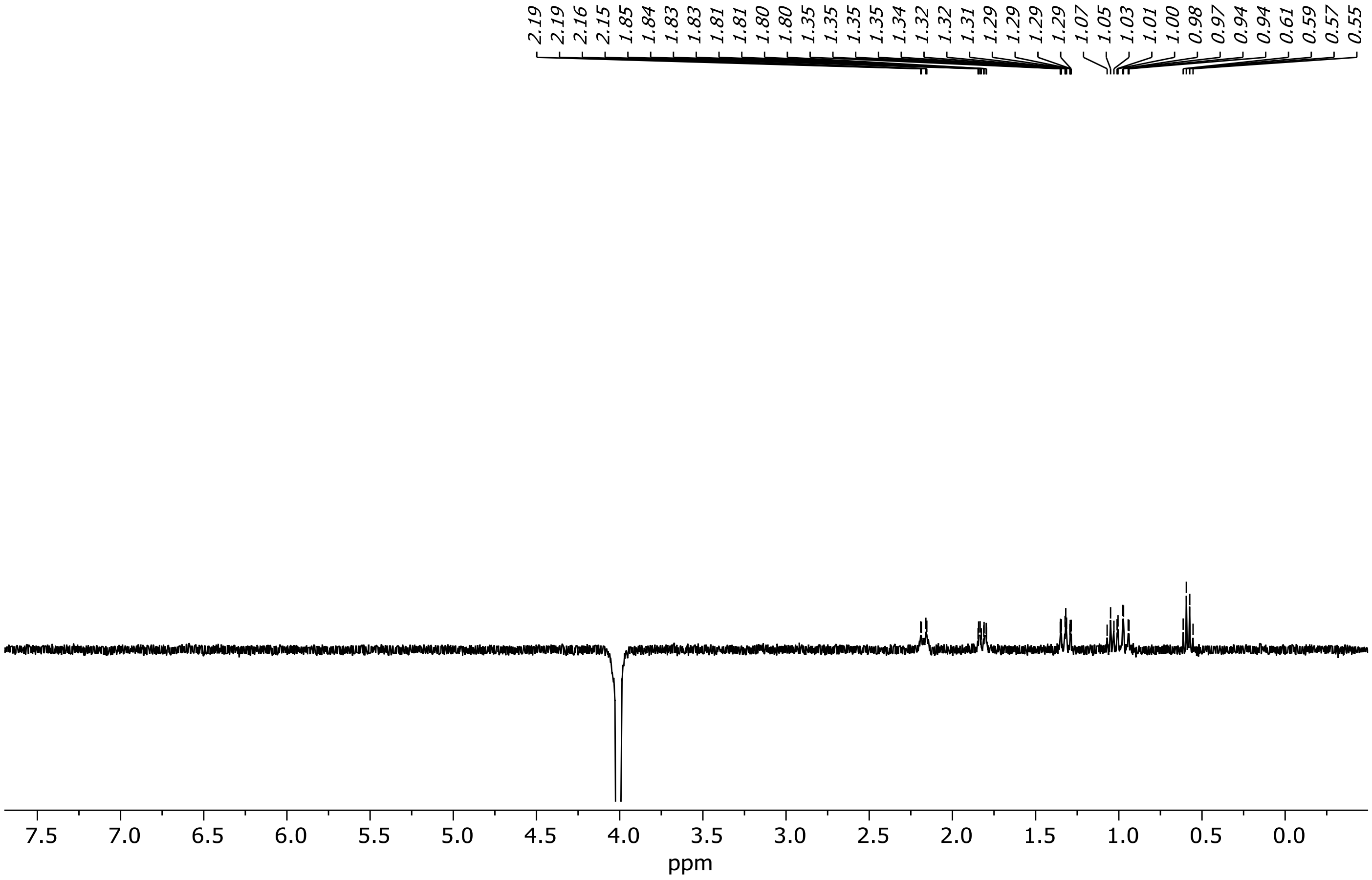

NOE-1D (400.16 MHz, freq. 2.17 ppm, C_6D_6)

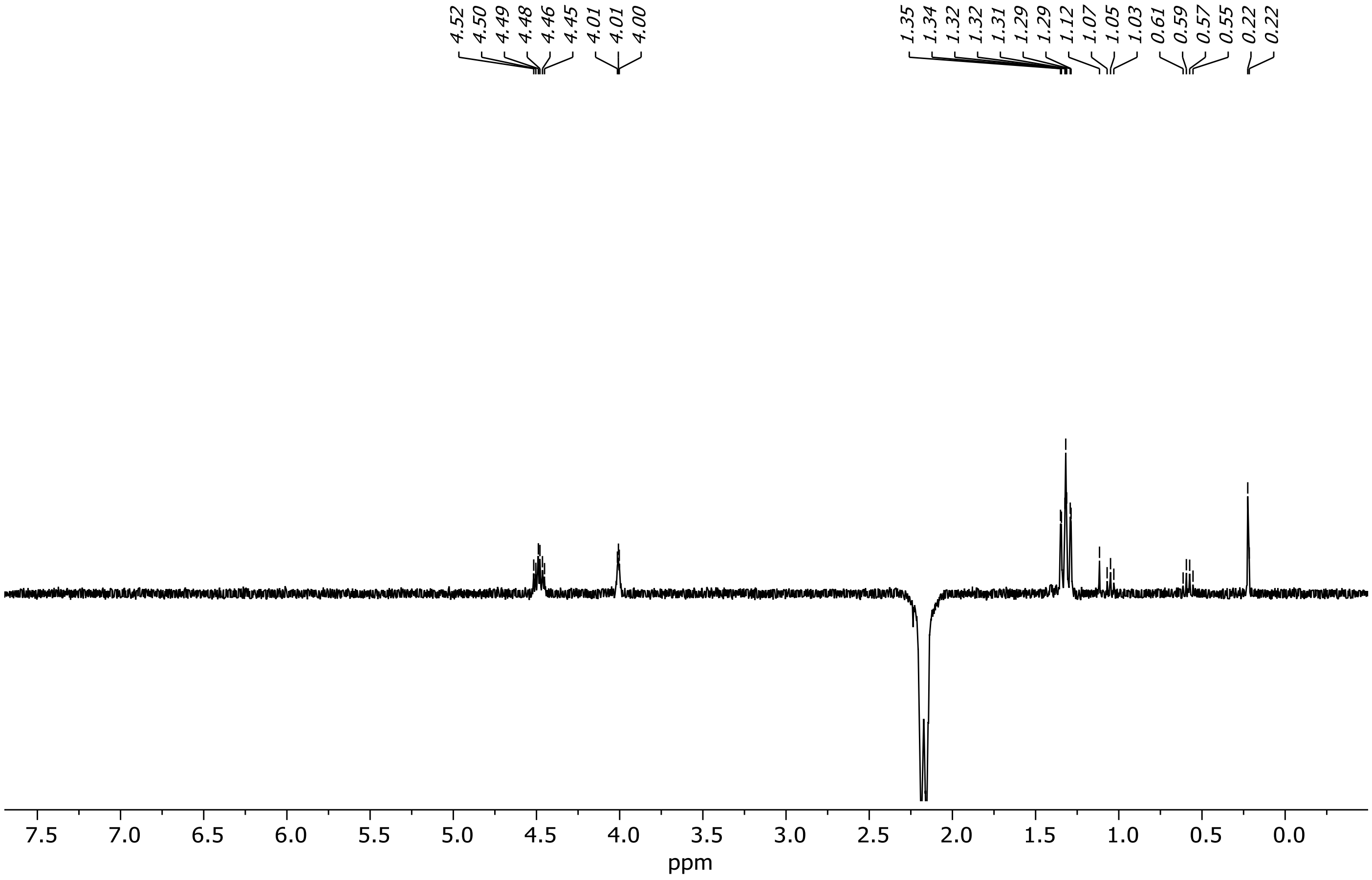

NOE-1D (400.16 MHz, freq. 1.82 ppm, C_6D_6)

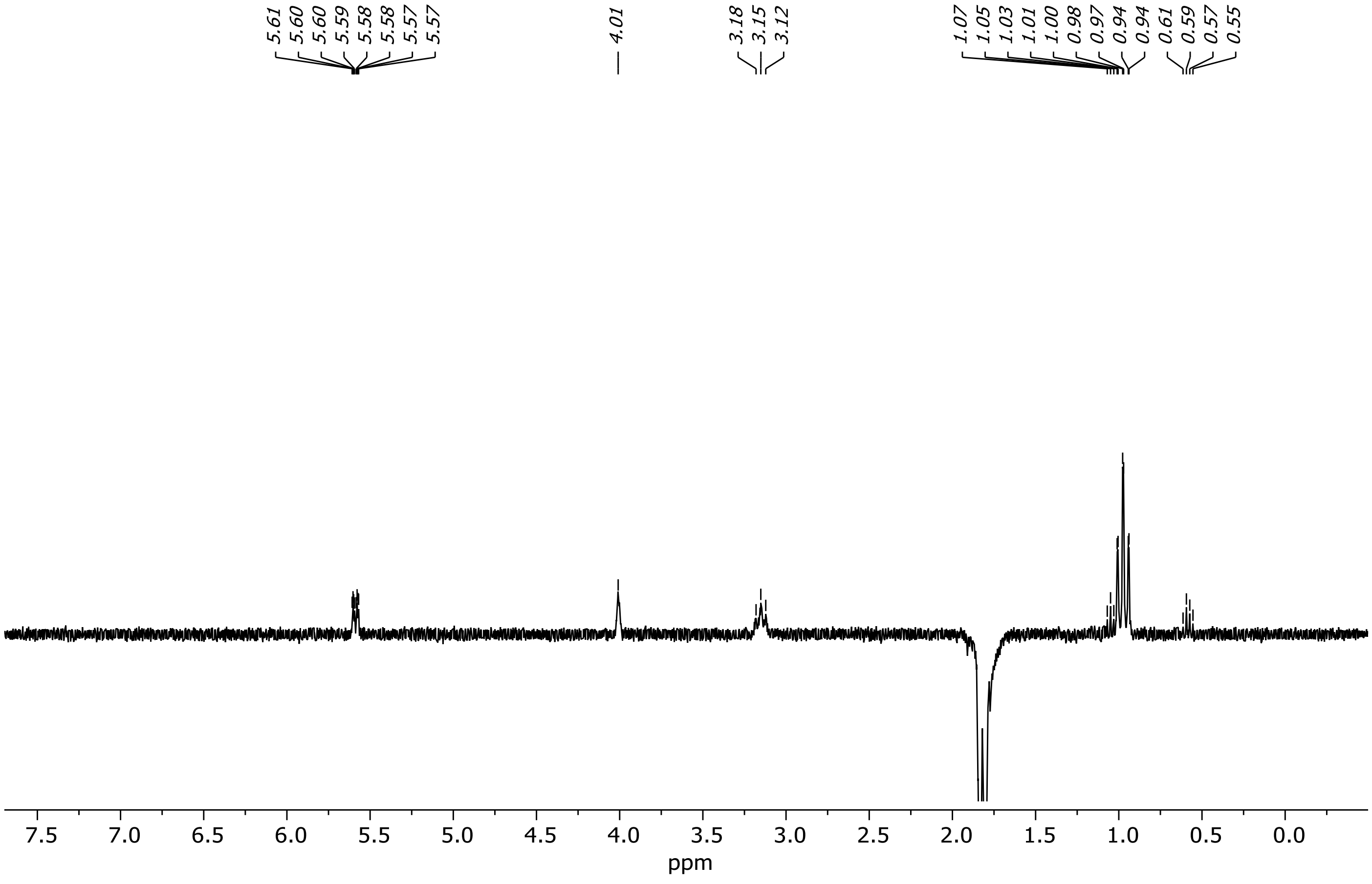

^{1}H-NMR (400.16 MHz, CD_3OD)

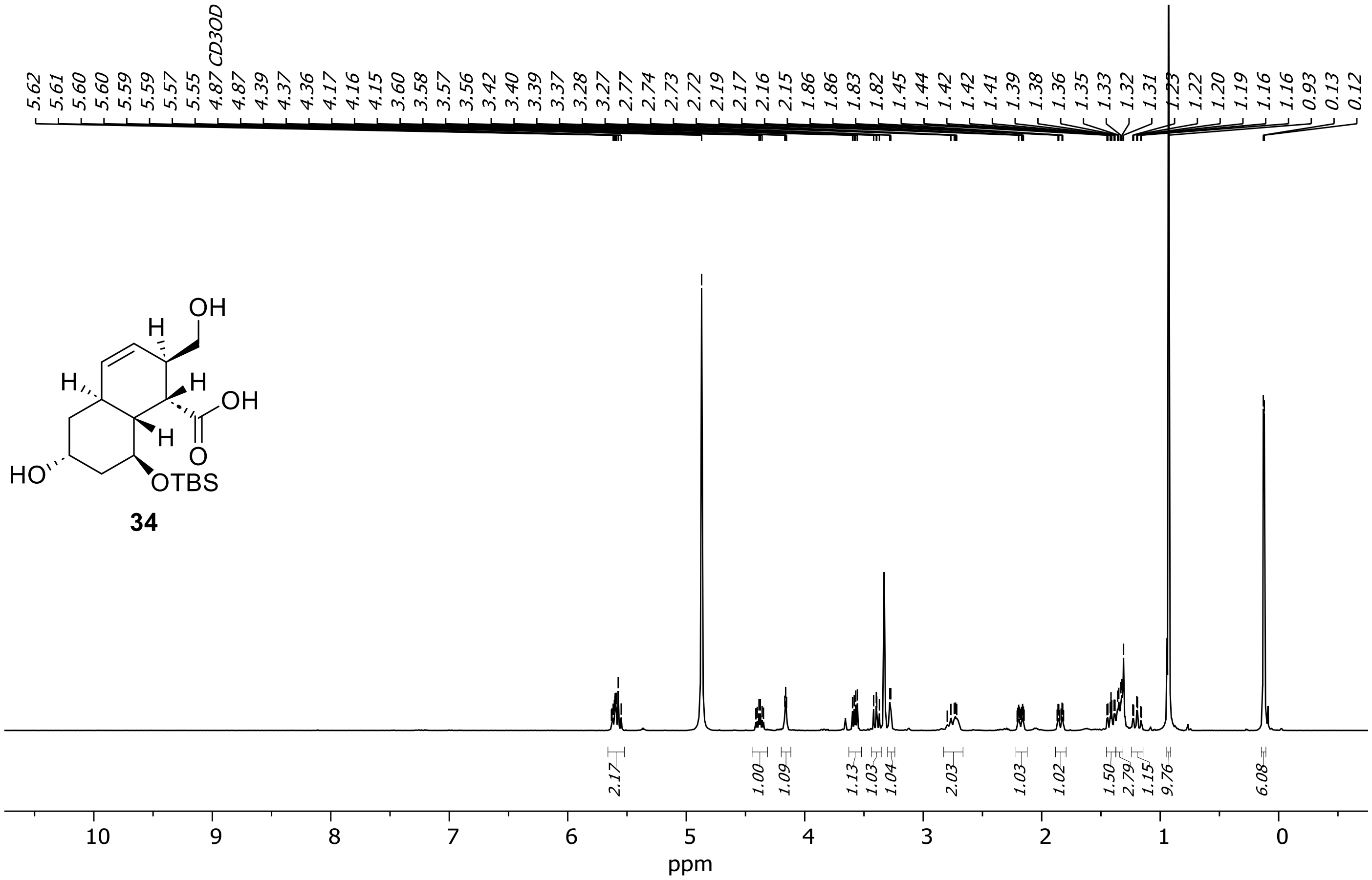

34

^{13}C-NMR (100.63 MHz, CD_3OD)

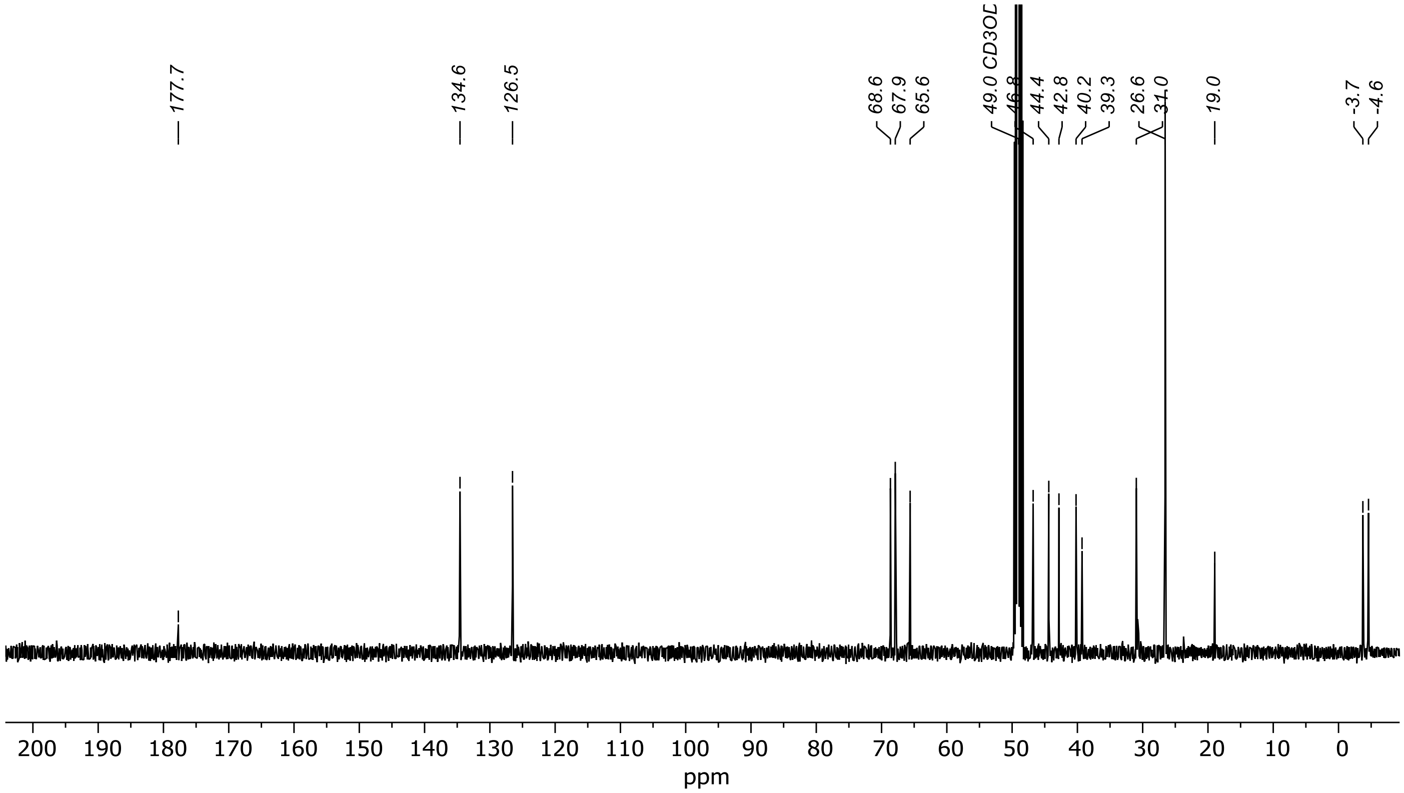

COSY (CD_3OD)

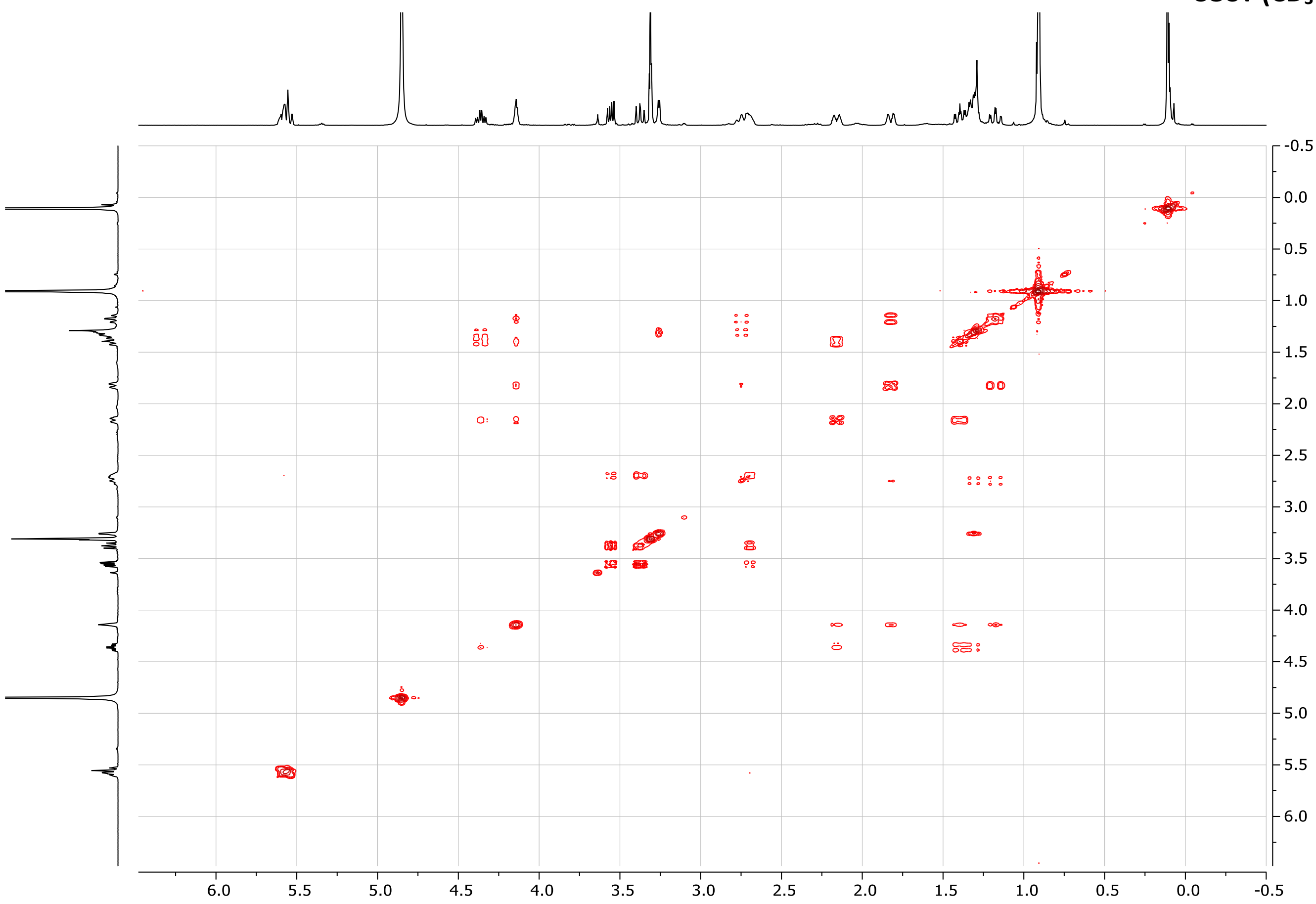

NOE-1D (400.16 MHz, freq. 4.36 ppm, CD_3OD)

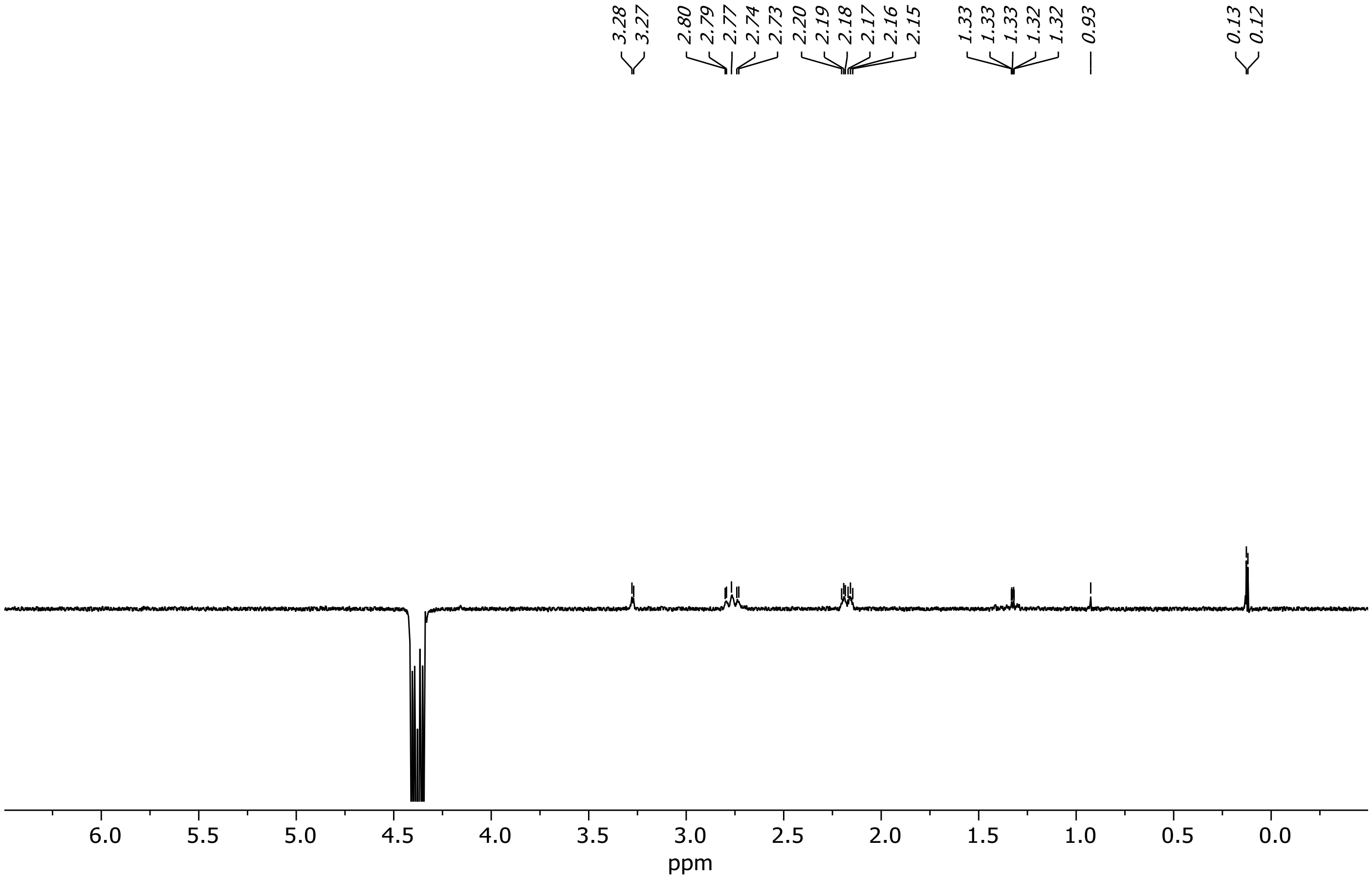

NOE-1D (400.16 MHz, freq. 4.14 ppm, CD_3OD)

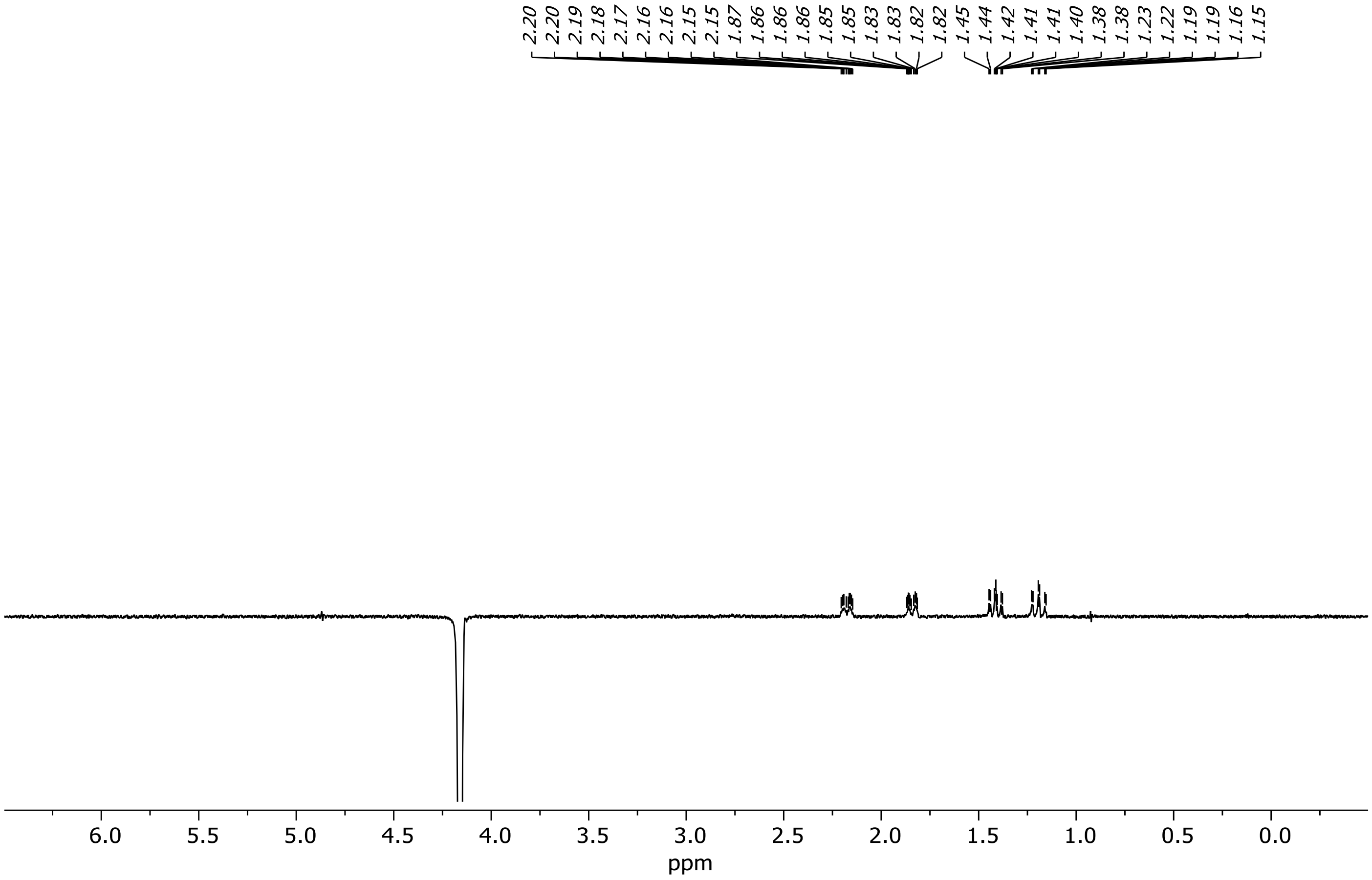

NOE-1D (400.16 MHz, freq. 3.26 ppm, CD_3OD)

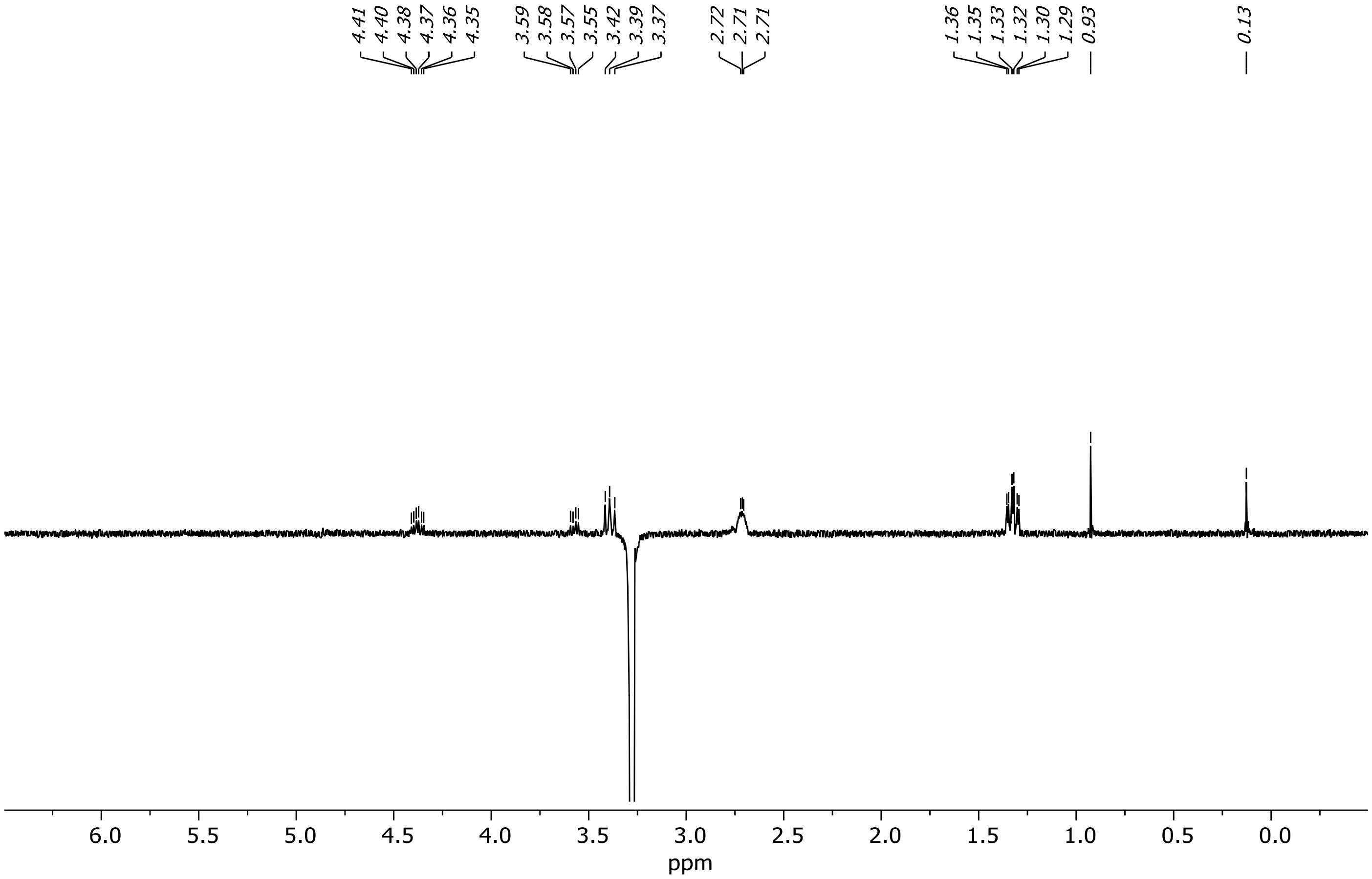

NOE-1D (400.16 MHz, freq. 2.76 ppm, CD_3OD)

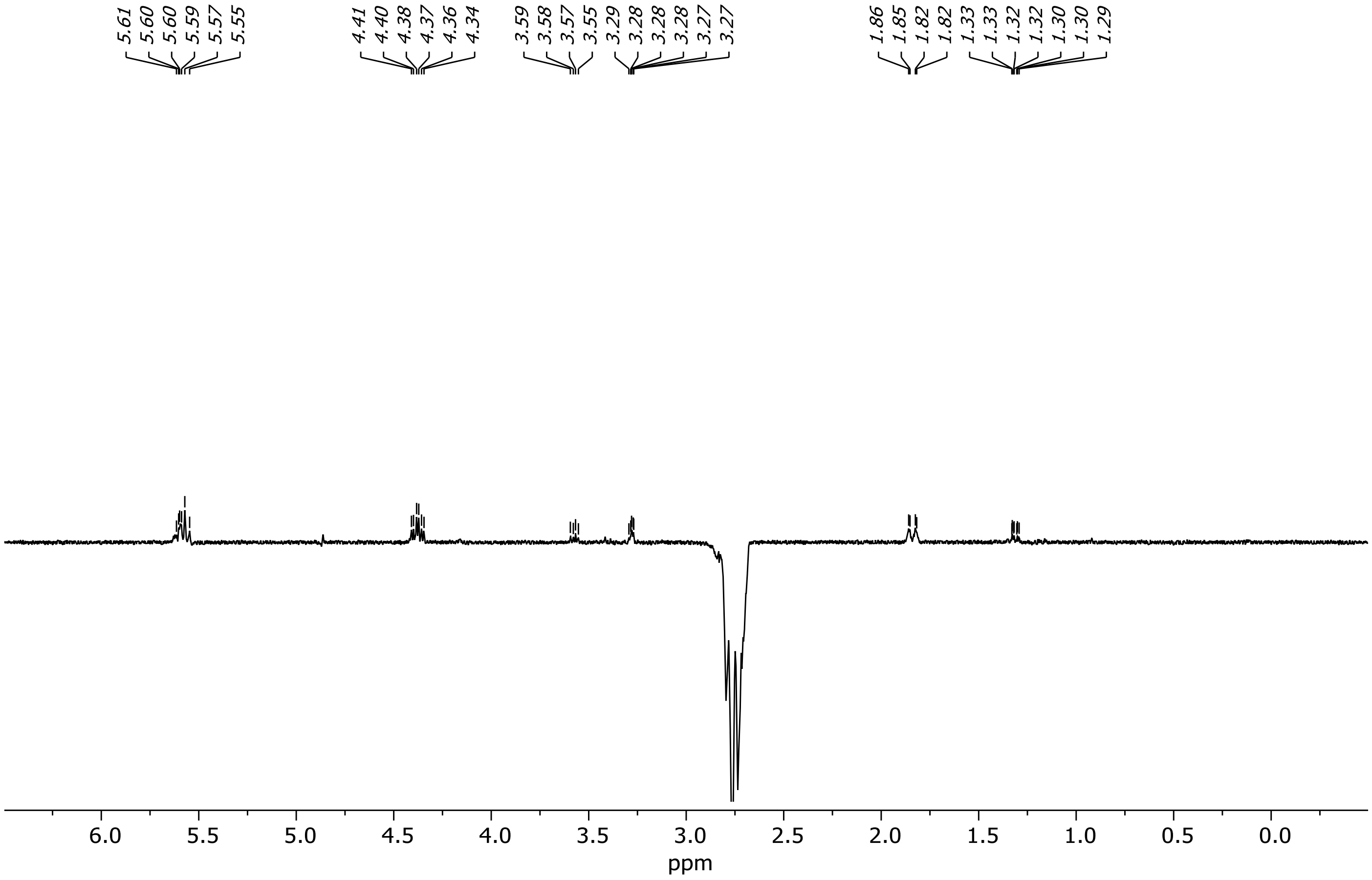

NOE-1D (400.16 MHz, freq. 2.71 ppm, CD_3OD)

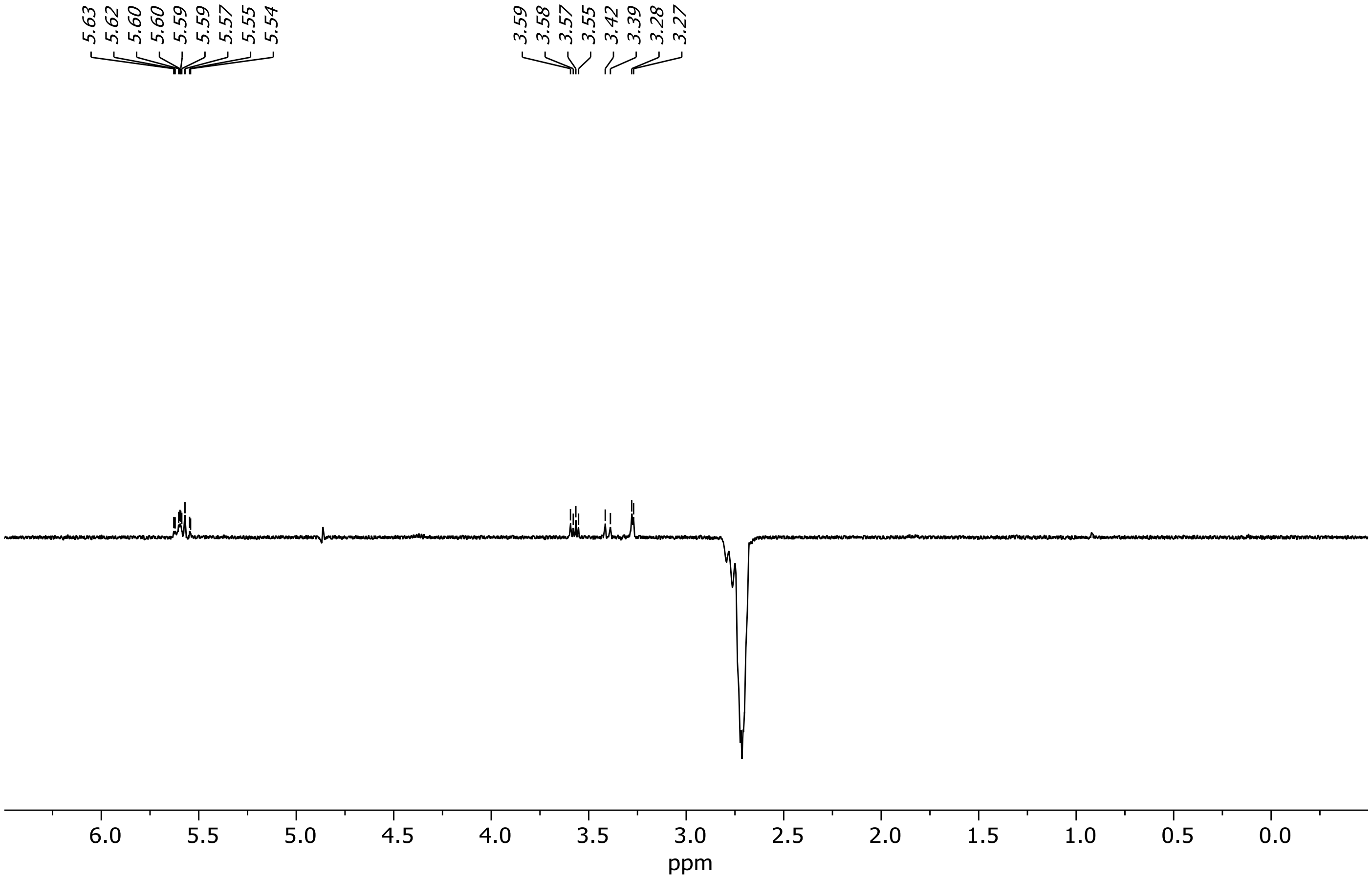

NOE-1D (400.16 MHz, freq. 1.31 ppm, CD_3OD)

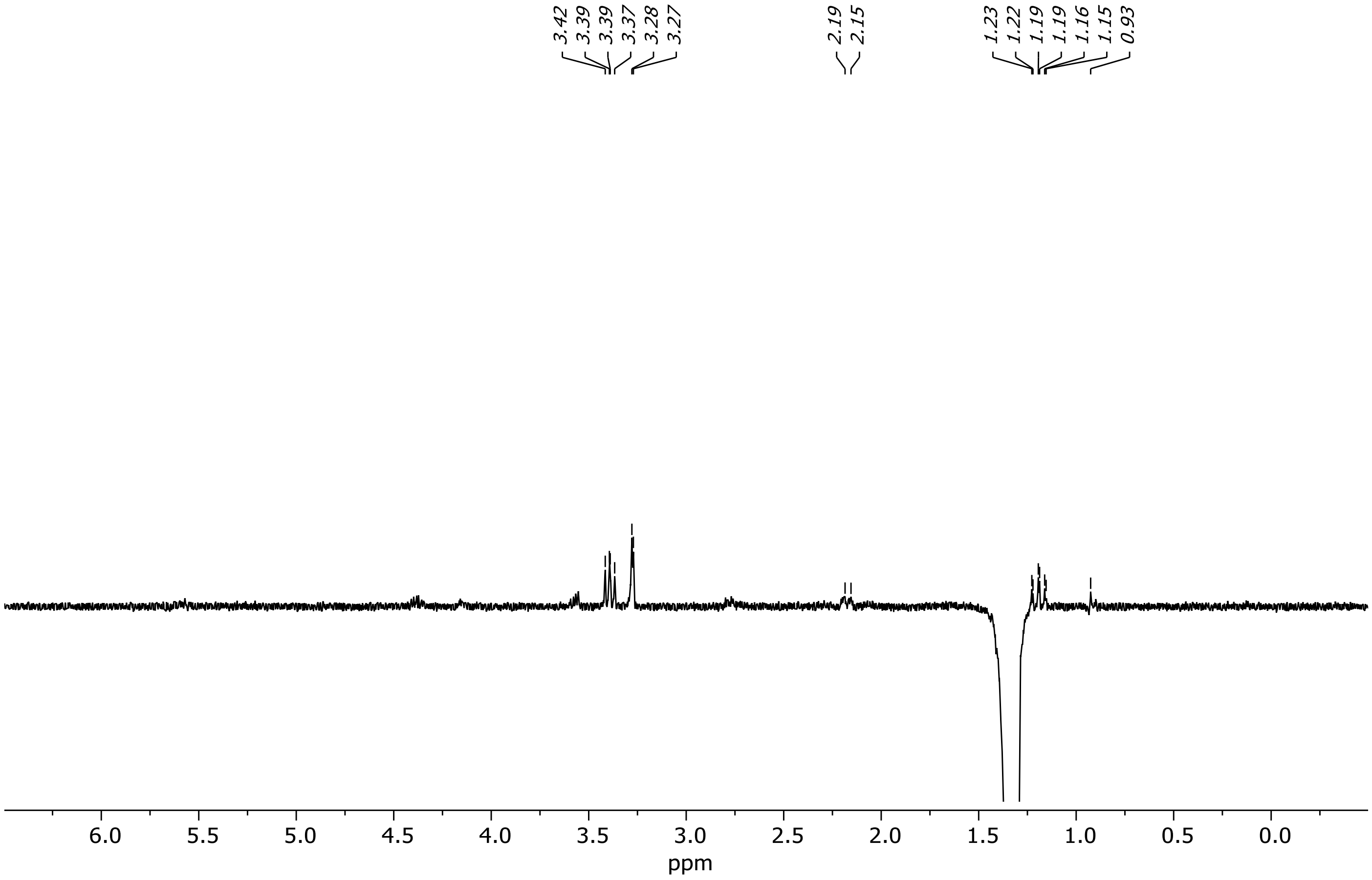

^{1}H-NMR (400.16 MHz, CD_3OD)

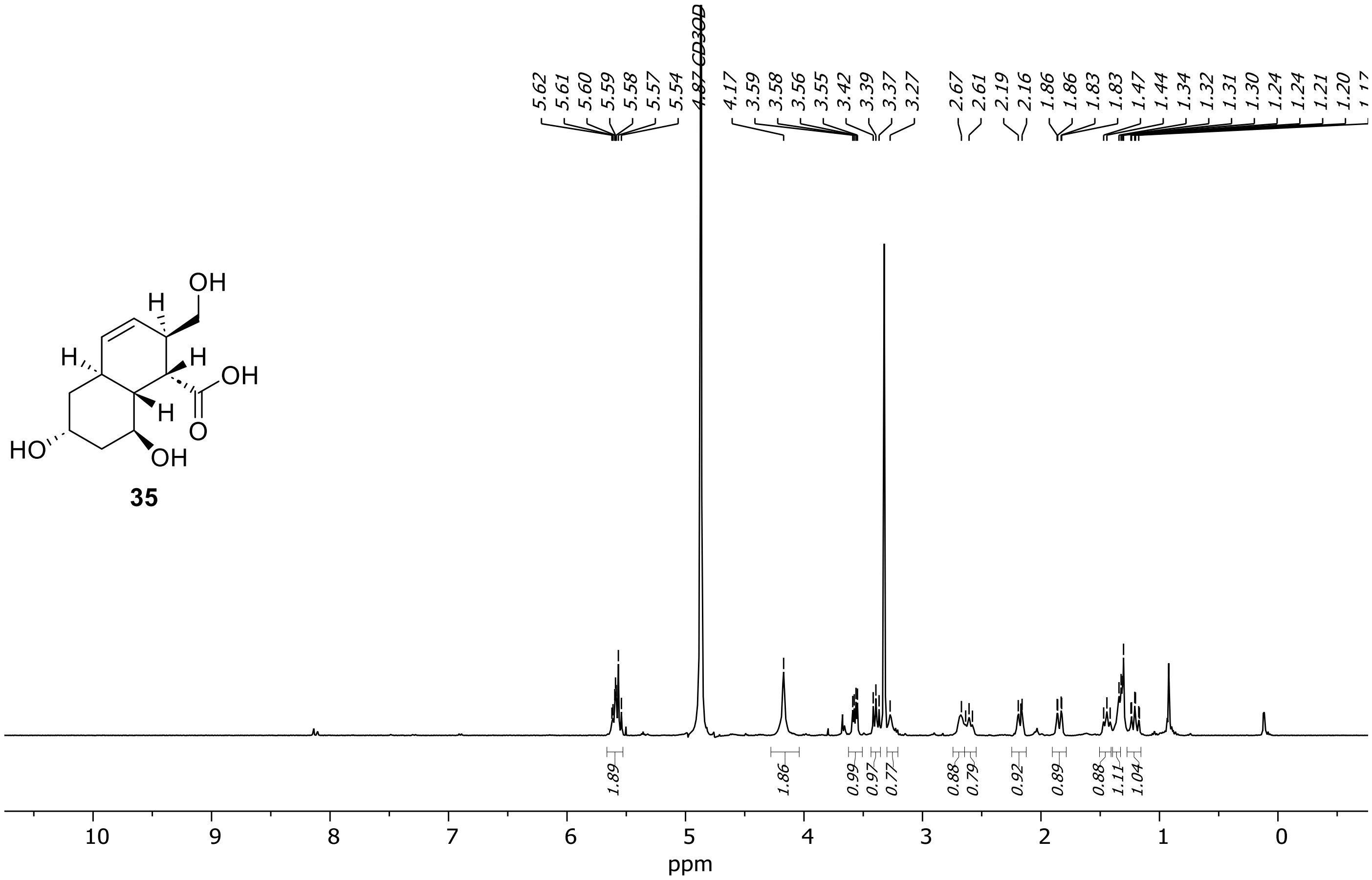

^{13}C-NMR (100.63 MHz, CD_3OD)

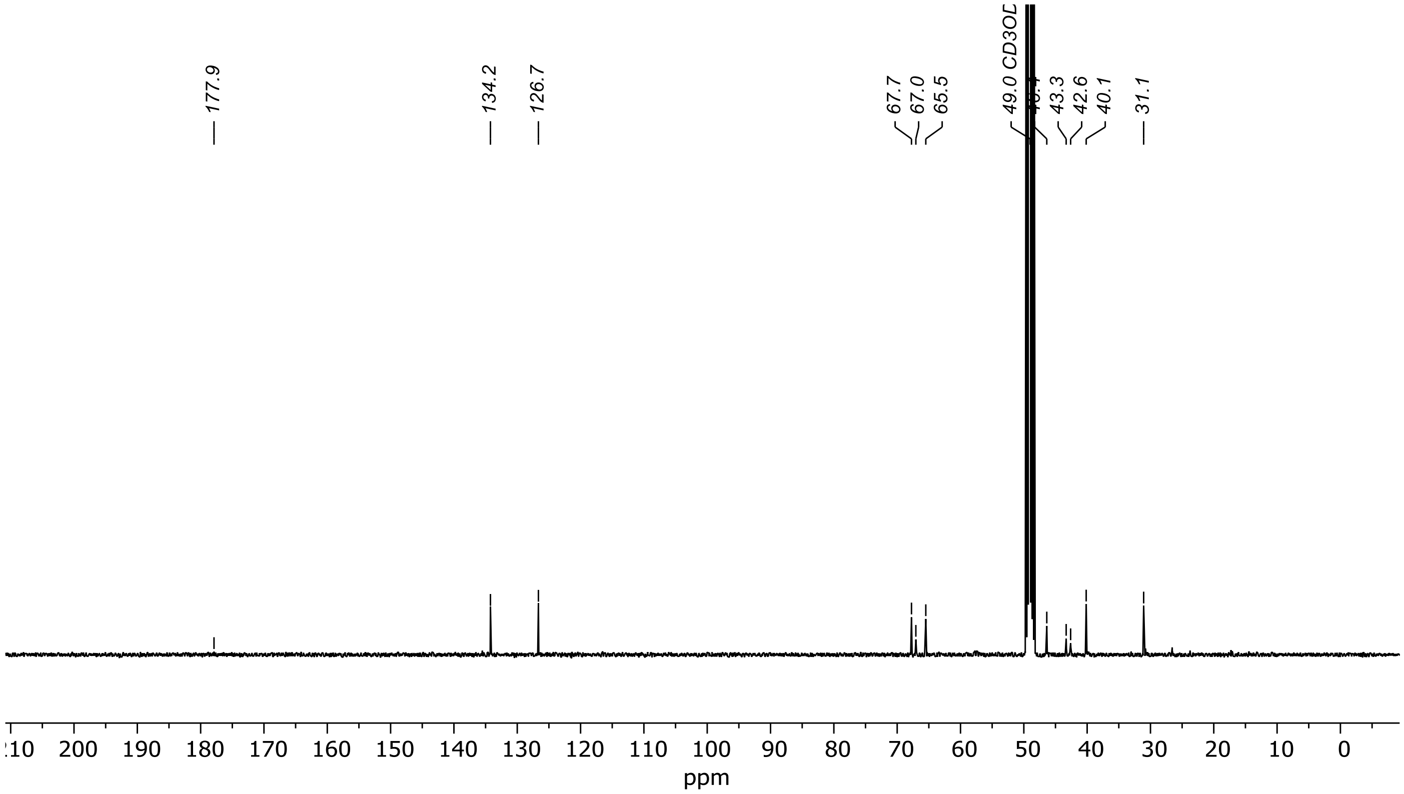

COSY (CD_3OD)

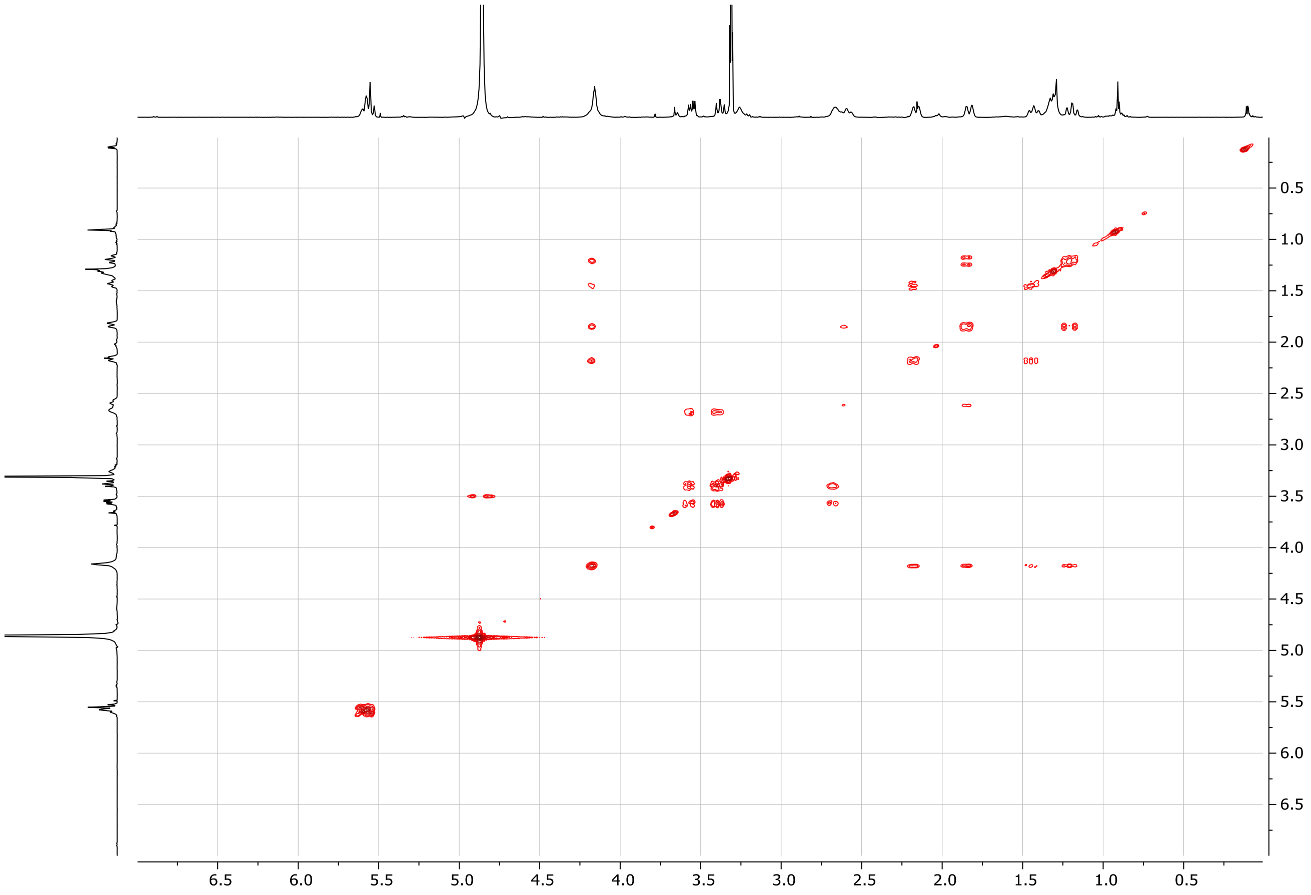

HMBC (CD_3OD)

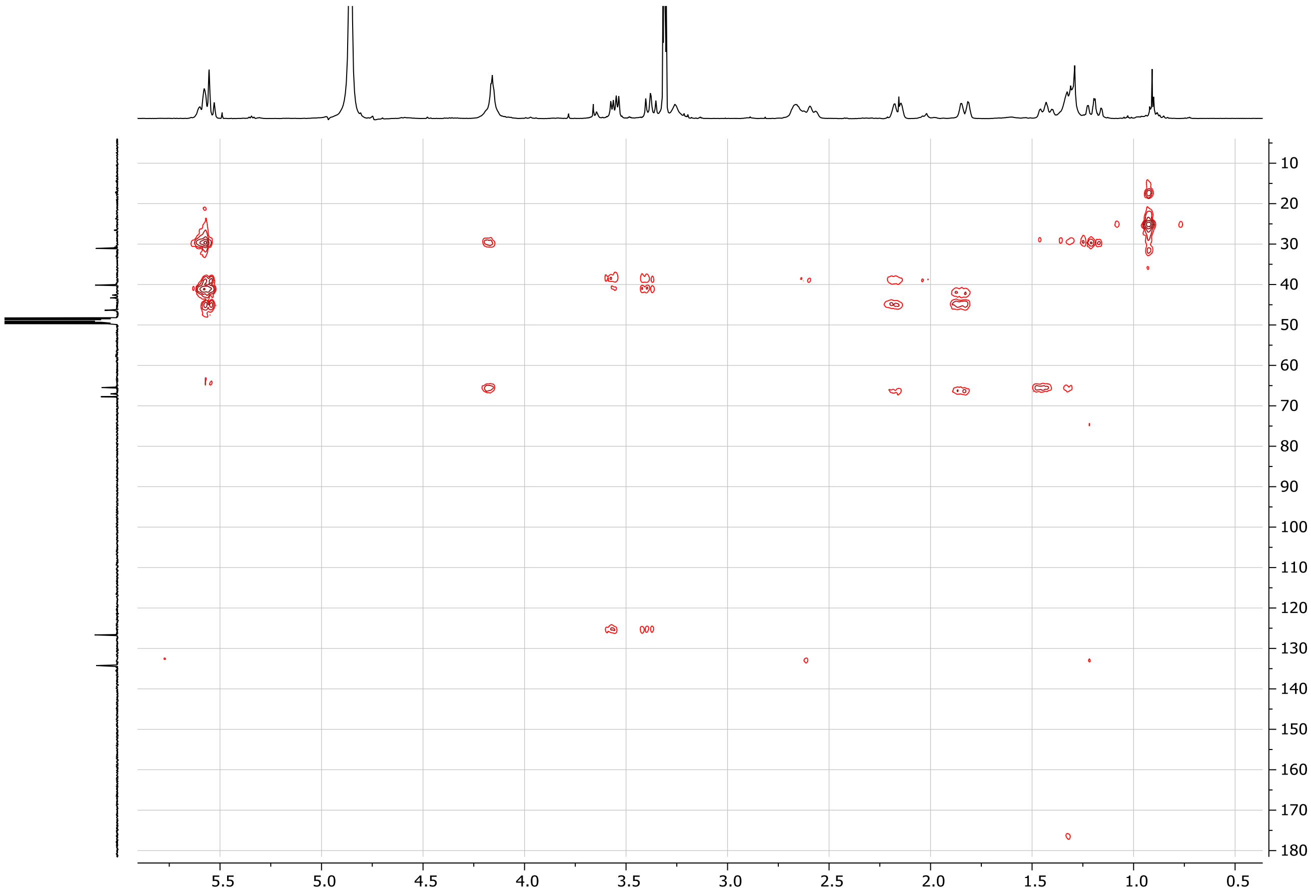

Supplement: Supplementary file 1 — ol4c02239_si_001.pdf [file ol4c02239_si_001.pdf]
